# Clinical updates on gliomas and implications of the 5th edition of the WHO classification of central nervous system tumors

**DOI:** 10.3389/fonc.2023.1131642

**Published:** 2023-03-14

**Authors:** Xiaopeng Guo, Yixin Shi, Delin Liu, Yilin Li, Wenlin Chen, Yaning Wang, Yuekun Wang, Hao Xing, Yu Xia, Junlin Li, Jiaming Wu, Tingyu Liang, Hai Wang, Qianshu Liu, Shanmu Jin, Tian Qu, Siying Guo, Huanzhang Li, Tianrui Yang, Kun Zhang, Yu Wang, Wenbin Ma

**Affiliations:** ^1^Department of Neurosurgery, Center for Malignant Brain Tumors, National Glioma MDT Alliance, Peking Union Medical College Hospital, Chinese Academy of Medical Sciences and Peking Union Medical College, Beijing, China; ^2^China Anti-Cancer Association Specialty Committee of Glioma, Beijing, China; ^3^Eight-year Medical Doctor Program, Chinese Academy of Medical Sciences and Peking Union Medical College, Beijing, China; ^4^’4+4’ Medical Doctor Program, Chinese Academy of Medical Sciences and Peking Union Medical College, Beijing, China

**Keywords:** glioma, WHO classification of central nervous system tumors, molecular alteration, integrated diagnosis, glioblastoma

## Abstract

**Background:**

The 5th edition of the World Health Organization (WHO) classification of central nervous system tumors incorporated specific molecular alterations into the categorization of gliomas. The major revision of the classification scheme effectuates significant changes in the diagnosis and management of glioma. This study aimed to depict the clinical, molecular, and prognostic characteristics of glioma and its subtypes according to the current WHO classification.

**Methods:**

Patients who underwent surgery for glioma at Peking Union Medical College Hospital during 11 years were re-examined for tumor genetic alterations using next-generation sequencing, polymerase chain reaction-based assay, and fluorescence *in situ* hybridization methods and enrolled in the analysis.

**Results:**

The enrolled 452 gliomas were reclassified into adult-type diffuse glioma (ntotal=373; astrocytoma, n=78; oligodendroglioma, n=104; glioblastoma, n=191), pediatric-type diffuse glioma (ntotal=23; low-grade, n=8; high-grade, n=15), circumscribed astrocytic glioma (n=20), and glioneuronal and neuronal tumor (n=36). The composition, definition, and incidence of adult- and pediatric-type gliomas changed significantly between the 4th and the 5th editions of the classification. The clinical, radiological, molecular, and survival characteristics of each subtype of glioma were identified. Alterations in CDK4/6, CIC, FGFR2/3/4, FUBP1, KIT, MET, NF1, PEG3, RB1, and NTRK2 were additional factors correlated with the survival of different subtypes of gliomas.

**Conclusions:**

The updated WHO classification based on histology and molecular alterations has updated our understanding of the clinical, radiological, molecular, survival, and prognostic characteristics of varied subtypes of gliomas and provided accurate guidance for diagnosis and potential prognosis for patients.

## 1 Introduction

Glioma is the most prevalent primary central nervous system (CNS) malignant tumor (1). Despite the combination of surgery, radiotherapy, chemotherapy, targeted therapy, and tumor-treating fields treatment, the overall survival (OS) of glioma remains dismal, with a 5-year survival rate of 7% for the most aggressive subtype of glioblastoma (2). Accurate tumor classification is the basis for individualized treatment selection and prediction of treatment response and patient prognosis (3). The 4th edition of the World Health Organization (WHO) classification of CNS tumors (WHO CNS4 classification) referred mostly to tumor histology (4). However, certain molecular alterations were recently have been reported to be associated with variable patient survival (5–7). Based on these findings, the newly published WHO CNS5 classification integrated specific molecular alterations with tumor histology in classifying CNS tumors, thereby emphasizing the impact of molecular changes on tumor progression, optimal treatment selection, and prognostic prediction (8).

Developed from the WHO CNS4 classification, the current edition reorganized gliomas into adult-type diffuse gliomas, pediatric-type low-grade and high-grade diffuse gliomas, glioneuronal and neuronal tumors, circumscribed astrocytic gliomas, and ependymal tumors (8). Some of the major changes were the re-defining of glioblastoma, IDH-wildtype, WHO grade 4 (6, 7), the re-grading of astrocytoma, IDH-mutant (5), and the systemic categorization of pediatric-type diffuse gliomas based on their well-established genetic alterations (9–11). However, our understanding of the categorization of gliomas and their diagnostic, therapeutic, and prognostic characteristics is limited since it was all from the research based on the previous classification scheme (12, 13). Recently, several studies have attempted to explore and update the characteristics of gliomas according to the WHO CNS5 classification, with conflicting results (14–18).

A detailed understanding of the categorization changes of gliomas, clinical characteristics of different subtypes, survival implications, and predictive ability of molecular features based on the current classification are still controversial but valuable. The present study aimed to subgroup the gliomas in the real world according to the current WHO classification and depict the clinical presentations, radiological features, pathological characteristics, and molecular alterations of different subtypes, as well as assess patient survival and the predictive values of molecular alterations for prognosis. To achieve the above objectives, we analyzed the data of patients with gliomas at the Department of Neurosurgery of Peking Union Medical College Hospital (PUMCH) over 11 years in order to provide a solid basis for the clinical categorization and decision-making of malignant gliomas.

## 2 Materials and methods

### 2.1 Study participants

A total of 605 patients who underwent surgery for glioma at PUMCH Neurosurgery from January 2011–2022 were screened. Among them, 452 patients with available and integrated clinical data were included for analyses. All enrolled patients signed inform consent, and the study was approved by the institutional review board of Peking Union Medical College Hospital (Approved ID of ethic committee:S-424).

### 2.2 Data acquisition

Clinical data were collected from the medical records of all patients regarding age at diagnosis, sex, body mass index, clinical symptoms, disease duration before admission, baseline Karnofsky Performance Scale (KPS) score, and the extent of surgical resection (ESR). The ESR included gross total resection (no radiographic evidence of residual tumor after surgery), subtotal resection (positive radiographic evidence of residual tumor), and biopsy. The OS was defined as the time span from the date of operation to the date of death or the last follow-up (censored).

The radiological characteristics of the patients with complete sets of preoperative and follow-up magnetic resonance imaging (MRI) sequences were collected. Data on variables, such as tumor location, maximal tumor diameter, number of tumors, tumor contact with functional areas, intensity on T1WI and T2WI, and presence of contrast enhancement and intratumoral necrosis, were extracted. Histological data on Ki-67 index and histological WHO grade were obtained from the pathological studies at our institute.

A total of 60 molecular markers of interest, including EGFR, TERT, CDKN2A/B, MYB, MYBL1, CDK4, CDK6, CIC, FGFR2/3/4, KIT, KMT5B, MET, MGMT, NF1, NTRK2, PEG, PTEN, RB1, and chromosome copy number variations, were analyzed in this study using the next-generation sequencing, the polymerase chain reaction-based assay, and fluorescence *in situ* hybridization methods. These markers were selected based on recent studies, with the initial perspective to differentiate the subtypes of gliomas according to the updated WHO CNS5 classification or to predict patient prognosis.

### 2.3 Classification of gliomas by the WHO CNS5 scheme

Glioblastomas were defined as grade-4 IDH1/2-wildtype diffuse gliomas with microvascular proliferation and/or intratumoral necrosis or grade 2-3 IDH1/2-wildtype astrocytic gliomas with at least one of the following molecular features: telomerase reverse transcriptase (TERT) promoter mutation, epidermal growth factor receptor (EGFR) amplification, or concomitant gain of chromosome 7 and loss of chromosome 10 (+7/-10 copy number changes). The term IDH-mutant glioblastoma was changed to WHO grade 4 astrocytoma, IDH-mutant according to the current classification. Astrocytoma consisted only of IDH-mutant diffuse glioma and was sub-divided into three grades (2–4) according to the histologic findings and the status of CDKN2A/B homozygous deletion. IDH-wildtype astrocytoma was reclassified as either molecular glioblastoma with specific molecular features or as other subtypes of gliomas. The pediatric-type diffuse gliomas were sub-grouped into low- and high-grade subtypes based on their genetic alterations (e.g., MYB- or MYBL1-altered for low-grade gliomas, and H3K27-altered for high-grade gliomas).

### 2.4 Statistical analyses

For clinical, radiological, and pathological data, categorical variables were presented as numbers and percentages, and continuous variables were presented as the means ± standard deviations (SDs) or medians plus interquartile range according to the data distribution. The comparison of categorical variables was performed using the chi-squared test. Student’s t-test was used to assess the differences between normally distributed continuous variables, while Mann–Whitney U test was used with variables that failed the normality test. Most parameters were analyzed for all patients enrolled in this study. However, for several variables, only the patients who had complete data available were enrolled for analysis. Statistical significance was considered when P<0.05. Sankey’s diagram was used to visualize the changes in the subtypes of gliomas from WHO CNS4 to CNS5 classification. The waterfall heatmap was to illustrate the molecular alterations in different subtypes of gliomas. The Median OS (mOS) and the 95% confidence interval (95% CI) were calculated for patients with different subtypes of gliomas and for those with distinct molecular features. Kaplan–Meier curves were drawn to illustrate the OS, and a log-rank P<0.05 indicated a significant survival difference between groups. SPSS (version 26.0, IBM, USA) was used for data analysis, and RStudio (PBC & Certified B Corp.^®^, USA) was used to generate graphs.

## 3 Results

### 3.1 Categorization changes from the WHO CNS4 to the WHO CNS5 classification

**Figure 1:**
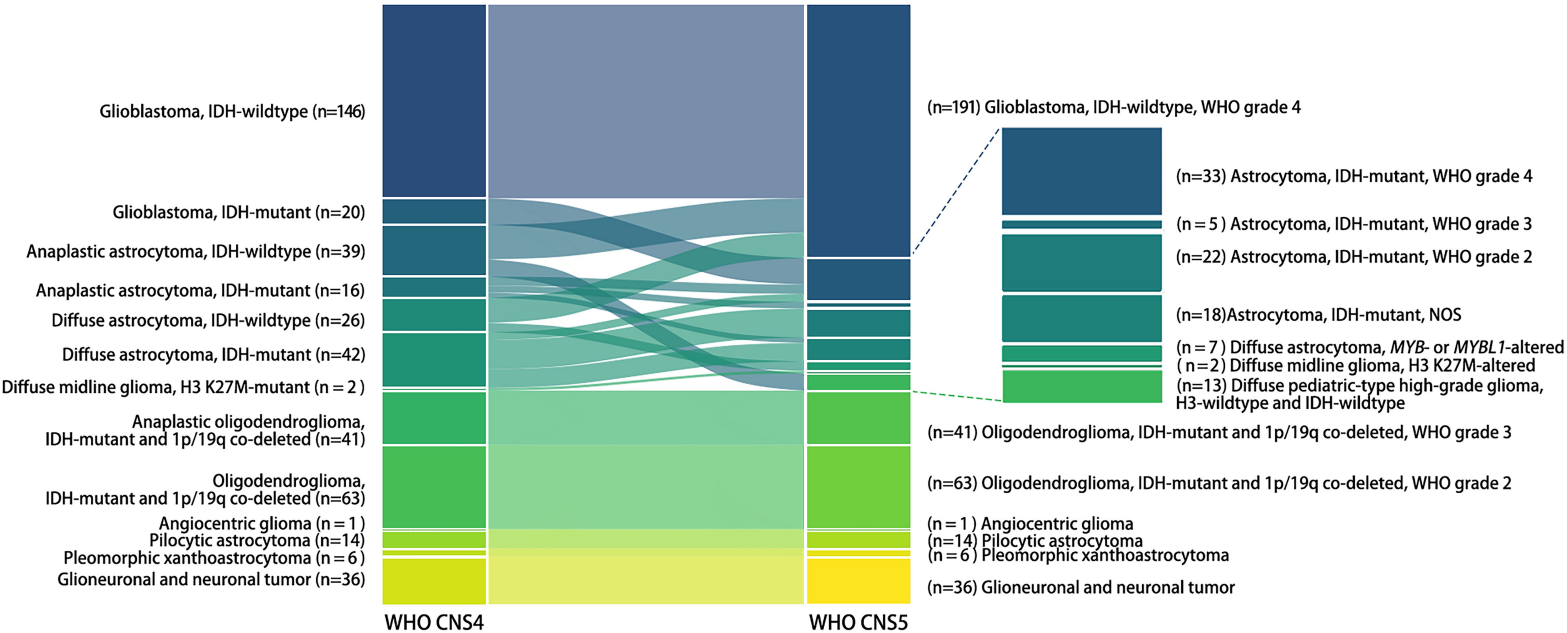
Categorization alterations of human gliomas from the 4^th^ to the 5^th^ edition of the WHO classification of CNS tumors. Each bar in the Sankey diagram represents a certain subtype of gliomas. The bars on the left represent the prior classification of gliomas (WHO CNS4), while those on the right represent the current classification (WHO CNS5). The name of each subtype and the number of tumors is marked to the lateral side of the bar.

The classification of adult- and pediatric-type gliomas changed greatly, while glioneuronal and neuronal tumors and circumscribed astrocytic glioma remained unchanged (**Figure 1**). In this analysis, glioblastoma defined by the current classification consisted of three entities defined by the WHO CNS4 classification: glioblastoma, IDH-wildtype (146/191), anaplastic astrocytoma, IDH-wildtype (26/191), and diffuse astrocytoma, IDH-wildtype (19/191). The remaining IDH-wildtype anaplastic astrocytomas (13/39) and IDH-wildtype diffuse astrocytomas (7/26) were reclassified as pediatric-type diffuse astrocytoma, *MYB-* or *MYBL1*-altered or high-grade diffuse glioma, H3- and IDH-wildtype. Astrocytoma was subdivided into grades 2–4 or not otherwise specified (NOS), consisting of diffuse astrocytoma, IDH-mutant (6 in grade 4, 22 in grade 2, and 14 in NOS), anaplastic astrocytoma, IDH-mutant (7 in grade 4, 5 in grade 3, and 4 in NOS), and glioblastoma, IDH-mutant (20 in grade 4) defined according to the previous classification. The nomenclature of anaplastic oligodendroglioma and diffuse midline glioma, H3 K27M-mutant was altered while the number of tumors remained unchanged.

### 3.2 Clinical, radiological, and pathological features of gliomas using the current classification

Adult-type diffuse gliomas, including glioblastoma (n=191), oligodendroglioma (n=104), and astrocytoma (n=78), were dominant subtypes among 452 gliomas, followed by glioneuronal and neuronal tumors (n=36), pediatric-type diffuse gliomas (n=23), and circumscribed astrocytic gliomas (n=20). The mean age of patients was 56 years for glioblastoma, 44 years for oligodendroglioma, 41 years for astrocytoma, and 25 years for circumscribed astrocytic glioma. Male patients accounted for the majority of pediatric-type diffuse gliomas and other subtypes except for circumscribed astrocytic gliomas. Epilepsy was most common in glioneuronal and neuronal tumors, intracranial hypertension was most common in glioblastomas, and neurologic impairments were most common in glioblastomas and circumscribed astrocytic gliomas. The disease duration was shortest in glioblastomas and longest in glioneuronal and neuronal tumors. Glioblastoma had the largest maximal tumor diameter of 4.3cm, while circumscribed astrocytic glioma had the smallest diameter of 2.3 cm. The contrast enhancement of tumors and necrosis was common in glioblastoma. Other clinical, radiological, and pathological features of different subtypes of gliomas were summarized in **Table 1**.

**Table 1:** Clinical characteristics of gliomas and subtypes classified by the 5^th^ edition of the WHO classification of CNS tumors.

	Astrocytoma (n=78)	Oligodendroglioma (n=104)	Glioblastoma (n=191)	Circumscribed astrocytic glioma (n=20)	Pediatric-type diffuse glioma (n=23)	Glioneuronal and neuronal tumor (n=36)
Age at diagnosis, year	41.2 ± 11.1	44.1 ± 11.4	55.5 ± 14.7 ↑	24.6 ± 13.3 ↓	47.3 ± 14.8	30.1 ± 16.7
Age at diagnosis ≥ 60, n/%	4/5.1%	13/12.5%	80/41.9% ↑	0/0% ↓	4/17.4%	4/11.1%
Male, n/%	54/69.2%	59/56.7%	100/52.4%	10/50.0% ↓	17/73.9% ↑	20/55.6%
BMI, kg/m^2^	24.3 ± 3.1	24.6 ± 3.7 ↑	23.8 ± 3.3	21.0 ± 5.2 ↓	23.4 ± 2.9	23.1 ± 4.4
Clinical symtoms						
Intracranial hypertension, n/%	37/47.4%	37/35.6%	92/48.2% ↑	6/30.0%	8/34.8%	8/22.2% ↓
Epilepsy, n/%	31/39.7%	44/42.3%	40/20.9%	6/30.0%	3/13.0% ↓	22/61.1% ↑
Neurologic impairment, n/%	40/51.3%	46/44.2%	129/67.5%	14/70.0% ↑	14/60.9%	13/36.1% ↓
Motor dysfunction, n/%	18/23.1%	18/17.3%	71/37.2%	8/40.0% ↑	9/39.1%	6/16.7% ↓
Aphasia, n/%	5/6.4%	6/5.8%	40/20.9% ↑	0/0% ↓	2/8.7%	2/5.6%
Sensory dysfunction, n/%	4/5.1%	7/6.7%	16/8.4%	6/30.0% ↑	6/26.1%	0/0% ↓
Visual field defect, n/%	4/5.1%	9/8.7%	16/8.4%	2/10.0% ↑	1/4.3% ↓	2/5.6%
Psychological changes or memory loss, n/%	5/6.4% ↑	5/4.8%	10/5.2%	0/0% ↓	0/0% ↓	1/2.8%
Disease duration before admission, week	8 (3, 27)	12 (4, 98)	5 (2, 13) ↓	21 (12, 120)	12 (3, 24)	46 (12, 275) ↑
Baseline KPS score, n	90 (80, 100)	95 (80, 100) ↑	80 (80, 100)	75 (70, 80) ↓	90 (80, 100)	80 (80, 90)
Radiological characteristics on MRI^a^						
Tumor maximal diameter, cm	4.1 (3.3, 5.8)	4.2 (3.4, 5.7)	4.3 (3.0, 5.4) ↑	2.3 (1.8, 4.3) ↓	3.3 (2.5, 4.9)	3.3 (1.9, 5.7)
Tumor location (if involved)						
Frontal lobe, n/%	55/65, 84.6% ↑	69/90, 76.7%	77/167, 46.1%	0/6, 0% ↓	6/14, 42.9%	6/17, 35.3%
Temporal lobe, n/%	17/65, 26.2% ↓	26/90, 28.9%	69/167, 41.3%	3/6, 50.0% ↑	5/14, 35.7%	8/17, 47.1%
Parietal lobe, n/%	12/65, 18.5%	19/90, 21.1%	55/167, 32.9% ↑	0/6, 0% ↓	4/14, 28.6%	2/17, 11.8%
Occipital lobe, n/%	2/65, 3.1% ↓	4/90, 4.4%	31/167, 18.6%	2/6, 33.3% ↑	2/14, 14.3%	2/17, 11.8%
Subtentorial structures, n/%	1/65, 1.5%	0/90, 0% ↓	2/167, 1.2%	1/6, 16.7% ↑	2/14, 14.3%	0/17, 0% ↓
Multiple tumors, n/%	5/65, 7.7%	4/90, 4.4%	31/167, 18.6% ↑	0/6, 0% ↓	2/14, 14.3%	0/17, 0% ↓
Functional area involvement, n/%	22/65, 33.8%	19/90, 21.1%	92/167, 55.1% ↑	1/6, 16.7% ↓	5/14, 35.7%	3/17, 17.6%
Hypointensive signal on T1WI, n/%	43/65, 66.2%	71/90, 78.9%	94/167, 56.3% ↓	5/6, 83.3% ↑	11/14, 78.6%	14/17, 82.4%
Hyperintensive signal on T2WI, n/%	37/65, 56.9%	56/90, 62.2%	84/167, 50.3% ↓	5/6, 83.3% ↑	11/14, 78.6%	11/17, 64.7%
Contrast enhancement, n/%	28/65, 43.1%	31/90, 34.4%	147/167, 88.0% ↑	5/6, 83.3%	10/14, 71.4%	12/17, 70.6%
Intratumoral necrosis, n/%	25/65, 38.5%	31/90, 34.4%	130/167, 77.8% ↑	2/6, 33.3%	7/14, 50.0%	8/17, 47.1%
Extent of surgical resection						
Gross total resection, n/%	47/60.3% ↓	77/74.0%	118/61.8%	15/75.0%	14/60.9%	32/88.9% ↑
Subtotal resection, n/%	20/25.6% ↑	15/14.4%	34/17.8%	3/15.0%	3/13.0%	3/8.3% ↓
Biopsy, n/%	11/14.1%	12/11.5%	39/20.4%	2/10.0%	6/26.1% ↑	1/2.8% ↓
Histological grade						
WHO grade 4, n/%	21/26.9%	0/0%	146/76.4% ↑	0/0% ↓	3/13.0%	0/0% ↓
WHO grade 3, n/%	16/20.5%	41/39.4%	27/14.1%	3/15.0%	11/47.8% ↑	4/11.1% ↓
WHO grade 2, n/%	41/52.6%	63/60.6% ↑	18/9.4%	2/10.0%	9/39.1%	3/8.3% ↓
WHO grade 1, n/%	0/0%	0/0%	0/0%	15/75.0%	0/0% ↓	29/80.6% ↑
Ki-67 index	8 (3, 25)	5 (3, 10)	30 (15, 50) ↑	2 (1, 5)	10 (4, 30)	1.5 (1, 3) ↓

KPS, karnofsky performance scale; MRI, magnetic resonance imaging; T1WI, T1-weighed image; T2WI, T2-weighed image.

a. In this section, only the patients with both the preoperative and postoperative DICOM files of MRIs were included for analysis.

b. ↑ indicated the highest values among these subtypes of gliomas, while ↓ indicated the lowest.

### 3.3 Overall survival of different subtypes of gliomas using the current classification system

**Figure 2:**
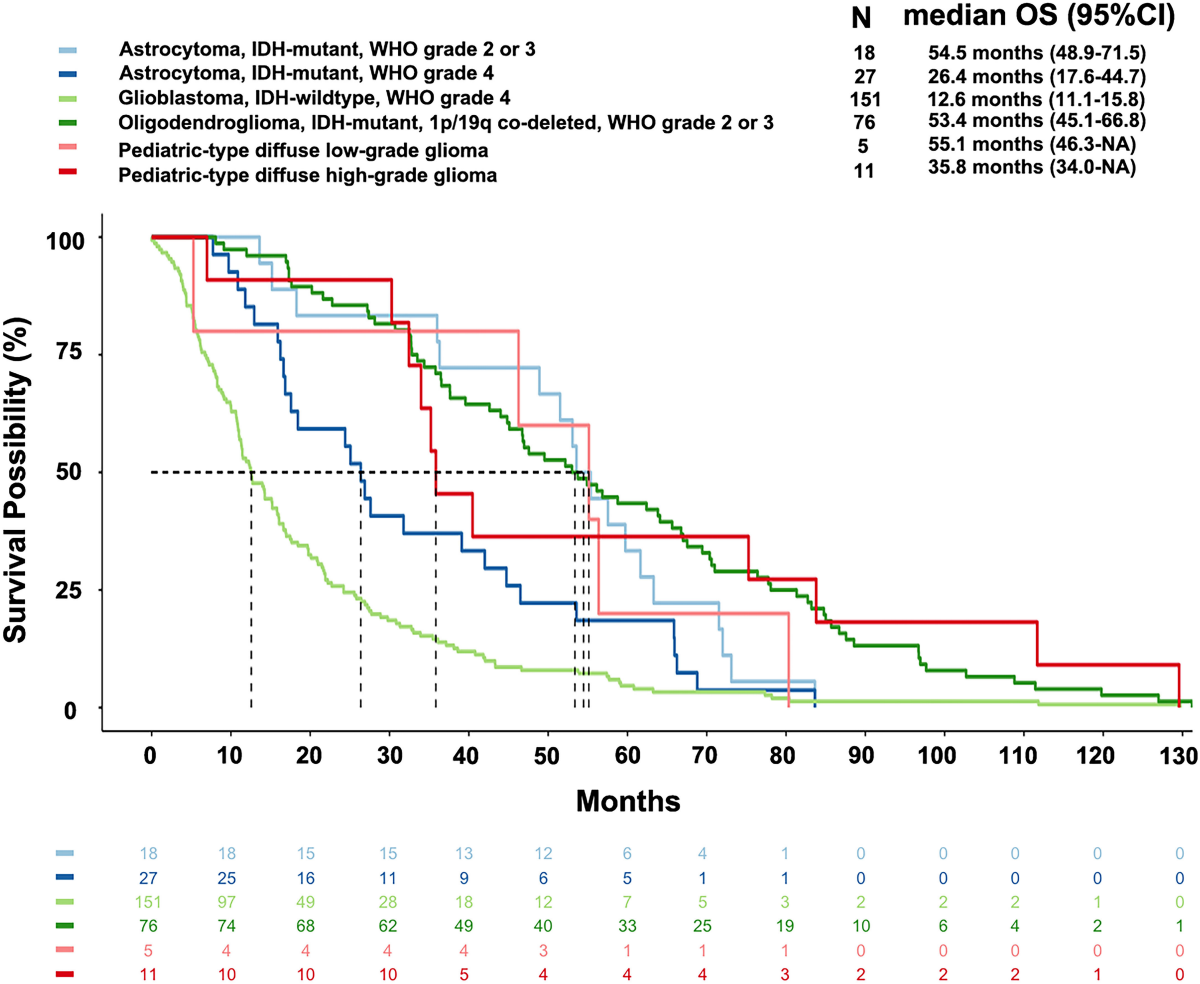
Overall survival of main subtypes of gliomas according to the current WHO classification. The median overall survival of glioblastoma, astrocytoma (WHO grade 4), pediatric-type high-grade diffuse glioma, oligodendroglioma (WHO grade 2-3), astrocytoma (WHO grade 2-3), and pediatric-type low-grade diffuse glioma were 12.6 months, 26.4 months, 35.8 months, 53.4 months, 54.5 months, and 55.1 months, respectively.

Glioblastoma, IDH-wildtype, WHO grade 4, had the shortest mOS of 12.6 months among all subtypes. Astrocytoma, IDH-mutant had different mOS according to the WHO grade: grade 4 (26.4 months), grade 3 (53.6 months), and grade 2 (55.4 months). The latter two were similar to oligodendroglioma, IDH-mutant and 1p/19q co-deleted, WHO grade 3 (45.8 months) and grade 2 (56.5 months). The pediatric-type high-grade diffuse gliomas had an mOS of 35.8 months, while the low-grade gliomas had an mOS of 55.1 months (**Figure 2**).

**Figure 3:**
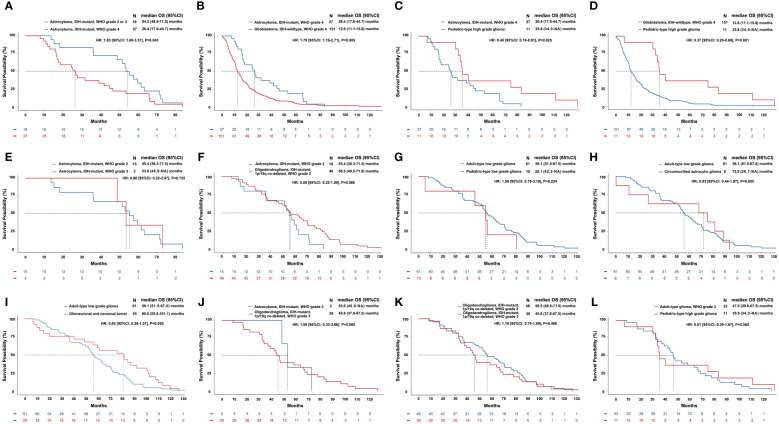
Comparisons of overall survival of different subtypes of gliomas classified by the current WHO classification of CNS tumors. The horizontal axis of each panel represents the survival time (months), while the vertical axis represents the survival probability (%). Kaplan–Meier curves were drawn, and median OS and 95% CI were calculated. We compared the differences of OS between patients with the same subtype of gliomas but different WHO grades **(A, E, K)** and between patients with different subtypes of gliomas but similar WHO grades **(B-D, F-J, L)**. A: astrocytoma, IDH-mutant with a relatively low grade (WHO grade 2-3) had a longer OS than astrocytoma, IDH-mutant, WHO grade 4 (mOS: 54.5 vs. 26.4, months, *P*=0.043). **(B)** Astrocytoma, IDH-mutant, WHO grade 4 had a longer OS than glioblastoma, IDH-wildtype, WHO grade 4 (mOS: 26.4 vs. 12.6, months, *P*=0.005). **(C)** Pediatric-type high-grade glioma had a longer OS than astrocytoma, IDH-mutant, WHO grade 4 (mOS: 35.8 vs. 26.4, months, *P*=0.025). **(D)** Pediatric-type high-grade glioma had a longer OS than glioblastoma, IDH-wildtype, WHO grade 4 (mOS: 35.8 vs. 12.6, months, *P*=0.001). **(E-L)** Differences of OS in other comparisons were not significant.

The mOS of astrocytoma, IDH-mutant, WHO grade 4 was shorter than that of astrocytoma with a lower WHO grade of 2-3 [hazard ratio (HR)=1.83, *P*=0.043]. The mOS of glioblastoma, IDH-wildtype, WHO grade 4 was shorter than that of astrocytoma, IDH-mutant, WHO grade 4 (HR=1.79, *P*=0.005). Pediatric-type high-grade glioma had an mOS of 35.8 months, which was significantly longer than both the glioblastoma (HR=0.40, *P*=0.025) and astrocytoma, WHO grade 4 (HR=0.37, *P*=0.001). The above results and other survival comparisons among adult-type diffuse gliomas and between different subtypes of gliomas were illustrated in **Figure 3**.

### 3.4 Molecular landscape as classified by the WHO CNS5 classification

**Figure 4:**
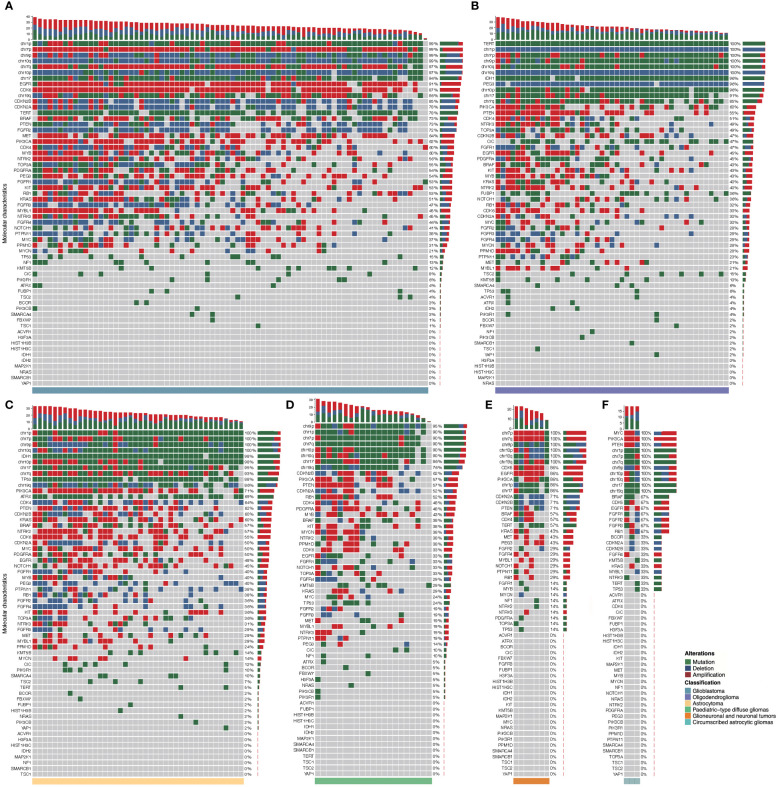
Molecular landscape of six major subtypes of gliomas classified by the current WHO classification. Each column represents an individual patient, and the subtype of glioma is displayed at the bottom of each waterfall heatmap. Each row indicates a molecular parameter, and these parameters are listed from top to bottom by the frequency of genetic alterations. Mutation is shown as green, deletion is shown as blue, and amplification is shown as red. The frequencies of mutation, deletion, and amplification of each gene are listed to the right of the histogram. **(A)**: Glioblastoma, IDH-wildtype; **(B)**: Oligodendroglioma, IDH-mutant and 1p/19q co-deleted; **(C)**: Astrocytoma, IDH-mutant; **(D)**: Pediatric-type diffuse gliomas; **(E)** Glioneuronal and neuronal tumors; **(F)**: Circumscribed astrocytic gliomas.

Each subtype of gliomas had distinct patterns of molecular alterations in chromosomes and genes (mutation, amplification, or deletion). A detailed molecular landscape of each subtype was shown in **Figure 4**. The exact numbers and percentages of alterations of specific chromosomes and genes of each subtype of gliomas as classified by the WHO CNS5 were shown in **Supplementary Table 1**.

### 3.5 Implications of molecular alterations with patient survival

**Figure 5:**
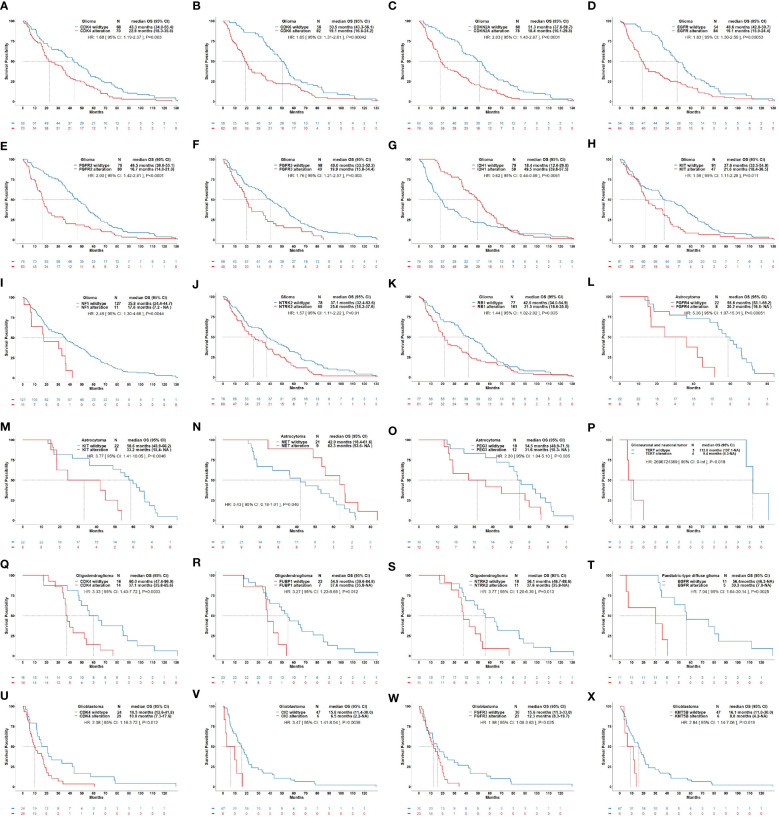
Correlations of molecular alteration with overall survival of patients with gliomas. This figure illustrates significant correlations of molecular alterations on the OS of glioma as a whole **(A–K)** and its subtypes including astrocytoma **(L–O)**, glioneuronal and neuronal tumor **(P)**, oligodendroglioma **(Q–S)**, pediatric-type diffuse glioma **(T)**, and glioblastoma **(U–X)**. **(A)**: OS curves for *CDK4* alteration vs. *CDK4* wildtype in glioma (HR: 1.68, *P*=0.003). **(B)**: OS curves for *CDK6* alteration vs. *CDK6* wildtype in glioma (HR: 1.85, *P*<0.001). **(C)**: OS curves for *CDKN2A* alteration vs. *CDKN2A* wildtype in glioma (HR: 2.03, *P*<0.001). **(D)**: OS curves for *EGFR* alteration vs. *EGFR* wildtype in glioma (HR: 1.83, *P*<0.001). **(E)**: OS curves for *FGFR2* alteration vs. *FGFR2* wildtype in glioma (HR: 2.00, *P*<0.001). **(F)**: OS curves for *FGFR3* alteration vs. *FGFR3* wildtype in glioma (HR: 1.76, *P*=0.003). **(G)**: OS curves for *IDH1* alteration vs. *IDH1* wildtype in glioma (HR: 0.62, *P*=0.006). **(H)**: OS curves for *KIT* alteration vs. *KIT* wildtype in glioma (HR: 1.59, *P*=0.011). **(I)**: OS curves for *NF1* alteration vs. *NF1* wildtype in glioma (HR: 2.46, *P*=0.004). **(J)**: OS curves for *NTRK2* alteration vs. *NTRK2* wildtype in glioma (HR: 1.57, *P*=0.01). **(K)**: OS curves for *RB1* alteration vs. *RB1* wildtype in astrocytoma (HR: 1.44, *P*=0.035). **(L)**: OS curves for *FGFR4* alteration vs. *FGFR4* wildtype in astrocytoma (HR: 5.36, *P*<0.001). **(M)**: OS curves for *KIT* alteration vs. *KIT* wildtype in astrocytoma (HR: 3.77, *P*=0.005). **(N)**: OS curves for *MET* alteration vs. *MET* wildtype in astrocytoma (HR: 0.43, *P*=0.046). **(O)**: OS curves for *PEG3* alteration vs. *PEG3* wildtype in astrocytoma (HR: 2.30, *P*=0.035). **(P)**: OS curves for *TERT* alteration vs. *TERT* wildtype in glioneuronal and neuronal tumor (HR: 2696724389, *P*=0.018). **(Q)**: OS curves for *CDK4* alteration vs. *CDK4* wildtype in oligodendroglioma (HR: 3.33, *P*=0.003). **(R)**: OS curves for *FUBP1* alteration vs. *FUBP1* wildtype in oligodendroglioma (HR: 3.27, *P*=0.012). **(S)**: OS curves for *NTRK2* alteration vs. *NTRK2* wildtype in oligodendroglioma (HR: 3.77, *P*=0.013). **(T)**: OS curves for *EGFR* alteration vs. *EGFR* wildtype in pediatric-type diffuse glioma (HR: 7.04, *P*=0.003). **(U)**: OS curves for *CDK4* alteration vs. *CDK4* wildtype in glioblastoma (HR: 2.08, *P*=0.012). **(V)**: OS curves for *CIC* alteration vs. *CIC* wildtype in glioblastoma (HR: 3.47, *P*=0.004). **(W)**: OS curves for *FGFR3* alteration vs. *FGFR3* wildtype in glioblastoma (HR: 1.98, *P*=0.025). **(X)**: OS curves for *KMT5B* alteration vs. *KMT5B* wildtype in glioblastoma (HR: 2.84, *P*=0.019).

In addition to *IDH1/2* mutation, *MGMT* promotor methylation, *EGFR* amplification, *TERT* promotor mutation, and *CDKN2A/B* homozygous deletion, we sought to elucidate the alterations of other potential molecular biomarkers that might provide clues for clinical decision-making in gliomas. The current results showed that alterations in *CDK4*, *CDK6*, *CDKN2A*, *EGFR*, *FGFR2*, *FGFR3*, *KIT*, *NF1*, *NTRK2*, and *RB1* were correlated with a short OS in gliomas (**Figure 5**). The alterations in *FGFR4*, *KIT*, and *PEG3* were correlated with a short OS in astrocytoma, alterations in *CDK4*, *FUBP1*, and *NTRK2* were correlated with a short OS in oligodendroglioma, and alterations in *CDK4*, *CIC*, *FGFR3*, and *KMT5B* were correlated with a short OS in glioblastoma. In pediatric-type diffuse gliomas and glioneuronal and neuronal tumors, alterations in *EGFR* and *TERT* were correlated with a poor prognosis, respectively. The correlations between other molecular changes and the survival of patients with gliomas were not significant and summarized in **Supplementary Figures 1**-**6**.

## 4 Discussion

The 2021 WHO CNS5 classification provides a comprehensive reclassifications and redefinitions of malignant gliomas. In this real-world study, we reported the current categorization of gliomas and observed that their incidence and composition changed from the previous classification. The clinical characteristics of each subtype, including demographic information, clinical symptoms, and radiological features, were summarized. All the patients included in the research were classified according to the criteria of both 2016 and 2021 WHO classification of Tumors of the Central Nervous System respectively under the guidance of experienced neuropathologists, including 72 patients who underwent surgery between 2021-2022. We informed the surviving patients by telephone follow-up and recommended potential therapies according to the new molecular classification guideline. The survival outcomes of each subgroup were analyzed and compared. Grade-4 gliomas, including glioblastoma and astrocytoma, had significantly worse survival than grade-2/3 oligodendroglioma and astrocytoma. The mOS of glioblastoma, grade-4 astrocytoma, and pediatric-type high-grade glioma were among the lowest and differed from each other. Additional molecular markers, except for *IDH*, *MGMT* promotor, and chromosome 1p/19q, were identified with remarkable prognostic implications. Overall, we conducted a comprehensive analysis of gliomas based on the current WHO classification, updated our knowledge, and provided guidance for the diagnosis and potential treatment for gliomas.

Adding specific molecular markers into the process of identifying a specific subtype of glioma and re-organizing the subgroups of adult- and pediatric-type gliomas were the two major strikes of the updated classification. In this study, we aimed to examine the subtype shifts and the corresponding changes in the clinical information that could assist clinicians in the preliminary diagnosis. IDH-wildtype glioblastoma comprised a quarter of newly-defined molecular subtype, slightly lower than the previous report of 39.16% (18). About 39% of IDH-mutant, grade-4 astrocytomas were grade 2/3 astrocytomas with CDKN2A/B homozygous deletion. One-third of IDH-wildtype astrocytomas in adults transformed into pediatric-type gliomas, while the remaining were re-classified as molecular glioblastomas. Regarding clinical characteristics, the elderly tended to suffer aggressive tumor types, as expected (1). Epilepsy is most prevalent in glioneuronal tumor (14) and also common in IDH-mutant glioma (19). Notably, neurological deficits had a bipolar distribution, which was common in the most aggressive glioblastoma and least aggressive circumscribed glioma.

With the addition of specific molecular markers, the WHO CNS5 classification can stratify the prognosis of glioma quite efficiently. Recent studies have reported the survival outcomes according to the latest classification. However, the majority of these studies have only focused on some subtypes, primarily the most malignant form, glioblastoma, IDH-wildtype. The analysis based on national data of gliomas from the USA showed that glioblastoma, IDH-wildtype had a 1-year survival rate of 53.7%, and astrocytoma, IDH-mutant, WHO grade 4 of 73.6% (14). Another multicenter study reported that histological glioblastoma and molecular glioblastoma had 26 months and 21 months of OS, respectively (18). The current study indicated an OS of 12.6 months for glioblastoma, IDH-wildtype, 26.4 months for astrocytoma, IDH-mutant, WHO grade 4, and 35.8 months for pediatric-type high-grade glioma. Similar to previous findings, the survival for glioblastoma was significantly worse than for astrocytoma, WHO grade 4, which is supporting evidence to distinguish IDH-mutant astrocytoma from IDH-wildtype glioblastoma (17). These outcomes validated the distinguishing value of WHO CNS5 classification for high-grade gliomas, based on the combination of histology and molecular patterns.

Next, we analyzed the outcomes of gliomas with a relatively low grade. The survival of grade 2-3 oligodendroglioma, grade 2-3 astrocytoma, and pediatric-type low-grade glioma was better than that of glioblastomas, grade-4 astrocytoma, and pediatric-type high-grade gliomas, but no significant difference was found, ranging from 53.6 months to 55.4 months. Whether the histological grading can distinguish the survival of grade-2 and -3 astrocytoma is controversial (20). One study has yielded that grade-2 astrocytoma has better survival than grade-3 astrocytoma (14). Phase III EORTC 26053-22054 trial emphasized the differential grading of astrocytoma and suggested that adjuvant temozolomide is exclusively beneficial only for grade-3 astrocytoma (3). These findings focused on the investigation of survival and accurate classification of relatively low grade IDH-mutant gliomas and indicated the significance of biomarkers in the stratification of the prognosis.

Since the publication of the WHO CNS5 classification, the importance of molecular markers in the diagnosis, prognosis, and treatment planning has been emphasized: *TERT* promoter mutation, *EGFR* amplification, and chromosome +7/-10 copy number variation for IDH-wildtype glioma; *CDKN2A/B* homozygous deletion for IDH-mutant astrocytoma; *H3K27* and *H3G34* alteration for pediatric-type glioma (8); *MGMT* promoter methylation for the treatment response to temozolomide (21). In this study, we sought to identify the additional potential molecular markers. *CDK4*, which is involved in the retinoblastoma signaling pathway, is associated with dismal survival in oligodendroglioma, astrocytoma, and glioblastoma (22). Some studies have shown that the *CDK4/6* inhibitor can overcome temozolomide resistance and reduce the number of inhibitory M2-macrophages in glioblastoma (23), although the phase II clinical trial for recurrent glioblastoma patients has failed (24). A recent study showed that the combined treatment with *CDK4/6* inhibitor and andoncolytic virus-induced immunogenic cell death, enhanced antitumor immunity, inhibited tumor growth, and prolonged the survival of tumor-bearing mice (25). Another promising therapy is the combination of *CDK4/6* and *PI3K/mTOR* inhibitors for the treatment of recurrent glioblastoma (26). The *FGFR* family might also be a promising biomarker available for astrocytoma and glioblastoma. Previous studies have shown that *FGFR* fusion and overexpression are associated with poor prognosis in gliomas (27–29), especially glioblastomas (30). FGFR inhibitors showed promising efficacy in recurrent gliomas harboring *FGFR1* or *FGFR3* point mutations or *FGFR3*-*TACC3* fusions (31). *CIC* and *FUBP1*, transcription factors that counteract the RTK/Ras/ERK signaling pathway, have been reported in oligodendroglioma (32–34). Although *CIC* had minimal expression in glioblastoma due to continuous E3 ligation (35), the genetic alteration correlated with short OS was meaningful in glioblastoma. Another molecular marker identified for glioblastoma was *KMT5B*. It serves as a risk gene for autism spectrum disorder and has only been reported in pediatric glioma and diffuse intrinsic pontine glioma. Our analysis revealed that *KMT5B* was associated with significantly shorter survival in glioblastoma, which requires further research and validation.

### 4.1 Limitations

Nevertheless, the present study has some limitations. Firstly, because our center mainly treated adult patients, 22/23 patients with pediatric-type gliomas based on the current classification were adults, raising the concern that the epidemiology and clinical data for this subtype of glioma might be biased. Also, the small number of pediatric-type diffuse low-grade glioma subgroup and pediatric-type diffuse high-grade glioma may introduce potential bias. However, these results provided valuable evidence for pediatric-type gliomas in adults, which were not deduced before the release of the updated classification. Secondly, because of loss or deterioration of some of the paraffin-embedded tissue samples during these years, only 452/605 (75%) patients treated at our center were enrolled in the analysis, further increased the selection bias. Thirdly, we pre-designed a panel of 60 molecular markers of interest for analyzing the molecular alterations. Although these markers were sufficient for categorization of gliomas according to the current classification scheme, there might be molecules not involved but affect the prognosis. Whole-exome sequencing could be an alternative tool to address this issue.

## 5 Conclusions

In this real-world study of 452 glioma patients during a follow-up of 11 years, we illustrated the comprehensive classification of gliomas according to the WHO CNS5 classification and presented the clinical, radiological, molecular, and survival features of each subtype. Clinicians are encouraged to acknowledge these diagnostic and therapeutic advances in this lethal brain malignancy since many clinical characteristics of glioma and its subtypes have changed significantly from the previous to the current classification. Additional biomarkers that might have prognostic potential have been identified, highlighting the value of an integrated histological and molecular classification scheme. The present study provided clinical implications of the revision of the WHO classification of gliomas that would guide future healthcare practice and the investigations in the diagnosis, treatment, prognosis, and molecular classification of gliomas.

## Data availability statement

The original contributions presented in the study are included in the article/**Supplementary Material**. Further inquiries can be directed to the corresponding authors.

## Author contributions

Substantial contributions to conception and design of this study: all authors; Substantial contributions to acquisition of data: all authors; Substantial contributions to analysis and interpretation of data: XG, YS, DL, YL, and WC; Drafting the original article: XG, YS, DL, YL, and WC; Revising the article critically for important intellectual content: YNW, YKW, HX, YX, JL, JW, TL, HW, QL, SJ, TQ, SG, HL, TY, KZ, YW, and WM. All authors contributed to the article and approved the submitted version.
