# Supplementary material for: Clinical updates on gliomas and implications of the 5th edition of the WHO classification of central nervous system tumors
Source: Front Oncol. 2023 Mar 14;13:1131642. doi: 10.3389/fonc.2023.1131642 (PMC10043404; doi:10.3389/fonc.2023.1131642)
Supplement: Supplementary file 1 [file DataSheet_1.pdf]

Supplement Figure 1. Kaplan–Meier curves showing no significant effects of certain genetic alterations on the overall survival of patients with glioma (1-36). Since the numbers of patients divided into two groups (e.g. BRAF wildtype/BRAF alteration) using different parameters varied, we only enrolled the parameters using which the number of patients in either group was above 3. Parameters used in this section were shown as follows.

(1) ATRX 1
(2) BCOR 2
(3) BRAF 3
(4) CDKN2B 4
(5) CIC 5
(6) FBXW7 6
(7) FGFR1 7
(8) FGFR4 8
(9) FUBP1 9
(10) KRAS 10
(11) MET 11
(12) MYB 12
(13) MYBL1 13
(14) MYC 14
(15) MYCN 15
(16) NOTCH1 16
(17) NTRK3 17
(18) PDGFRA 18
(19) PEG3 19
(20) PIK3CA 20
(21) PIK3CB 21
(22) PIK3R1 22
(23) PPM1D 23
(24) PTEN 24
(25) PTPN11 25
(26) SMARCA4 26
(27) TERT 27
(28) TOP3A 28
(29) TP53 29
(30) TSC2 30
(31) chr1p 31
(32) chr7p 32
(33) chr7q 33
(34) chr10p 34
(35) chr17 35
(36) chr19q 36

(1)

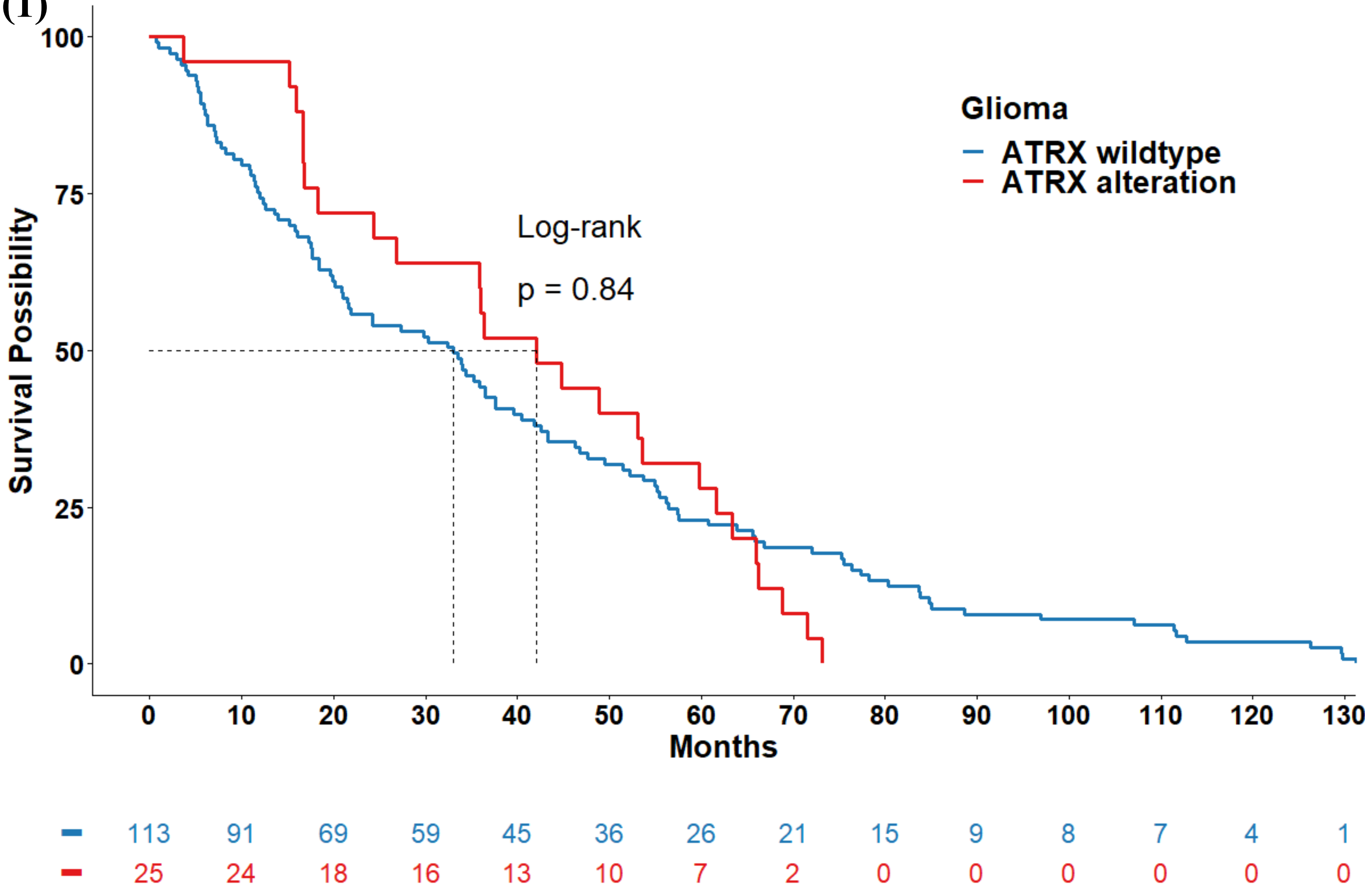

Glioma
ATRX wildtype
ATRX alteration
Log-rank
p = 0.84
Survival Possibility
100
75
50
25
0
0
10
20
30
40
50
60
70
80
90
100
110
120
130
Months
113 91 69 59 45 36 26 21 15 9 8 7 4 1
25 24 18 16 13 10 7 2 0 0 0 0 0 0

(2)

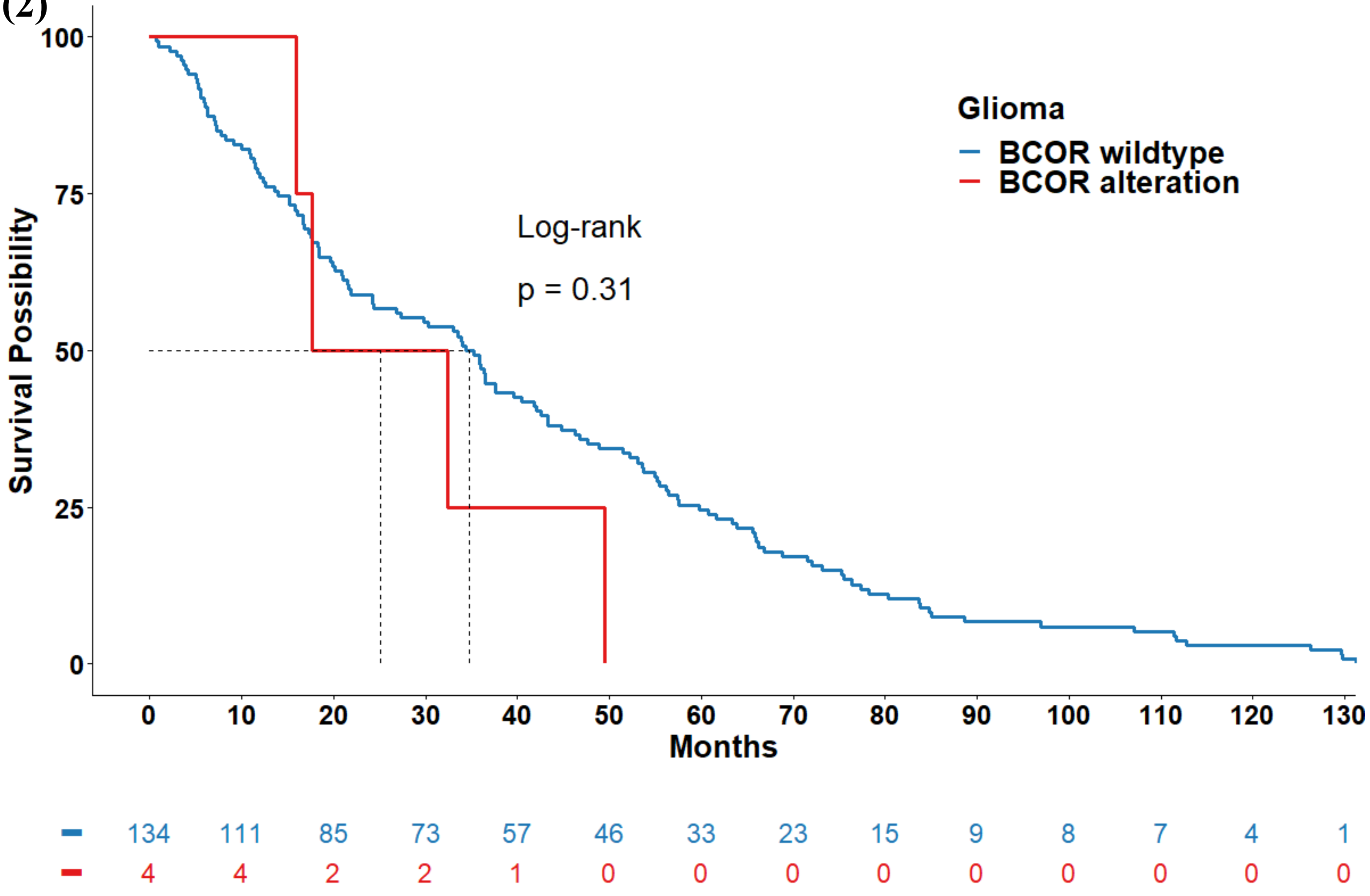

Glioma
BCOR wildtype
BCOR alteration
Log-rank
p = 0.31
Survival Possibility
100
75
50
25
0
0 10 20 30 40 50 60 70 80 90 100 110 120 130
Months
134 111 85 73 57 46 33 23 15 9 8 7 4 1
4 4 2 2 1 0 0 0 0 0 0 0 0 0

(3)

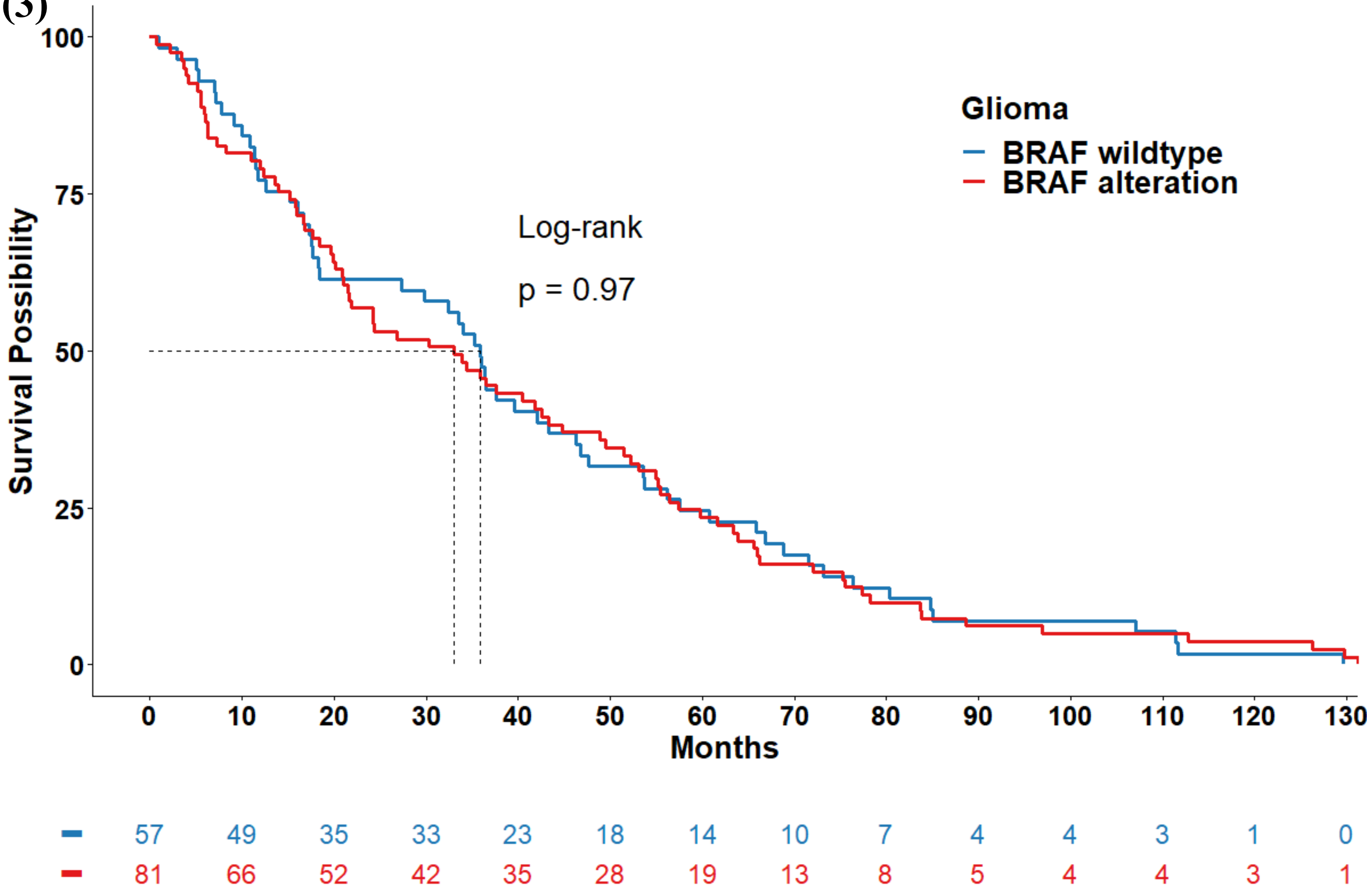

Survival Possibility
100
75
50
25
0
Log-rank
p = 0.97
Glioma
BRAF wildtype
BRAF alteration
0
10
20
30
40
50
60
70
80
90
100
110
120
130
Months
57 49 35 33 23 18 14 10 7 4 4 3 1 0
81 66 52 42 35 28 19 13 8 5 4 4 3 1

(4)

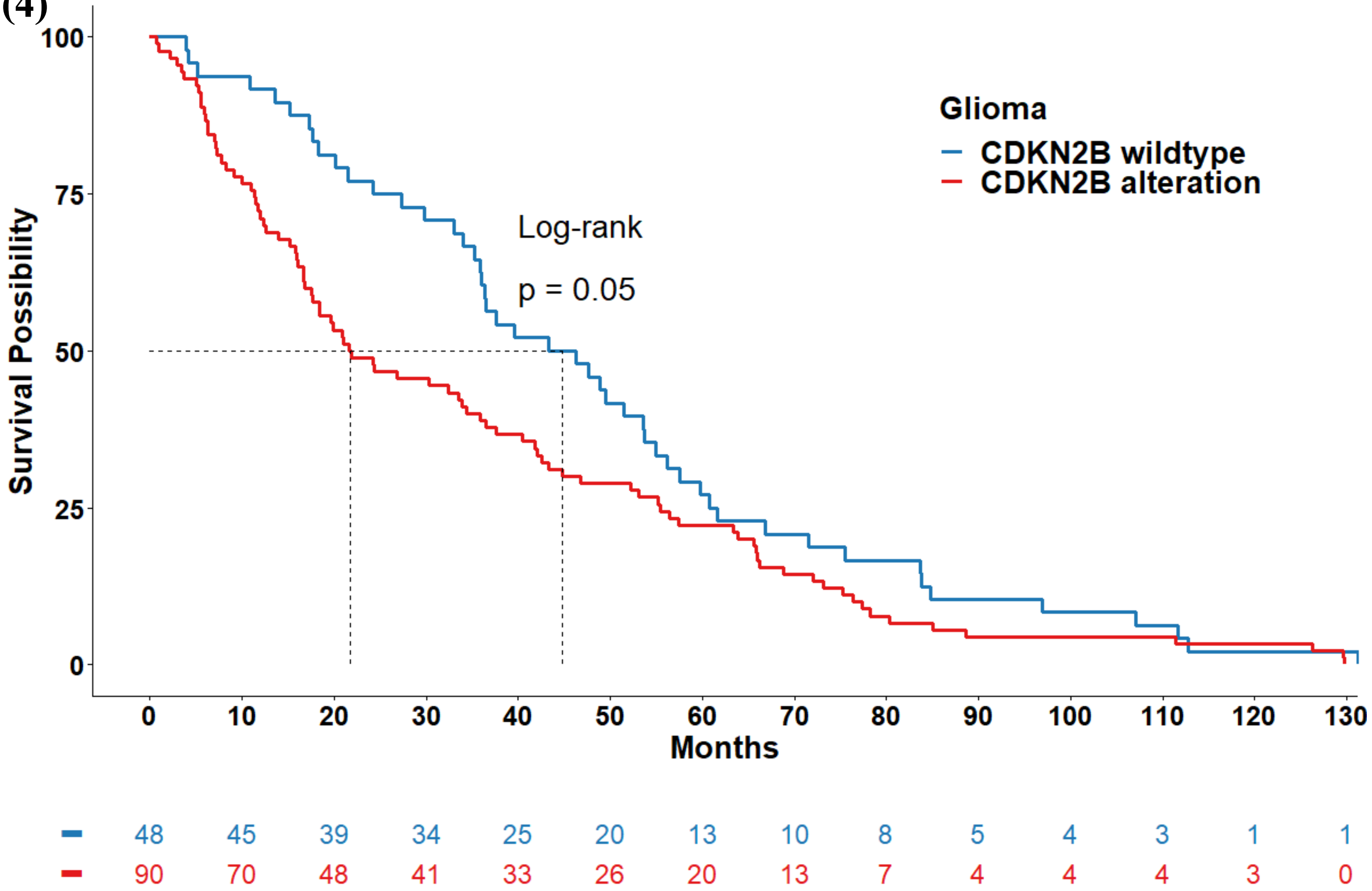

Survival Possibility
100
75
50
25
0
Log-rank
p = 0.05
Glioma
CDKN2B wildtype
CDKN2B alteration
0
10
20
30
40
50
60
70
80
90
100
110
120
130
Months
48 45 39 34 25 20 13 10 8 5 4 3 1 1
90 70 48 41 33 26 20 13 7 4 4 4 3 0

(5)

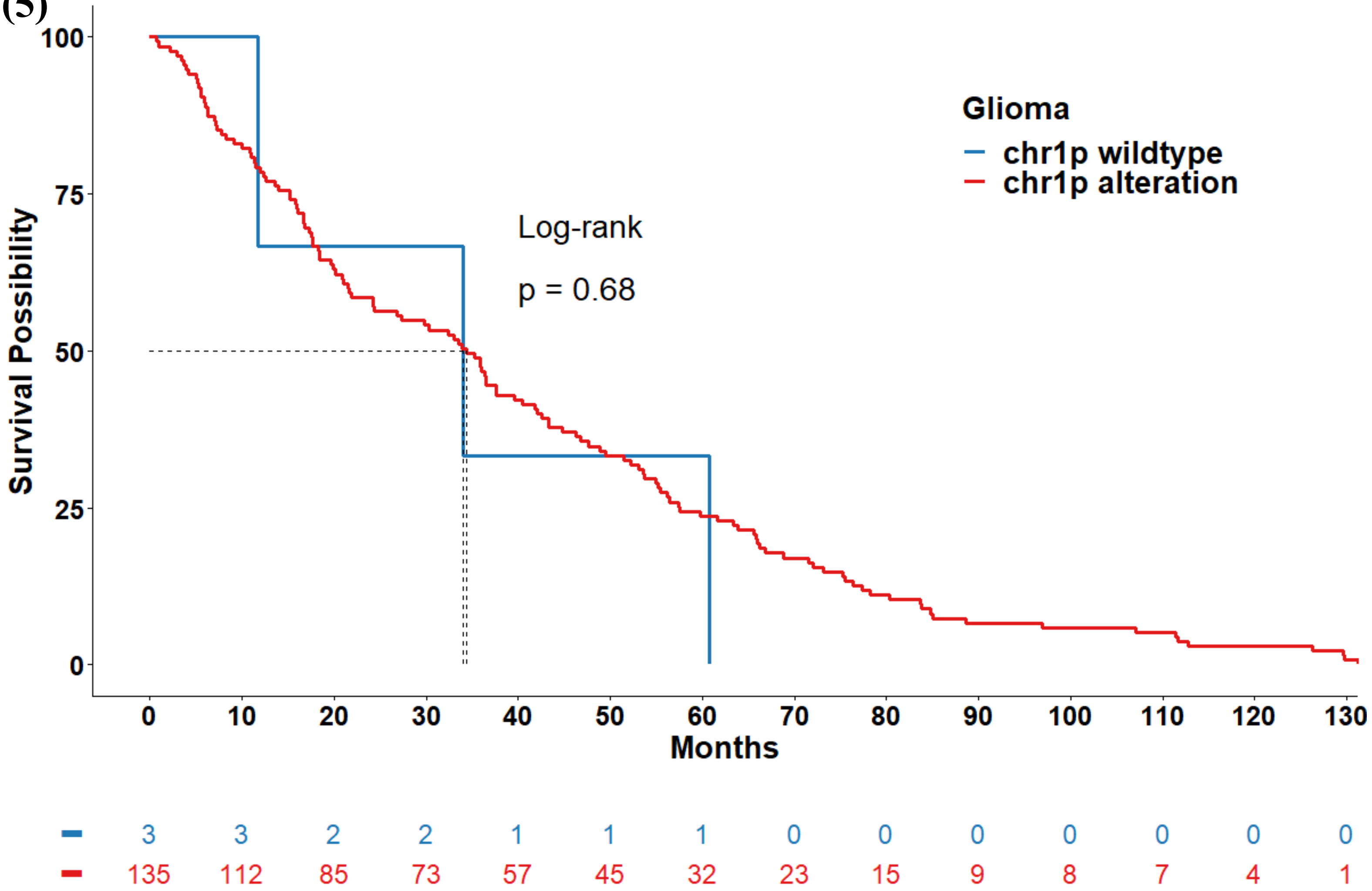

Glioma
chr1p wildtype
chr1p alteration
Log-rank
p = 0.68
Survival Possibility
100
75
50
25
0
0 10 20 30 40 50 60 70 80 90 100 110 120 130
Months
3 3 2 2 1 1 1 0 0 0 0 0 0 0
135 112 85 73 57 45 32 23 15 9 8 7 4 1

(6)

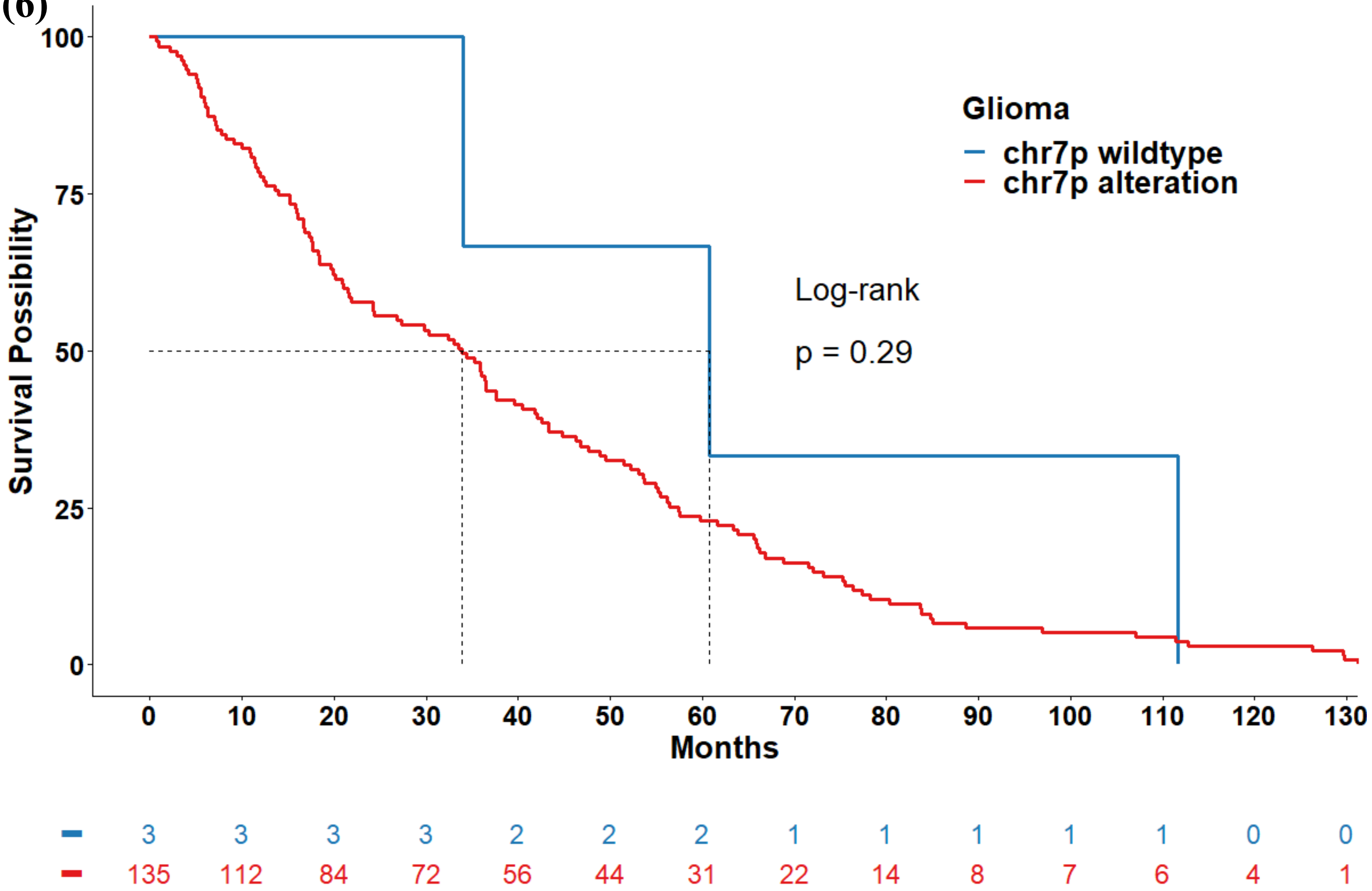
Survival Possibility
100
75
50
25
0
Glioma
chr7p wildtype
chr7p alteration
Log-rank
p = 0.29
0
10
20
30
40
50
60
70
80
90
100
110
120
130
Months
3 3 3 3 2 2 2 1 1 1 1 1 0 0
135 112 84 72 56 44 31 22 14 8 7 6 4 1

(7)

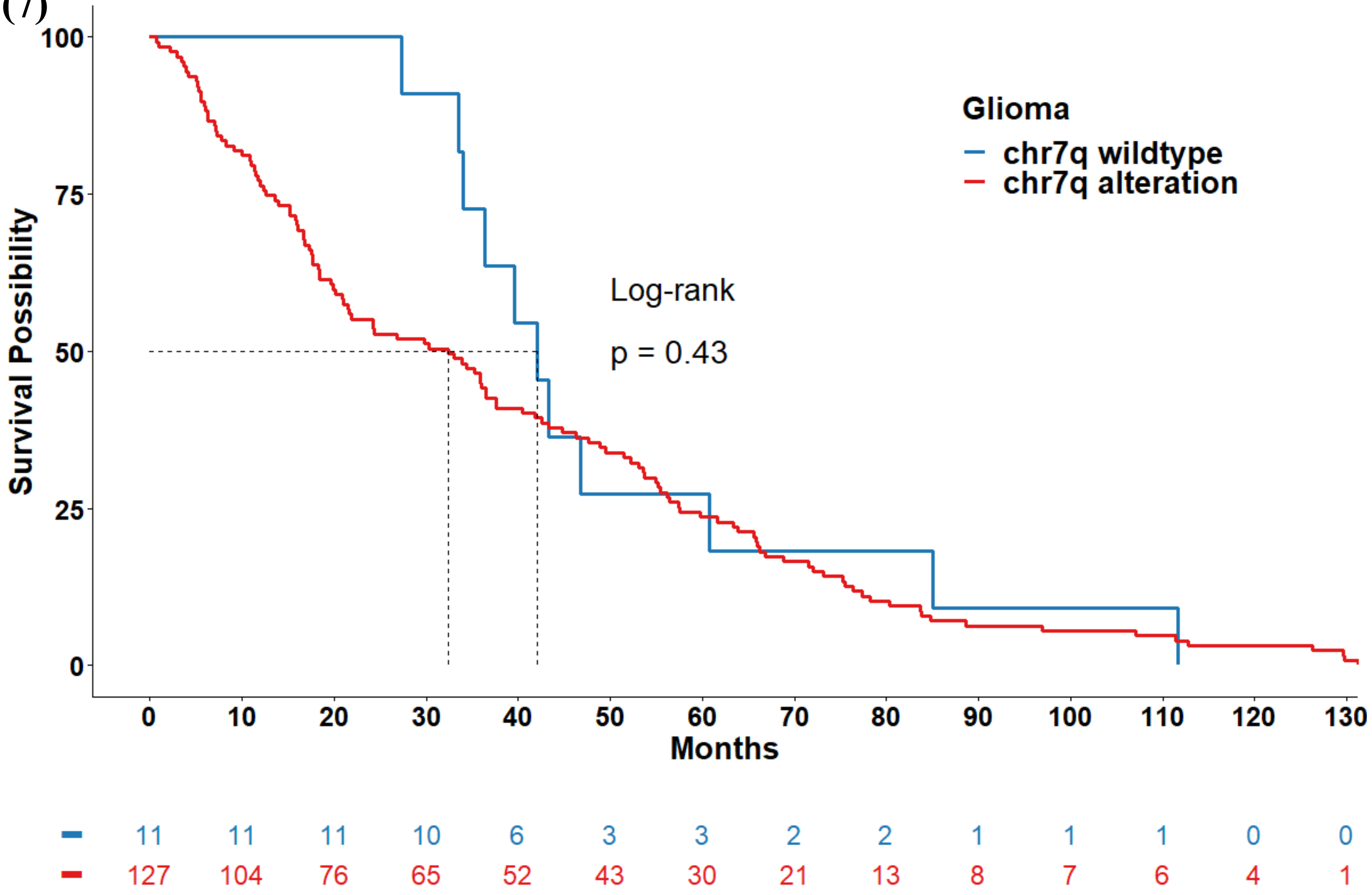
Survival Possibility
100
75
50
25
0
Glioma
chr7q wildtype
chr7q alteration
Log-rank
p = 0.43
0
10
20
30
40
50
60
70
80
90
100
110
120
130
Months
11 11 11 10 6 3 3 2 2 1 1 1 0 0
127 104 76 65 52 43 30 21 13 8 7 6 4 1

(8)

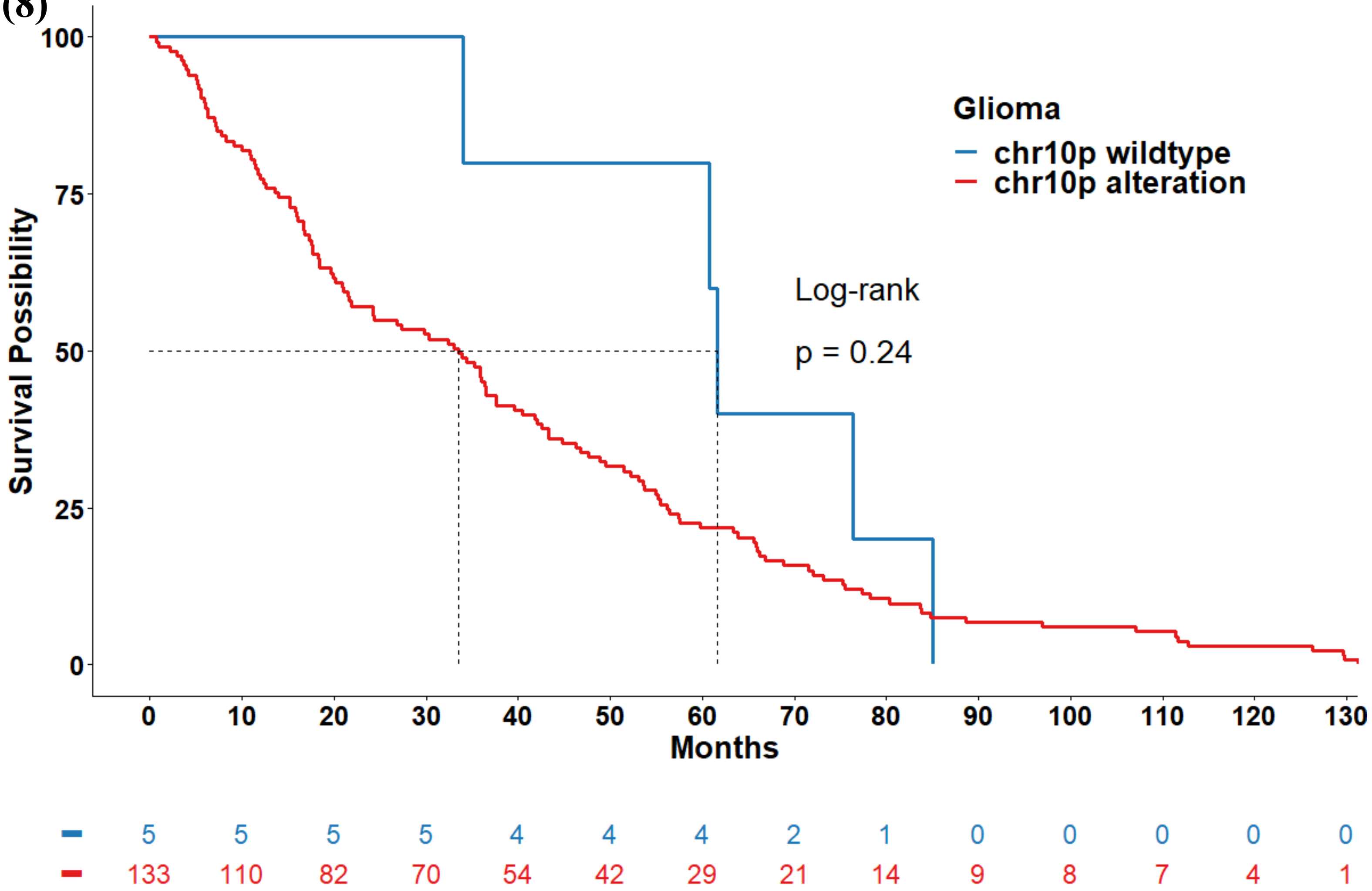
Survival Possibility
100
75
50
25
0
0 10 20 30 40 50 60 70 80 90 100 110 120 130
Months
Glioma
chr10p wildtype
chr10p alteration
Log-rank
p = 0.24
5 5 5 5 4 4 4 2 1 0 0 0 0 0
133 110 82 70 54 42 29 21 14 9 8 7 4 1

(9)

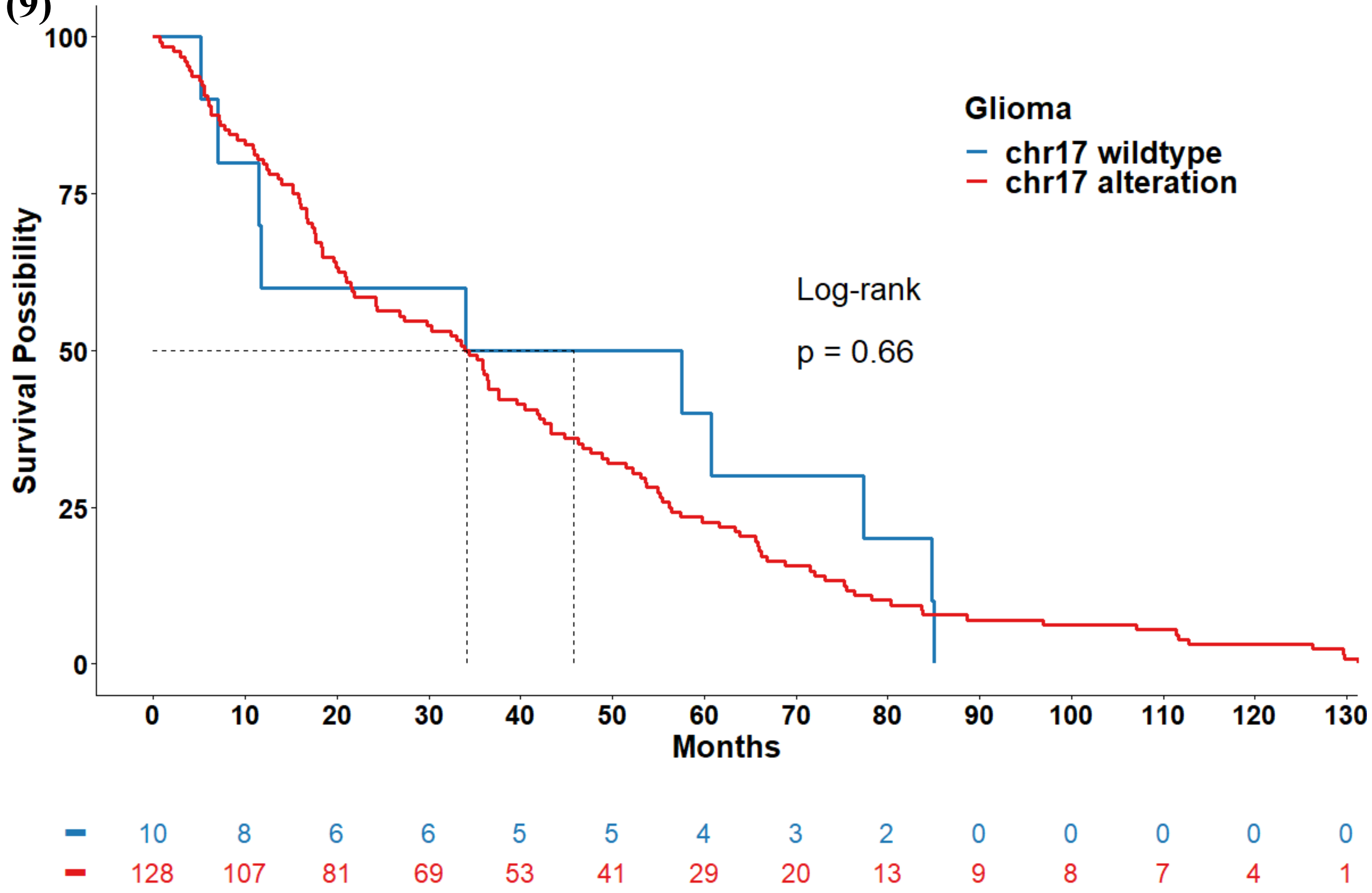

Glioma
chr17 wildtype
chr17 alteration
Log-rank
p = 0.66
Survival Possibility
100
75
50
25
0
0 10 20 30 40 50 60 70 80 90 100 110 120 130
Months
10 8 6 6 5 5 4 3 2 0 0 0 0 0
128 107 81 69 53 41 29 20 13 9 8 7 4 1

(10)

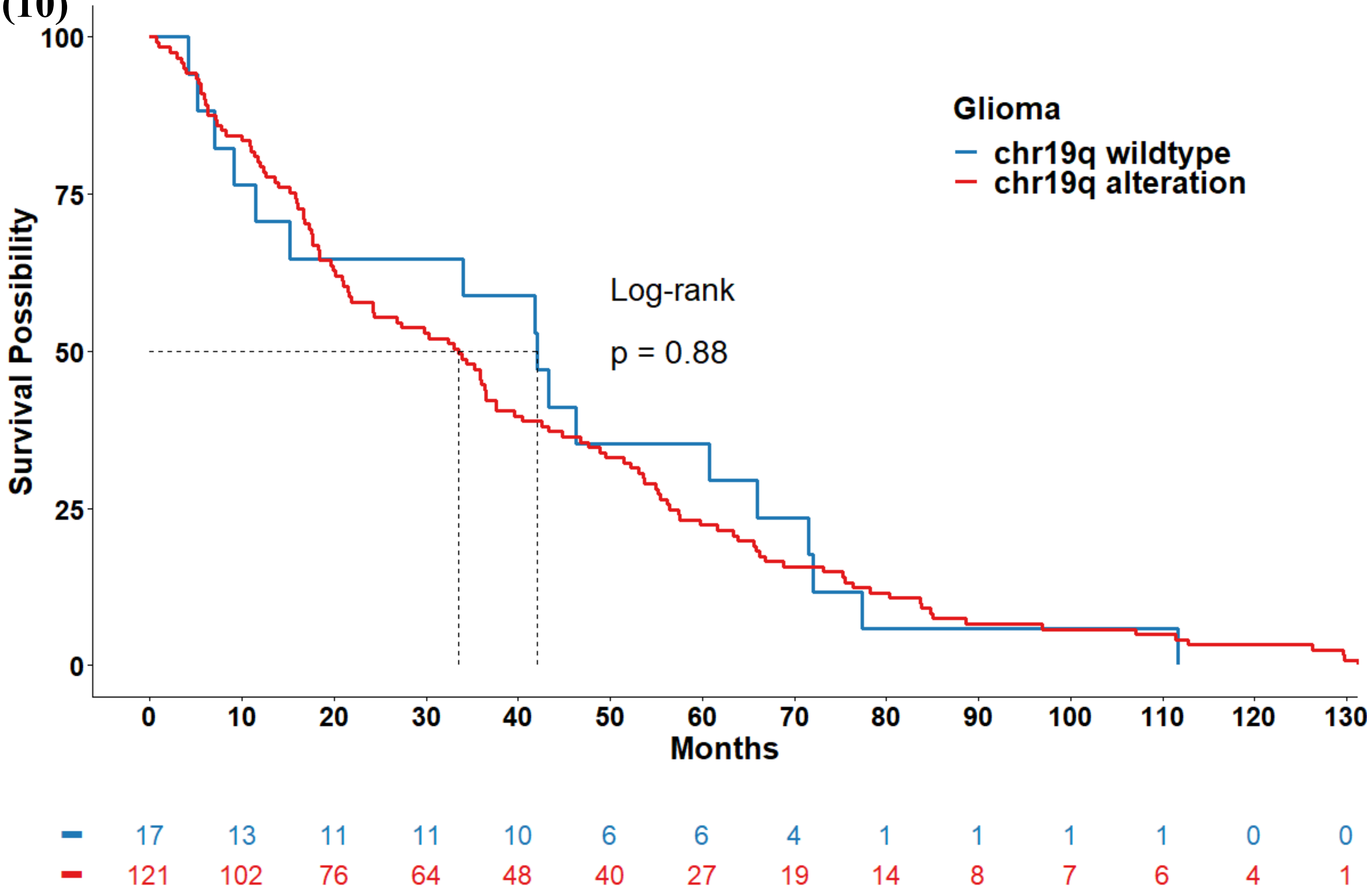

Survival Possibility
100
75
50
25
0
0 10 20 30 40 50 60 70 80 90 100 110 120 130
Months
Glioma
chr19q wildtype
chr19q alteration
Log-rank
p = 0.88
17 13 11 11 10 6 6 4 1 1 1 1 0 0
121 102 76 64 48 40 27 19 14 8 7 6 4 1

(11)

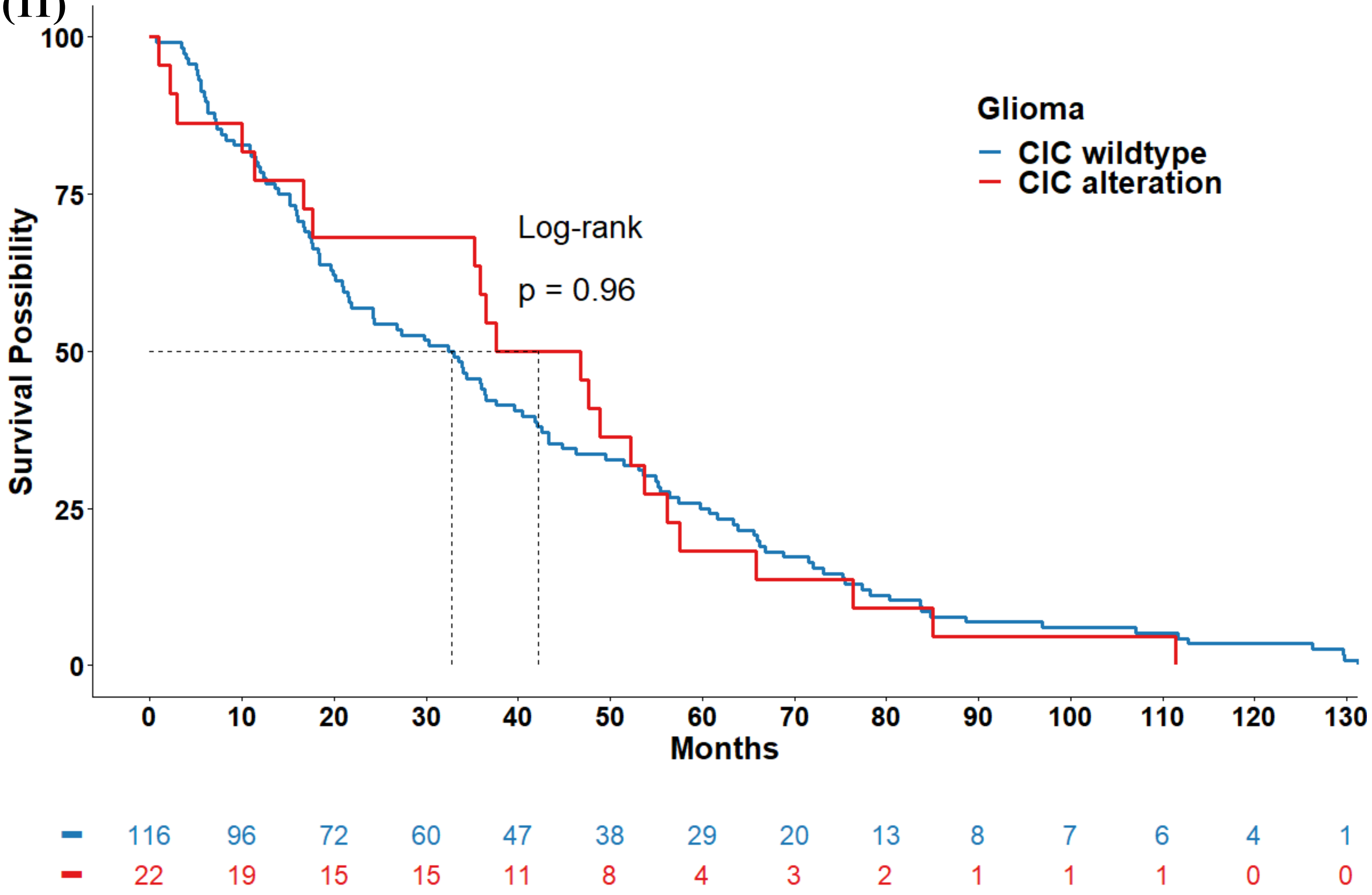

Survival Possibility
100
75
50
25
0
Log-rank
p = 0.96
Glioma
CIC wildtype
CIC alteration
0 10 20 30 40 50 60 70 80 90 100 110 120 130
Months
116 96 72 60 47 38 29 20 13 8 7 6 4 1
22 19 15 15 11 8 4 3 2 1 1 1 0 0

(12)

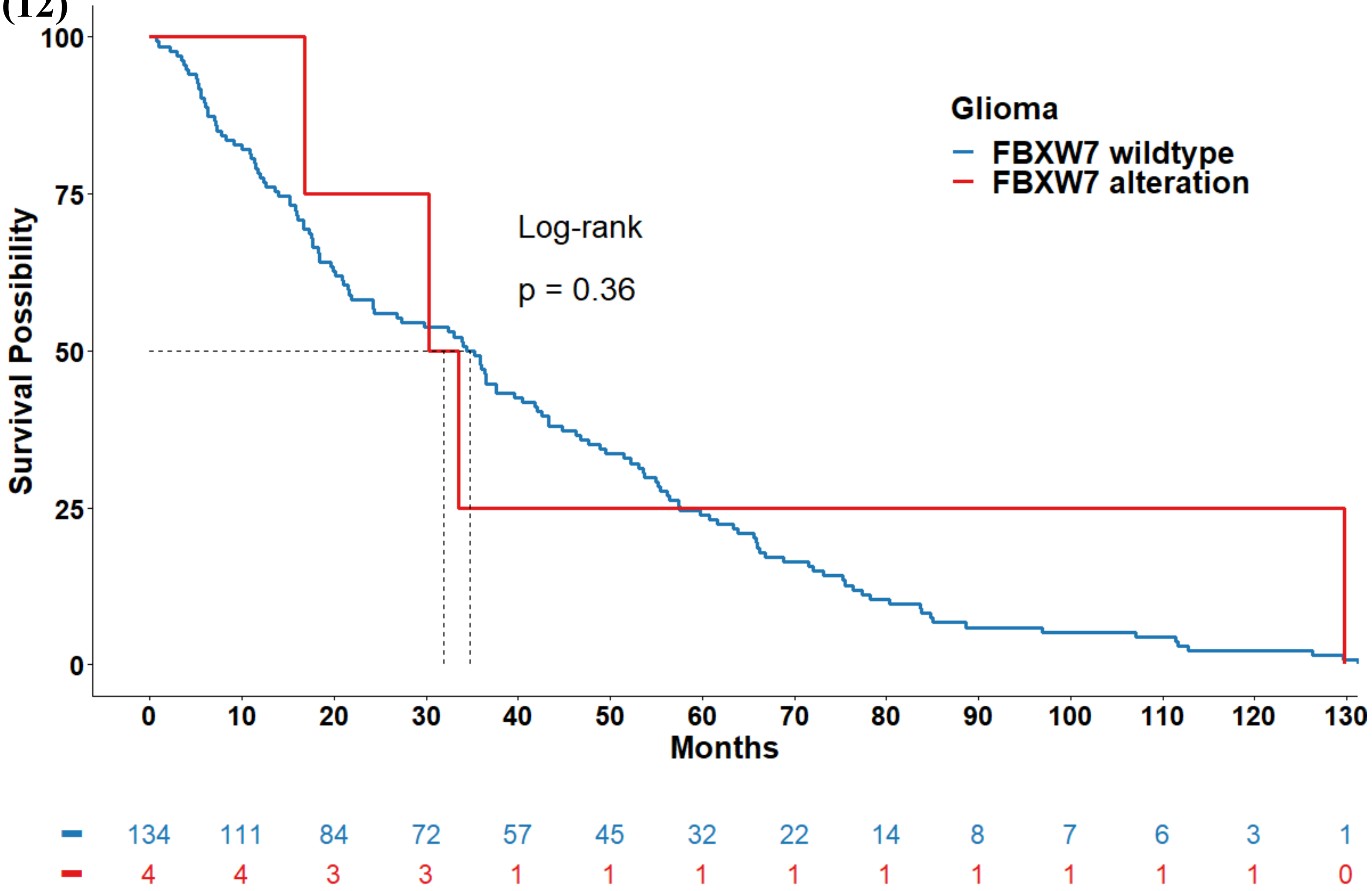

Survival Possibility
100
75
50
25
0
Glioma
FBXW7 wildtype
FBXW7 alteration
Log-rank
p = 0.36
0
10
20
30
40
50
60
70
80
90
100
110
120
130
Months
134 111 84 72 57 45 32 22 14 8 7 6 3 1
4 4 3 3 1 1 1 1 1 1 1 1 1 0

(13)

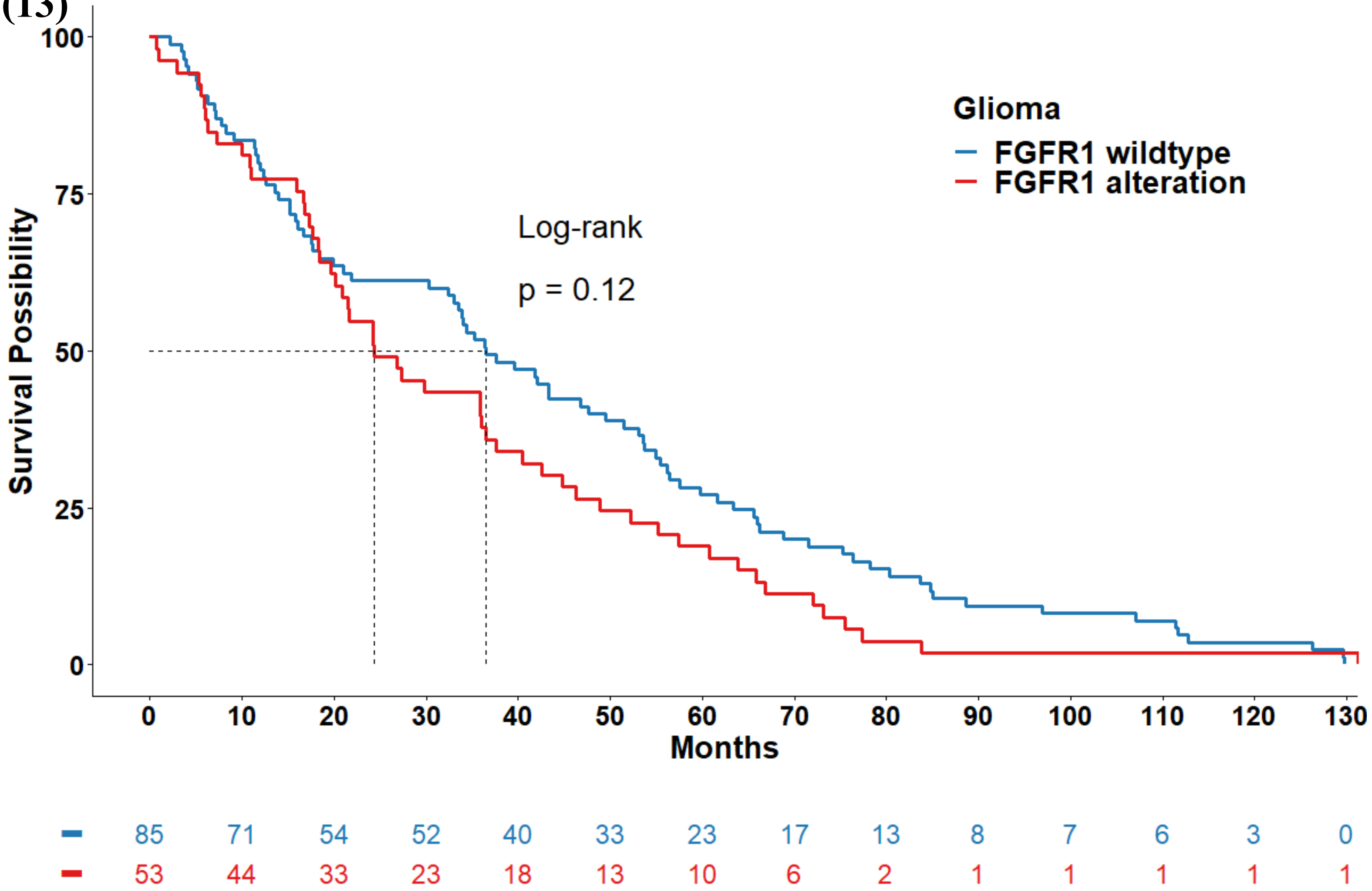

Glioma
FGFR1 wildtype
FGFR1 alteration
Log-rank
p = 0.12
Survival Possibility
100
75
50
25
0
0 10 20 30 40 50 60 70 80 90 100 110 120 130
Months
85 71 54 52 40 33 23 17 13 8 7 6 3 0
53 44 33 23 18 13 10 6 2 1 1 1 1 1

(14)

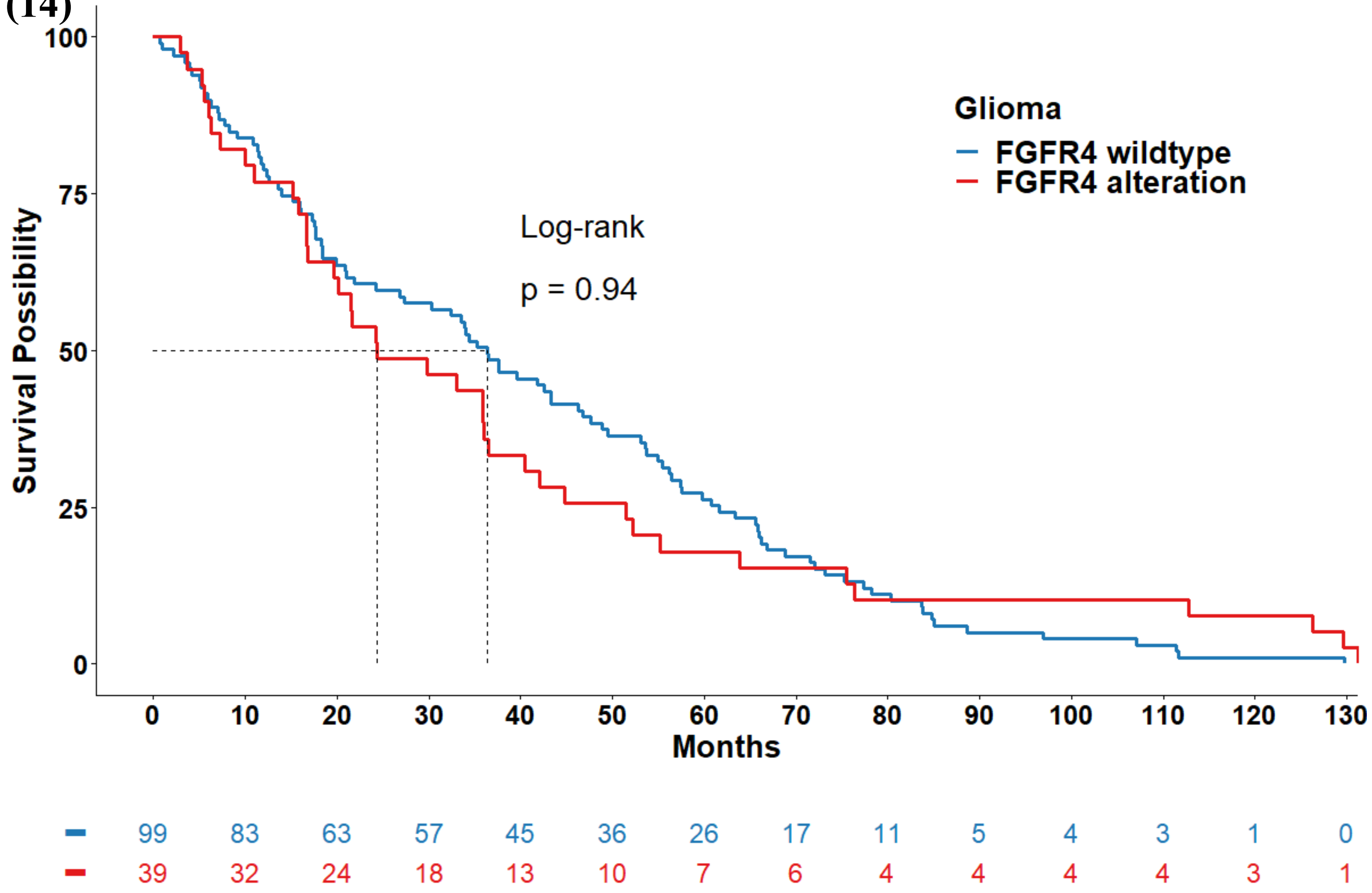

Survival Possibility
100
75
50
25
0
Glioma
FGFR4 wildtype
FGFR4 alteration
Log-rank
p = 0.94
0 10 20 30 40 50 60 70 80 90 100 110 120 130
Months
99 83 63 57 45 36 26 17 11 5 4 3 1 0
39 32 24 18 13 10 7 6 4 4 4 4 3 1

(15)

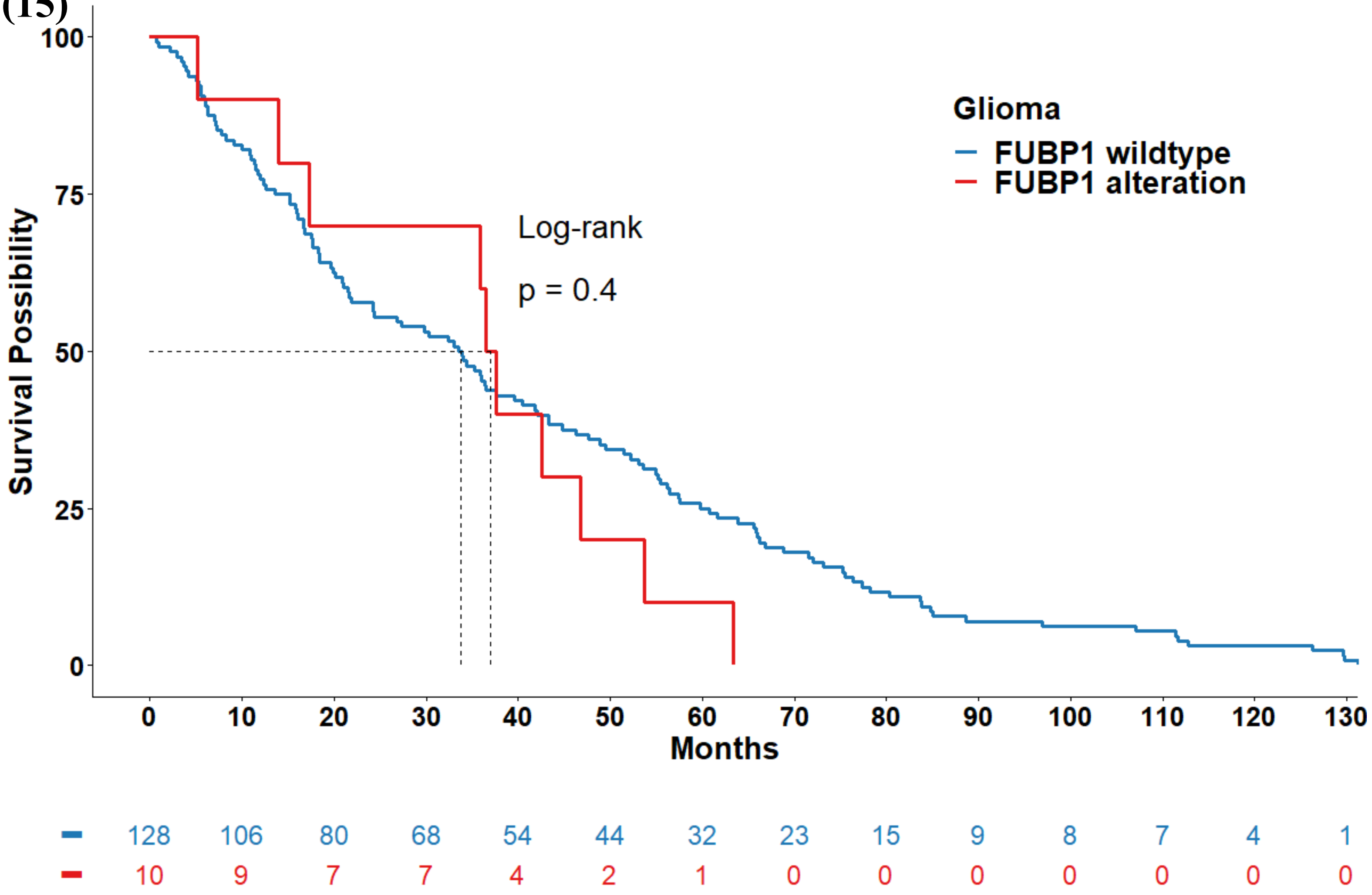
Glioma
FUBP1 wildtype
FUBP1 alteration
Log-rank
p = 0.4
Survival Possibility
100
75
50
25
0
0 10 20 30 40 50 60 70 80 90 100 110 120 130
Months
128 106 80 68 54 44 32 23 15 9 8 7 4 1
10 9 7 7 4 2 1 0 0 0 0 0 0 0

(16)

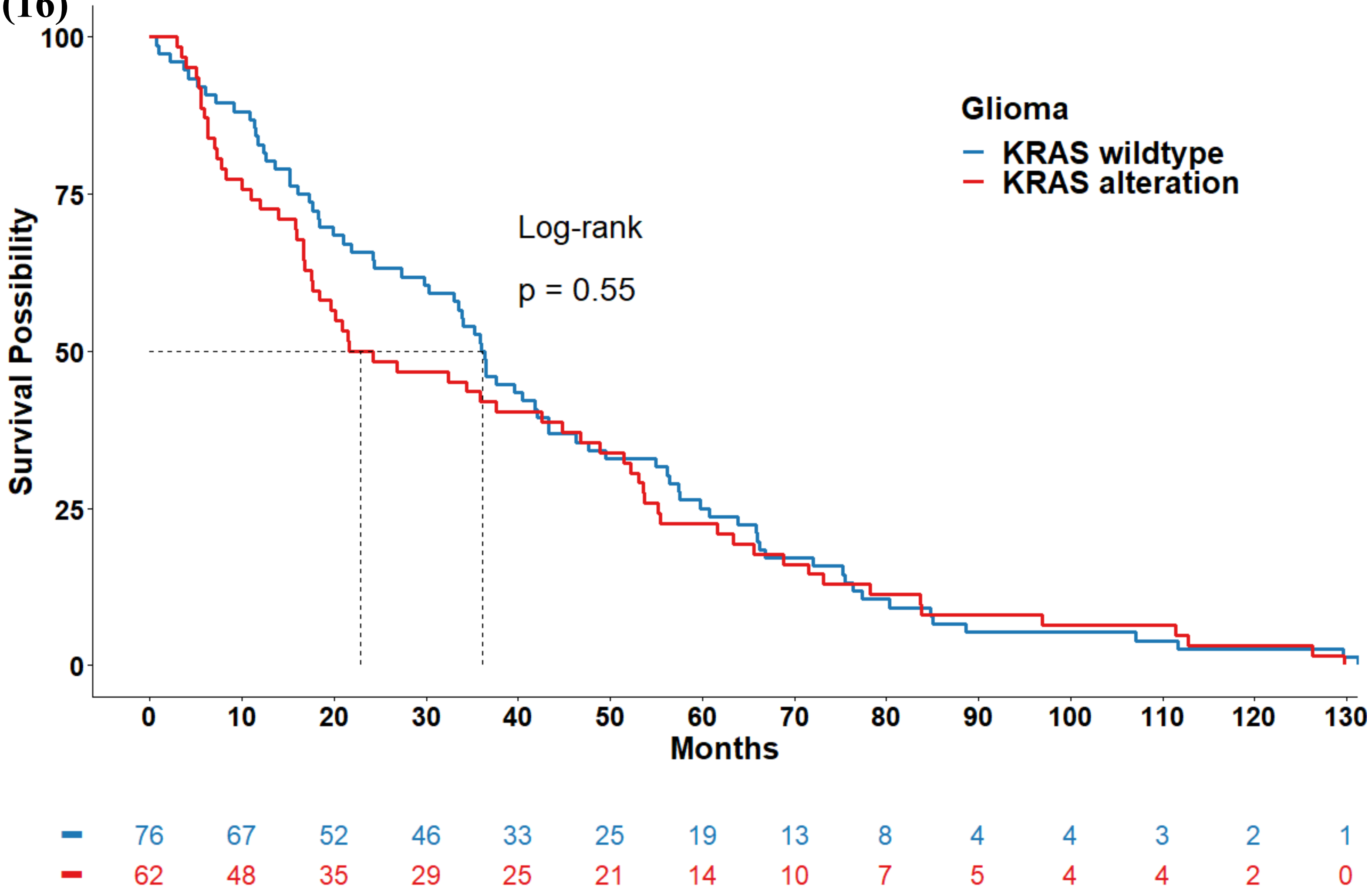
Survival Possibility
100
75
50
25
0
Log-rank
p = 0.55
Glioma
KRAS wildtype
KRAS alteration
0
10
20
30
40
50
60
70
80
90
100
110
120
130
Months
76 67 52 46 33 25 19 13 8 4 4 3 2 1
62 48 35 29 25 21 14 10 7 5 4 4 2 0

(17)

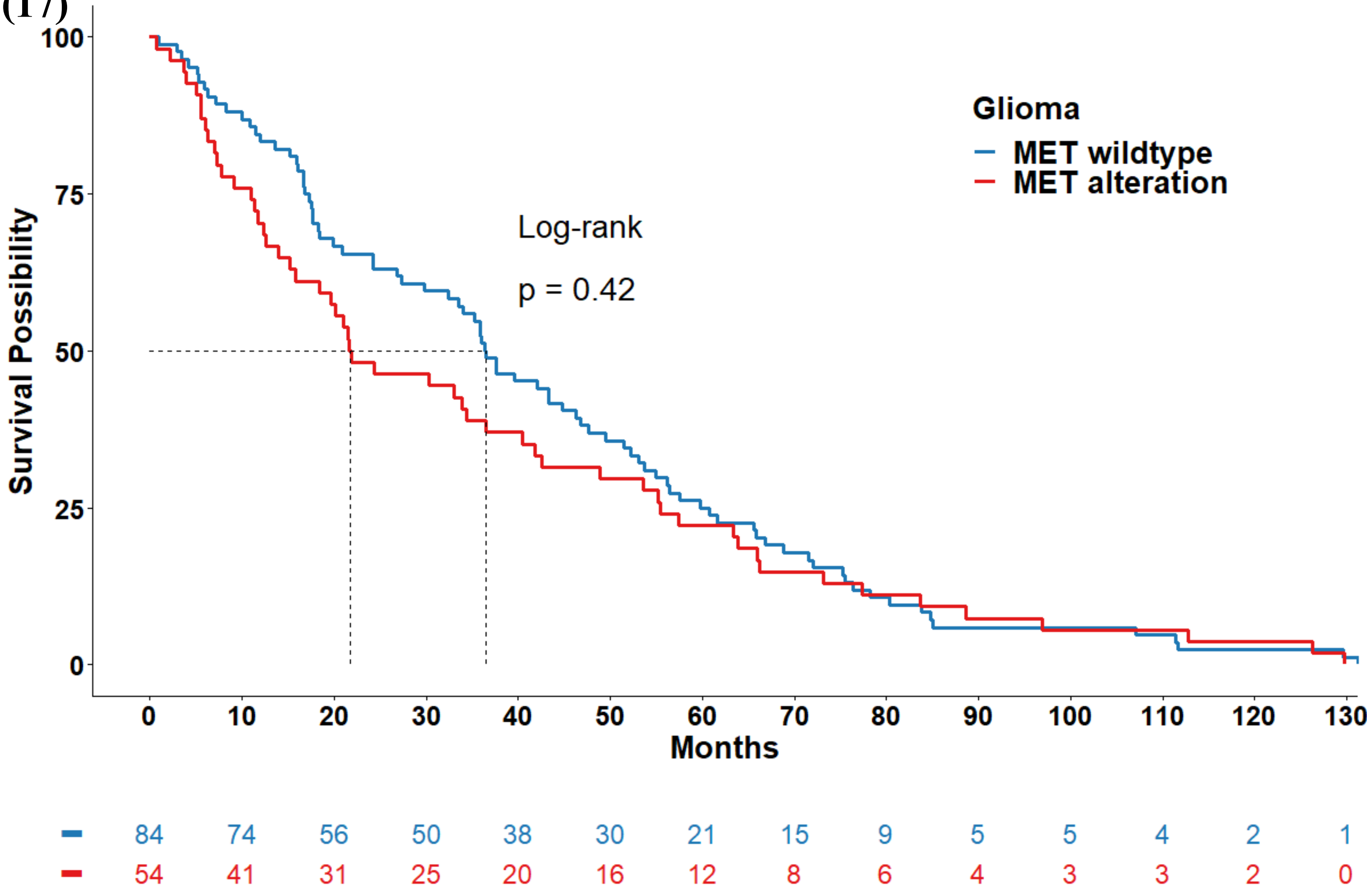

Survival Possibility
100
75
50
25
0
Glioma
MET wildtype
MET alteration
Log-rank
p = 0.42
0 10 20 30 40 50 60 70 80 90 100 110 120 130
Months
84 74 56 50 38 30 21 15 9 5 5 4 2 1
54 41 31 25 20 16 12 8 6 4 3 3 2 0

(18)

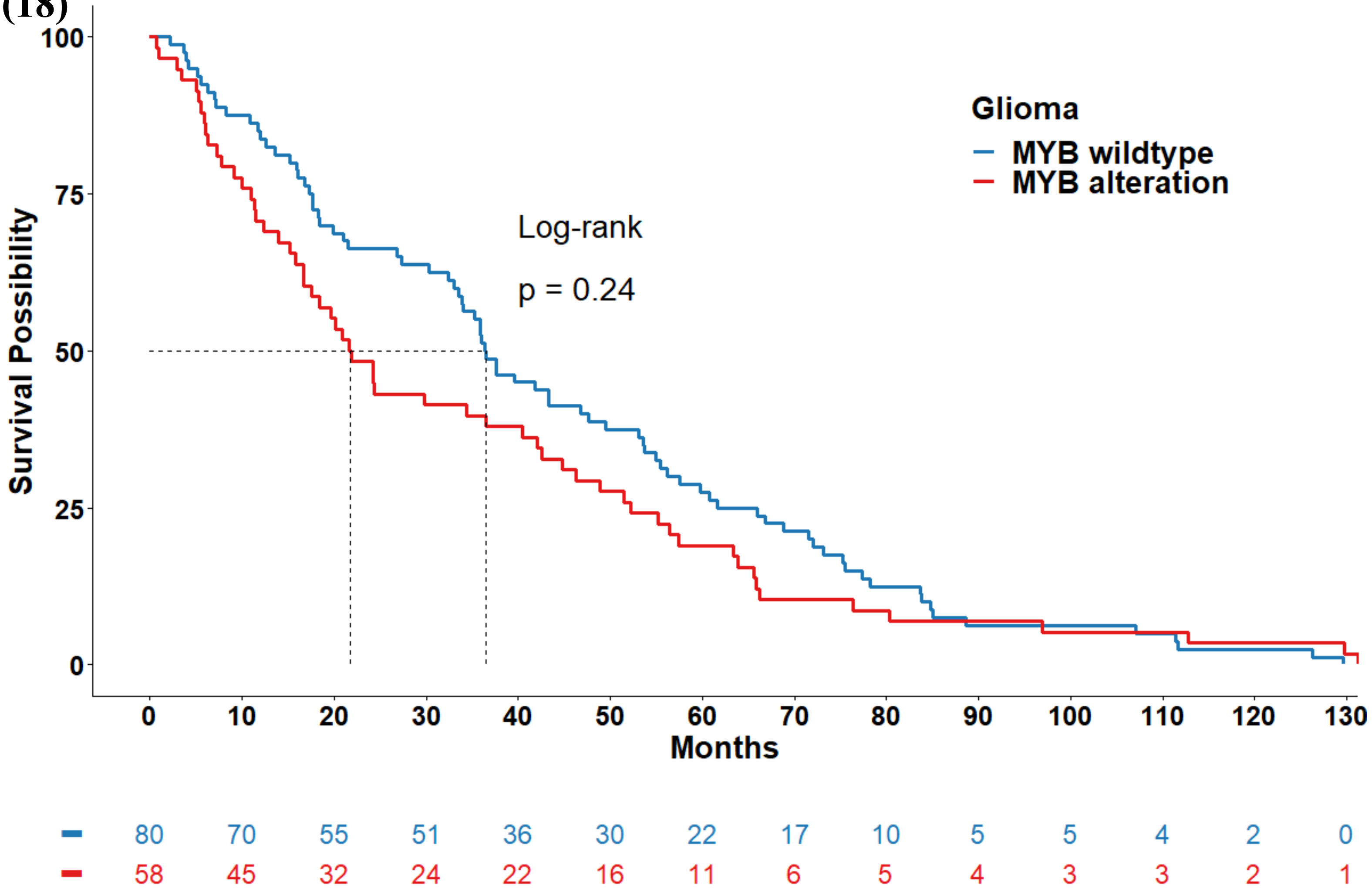

Glioma
MYB wildtype
MYB alteration
Log-rank
p = 0.24
Survival Possibility
100
75
50
25
0
0
10
20
30
40
50
60
70
80
90
100
110
120
130
Months
80 70 55 51 36 30 22 17 10 5 5 4 2 0
58 45 32 24 22 16 11 6 5 4 3 3 2 1

(19)

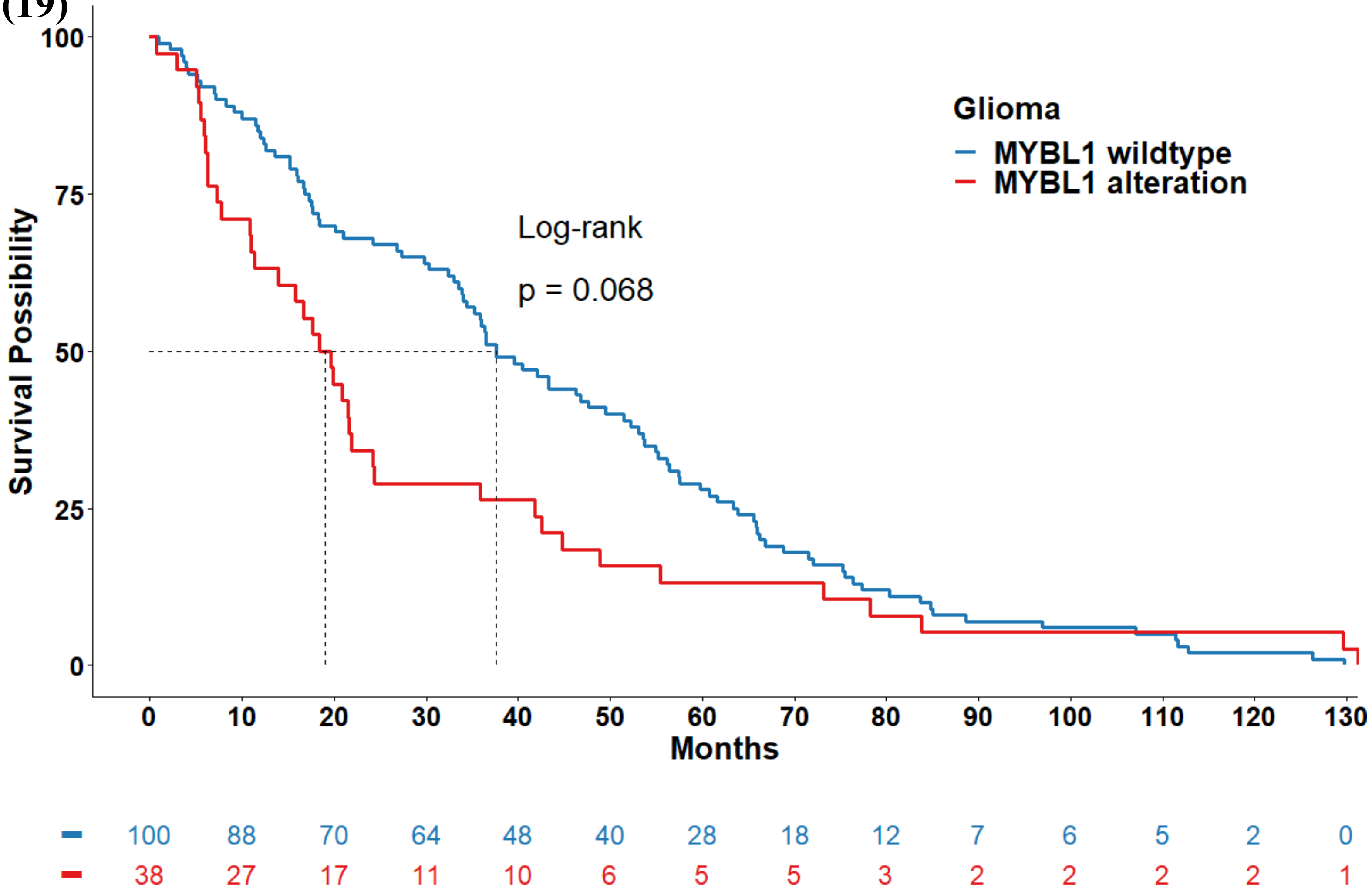

Glioma
MYBL1 wildtype
MYBL1 alteration
Log-rank
p = 0.068
Survival Possibility
100
75
50
25
0
0 10 20 30 40 50 60 70 80 90 100 110 120 130
Months
100 88 70 64 48 40 28 18 12 7 6 5 2 0
38 27 17 11 10 6 5 5 3 2 2 2 2 1

(20)

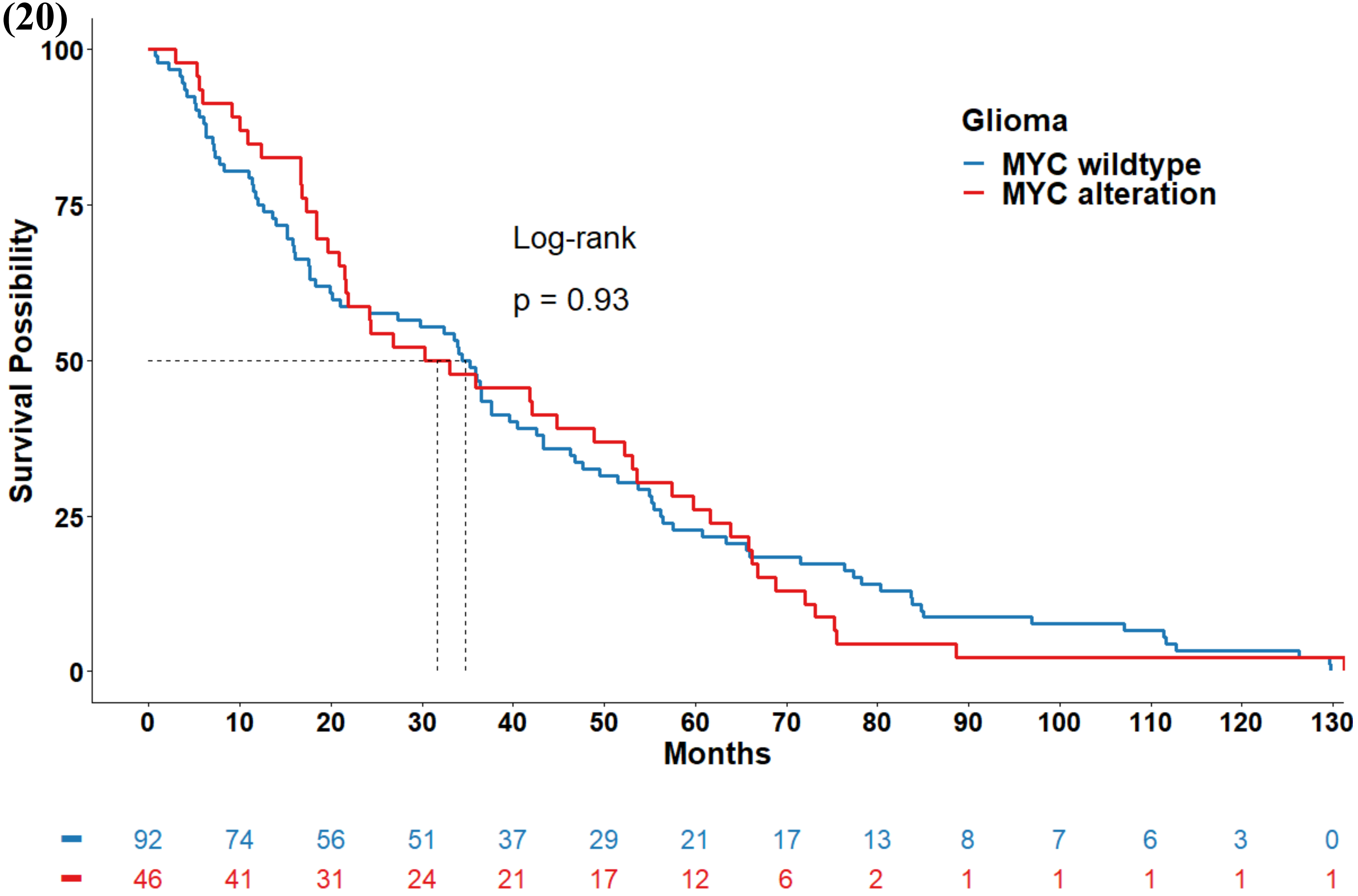

Survival Possibility
100
75
50
25
0
Glioma
MYC wildtype
MYC alteration
Log-rank
p = 0.93
0
10
20
30
40
50
60
70
80
90
100
110
120
130
Months
92 74 56 51 37 29 21 17 13 8 7 6 3 0
46 41 31 24 21 17 12 6 2 1 1 1 1 1

(21)

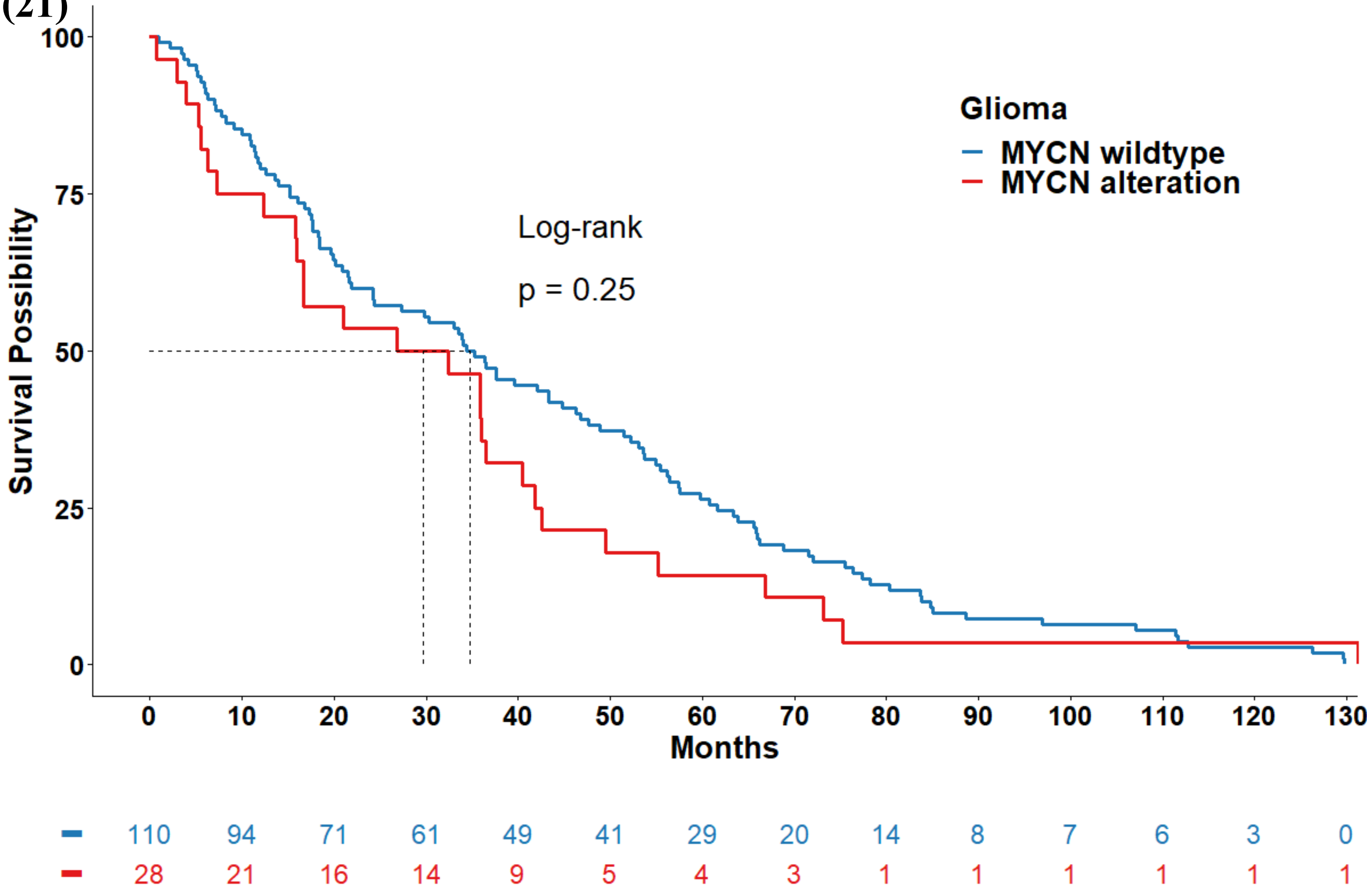
Survival Possibility
100
75
50
25
0
Log-rank
p = 0.25
Glioma
MYCN wildtype
MYCN alteration
0
10
20
30
40
50
60
70
80
90
100
110
120
130
Months
110 94 71 61 49 41 29 20 14 8 7 6 3 0
28 21 16 14 9 5 4 3 1 1 1 1 1 1

(22)

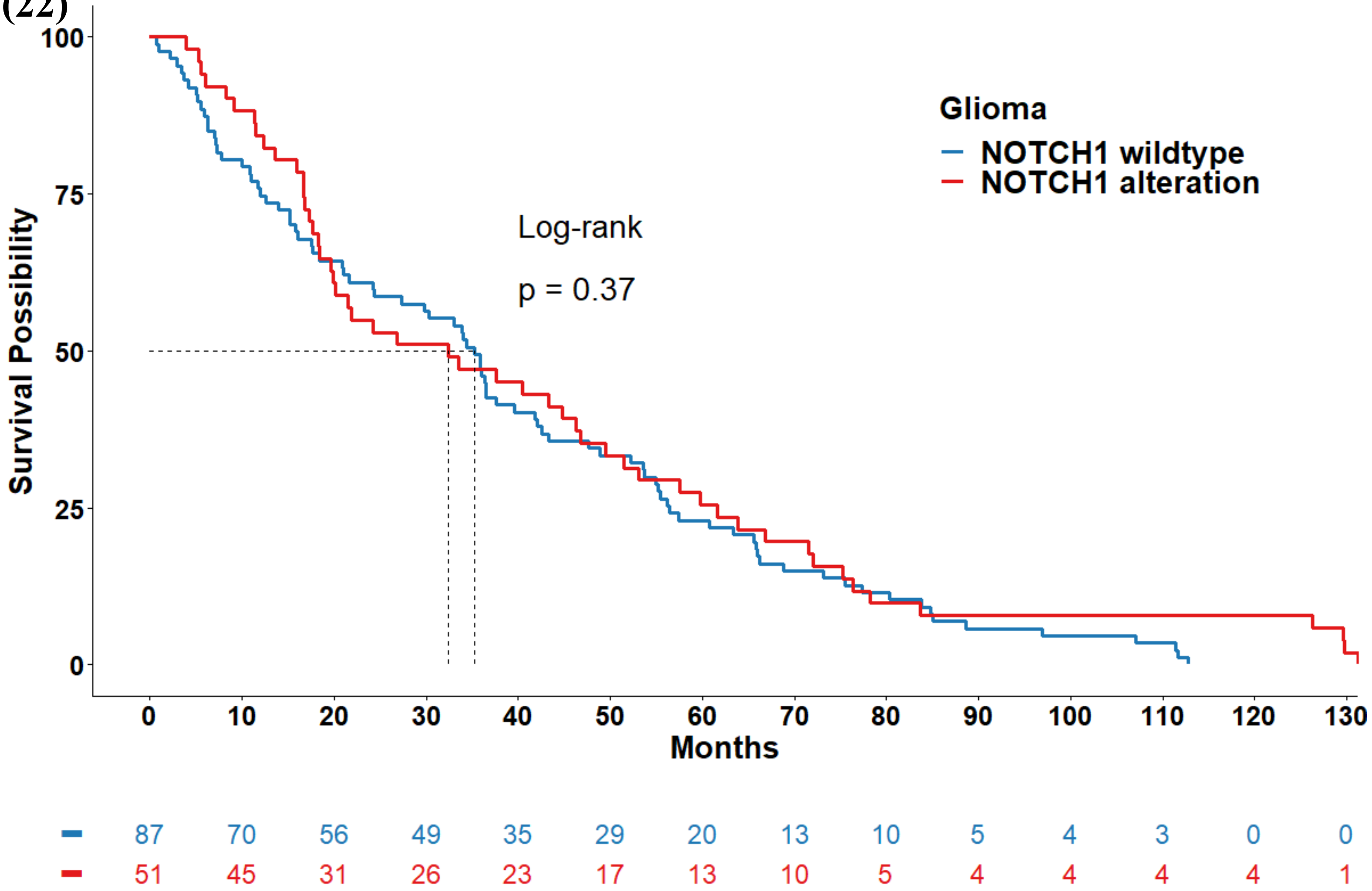
Glioma
NOTCH1 wildtype
NOTCH1 alteration
Log-rank
p = 0.37
Survival Possibility
100
75
50
25
0
0 10 20 30 40 50 60 70 80 90 100 110 120 130
Months
87 70 56 49 35 29 20 13 10 5 4 3 0 0
51 45 31 26 23 17 13 10 5 4 4 4 4 1

(23)

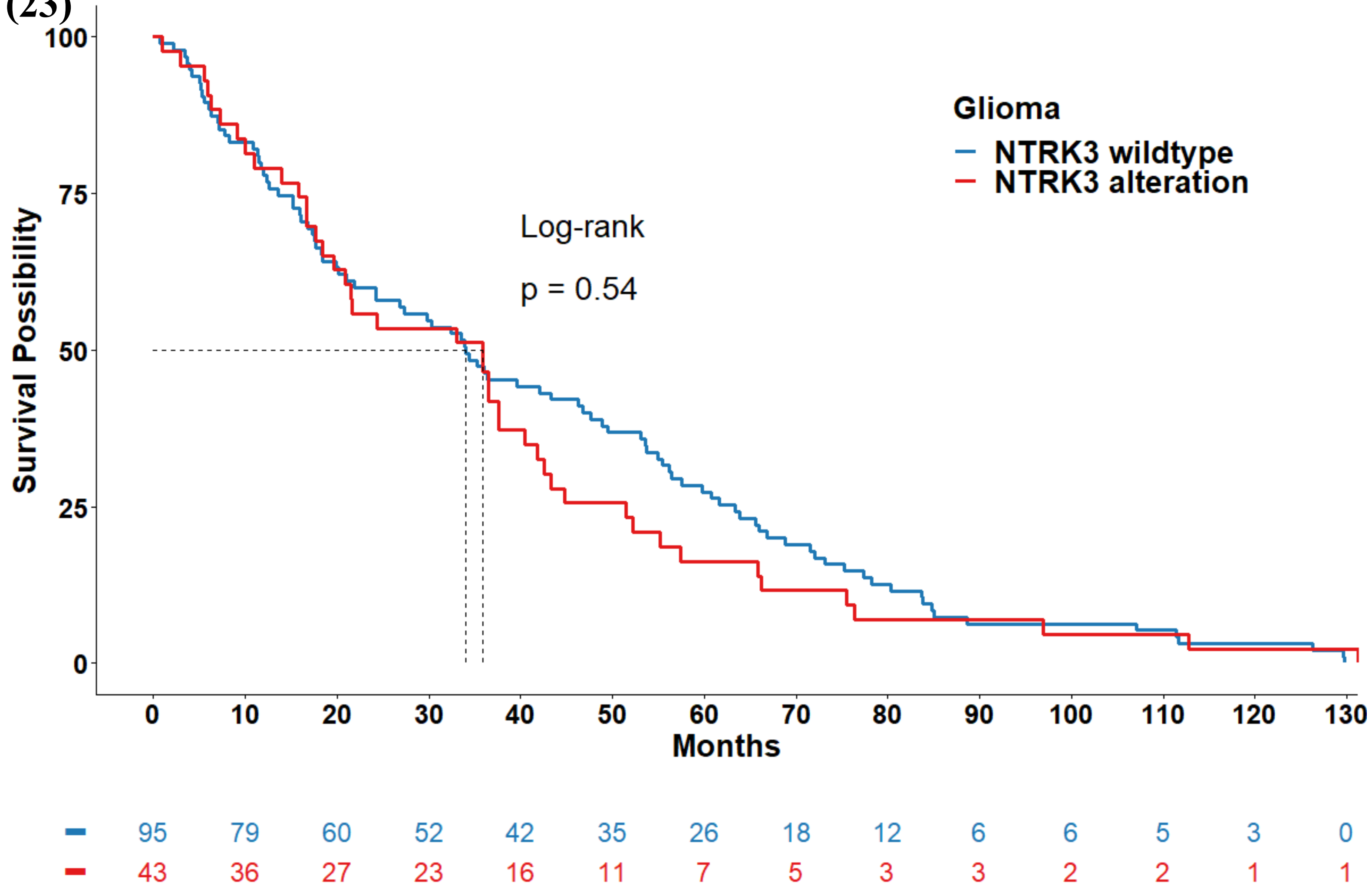

Glioma
NTRK3 wildtype
NTRK3 alteration
Log-rank
p = 0.54
Survival Possibility
100
75
50
25
0
0 10 20 30 40 50 60 70 80 90 100 110 120 130
Months
95 79 60 52 42 35 26 18 12 6 6 5 3 0
43 36 27 23 16 11 7 5 3 3 2 2 1 1

(24)

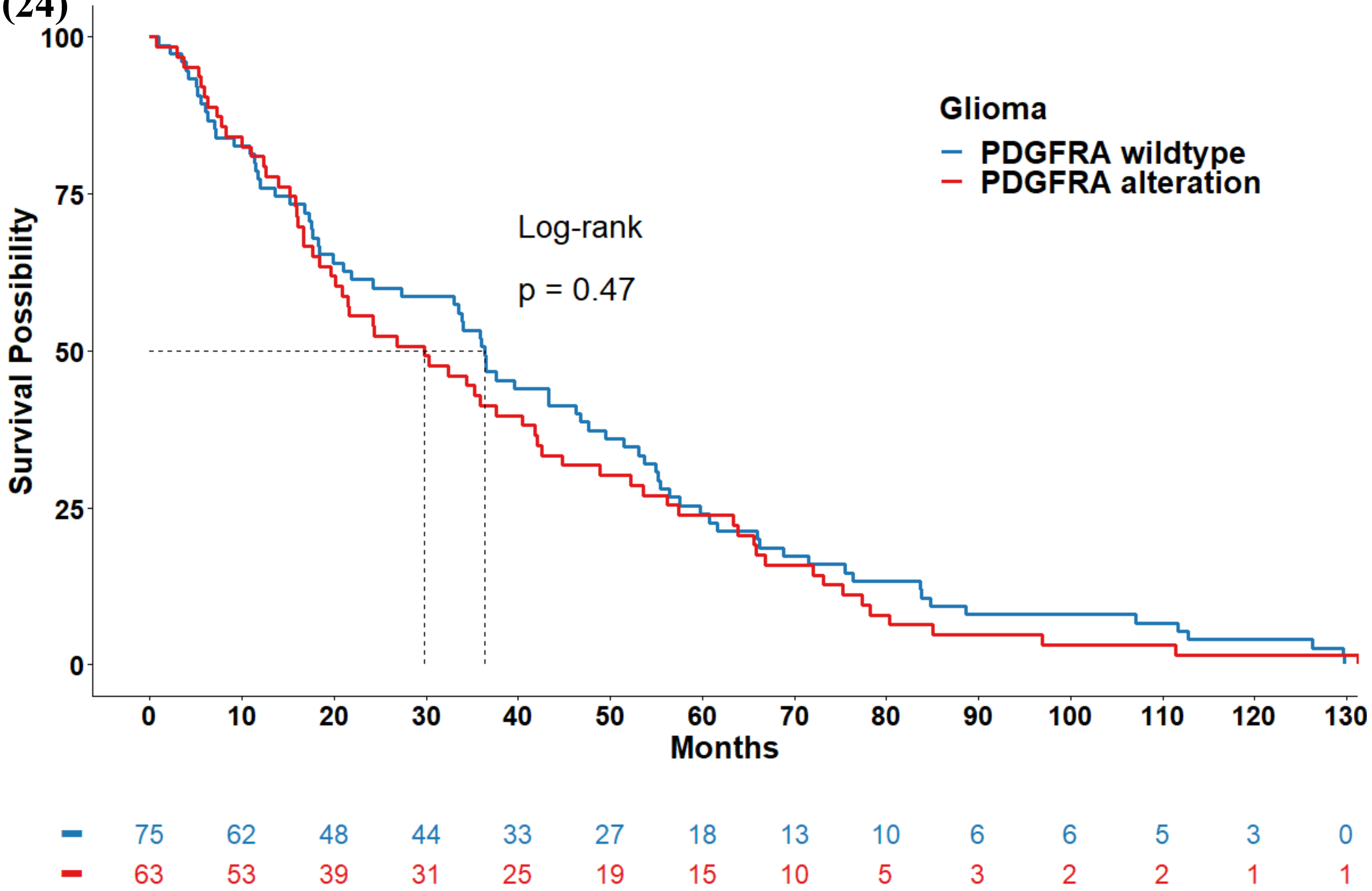

Glioma
PDGFRA wildtype
PDGFRA alteration
Log-rank
p = 0.47
Survival Possibility
100
75
50
25
0
0 10 20 30 40 50 60 70 80 90 100 110 120 130
Months
75 62 48 44 33 27 18 13 10 6 6 5 3 0
63 53 39 31 25 19 15 10 5 3 2 2 1 1

(25)

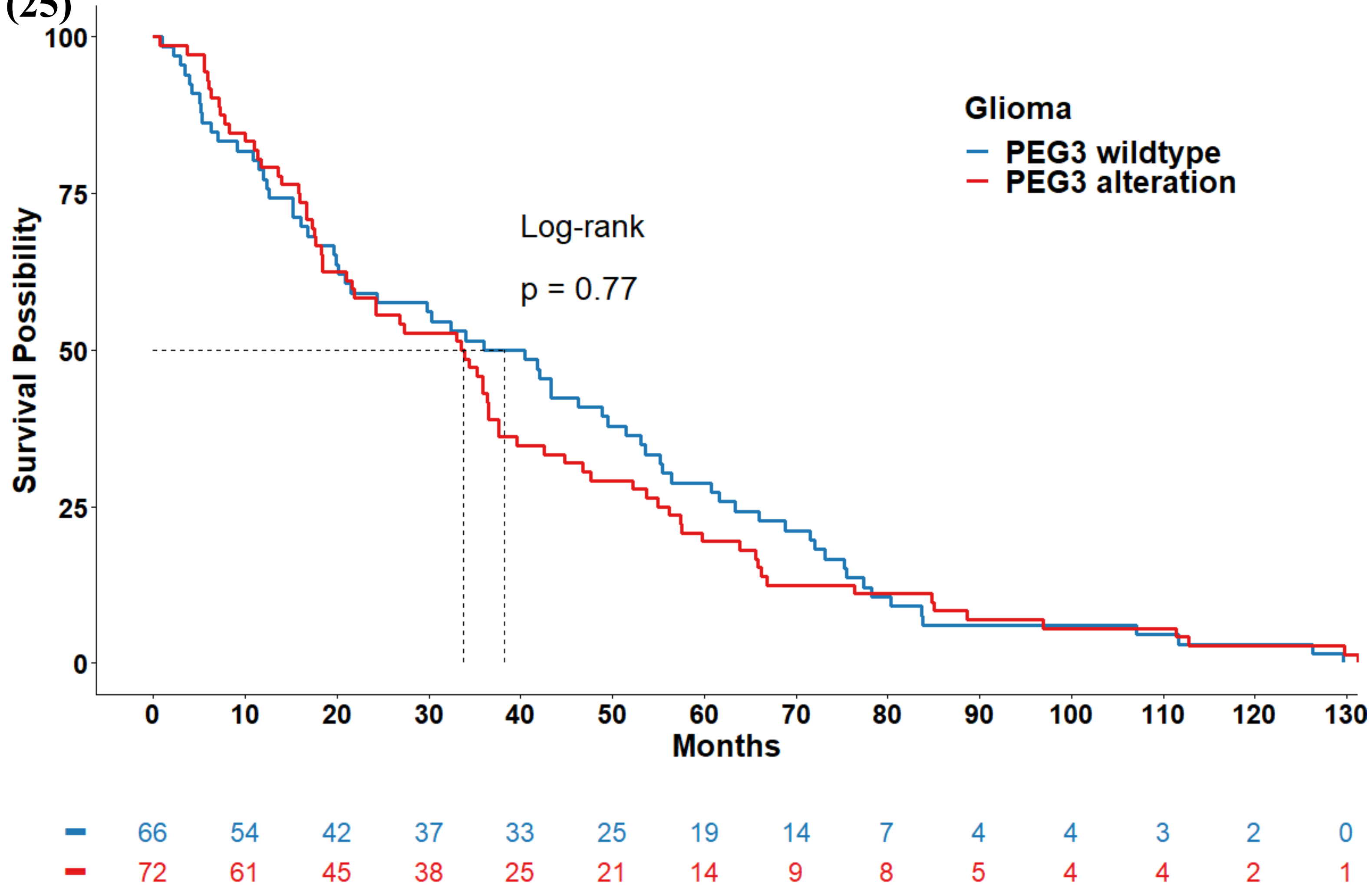

Glioma
PEG3 wildtype
PEG3 alteration
Log-rank
p = 0.77
Survival Possibility
100
75
50
25
0
0 10 20 30 40 50 60 70 80 90 100 110 120 130
Months
66 54 42 37 33 25 19 14 7 4 4 3 2 0
72 61 45 38 25 21 14 9 8 5 4 4 2 1

(26)

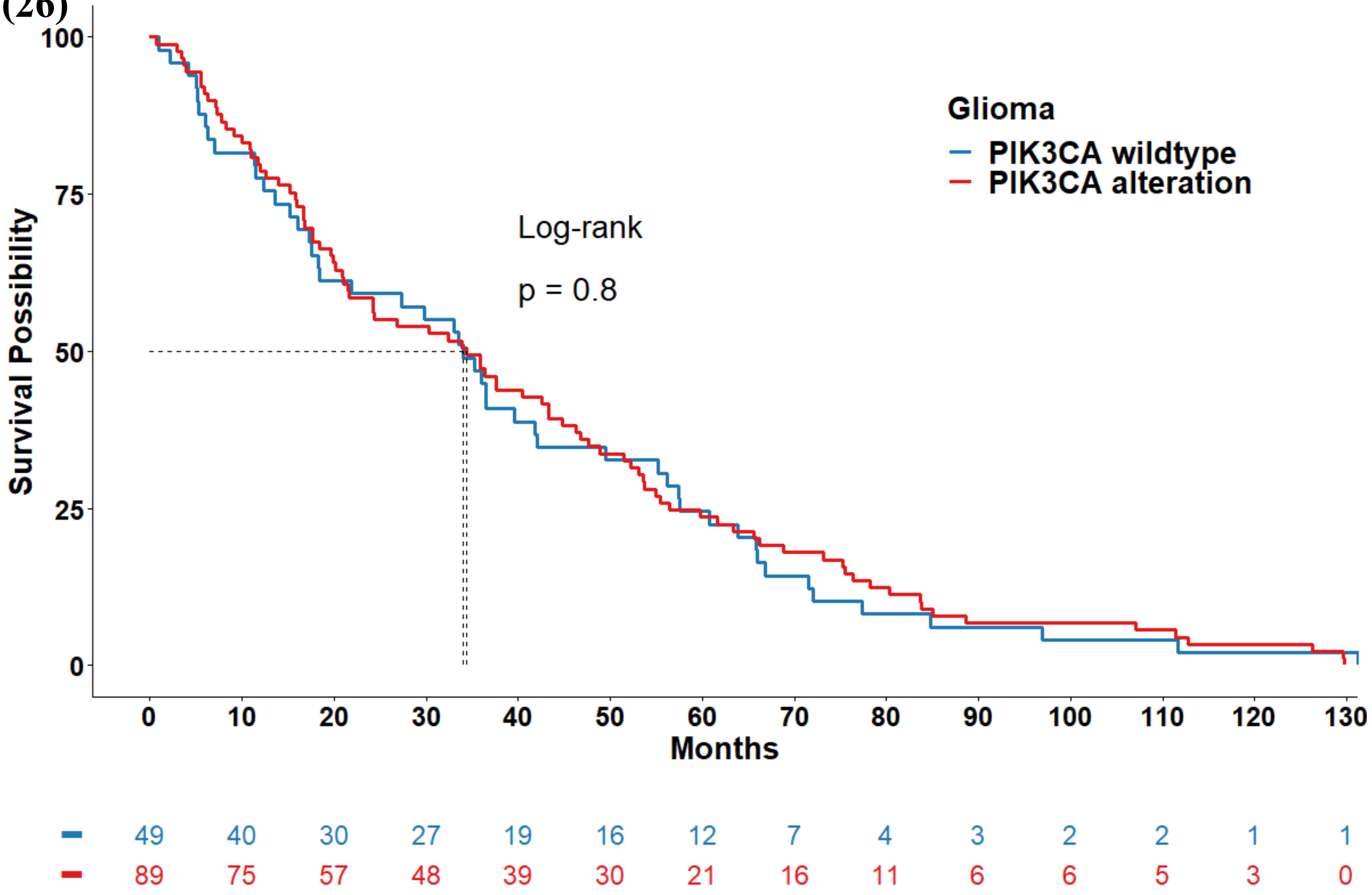

100
75
50
25
0
Survival Possibility
0
10
20
30
40
50
60
70
80
90
100
110
120
130
Months
Glioma
PIK3CA wildtype
PIK3CA alteration
Log-rank
p = 0.8
49 40 30 27 19 16 12 7 4 3 2 2 1 1
89 75 57 48 39 30 21 16 11 6 6 5 3 0

(27)

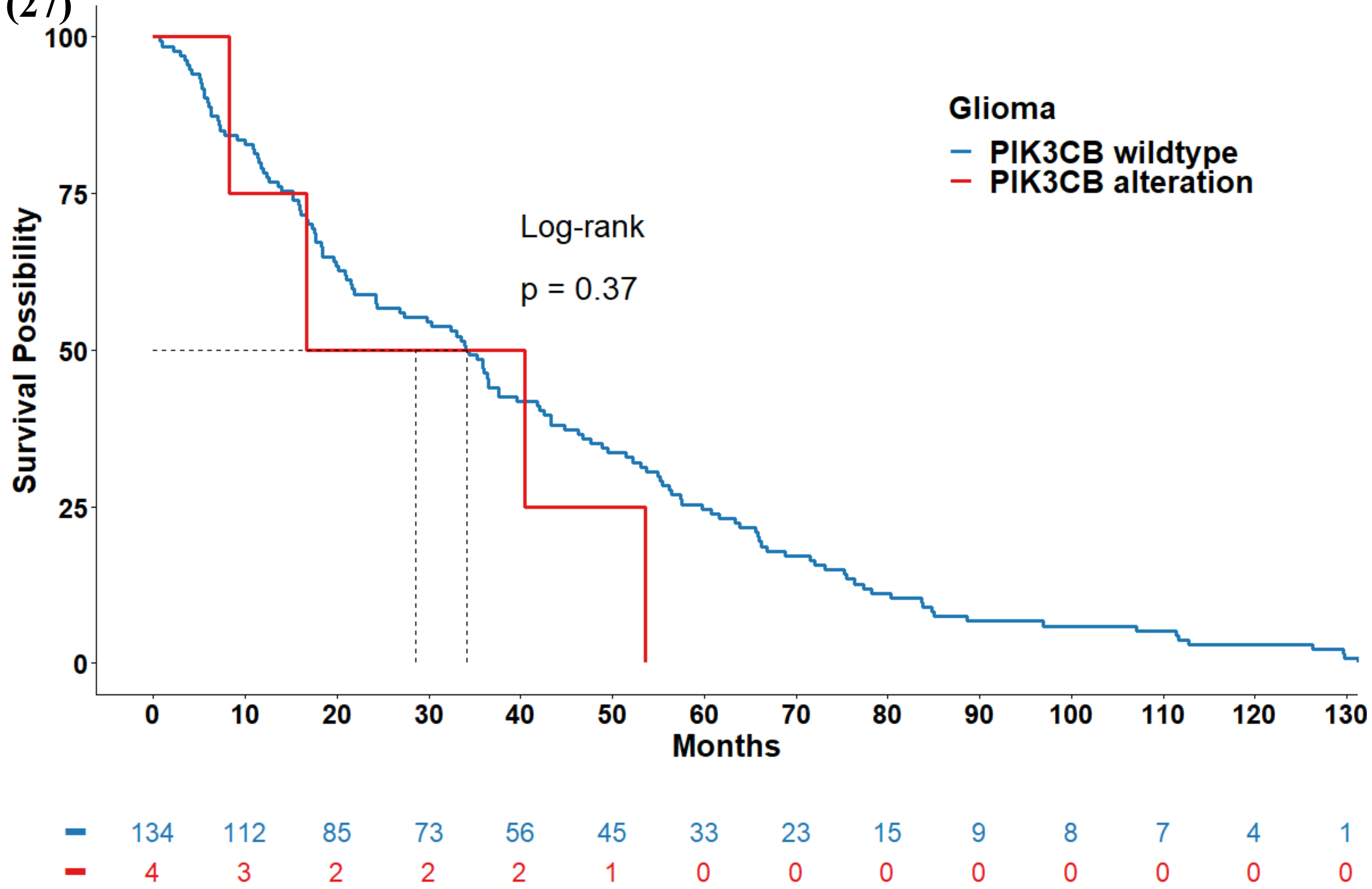

Survival Possibility
100
75
50
25
0
Glioma
PIK3CB wildtype
PIK3CB alteration
Log-rank
p = 0.37
0
10
20
30
40
50
60
70
80
90
100
110
120
130
Months
134 112 85 73 56 45 33 23 15 9 8 7 4 1
4 3 2 2 2 1 0 0 0 0 0 0 0 0

(28)

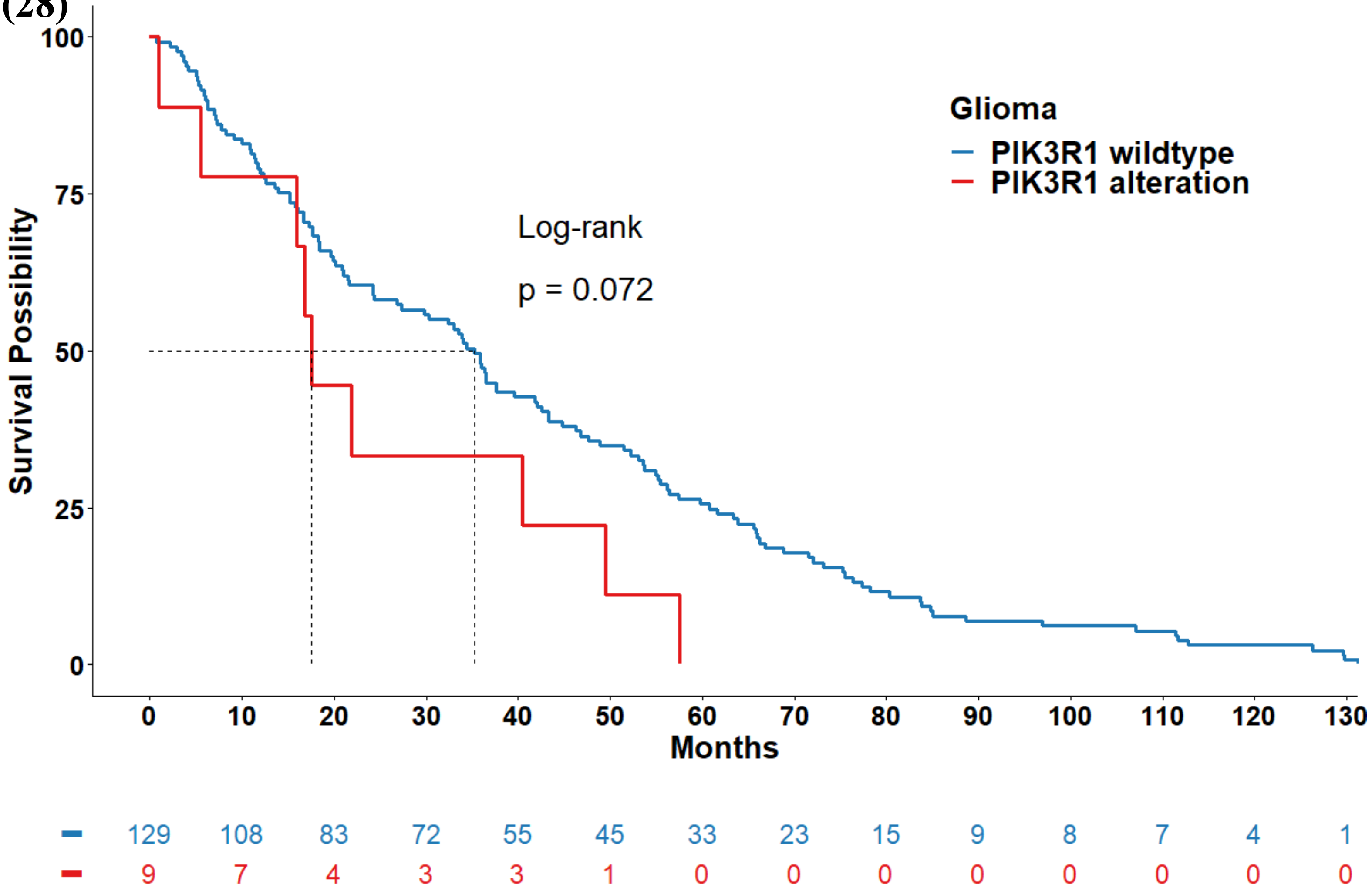
Survival Possibility
100
75
50
25
0
Log-rank
p = 0.072
Glioma
PIK3R1 wildtype
PIK3R1 alteration
0 10 20 30 40 50 60 70 80 90 100 110 120 130
Months
129 108 83 72 55 45 33 23 15 9 8 7 4 1
9 7 4 3 3 1 0 0 0 0 0 0 0 0

(29)

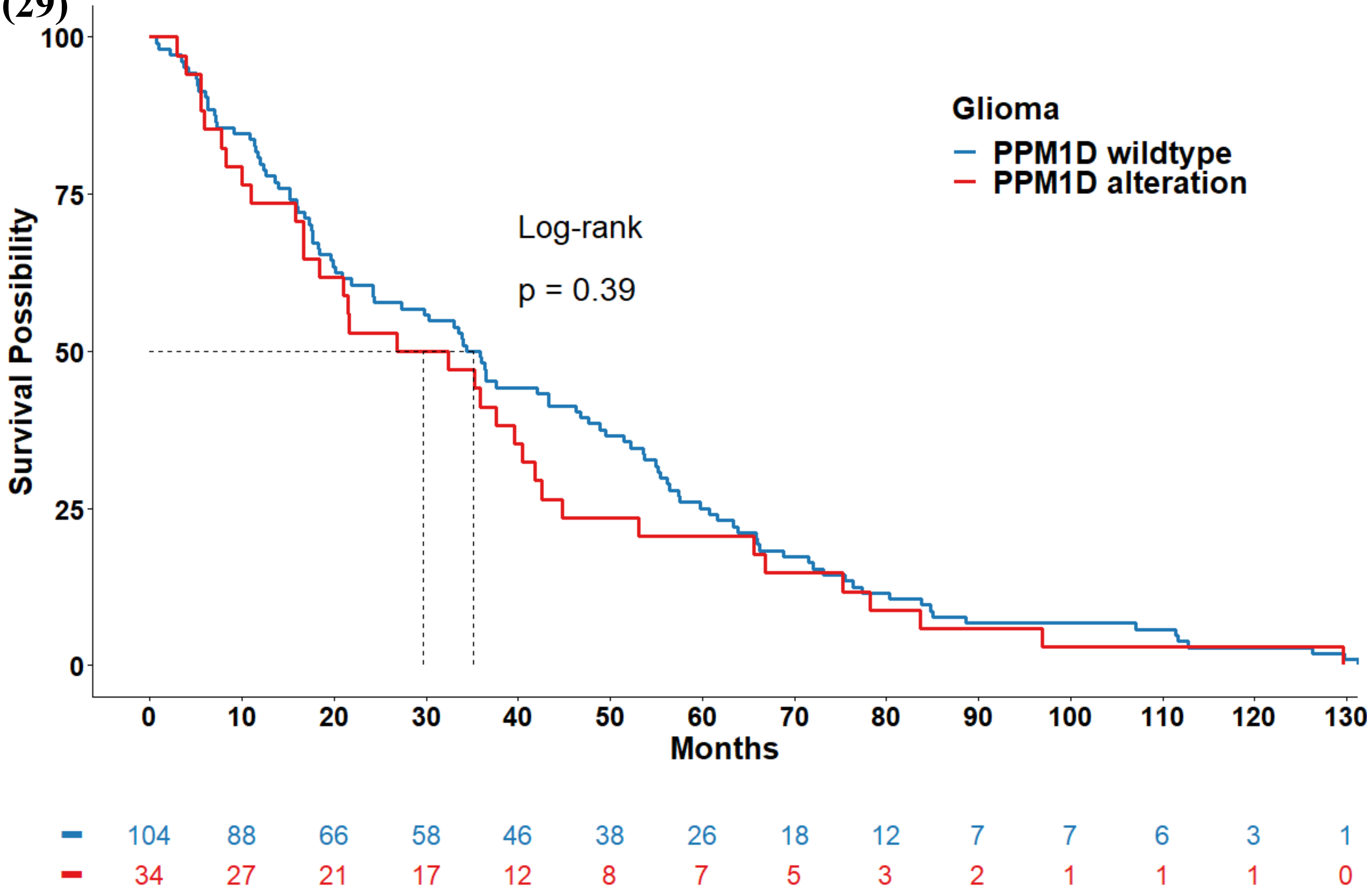
Glioma
PPM1D wildtype
PPM1D alteration
Log-rank
p = 0.39
Survival Possibility
100
75
50
25
0
0 10 20 30 40 50 60 70 80 90 100 110 120 130
Months
104 88 66 58 46 38 26 18 12 7 7 6 3 1
34 27 21 17 12 8 7 5 3 2 1 1 1 0

(30)

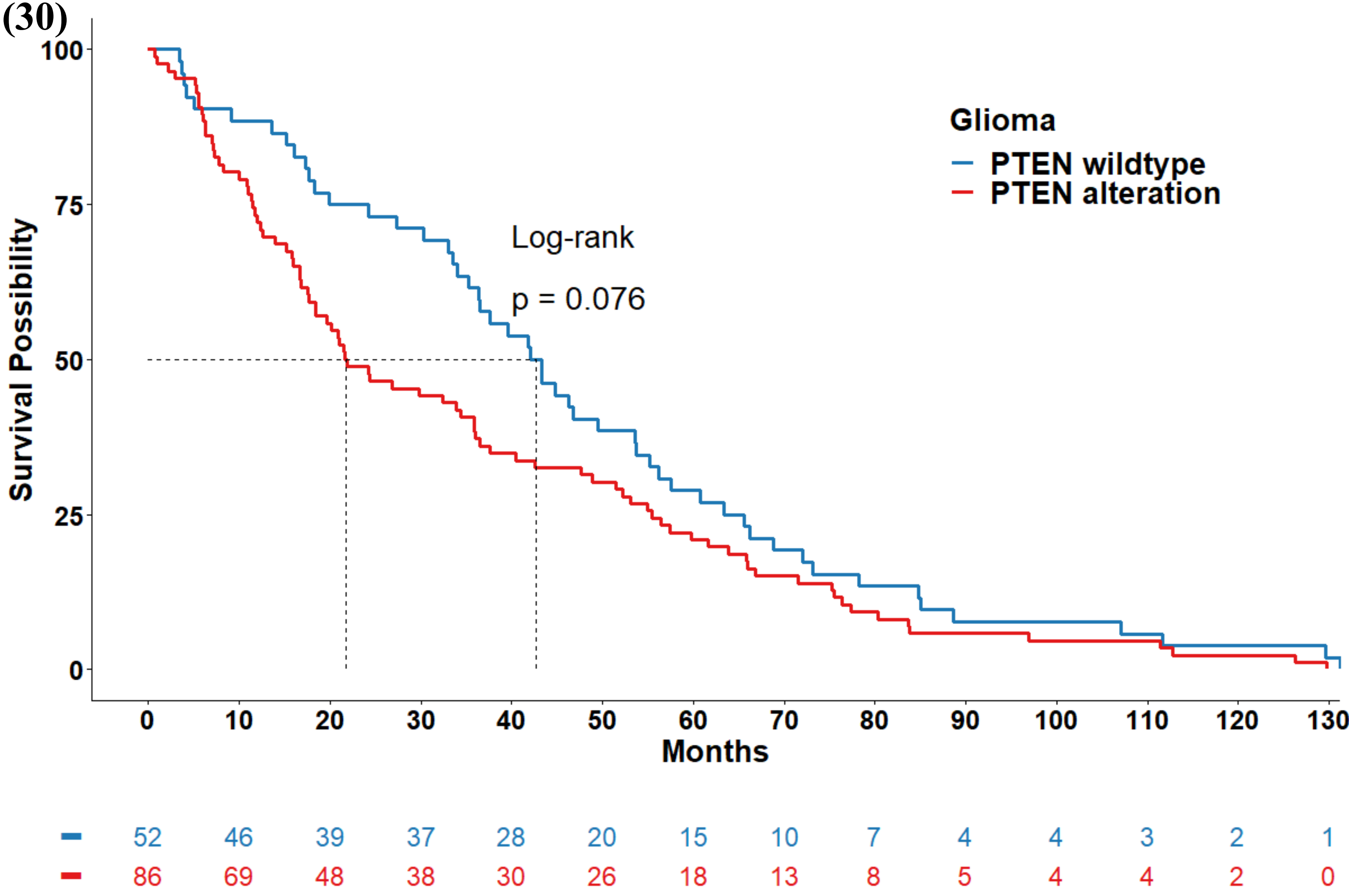
Glioma
PTEN wildtype
PTEN alteration
Log-rank
p = 0.076
Survival Possibility
100
75
50
25
0
0
10
20
30
40
50
60
70
80
90
100
110
120
130
Months
52 46 39 37 28 20 15 10 7 4 4 3 2 1
86 69 48 38 30 26 18 13 8 5 4 4 2 0

(31)

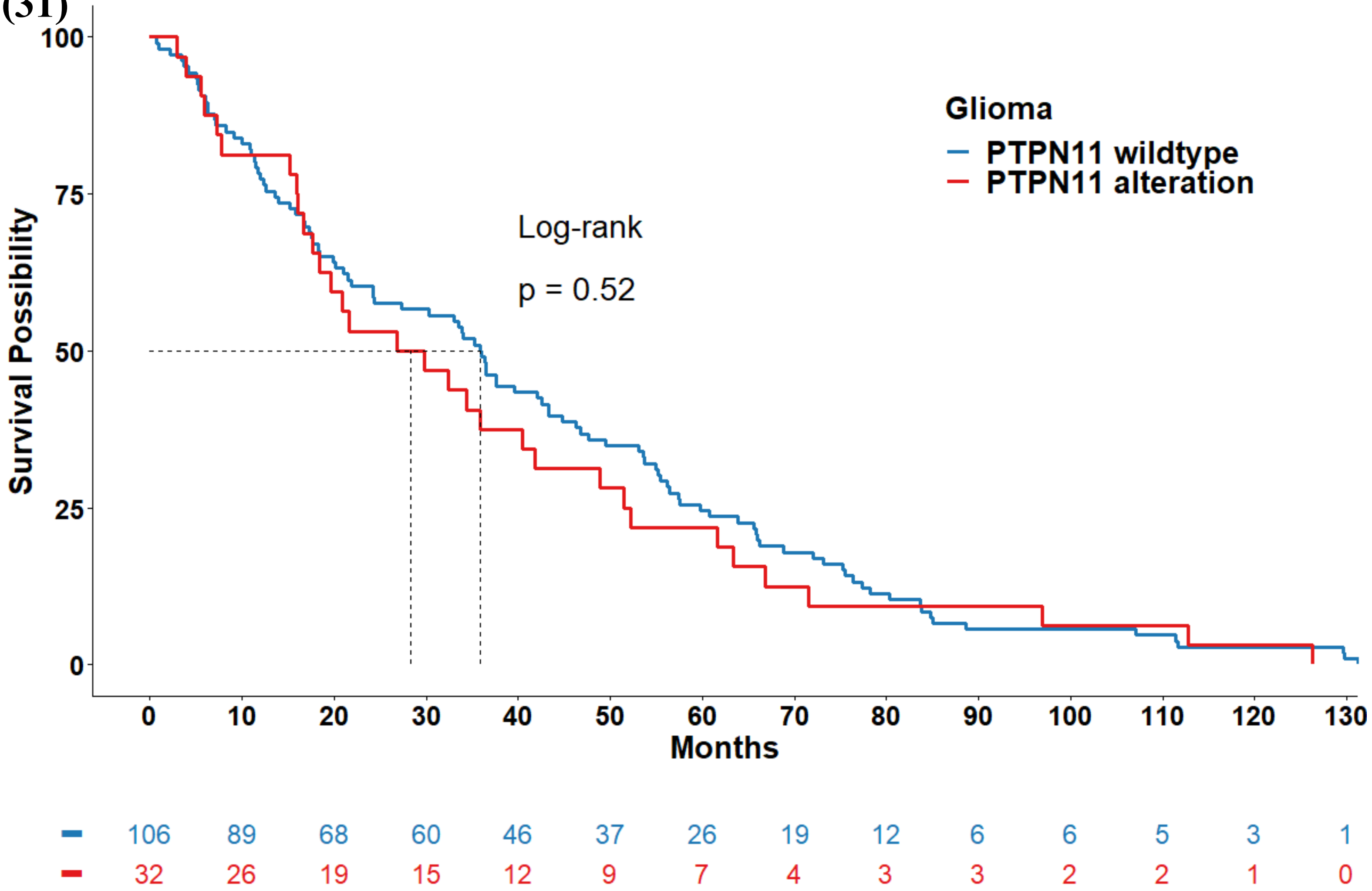
Glioma
PTPN11 wildtype
PTPN11 alteration
Log-rank
p = 0.52
Survival Possibility
100
75
50
25
0
0
10
20
30
40
50
60
70
80
90
100
110
120
130
Months
106 89 68 60 46 37 26 19 12 6 6 5 3 1
32 26 19 15 12 9 7 4 3 3 2 2 1 0

(32)

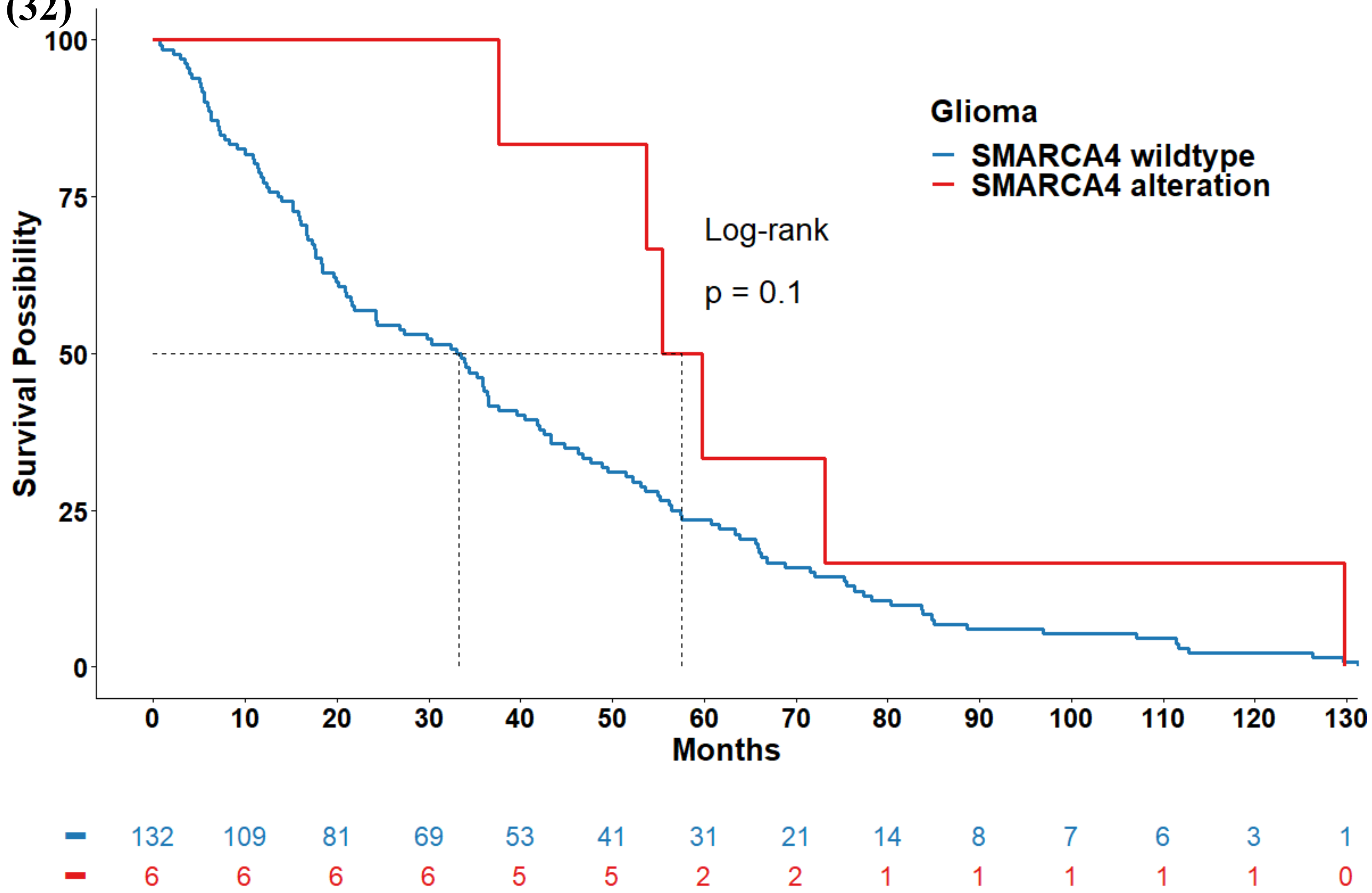
100
75
50
25
0
Survival Possibility
Glioma
SMARCA4 wildtype
SMARCA4 alteration
Log-rank
p = 0.1
0
10
20
30
40
50
60
70
80
90
100
110
120
130
Months
132 109 81 69 53 41 31 21 14 8 7 6 3 1
6 6 6 6 5 5 2 2 1 1 1 1 1 0

(33)

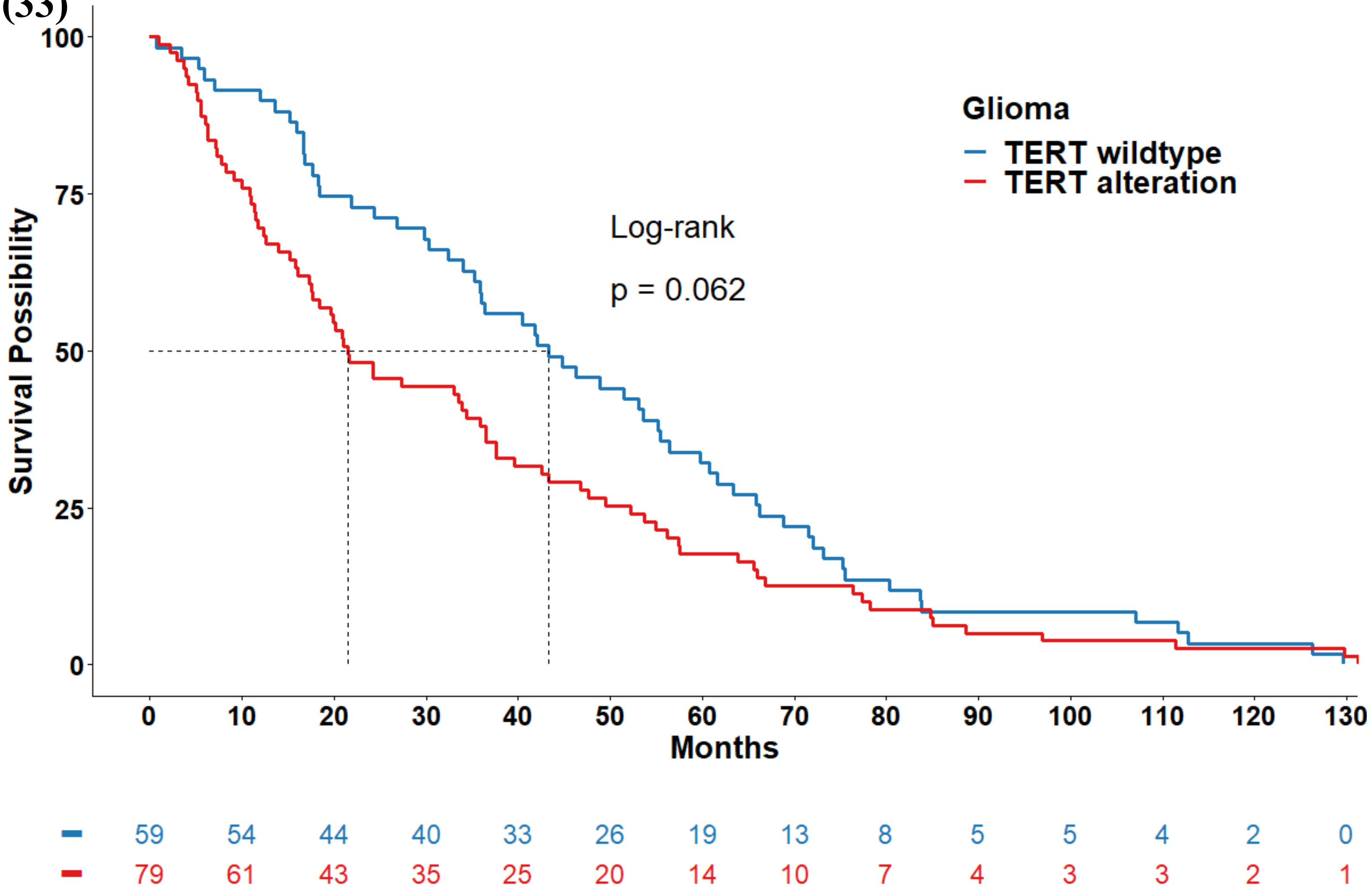
Glioma
TERT wildtype
TERT alteration
Log-rank
p = 0.062
Survival Possibility
100
75
50
25
0
0 10 20 30 40 50 60 70 80 90 100 110 120 130
Months
59 54 44 40 33 26 19 13 8 5 5 4 2 0
79 61 43 35 25 20 14 10 7 4 3 3 2 1

(34)

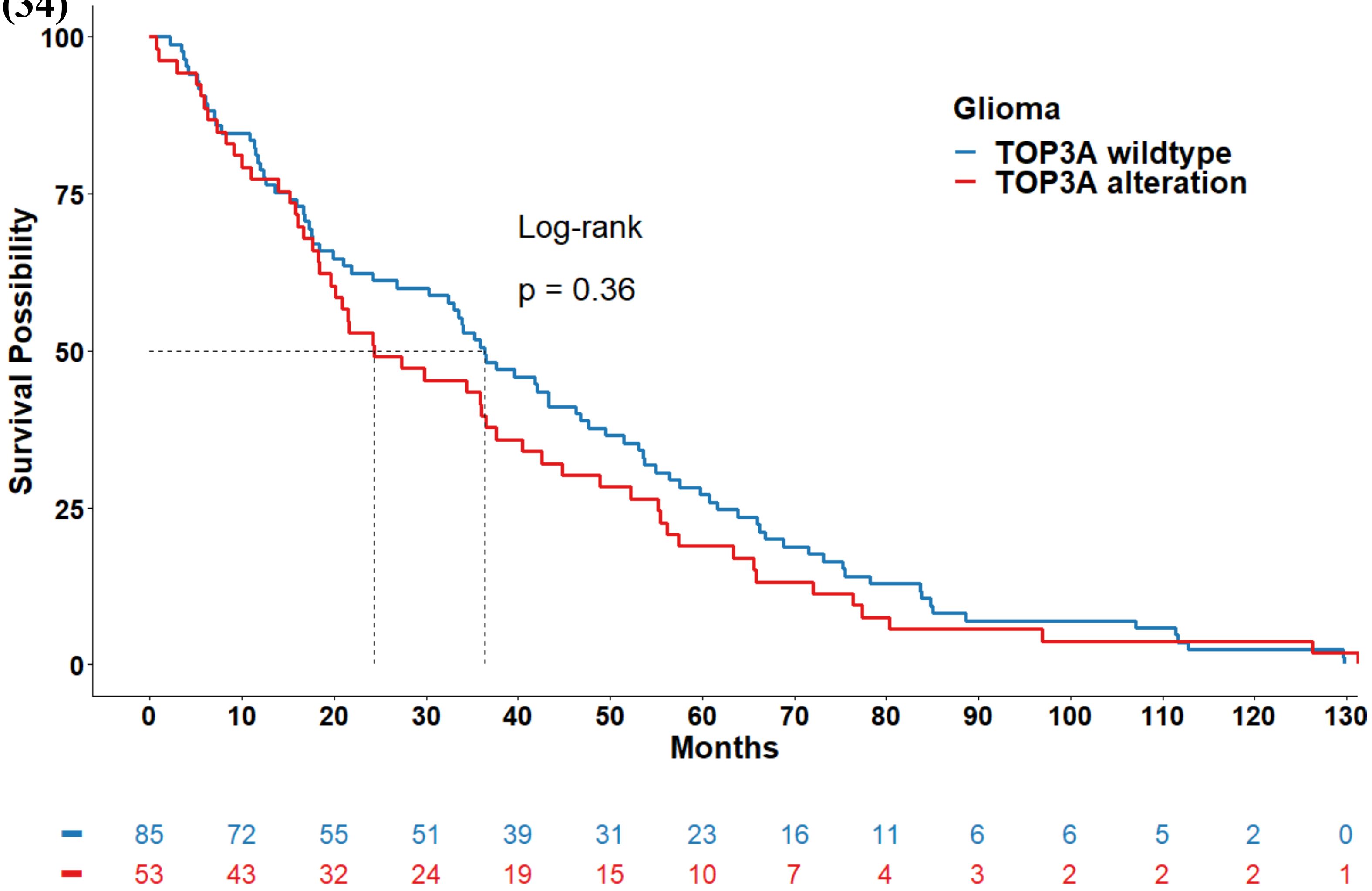
100
75
50
25
0
Survival Possibility
0
10
20
30
40
50
60
70
80
90
100
110
120
130
Months
Glioma
TOP3A wildtype
TOP3A alteration
Log-rank
p = 0.36
85 72 55 51 39 31 23 16 11 6 6 5 2 0
53 43 32 24 19 15 10 7 4 3 2 2 2 1

(35)

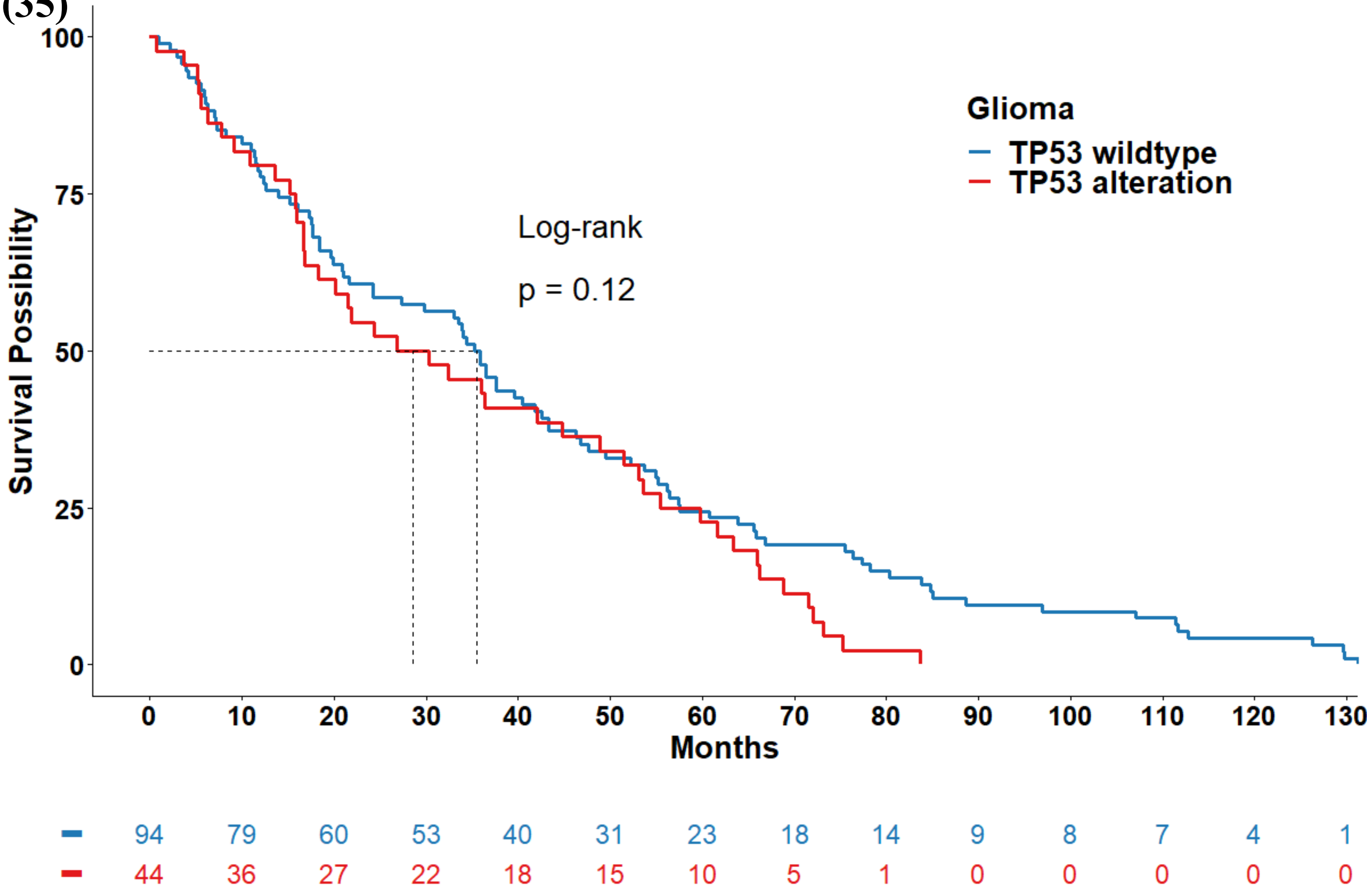
Survival Possibility
100
75
50
25
0
Glioma
TP53 wildtype
TP53 alteration
Log-rank
p = 0.12
0
10
20
30
40
50
60
70
80
90
100
110
120
130
Months
94 79 60 53 40 31 23 18 14 9 8 7 4 1
44 36 27 22 18 15 10 5 1 0 0 0 0 0

(36)

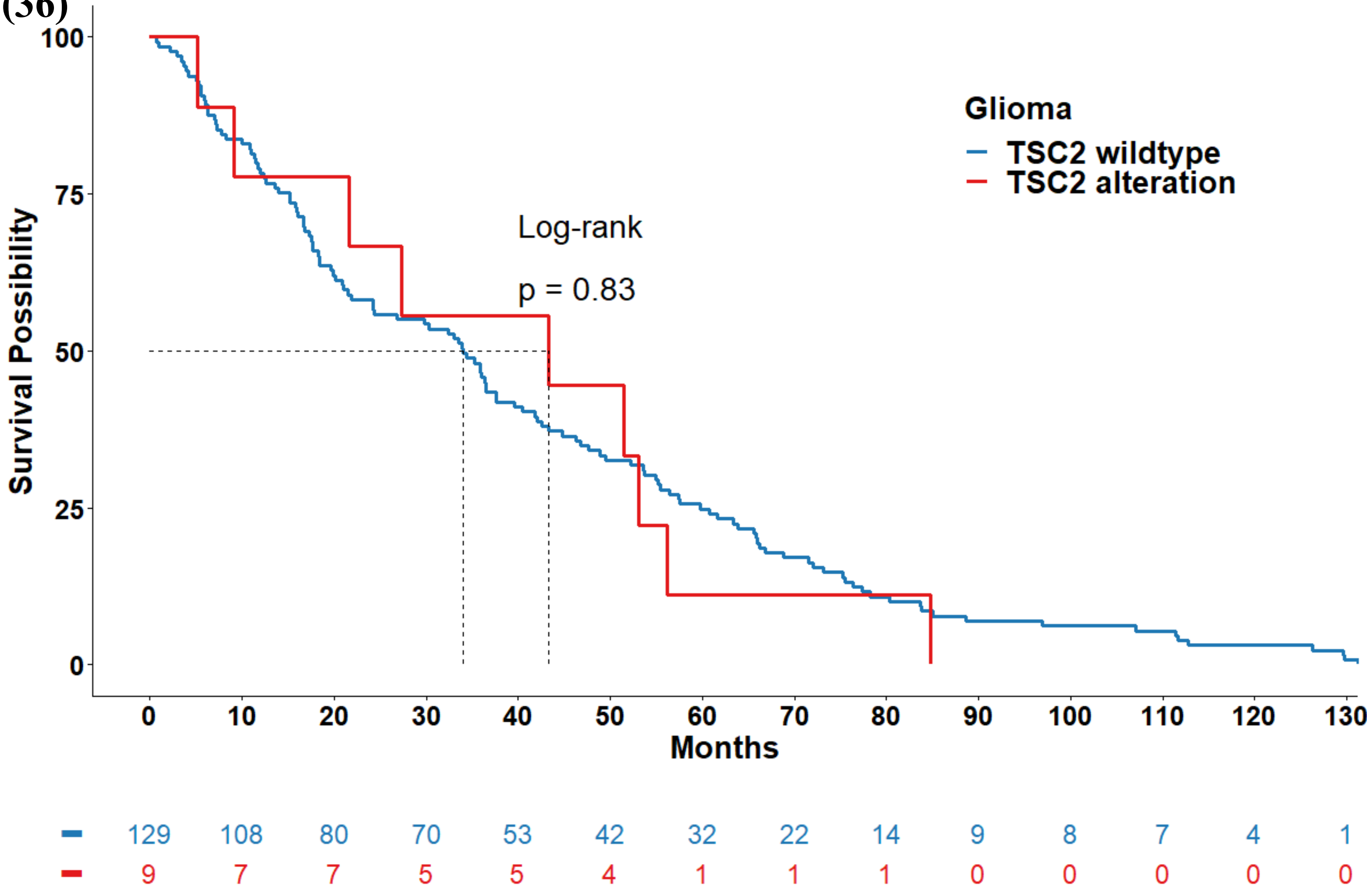

Survival Possibility
100
75
50
25
0
Glioma
TSC2 wildtype
TSC2 alteration
Log-rank
p = 0.83
0 10 20 30 40 50 60 70 80 90 100 110 120 130
Months
129 108 80 70 53 42 32 22 14 9 8 7 4 1
9 7 7 5 5 4 1 1 1 0 0 0 0 0
