# Supplementary material for: Clinical updates on gliomas and implications of the 5th edition of the WHO classification of central nervous system tumors
Source: Front Oncol. 2023 Mar 14;13:1131642. doi: 10.3389/fonc.2023.1131642 (PMC10043404; doi:10.3389/fonc.2023.1131642)
Supplement: Supplementary file 2 [file DataSheet_2.pdf]

Supplement Figure 2. Kaplan–Meier curves showing no significant effects of certain genetic alterations on the overall survival of patients with astrocytoma (1-31). Since the numbers of patients divided into two groups (e.g. BRAF wildtype/BRAF alteration) using different parameters varied, we only enrolled the parameters using which the number of patients in either group was above 3. Parameters used in this section were shown as follows.

(1) ATRX ... 1
(2) BRAF ... 2
(3) CDK4 ... 3
(4) CDK6 ... 4
(5) CDKN2A ... 5
(6) CDKN2B ... 6
(7) CIC ... 7
(8) EGFR ... 8
(9) FGFR1 ... 9
(10) FGFR2 ... 10
(11) FGFR3 ... 11
(12) KMT5B ... 12
(13) KRAS ... 13
(14) MYB ... 14
(15) MYBL1 ... 15
(16) MYC ... 16
(17) MYCN ... 17
(18) NOTCH1 ... 18
(19) NTRK2 ... 19
(20) NTRK3 ... 20
(21) PDGFRA ... 21
(22) PIK3CA ... 22
(23) PIK3R1 ... 23
(24) PPM1D ... 24
(25) PTEN ... 25
(26) PTPN11 ... 26
(27) RB1 ... 27
(28) SMARCA4 ... 28
(29) TOP3A ... 29
(30) TP53 ... 30
(31) chr19q ... 31

(1)

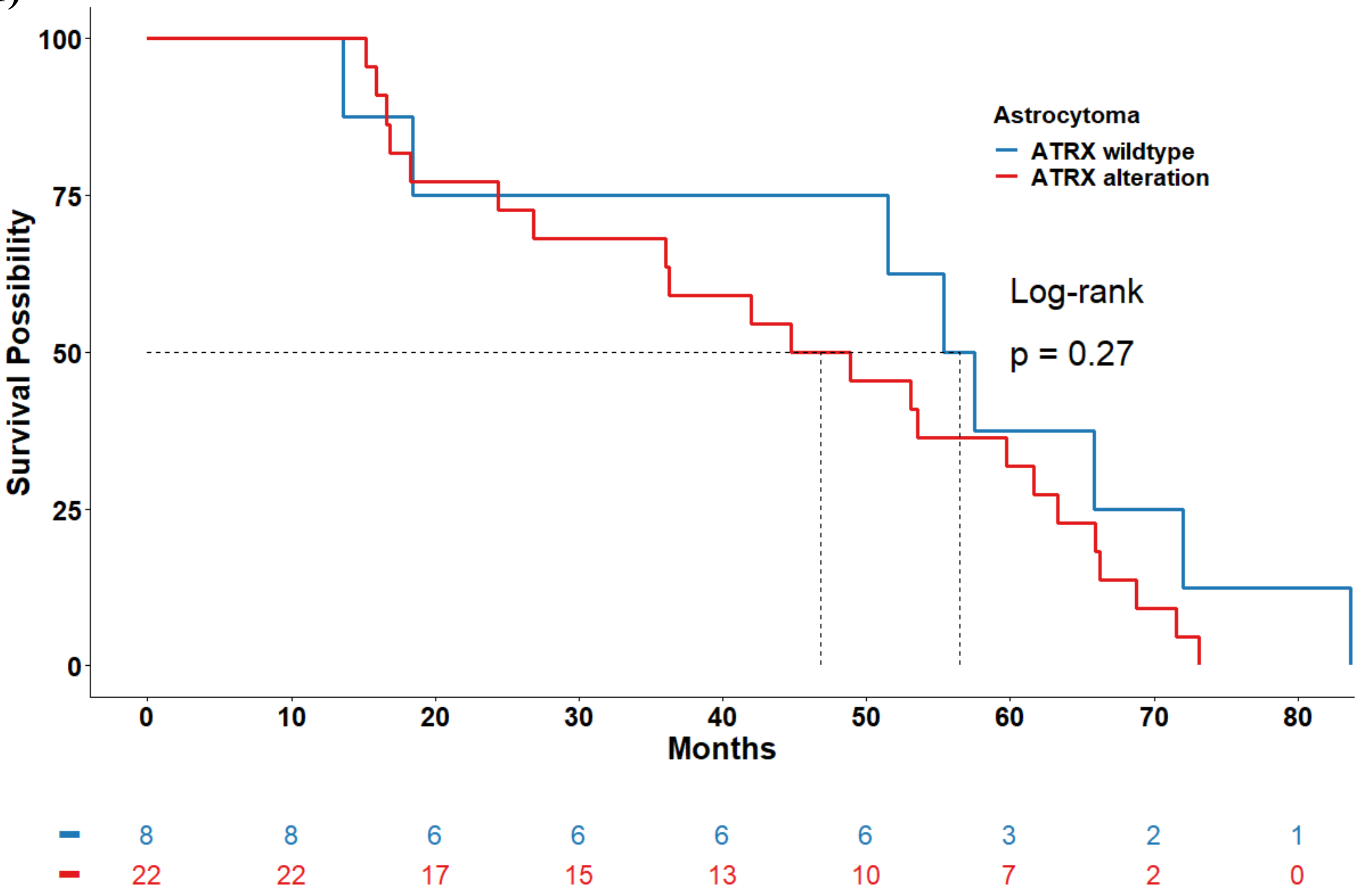

Astrocytoma
ATRX wildtype
ATRX alteration
Log-rank
p = 0.27
Survival Possibility
100
75
50
25
0
0
10
20
30
40
50
60
70
80
Months
8 8 6 6 6 6 3 2 1
22 22 17 15 13 10 7 2 0

(2)

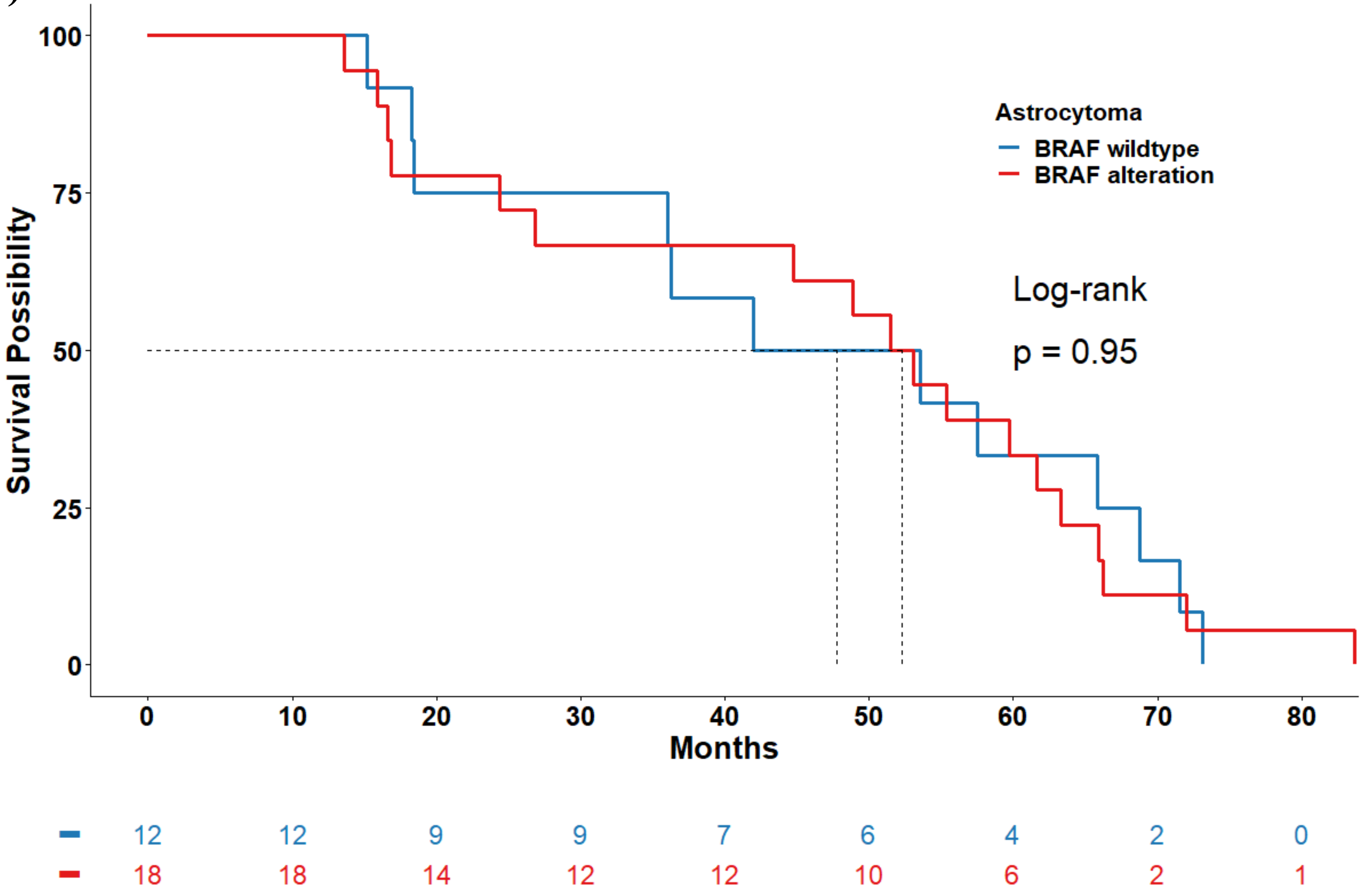

Survival Possibility
100
75
50
25
0
0
10
20
30
40
50
60
70
80
Months
Astrocytoma
BRAF wildtype
BRAF alteration
Log-rank
p = 0.95
12 12 9 9 7 6 4 2 0
18 18 14 12 12 10 6 2 1

(3)

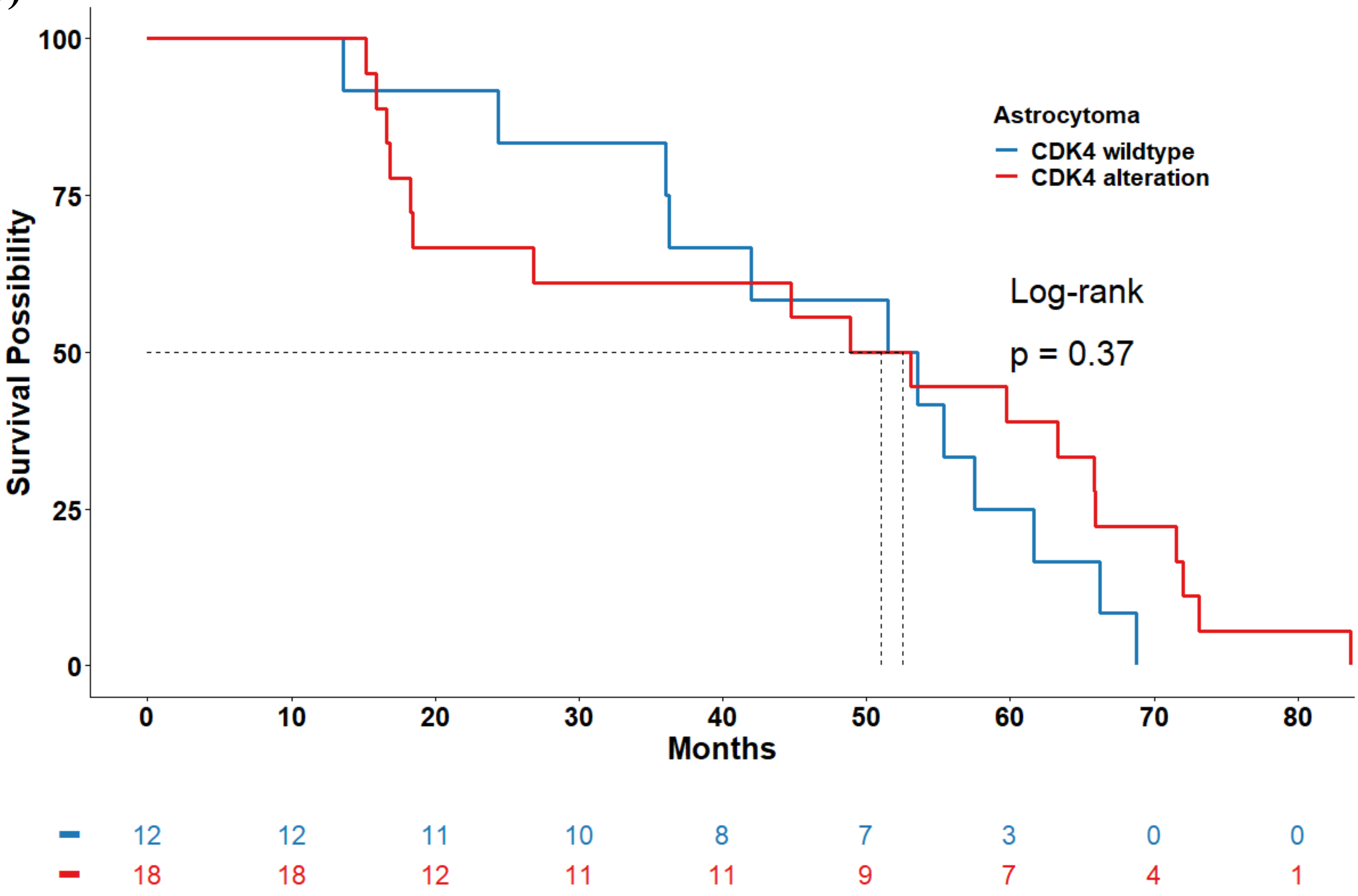
Survival Possibility
100
75
50
25
0
0
10
20
30
40
50
60
70
80
Months
Astrocytoma
CDK4 wildtype
CDK4 alteration
Log-rank
p = 0.37
12 12 11 10 8 7 3 0 0
18 18 12 11 11 9 7 4 1

(4)

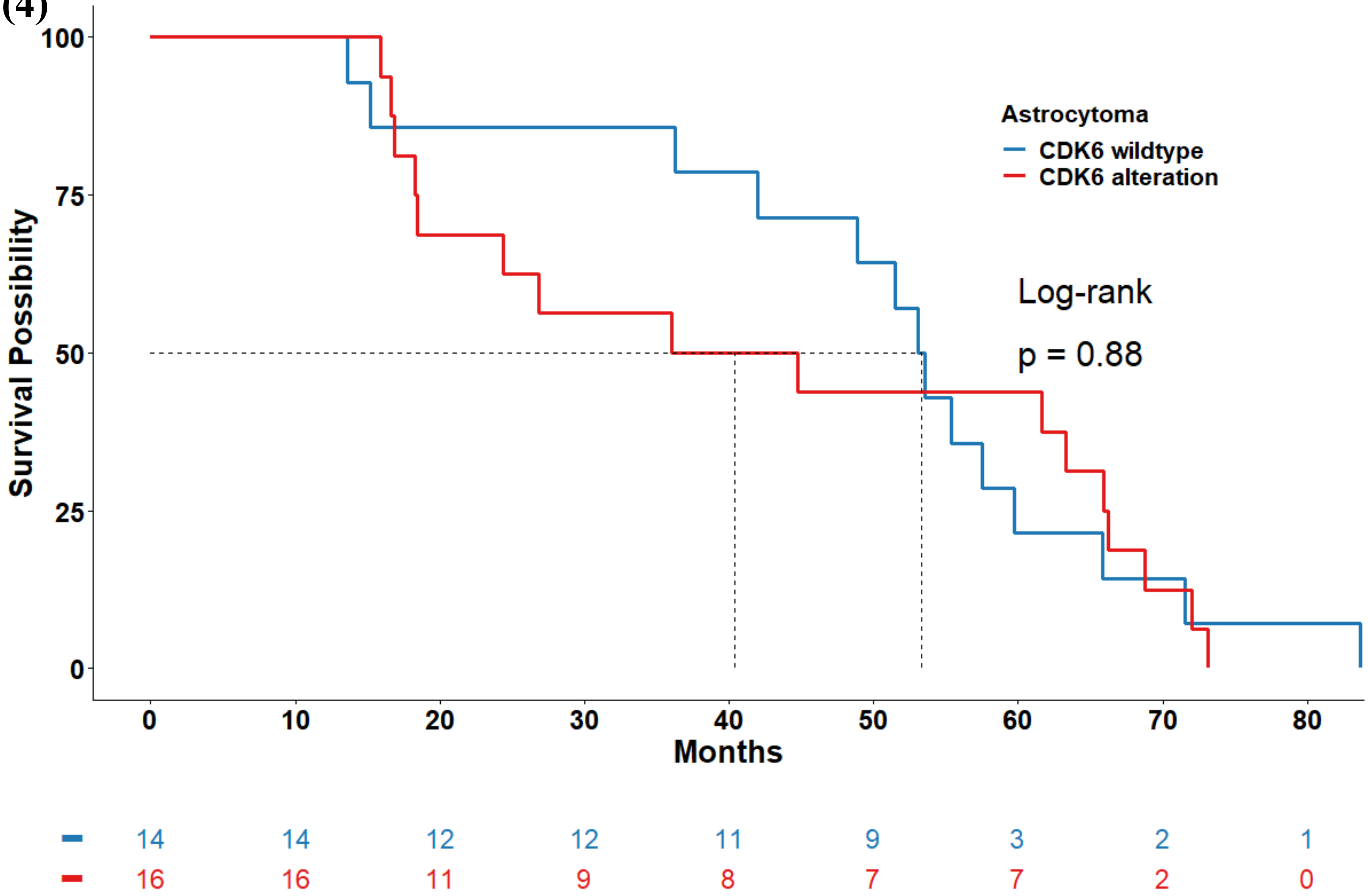
Survival Possibility
100
75
50
25
0
0
10
20
30
40
50
60
70
80
Months
Astrocytoma
CDK6 wildtype
CDK6 alteration
Log-rank
p = 0.88
14 14 12 12 11 9 3 2 1
16 16 11 9 8 7 7 2 0

(5)

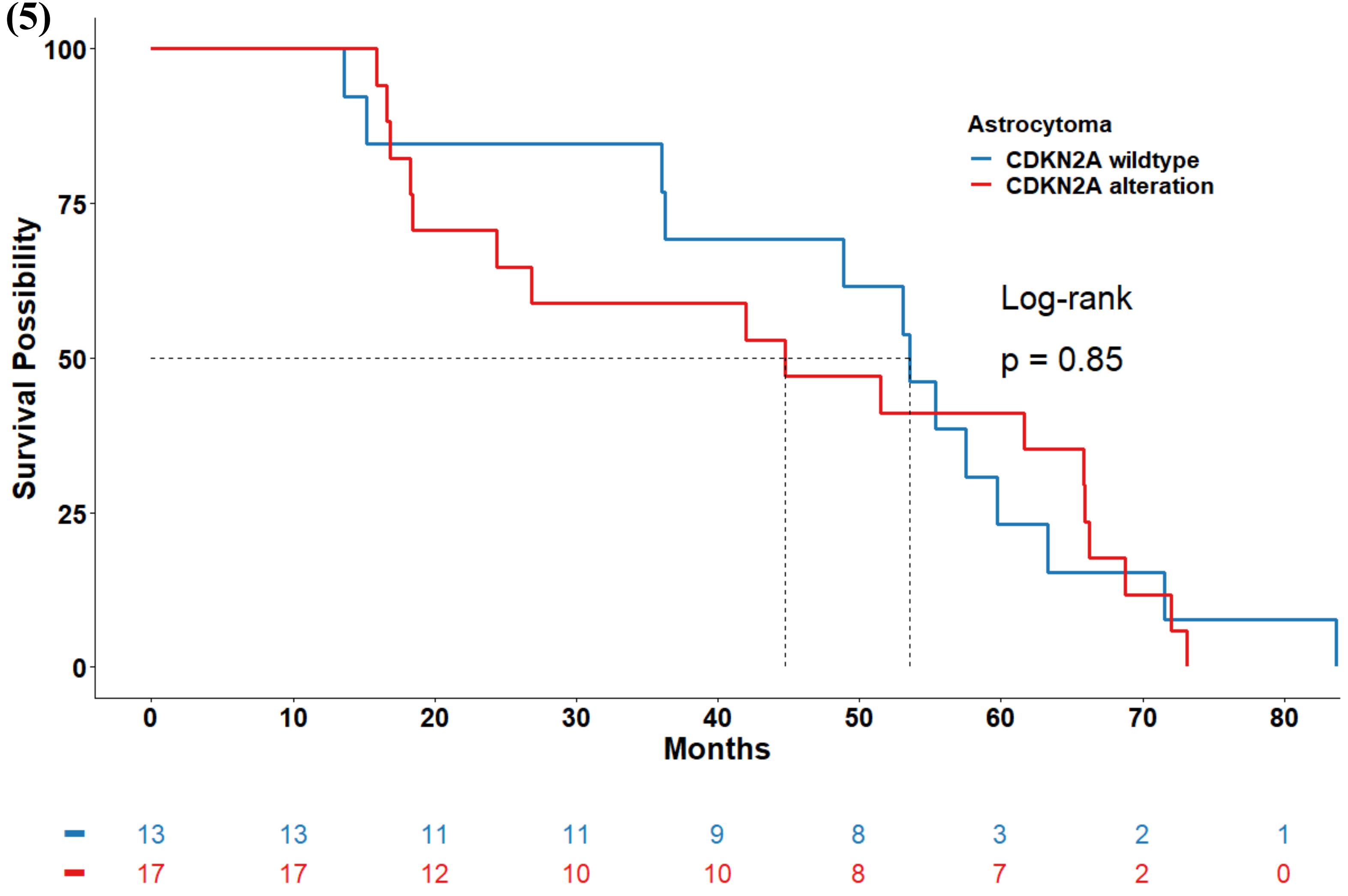
Astrocytoma
CDKN2A wildtype
CDKN2A alteration
Log-rank
p = 0.85
Survival Possibility
100
75
50
25
0
Months
0 10 20 30 40 50 60 70 80
13 13 11 11 9 8 3 2 1
17 17 12 10 10 8 7 2 0

(6)

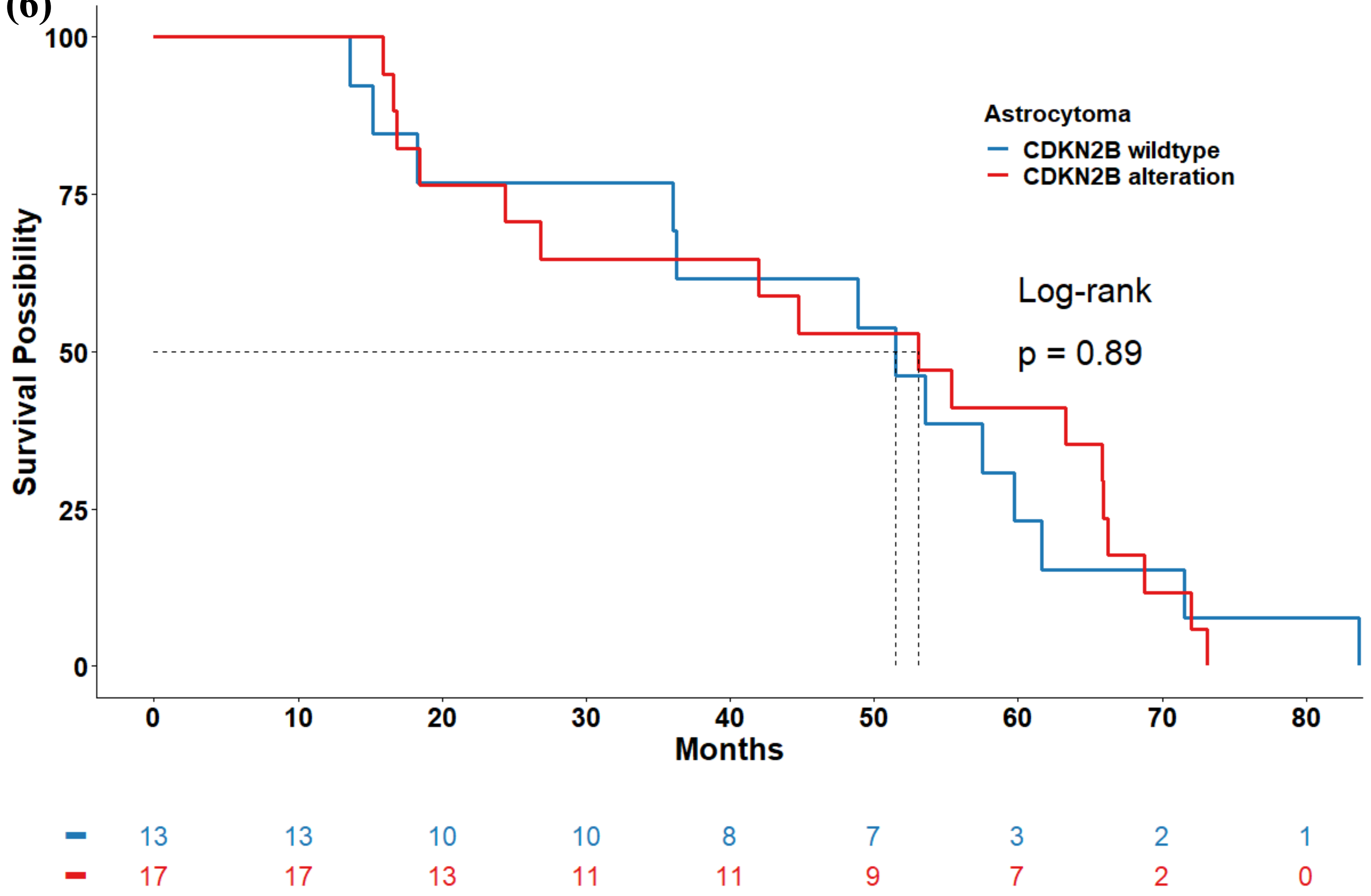

100
75
50
25
0
Survival Possibility
0
10
20
30
40
50
60
70
80
Months
Astrocytoma
CDKN2B wildtype
CDKN2B alteration
Log-rank
p = 0.89
13 13 10 10 8 7 3 2 1
17 17 13 11 11 9 7 2 0

(7)

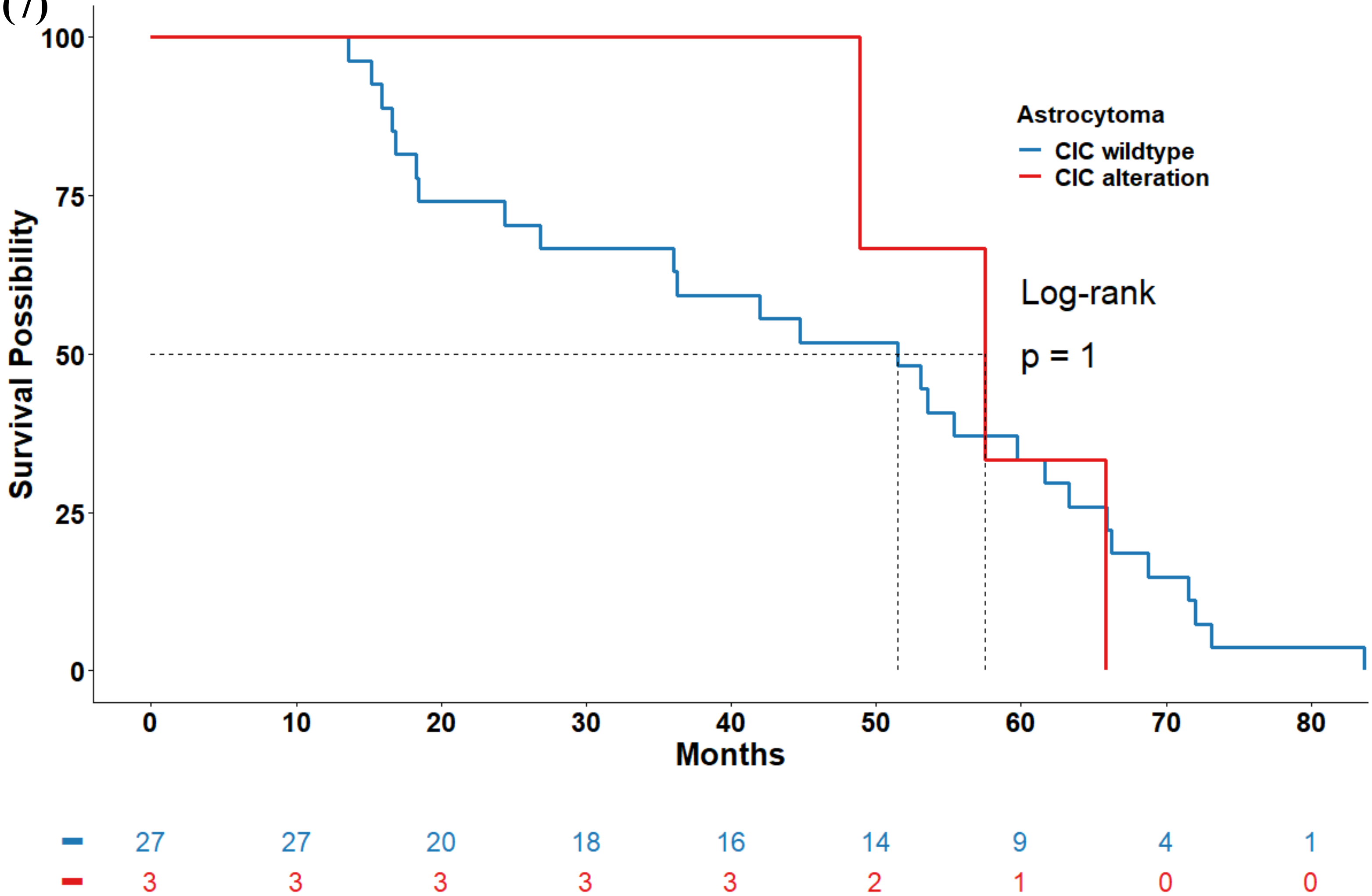
Survival Possibility
100
75
50
25
0
Astrocytoma
CIC wildtype
CIC alteration
Log-rank
p = 1
0
10
20
30
40
50
60
70
80
Months
27 27 20 18 16 14 9 4 1
3 3 3 3 3 2 1 0 0

(8)

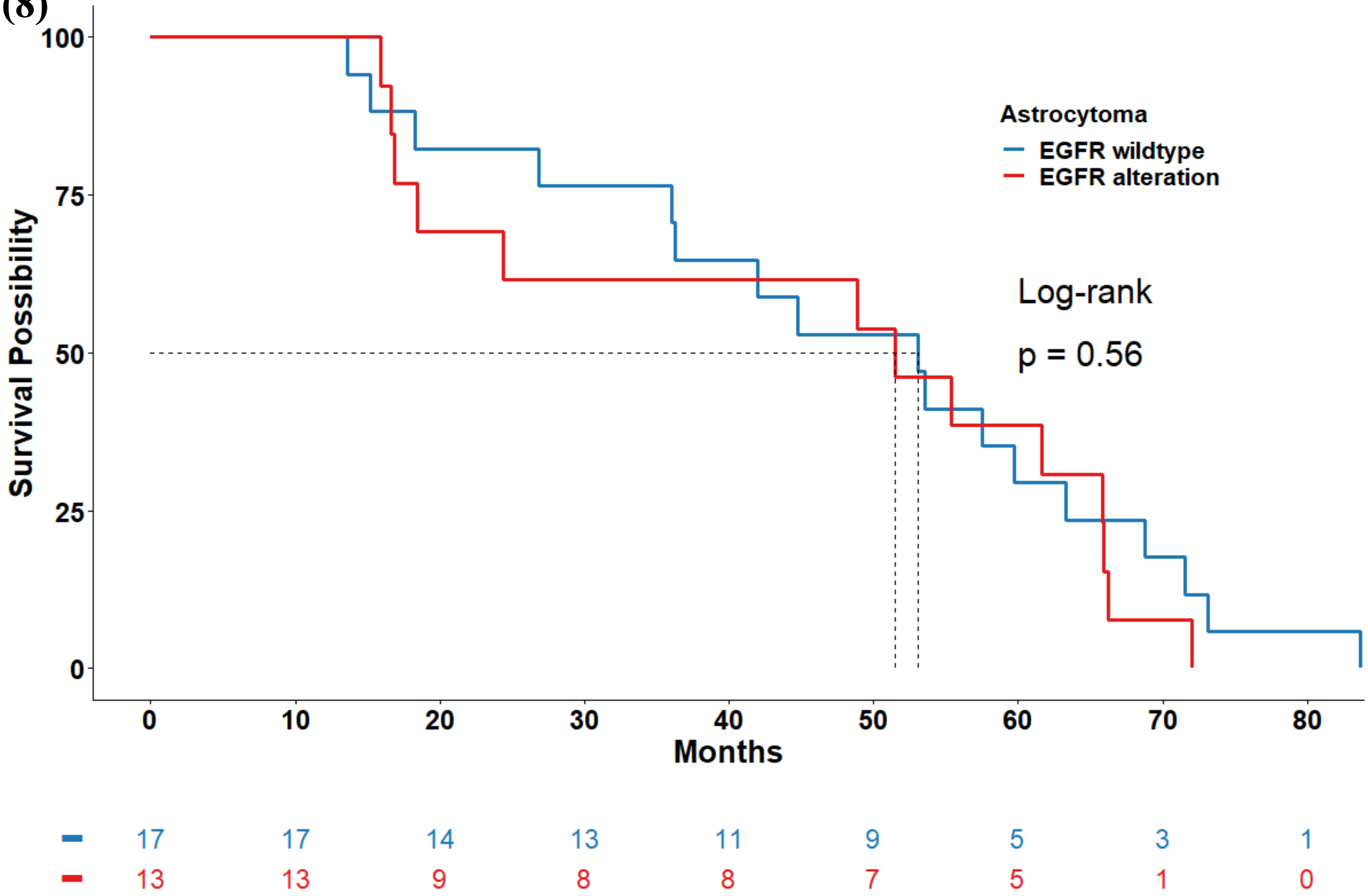

Survival Possibility
100
75
50
25
0
Astrocytoma
EGFR wildtype
EGFR alteration
Log-rank
p = 0.56
0
10
20
30
40
50
60
70
80
Months
17 17 14 13 11 9 5 3 1
13 13 9 8 8 7 5 1 0

(9)

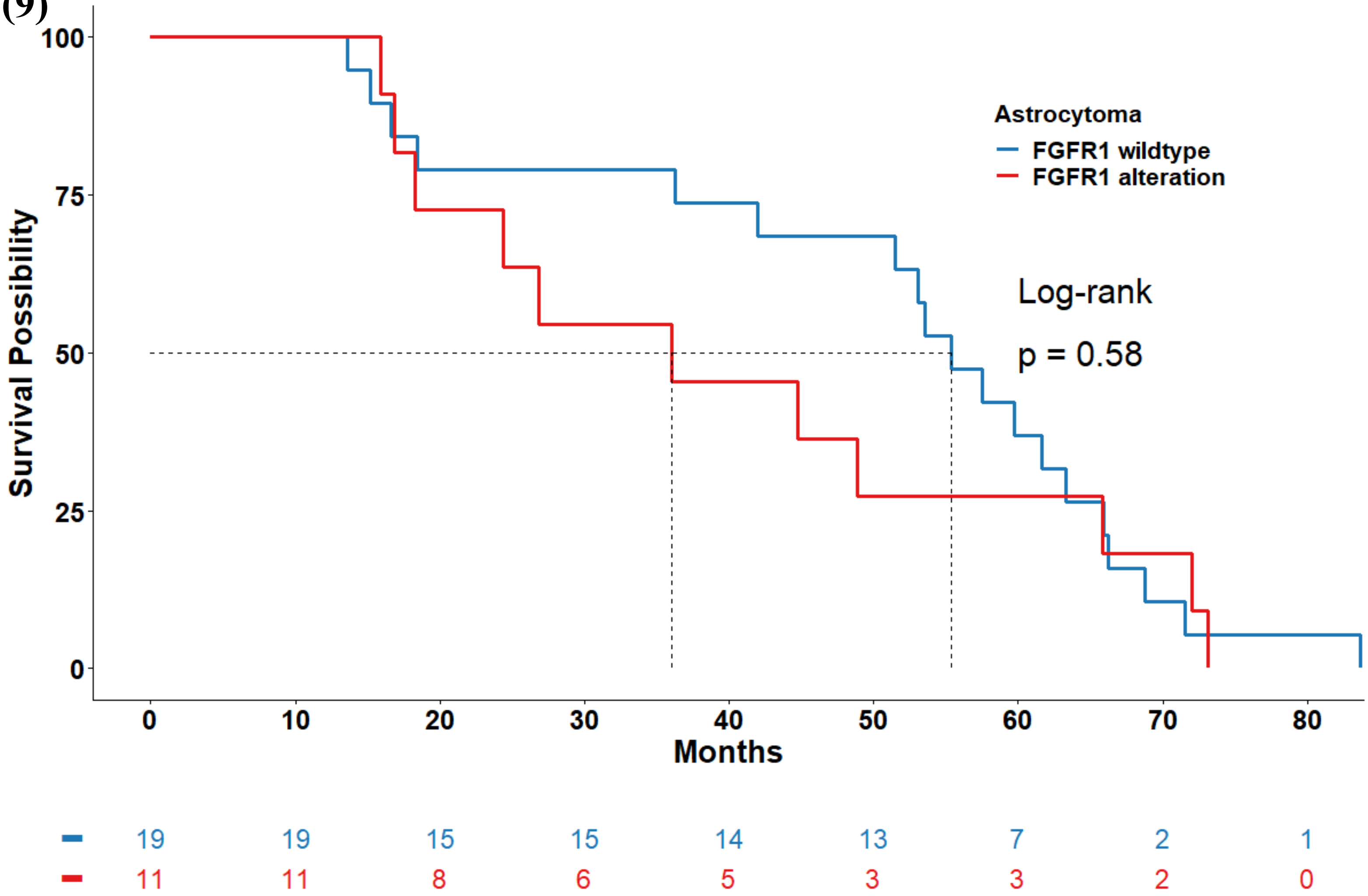

Astrocytoma
FGFR1 wildtype
FGFR1 alteration
Survival Possibility
100
75
50
25
0
Log-rank
p = 0.58
0
10
20
30
40
50
60
70
80
Months
19 19 15 15 14 13 7 2 1
11 11 8 6 5 3 3 2 0

(10)

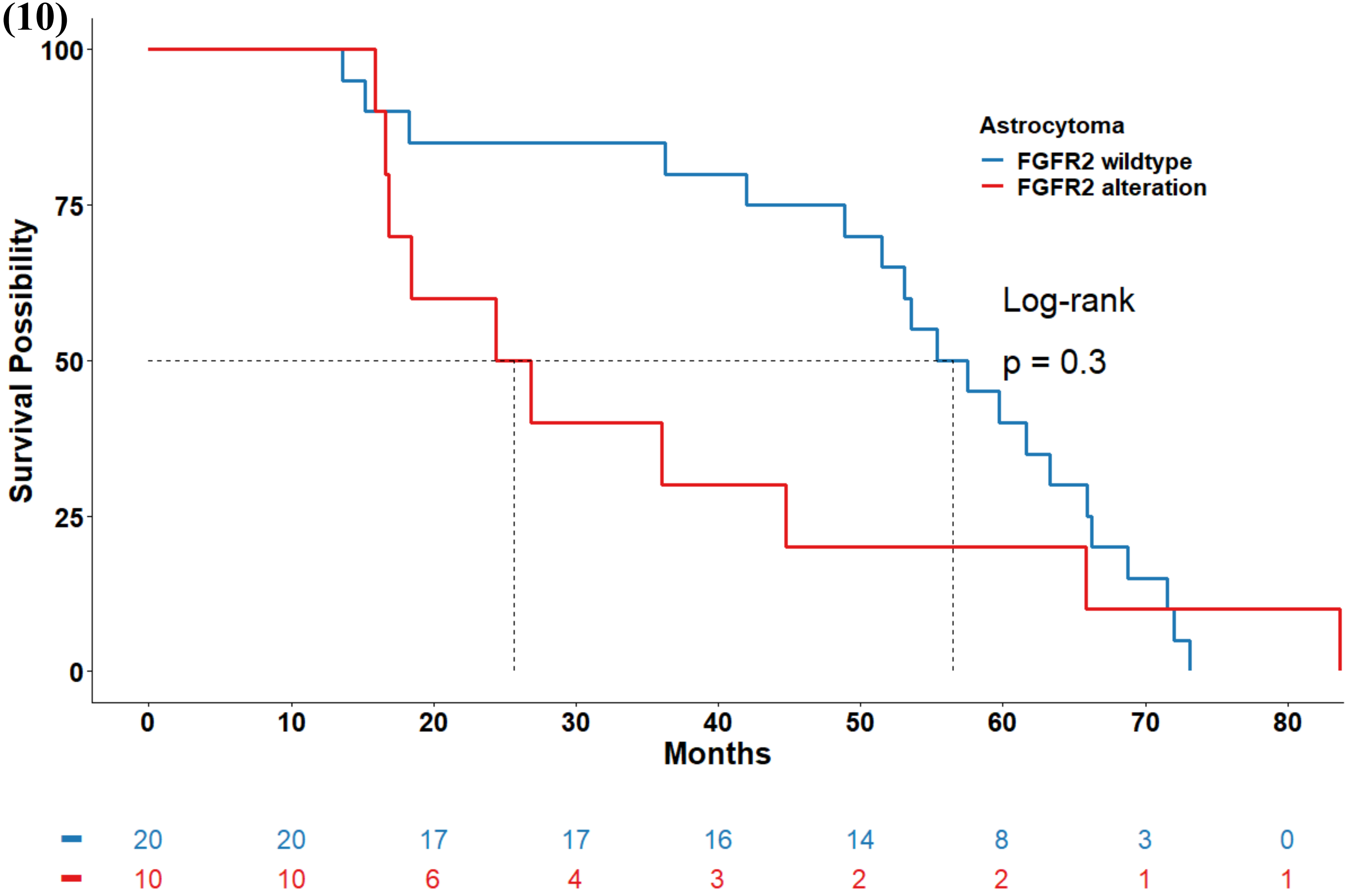

Survival Possibility
100
75
50
25
0
Astrocytoma
FGFR2 wildtype
FGFR2 alteration
Log-rank
p = 0.3
0
10
20
30
40
50
60
70
80
Months
20 20 17 17 16 14 8 3 0
10 10 6 4 3 2 2 1 1

(11)

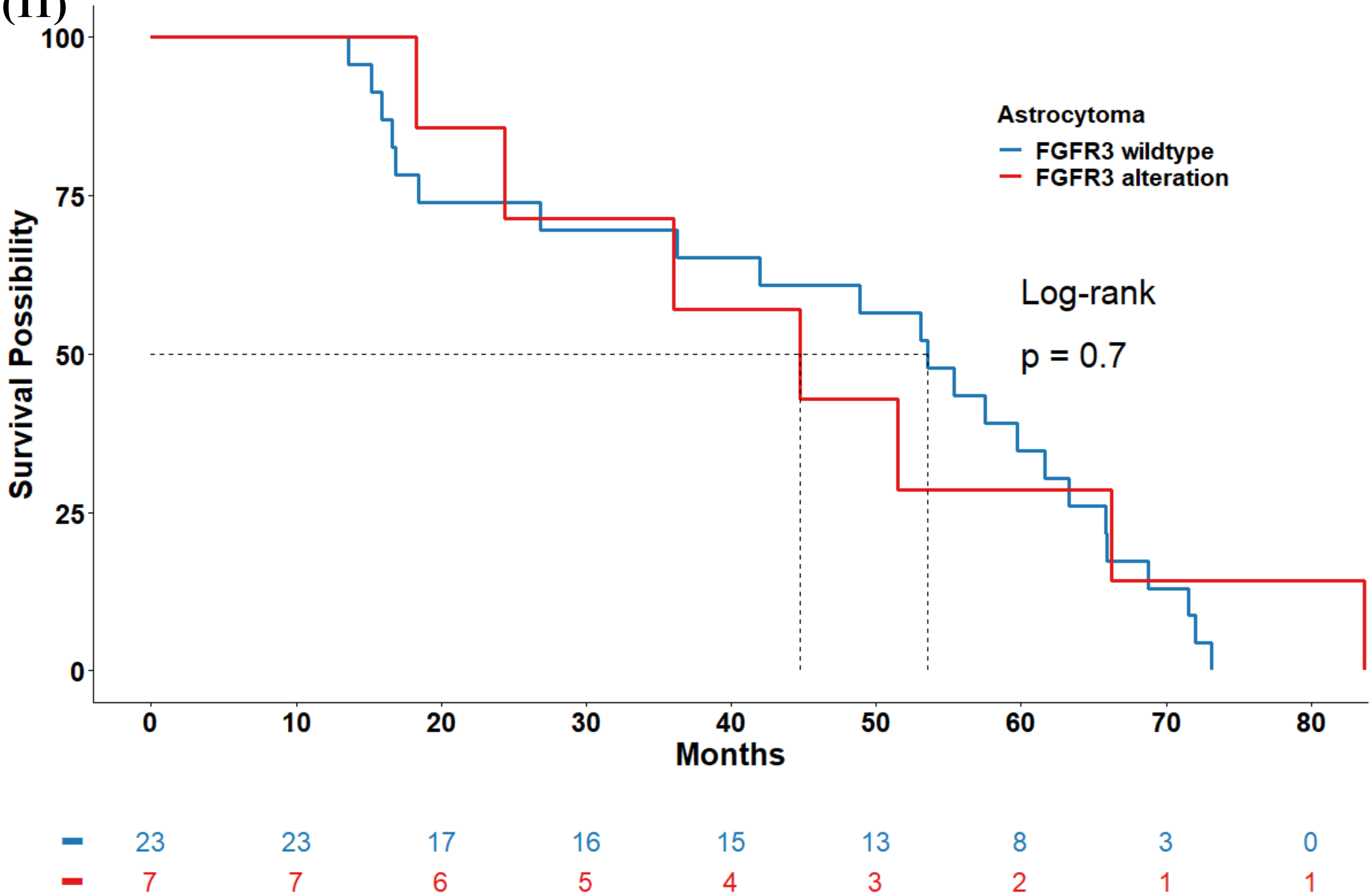

Survival Possibility
100
75
50
25
0
Astrocytoma
FGFR3 wildtype
FGFR3 alteration
Log-rank
p = 0.7
0
10
20
30
40
50
60
70
80
Months
23 23 17 16 15 13 8 3 0
7 7 6 5 4 3 2 1 1

(12)

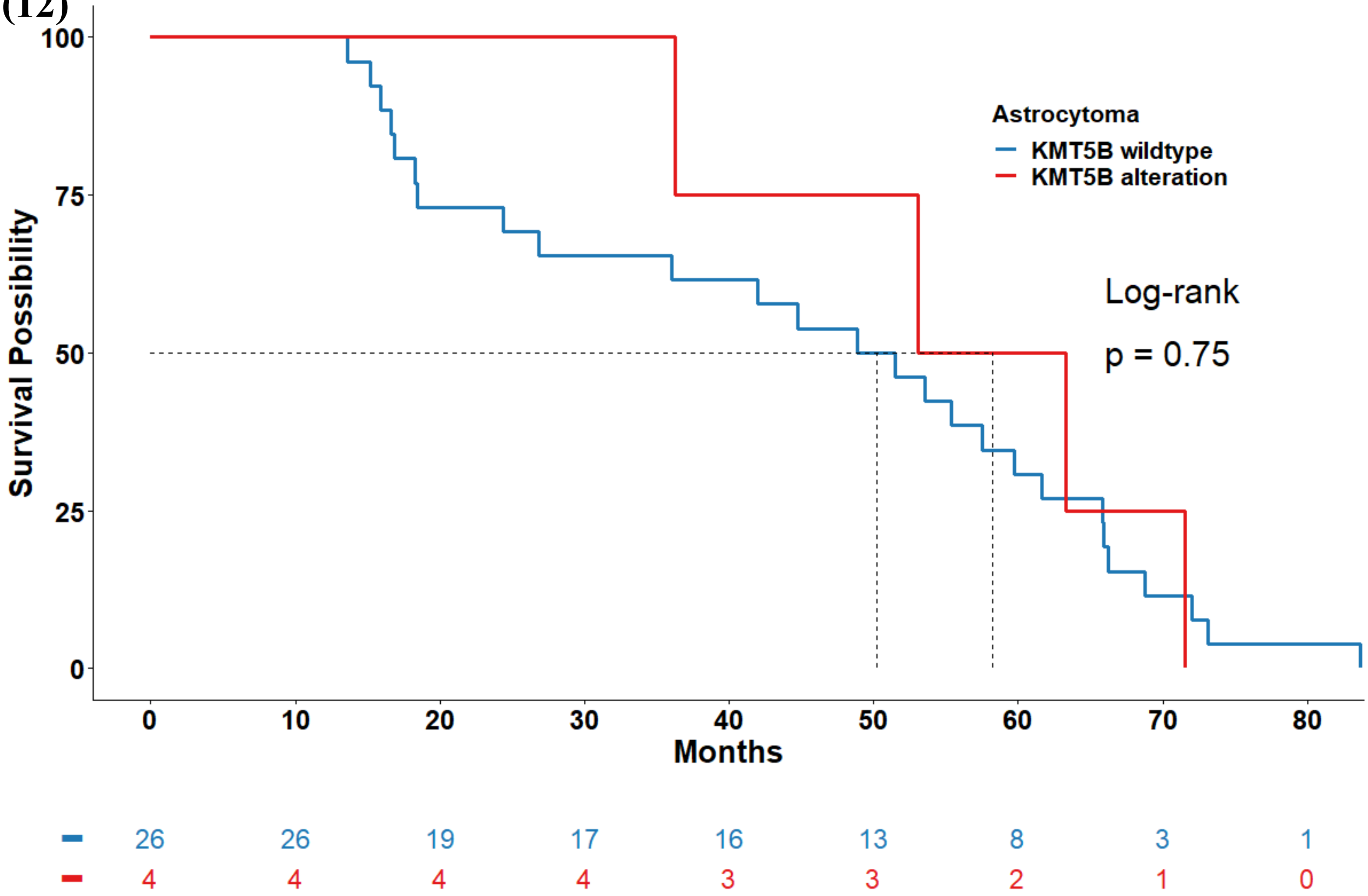
100
75
50
25
0
Survival Possibility
0
10
20
30
40
50
60
70
80
Months
Astrocytoma
KMT5B wildtype
KMT5B alteration
Log-rank
p = 0.75
26 26 19 17 16 13 8 3 1
4 4 4 4 3 3 2 1 0

(13)

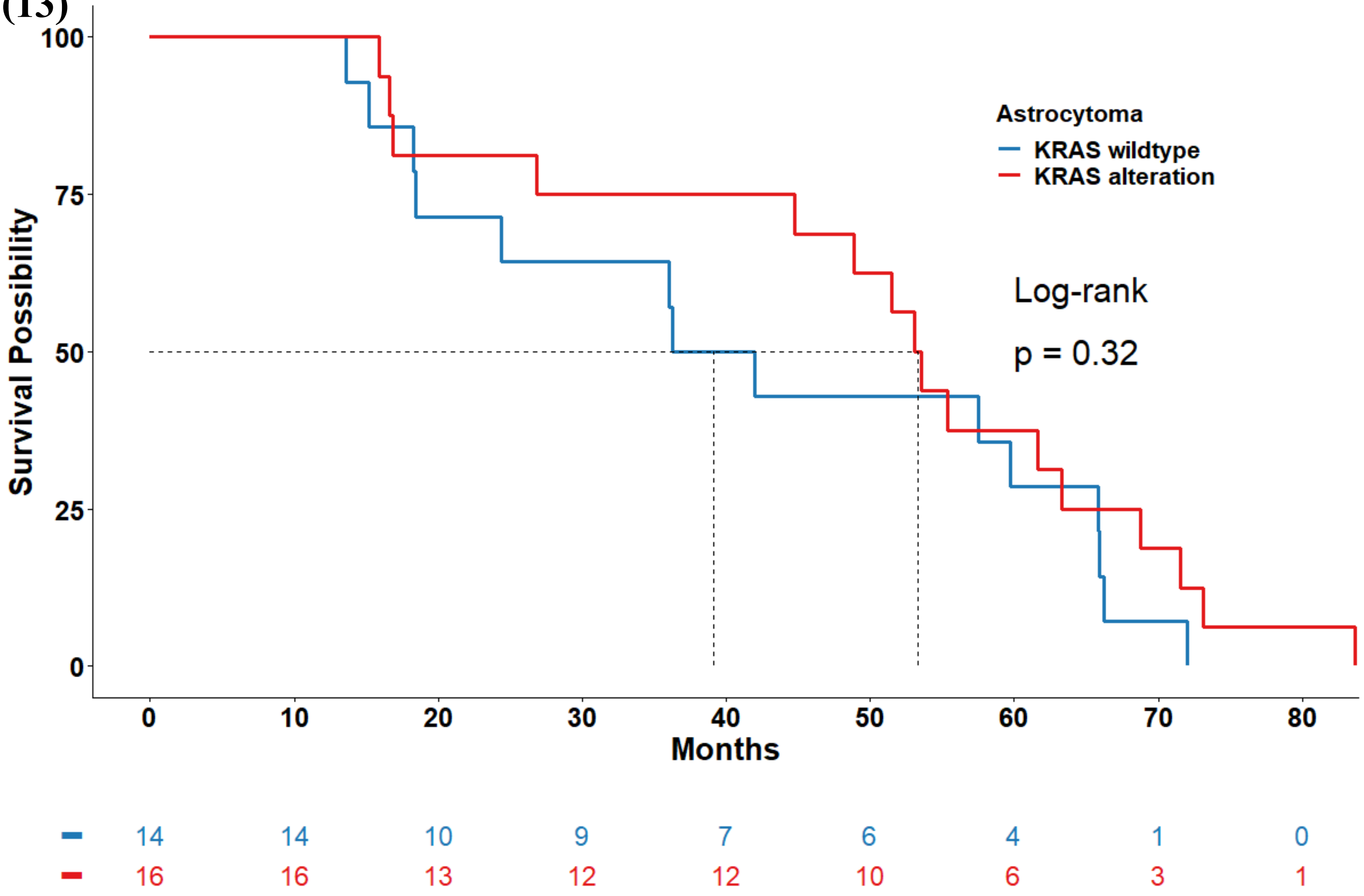

Survival Possibility
100
75
50
25
0
Astrocytoma
KRAS wildtype
KRAS alteration
Log-rank
p = 0.32
0
10
20
30
40
50
60
70
80
Months
14 14 10 9 7 6 4 1 0
16 16 13 12 12 10 6 3 1

(14)

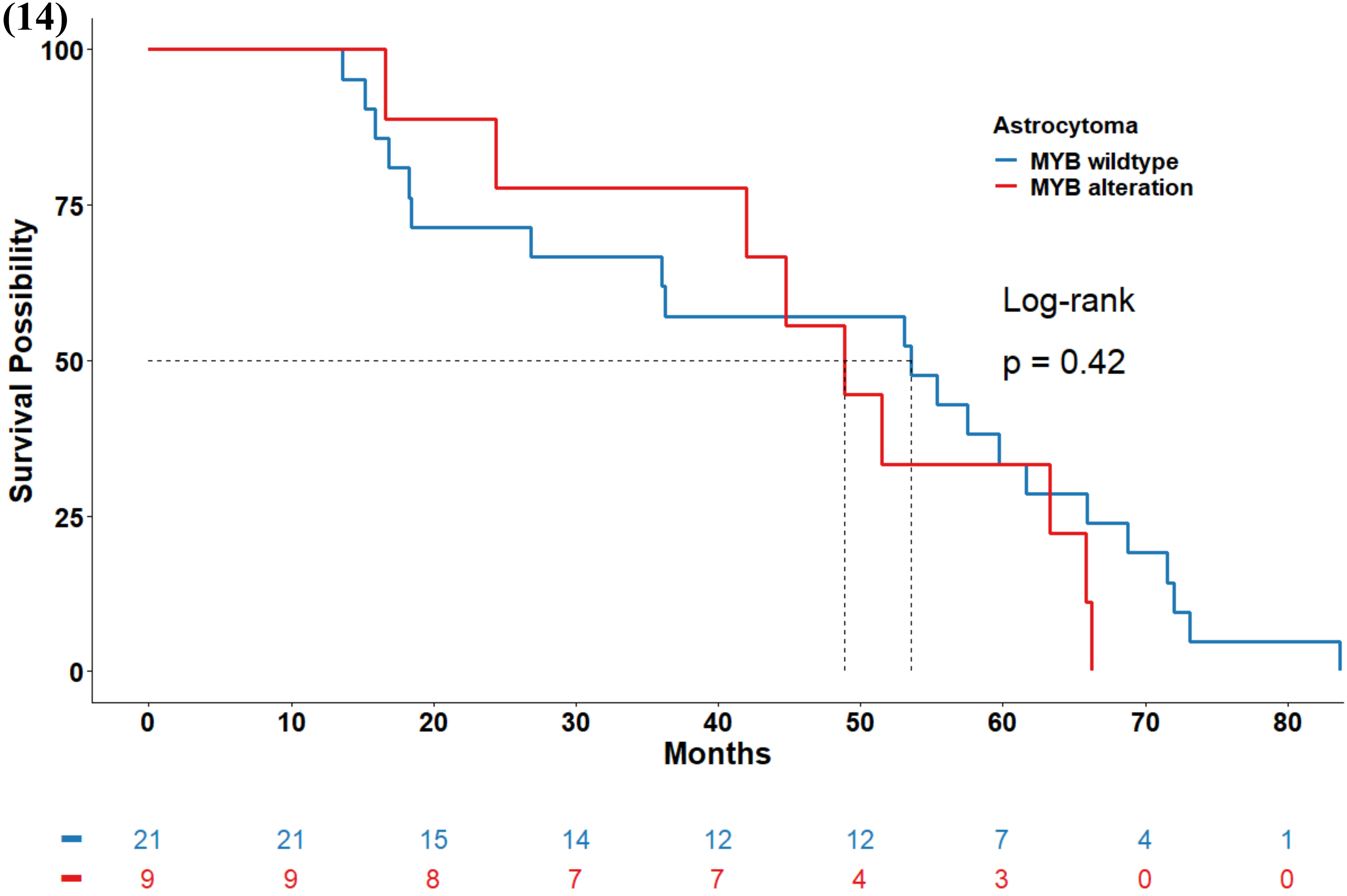
Survival Possibility
100
75
50
25
0
0
10
20
30
40
50
60
70
80
Months
Astrocytoma
MYB wildtype
MYB alteration
Log-rank
p = 0.42
21 21 15 14 12 12 7 4 1
9 9 8 7 7 4 3 0 0

(15)

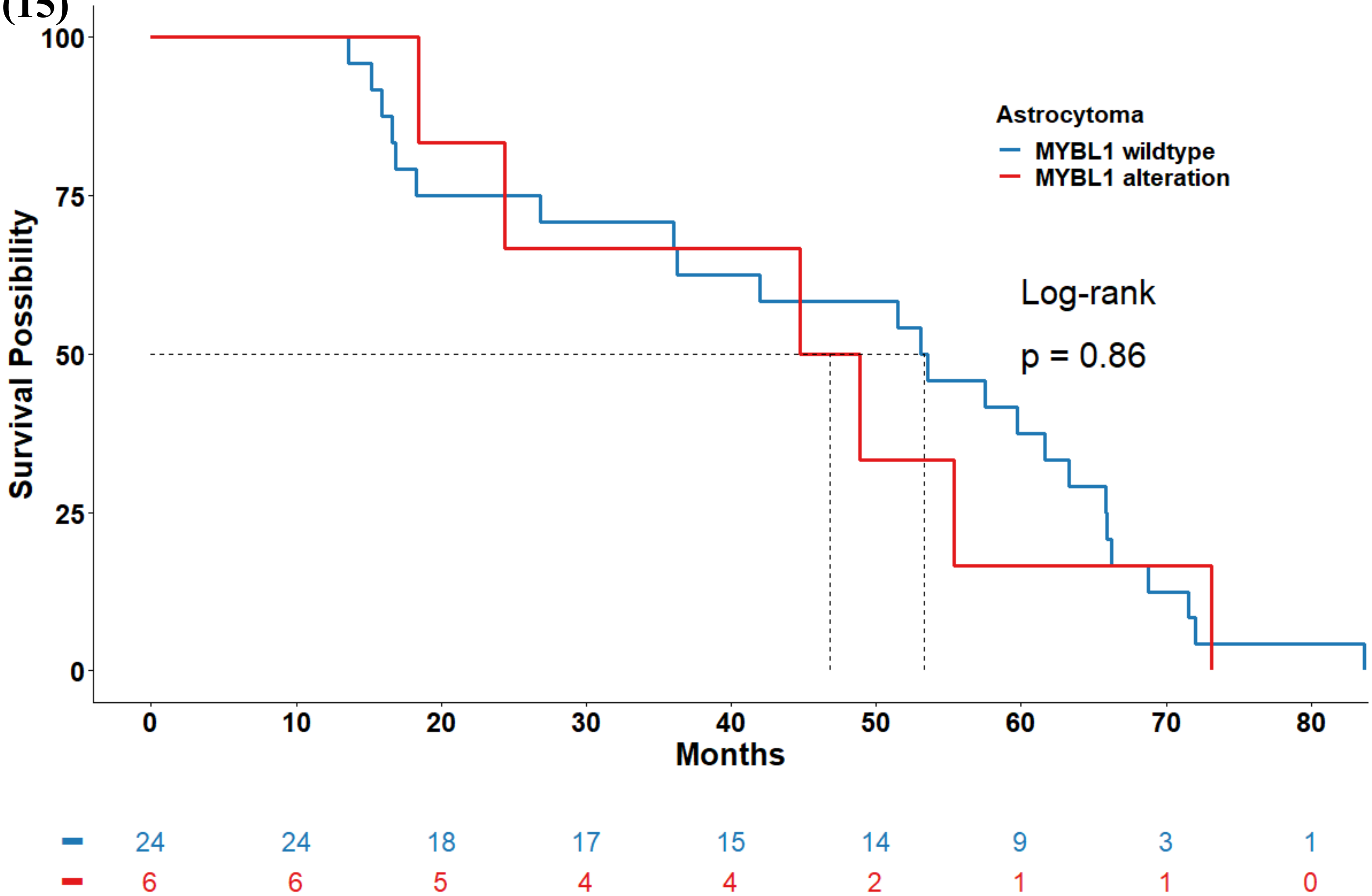
Astrocytoma
MYBL1 wildtype
MYBL1 alteration
Log-rank
p = 0.86
Survival Possibility
100
75
50
25
0
0
10
20
30
40
50
60
70
80
Months
24 24 18 17 15 14 9 3 1
6 6 5 4 4 2 1 1 0

(16)

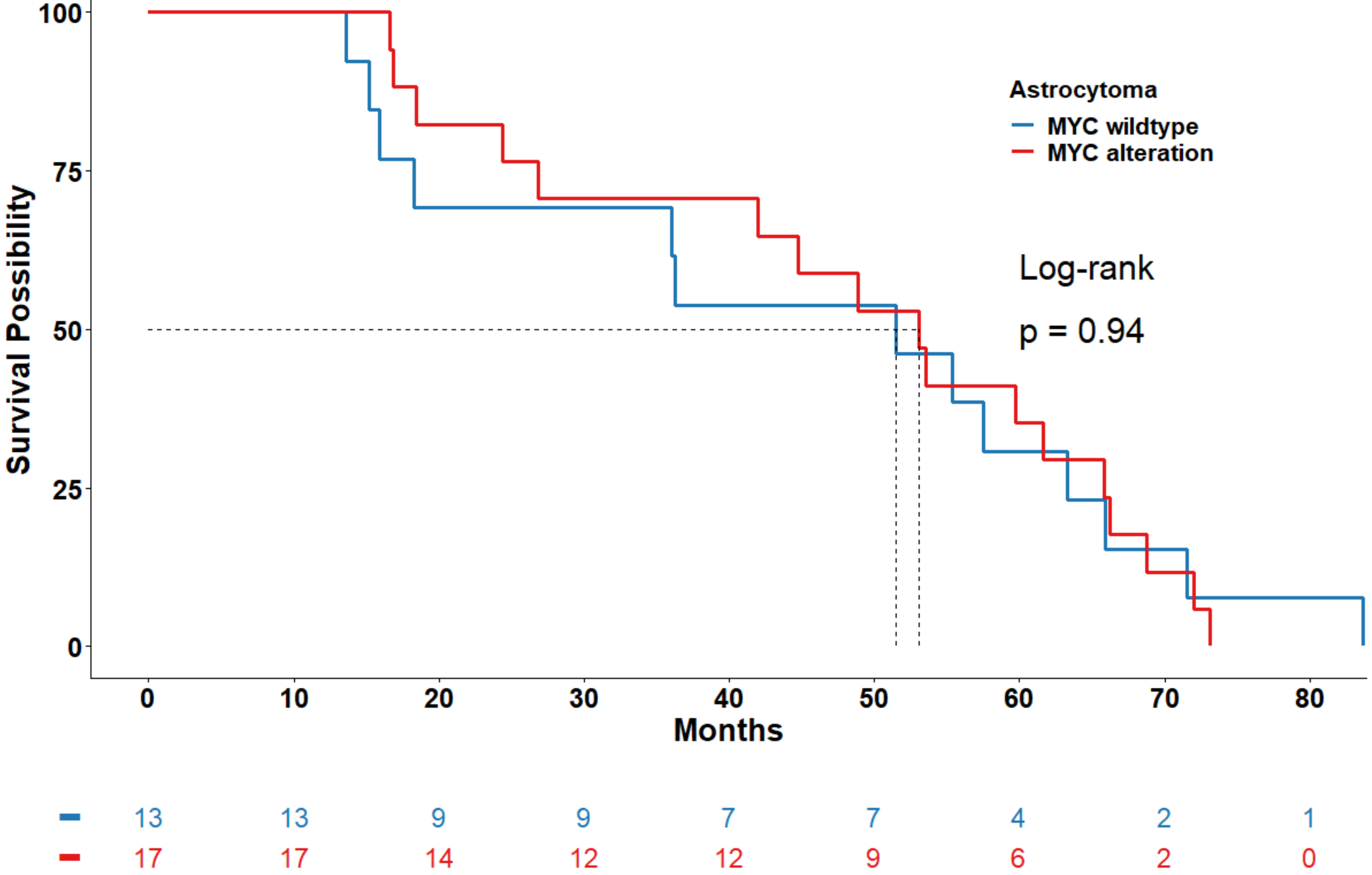
Survival Possibility
100
75
50
25
0
0
10
20
30
40
50
60
70
80
Months
Astrocytoma
MYC wildtype
MYC alteration
Log-rank
p = 0.94
13 13 9 9 7 7 4 2 1
17 17 14 12 12 9 6 2 0

(17)

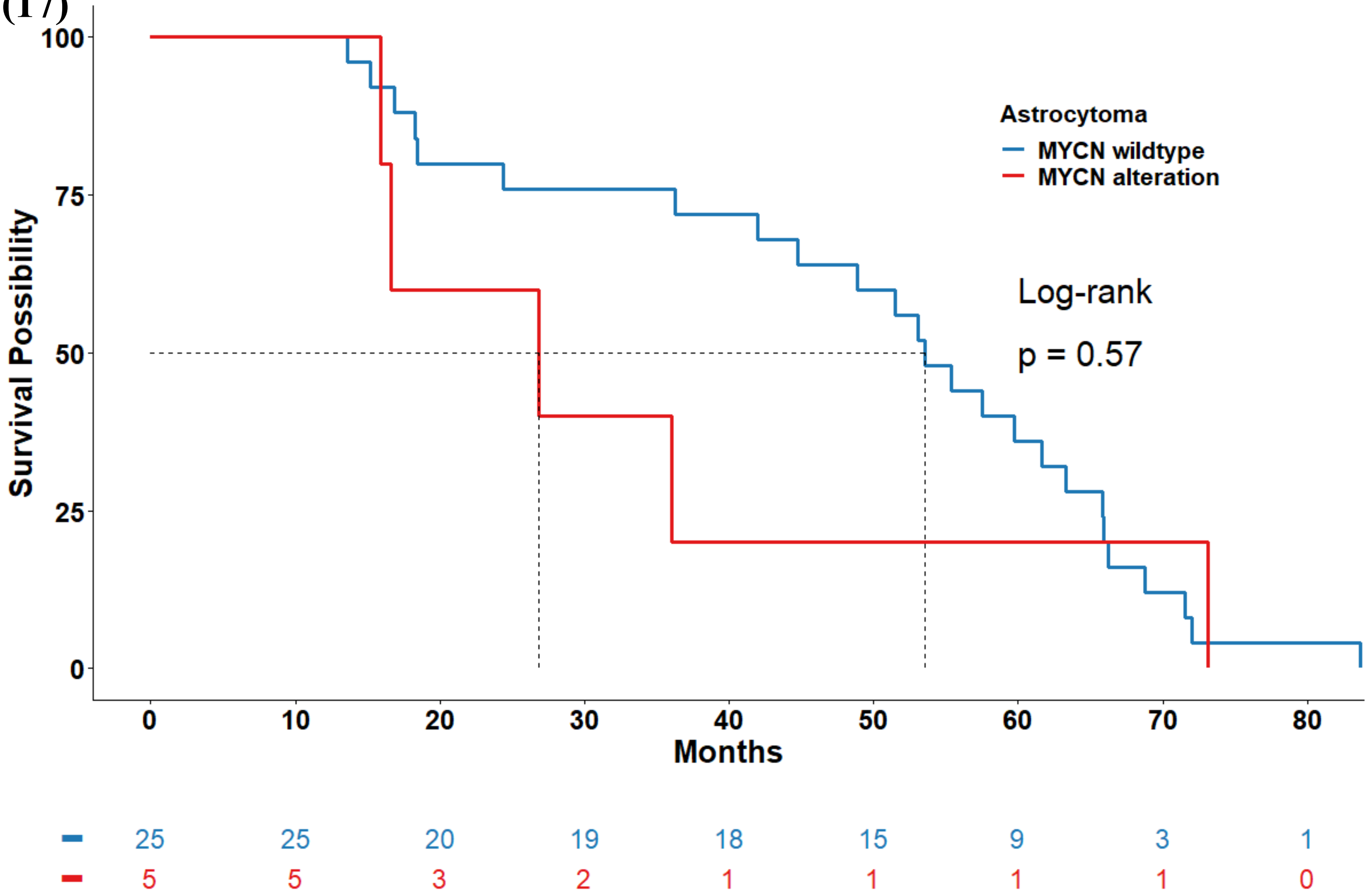

100
75
50
25
0
Survival Possibility
0
10
20
30
40
50
60
70
80
Months
Astrocytoma
MYCN wildtype
MYCN alteration
Log-rank
p = 0.57
25 25 20 19 18 15 9 3 1
5 5 3 2 1 1 1 1 0

(18)

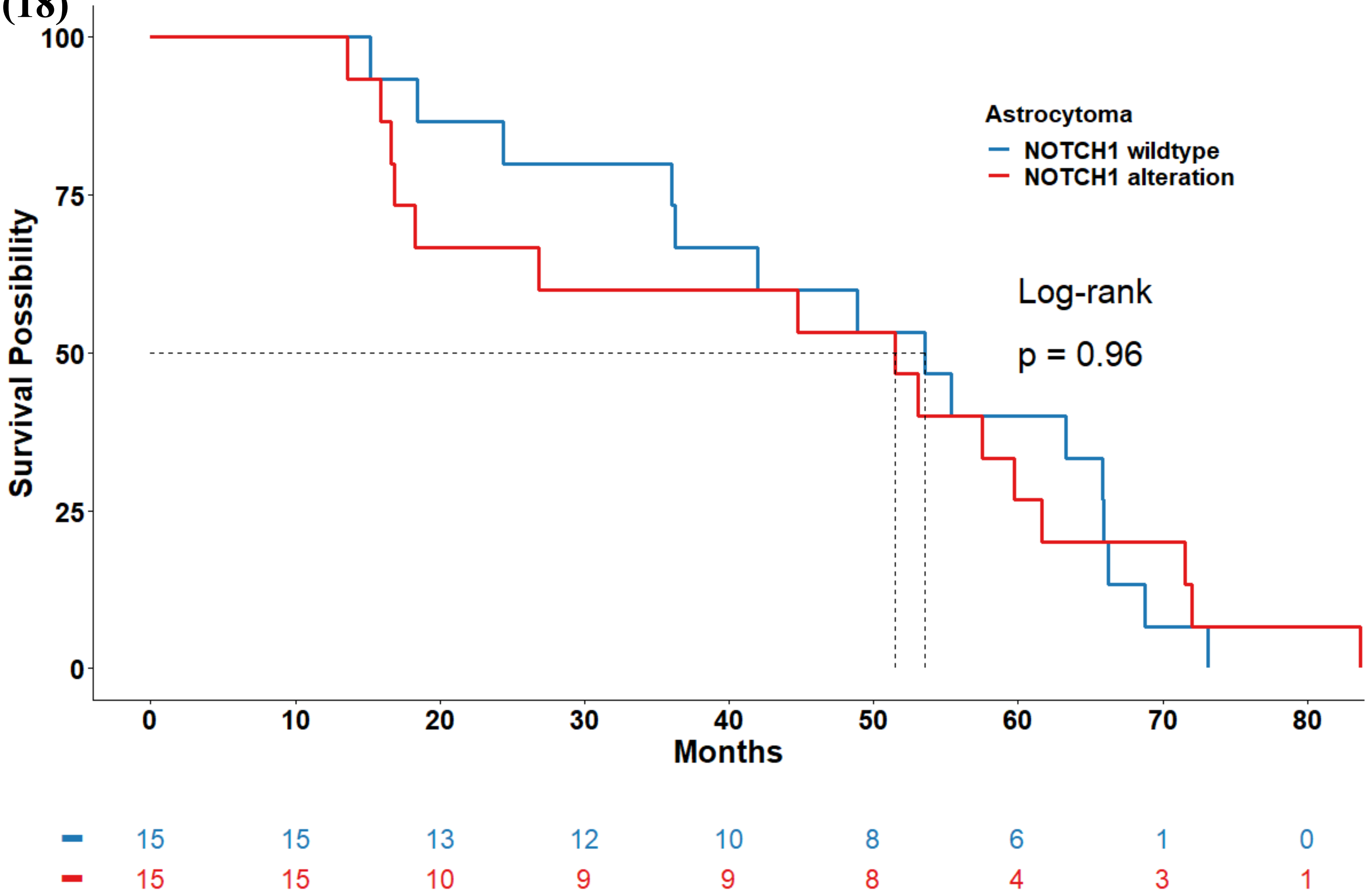
Survival Possibility
100
75
50
25
0
Astrocytoma
NOTCH1 wildtype
NOTCH1 alteration
Log-rank
p = 0.96
0
10
20
30
40
50
60
70
80
Months
15 15 13 12 10 8 6 1 0
15 15 10 9 9 8 4 3 1

(19)

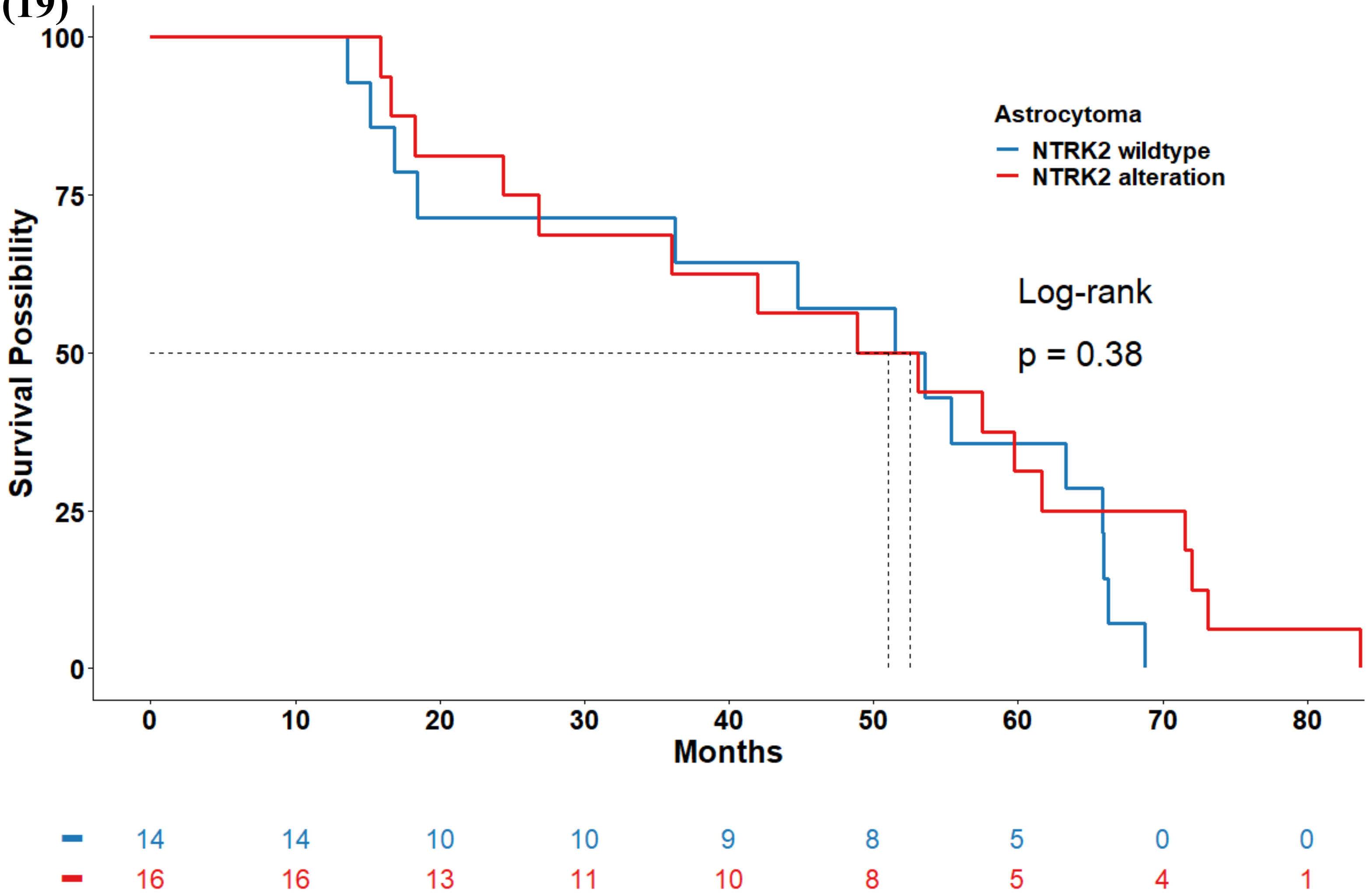

Astrocytoma
NTRK2 wildtype
NTRK2 alteration
Log-rank
p = 0.38
Survival Possibility
100
75
50
25
0
0
10
20
30
40
50
60
70
80
Months
14 14 10 10 9 8 5 0 0
16 16 13 11 10 8 5 4 1

(20)

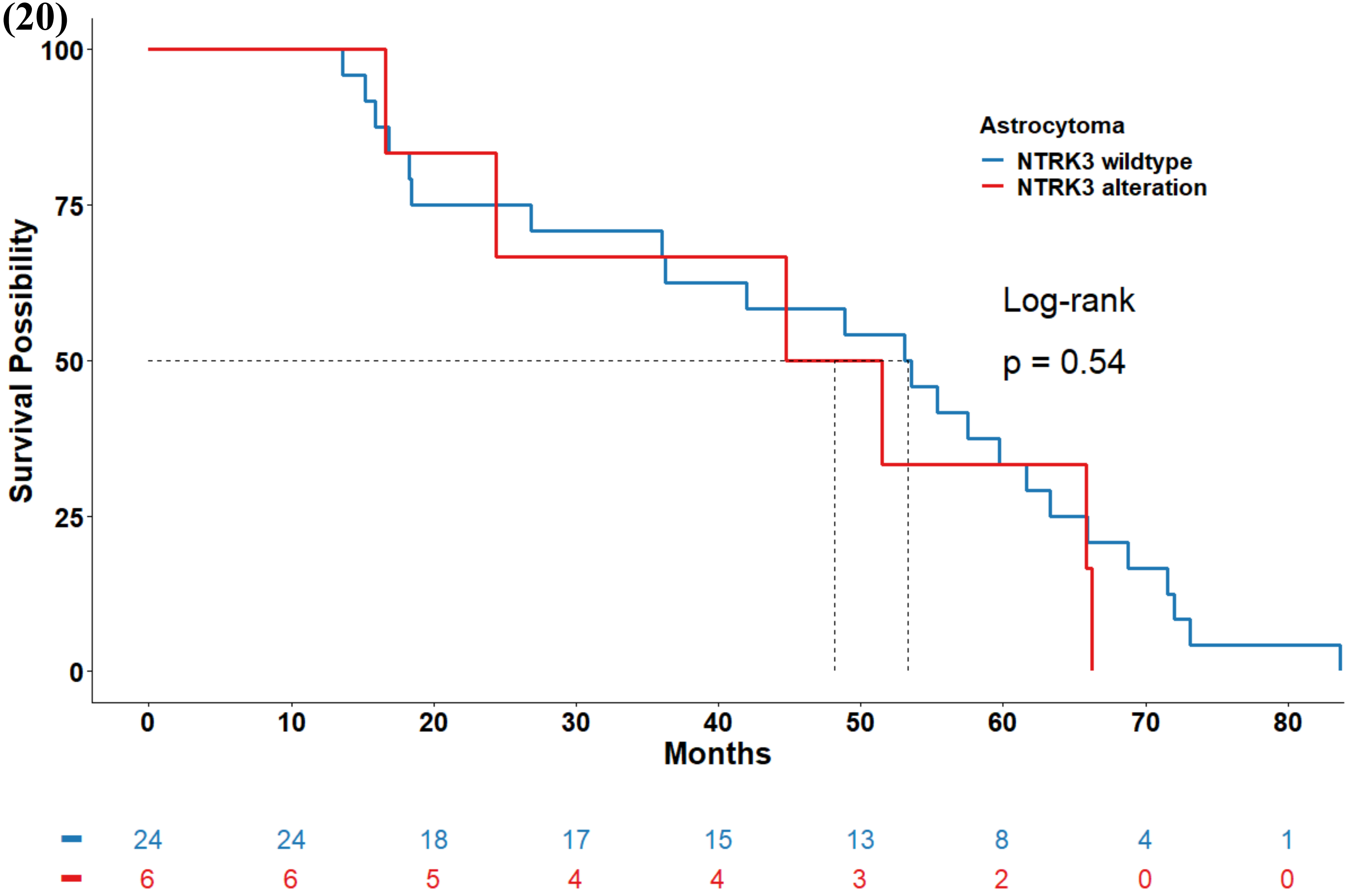

Astrocytoma
NTRK3 wildtype
NTRK3 alteration
Survival Possibility
100
75
50
25
0
0
10
20
30
40
50
60
70
80
Months
Log-rank
p = 0.54
24 24 18 17 15 13 8 4 1
6 6 5 4 4 3 2 0 0

(21)

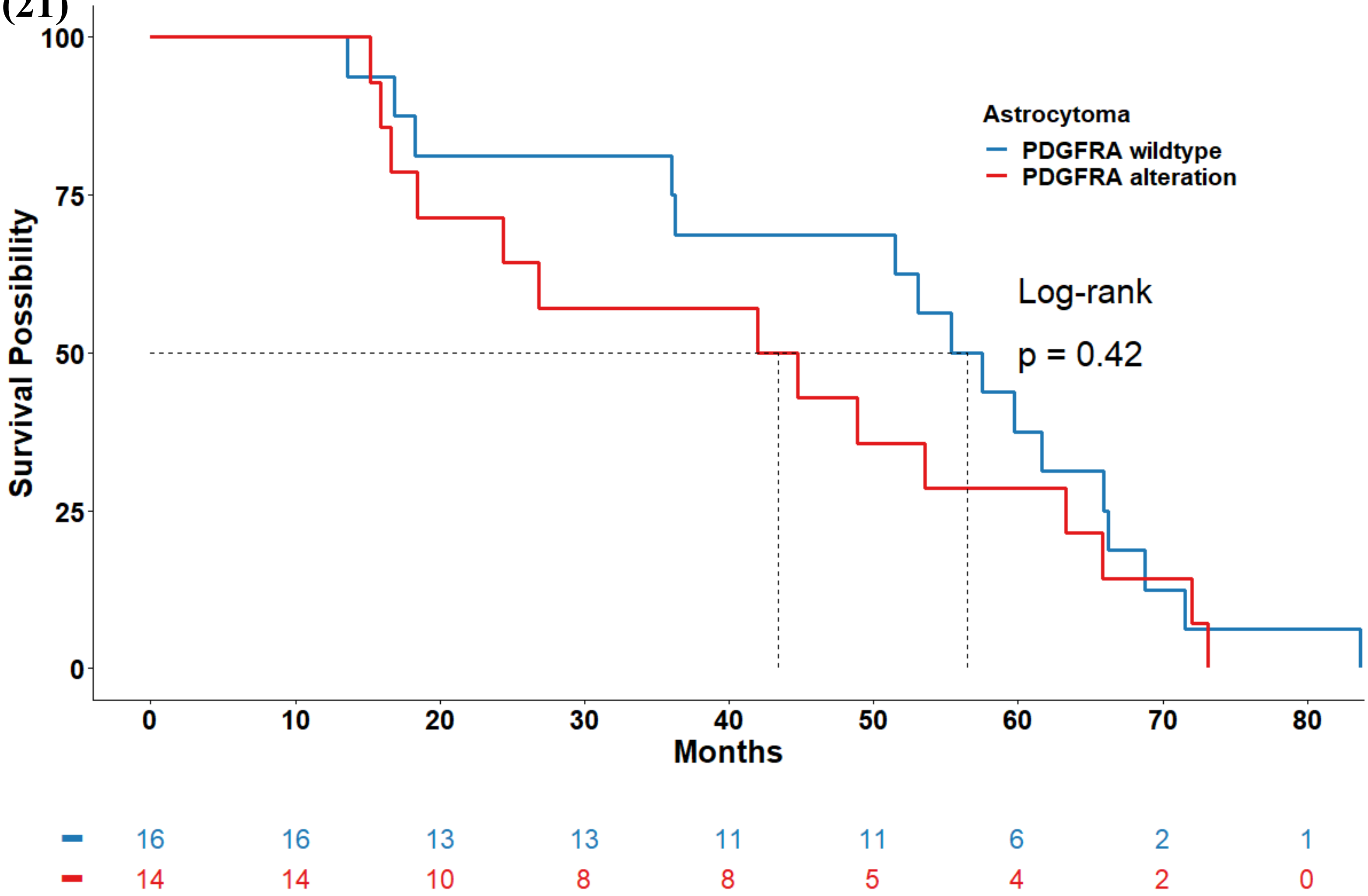
100
75
50
25
0
Survival Possibility
0
10
20
30
40
50
60
70
80
Months
Astrocytoma
PDGFRA wildtype
PDGFRA alteration
Log-rank
p = 0.42
16 16 13 13 11 11 6 2 1
14 14 10 8 8 5 4 2 0

(22)

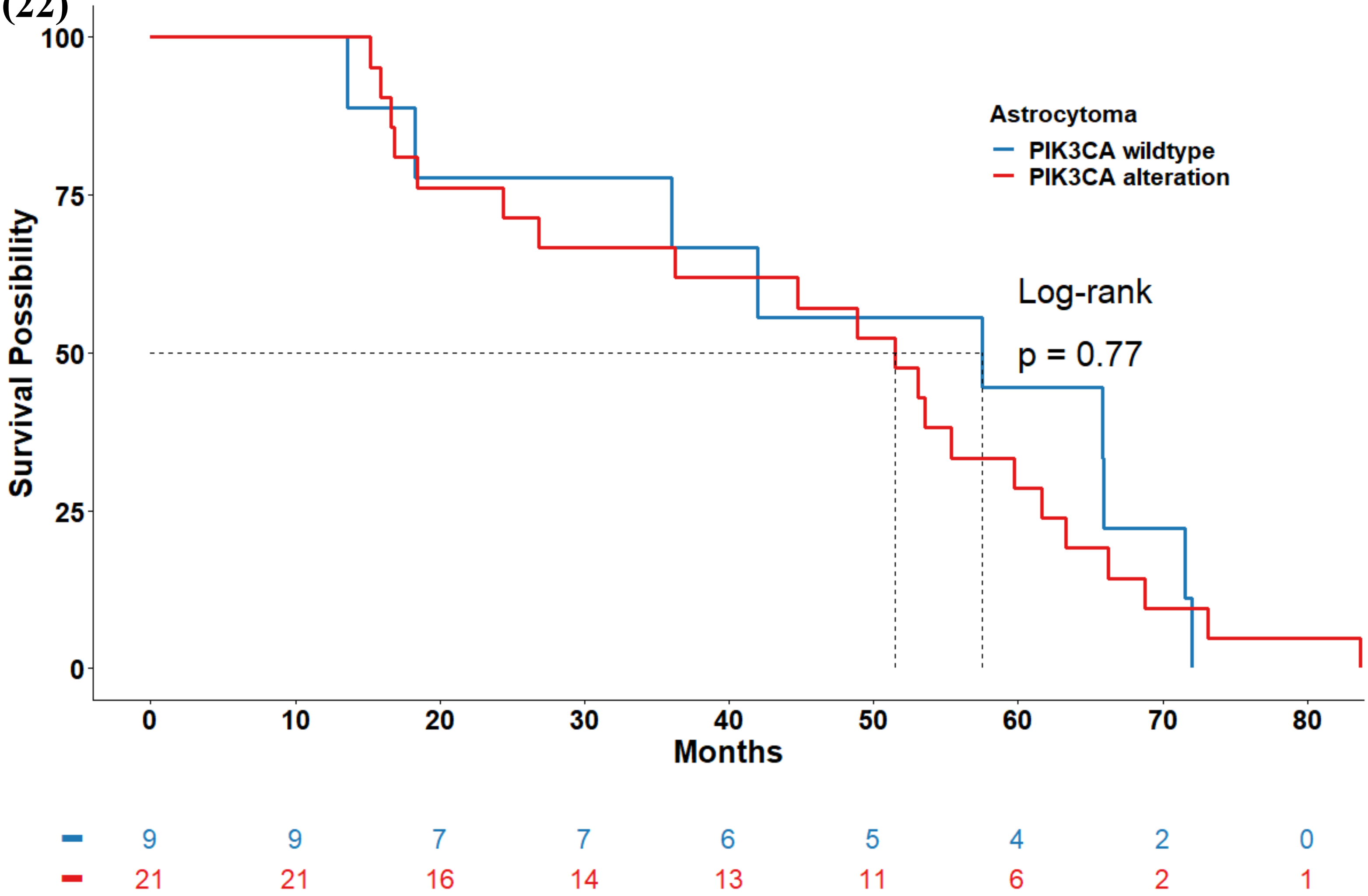
Survival Possibility
100
75
50
25
0
0
10
20
30
40
50
60
70
80
Months
Astrocytoma
PIK3CA wildtype
PIK3CA alteration
Log-rank
p = 0.77
9
9
7
7
6
5
4
2
0
21
21
16
14
13
11
6
2
1

(23)

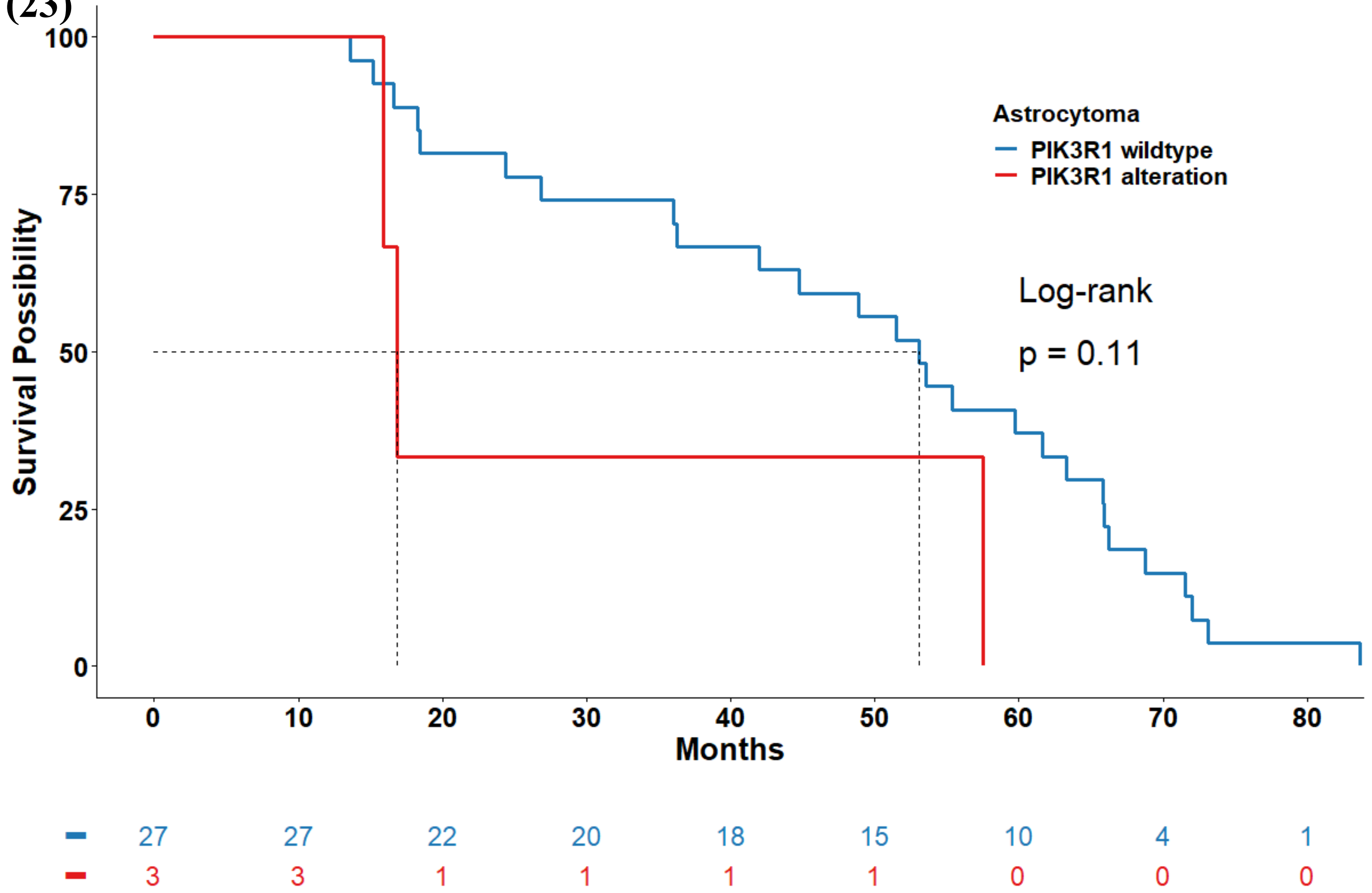
Survival Possibility
100
75
50
25
0
0
10
20
30
40
50
60
70
80
Months
Astrocytoma
PIK3R1 wildtype
PIK3R1 alteration
Log-rank
p = 0.11
27 27 22 20 18 15 10 4 1
3 3 1 1 1 1 0 0 0

(24)

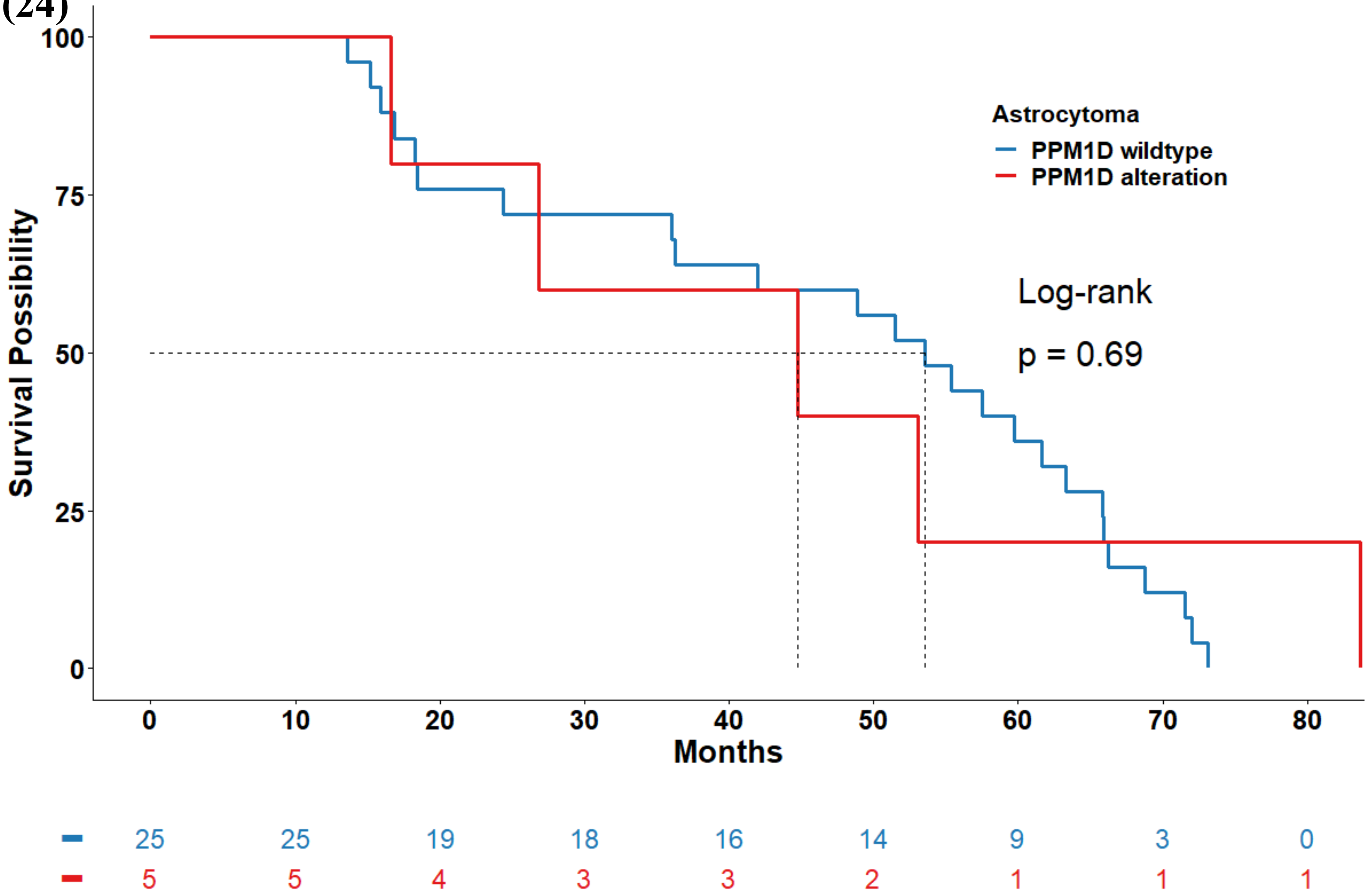

Survival Possibility
100
75
50
25
0
0
10
20
30
40
50
60
70
80
Months
Astrocytoma
PPM1D wildtype
PPM1D alteration
Log-rank
p = 0.69
25 25 19 18 16 14 9 3 0
5 5 4 3 3 2 1 1 1

(25)

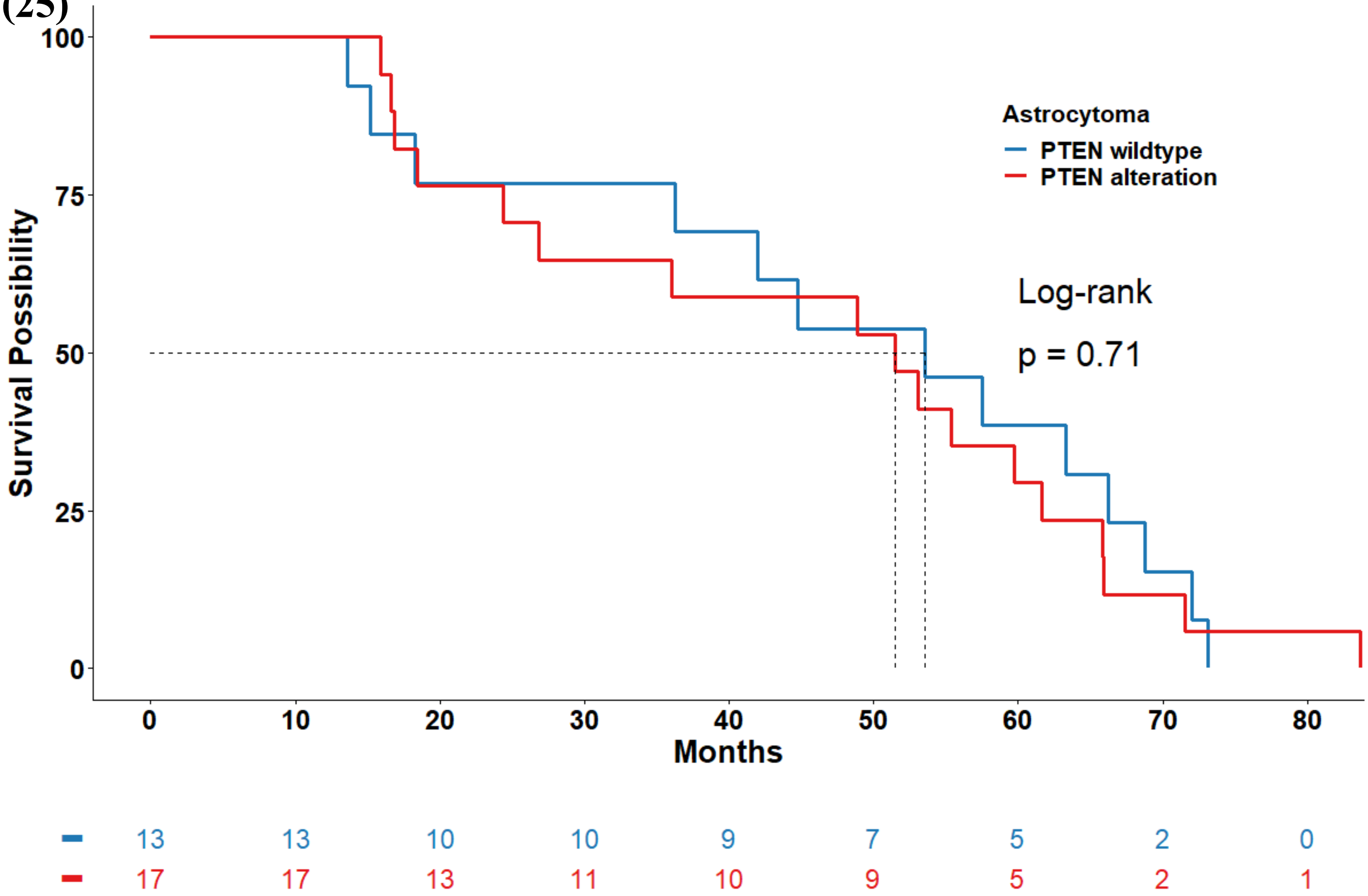
100
75
50
25
0
Survival Possibility
Astrocytoma
PTEN wildtype
PTEN alteration
Log-rank
p = 0.71
0
10
20
30
40
50
60
70
80
Months
13 13 10 10 9 7 5 2 0
17 17 13 11 10 9 5 2 1

(26)

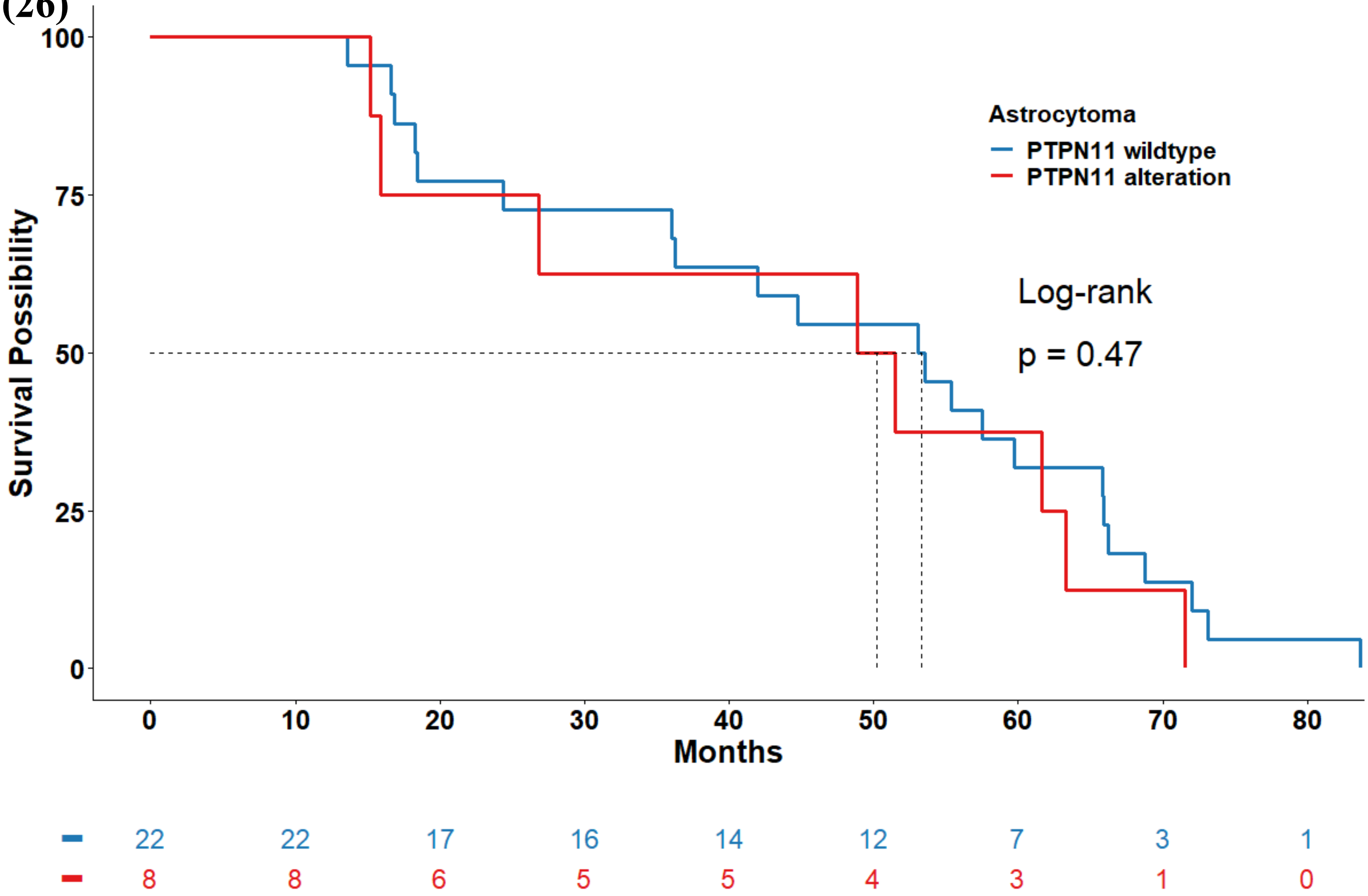
Survival Possibility
100
75
50
25
0
Astrocytoma
PTPN11 wildtype
PTPN11 alteration
Log-rank
p = 0.47
0
10
20
30
40
50
60
70
80
Months
22 22 17 16 14 12 7 3 1
8 8 6 5 5 4 3 1 0

(27)

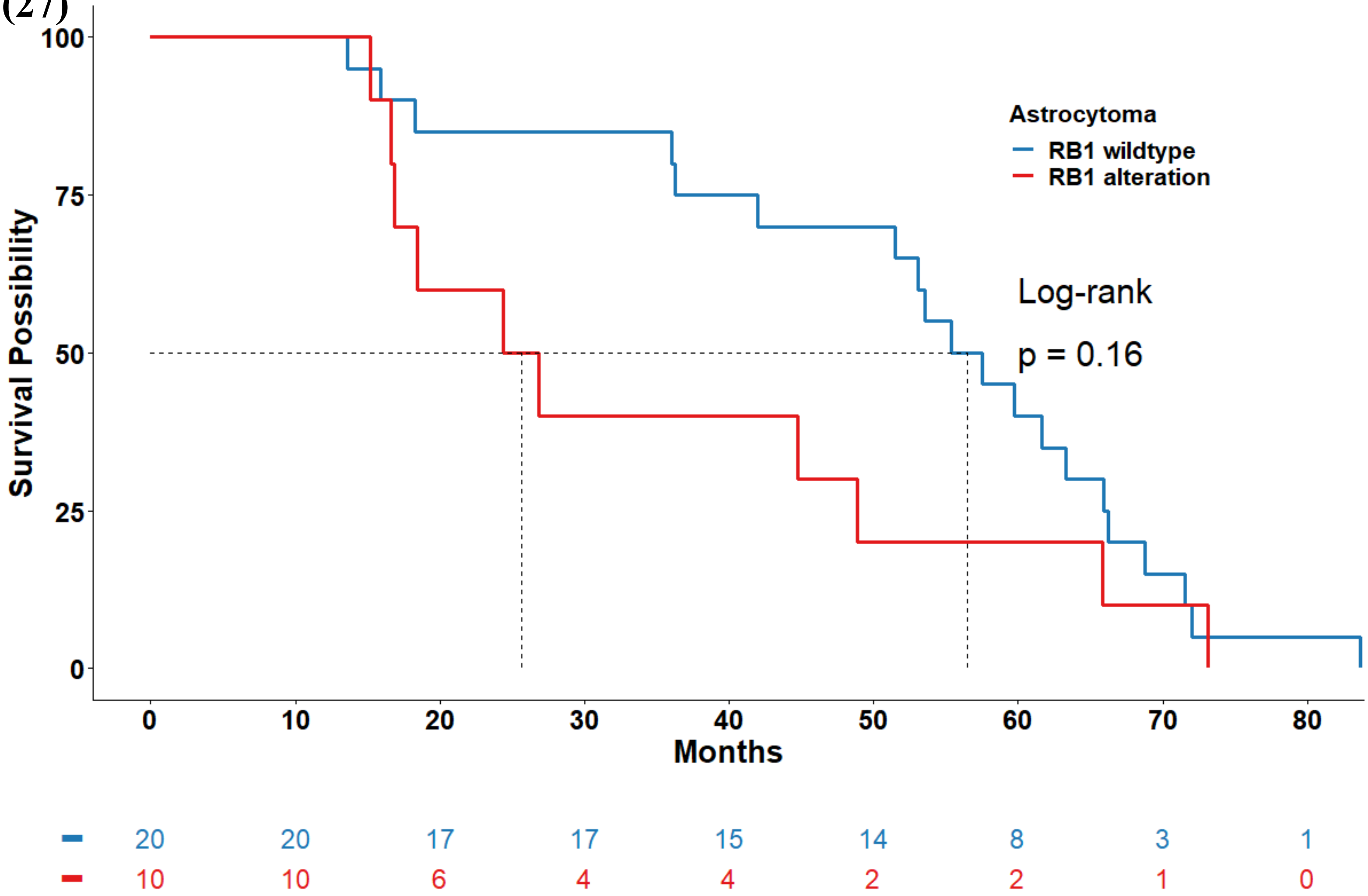

Astrocytoma
RB1 wildtype
RB1 alteration
Log-rank
p = 0.16
Survival Possibility
100
75
50
25
0
0
10
20
30
40
50
60
70
80
Months
20 20 17 17 15 14 8 3 1
10 10 6 4 4 2 2 1 0

(28)

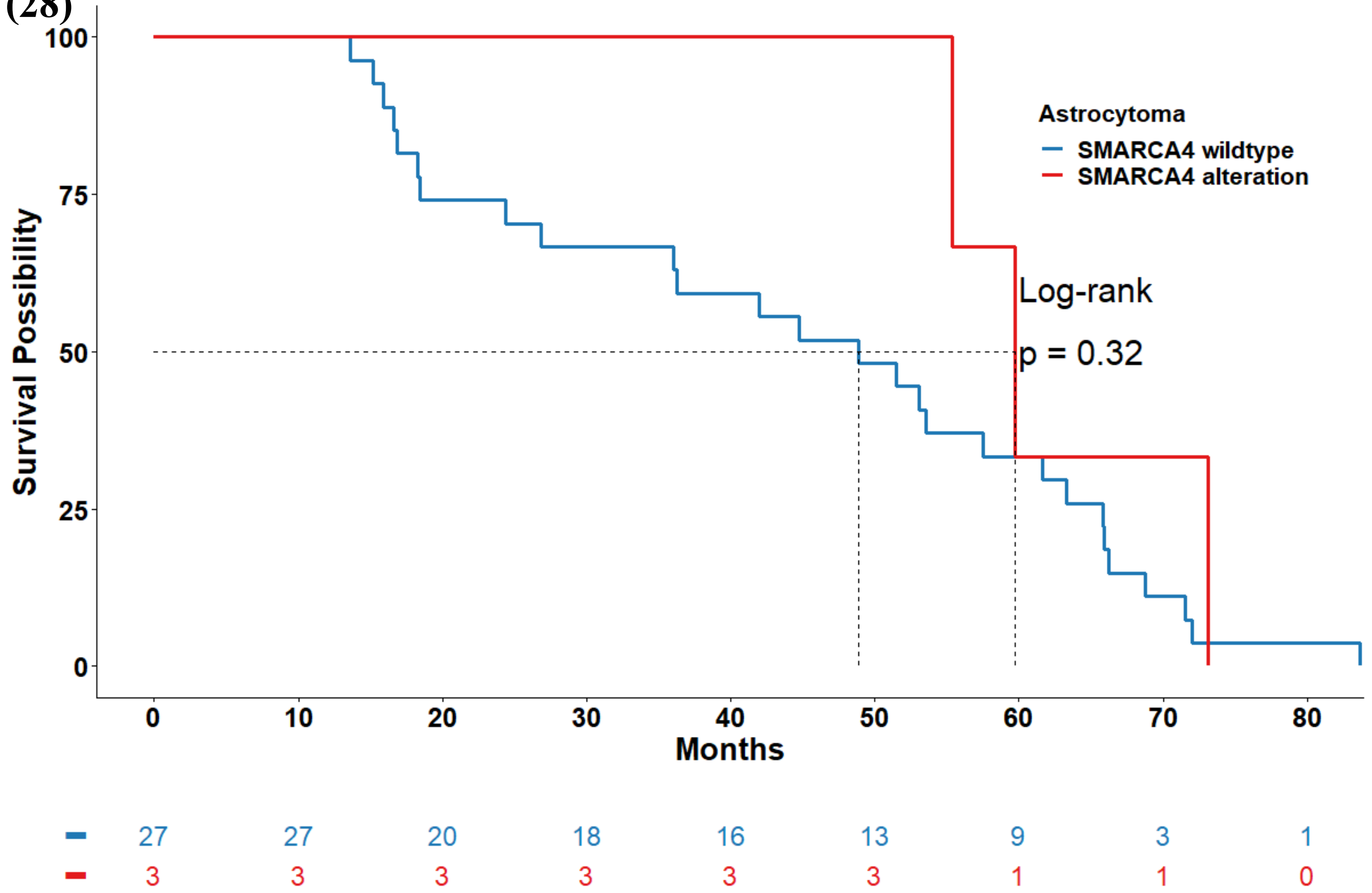
Astrocytoma
SMARCA4 wildtype
SMARCA4 alteration
Log-rank
p = 0.32
Survival Possibility
100
75
50
25
0
0
10
20
30
40
50
60
70
80
Months
27 27 20 18 16 13 9 3 1
3 3 3 3 3 3 1 1 0

(29)

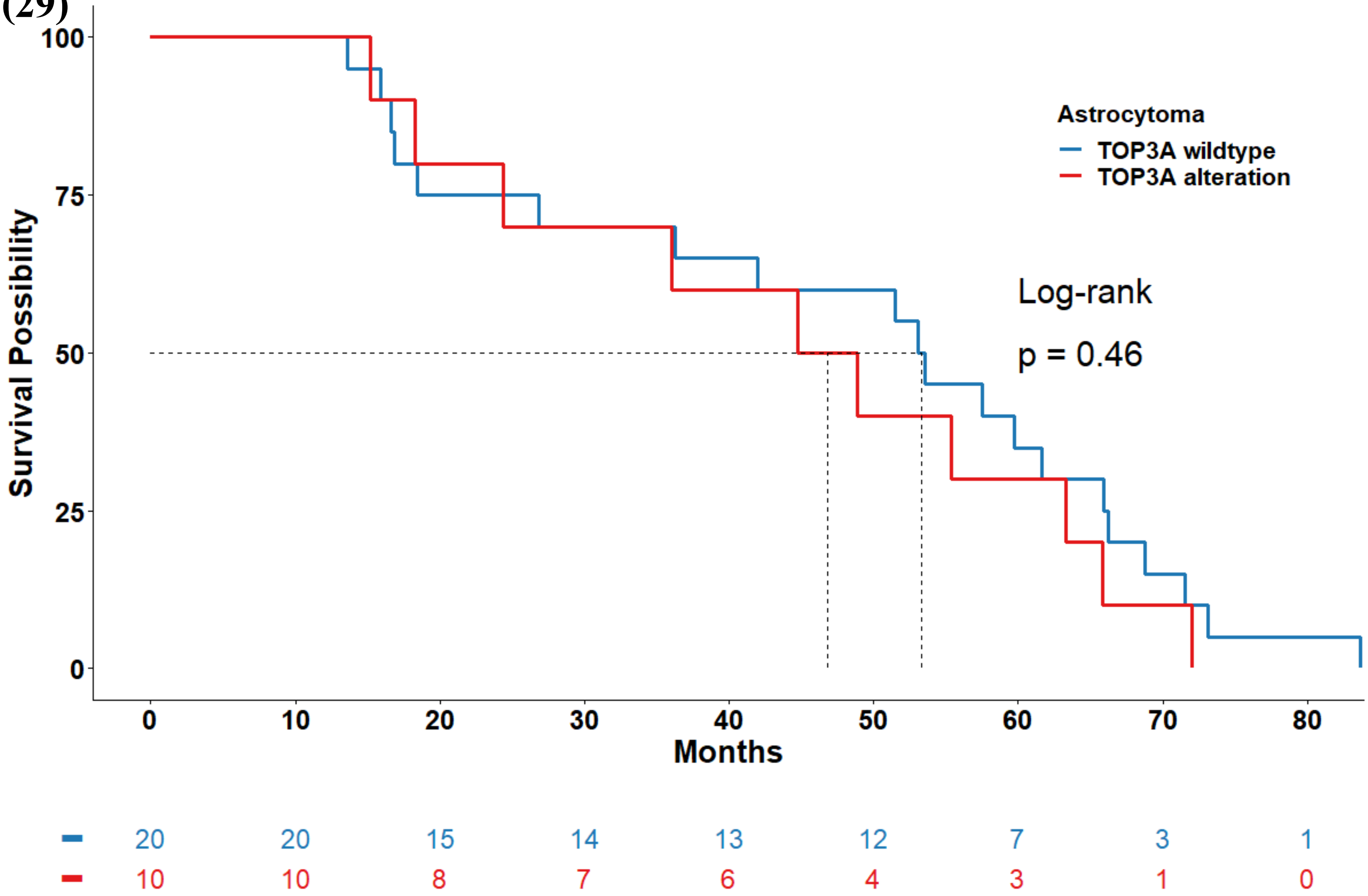

Astrocytoma
TOP3A wildtype
TOP3A alteration
Log-rank
p = 0.46
Survival Possibility
100
75
50
25
0
0
10
20
30
40
50
60
70
80
Months
20 20 15 14 13 12 7 3 1
10 10 8 7 6 4 3 1 0

(30)

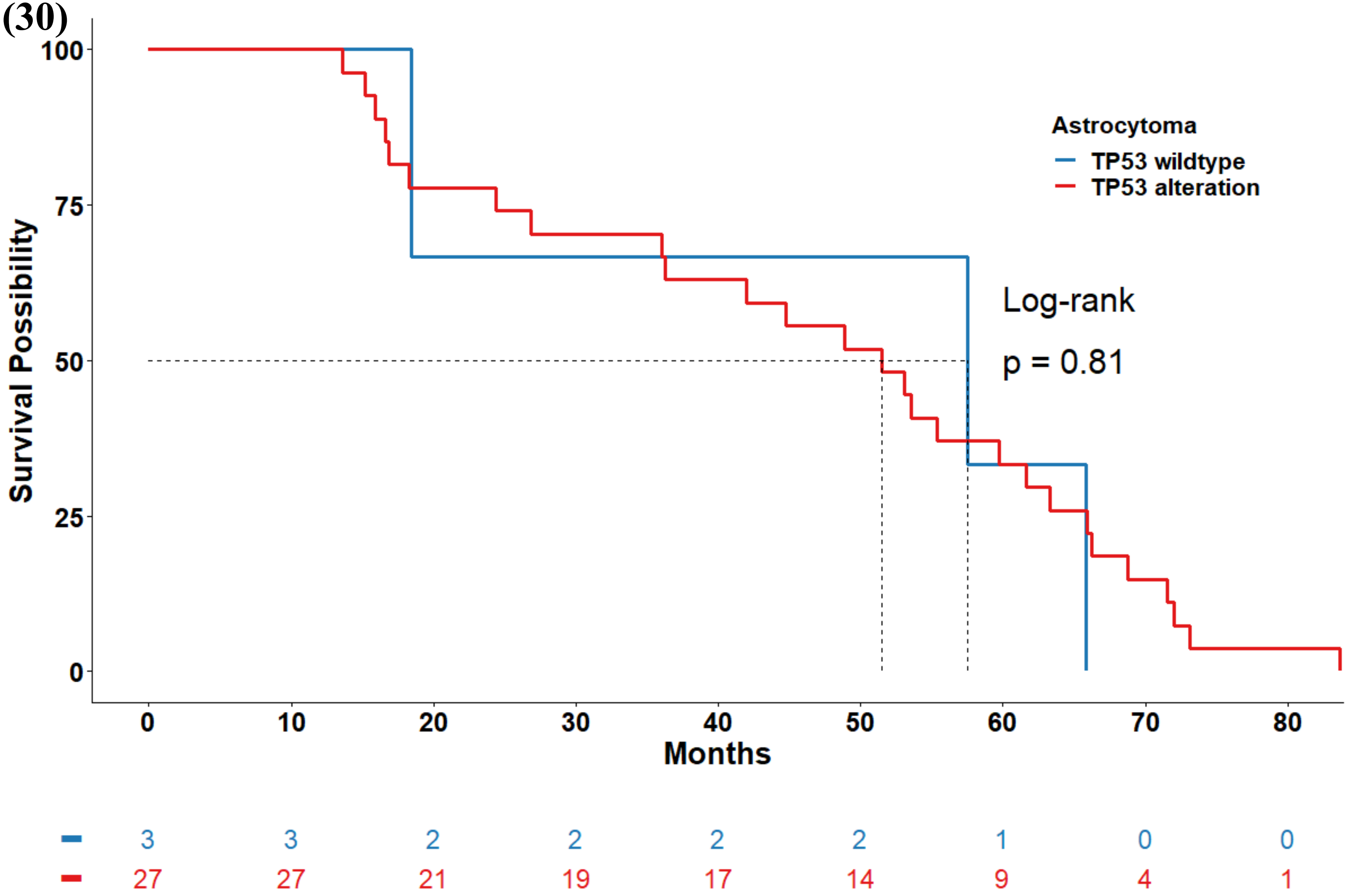
Astrocytoma
TP53 wildtype
TP53 alteration
Survival Possibility
100
75
50
25
0
Log-rank
p = 0.81
0
10
20
30
40
50
60
70
80
Months
3
3
2
2
2
2
1
0
0
27
27
21
19
17
14
9
4
1

(31)

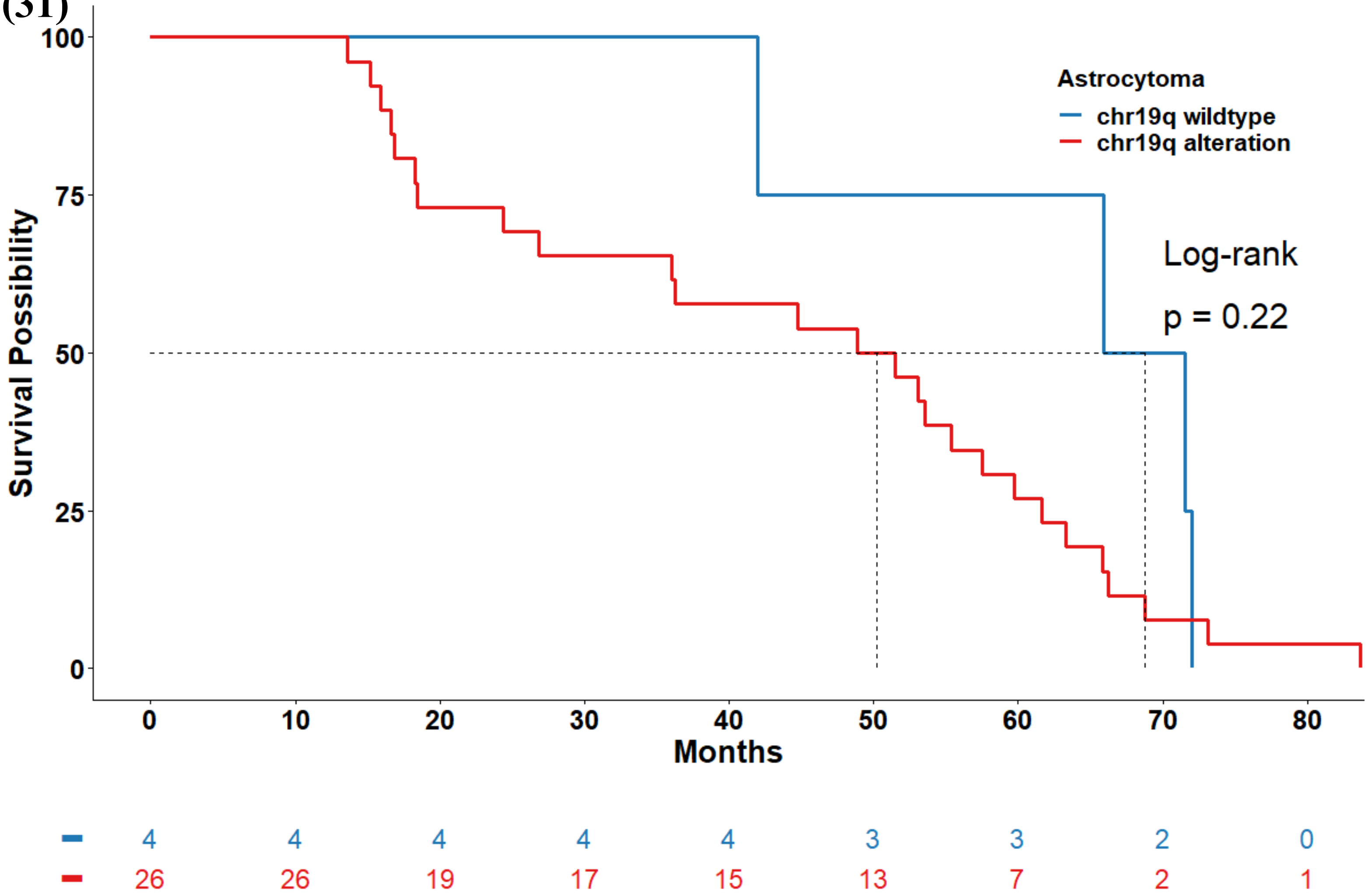
Astrocytoma
chr19q wildtype
chr19q alteration
Log-rank
p = 0.22
Survival Possibility
100
75
50
25
0
0
10
20
30
40
50
60
70
80
Months
4 4 4 4 4 3 3 2 0
26 26 19 17 15 13 7 2 1
