# Supplementary material for: Clinical updates on gliomas and implications of the 5th edition of the WHO classification of central nervous system tumors
Source: Front Oncol. 2023 Mar 14;13:1131642. doi: 10.3389/fonc.2023.1131642 (PMC10043404; doi:10.3389/fonc.2023.1131642)
Supplement: Supplementary file 3 [file DataSheet_3.pdf]

Supplement Figure 3. Kaplan–Meier curves showing no significant effects of certain genetic alterations on the overall survival of patients with glioneuronal and neuronal tumor(1-5). Since the numbers of patients divided into two groups (e.g. BRAF wildtype/ BRAF alteration) using different parameters varied, we only enrolled the parameters using which the number of patients in either group was above 3. Parameters used in this section were shown as follows.

(1) BRAF 1
(2) CDK4 2
(3) KRAS 3
(4) MET 4
(5) PEG3 5

(1)

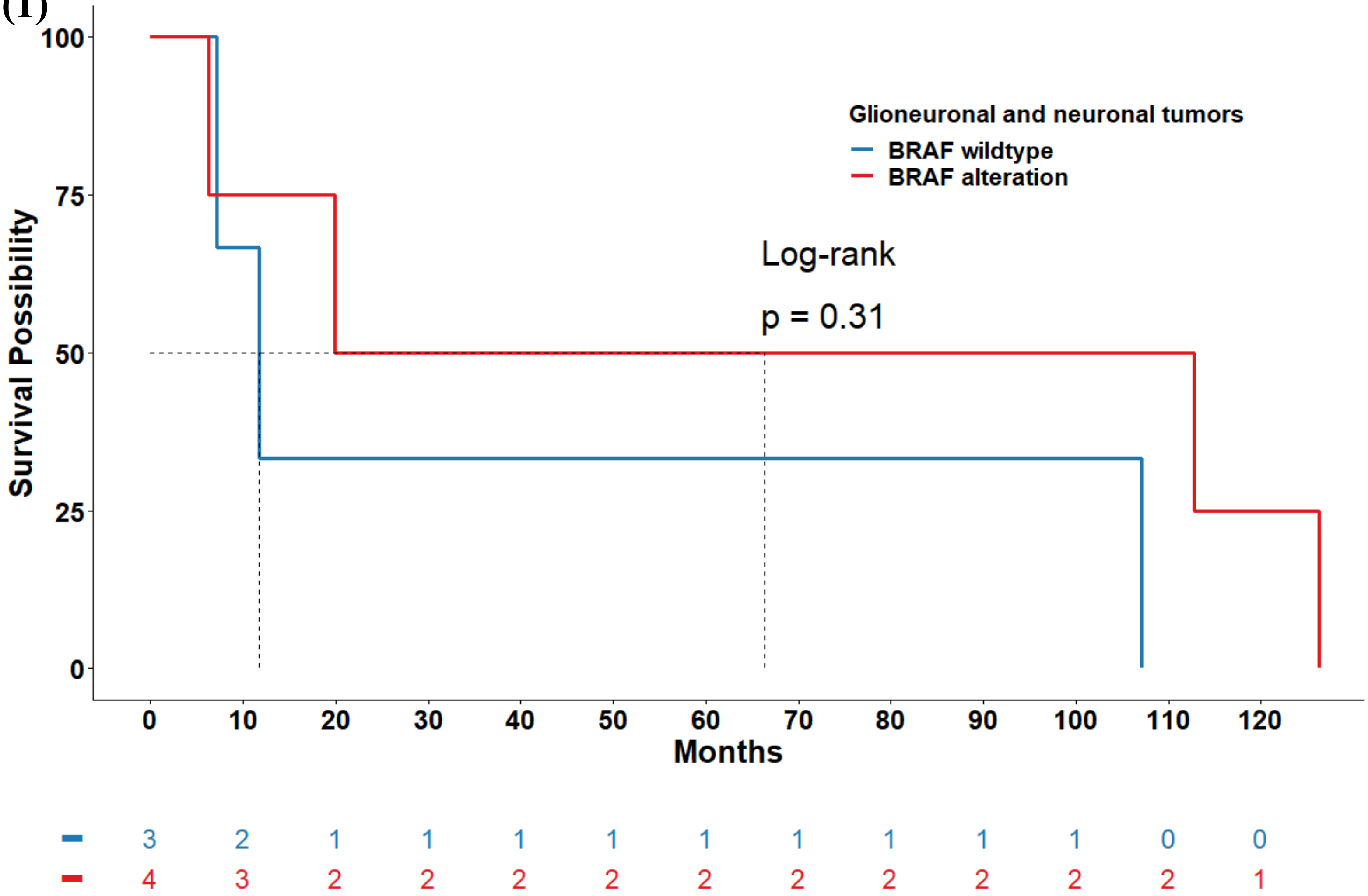

Glioneuronal and neuronal tumors
BRAF wildtype
BRAF alteration
Log-rank
p = 0.31
Survival Possibility
100
75
50
25
0
0
10
20
30
40
50
60
70
80
90
100
110
120
Months
3 2 1 1 1 1 1 1 1 1 1 0 0
4 3 2 2 2 2 2 2 2 2 2 2 1

(2)

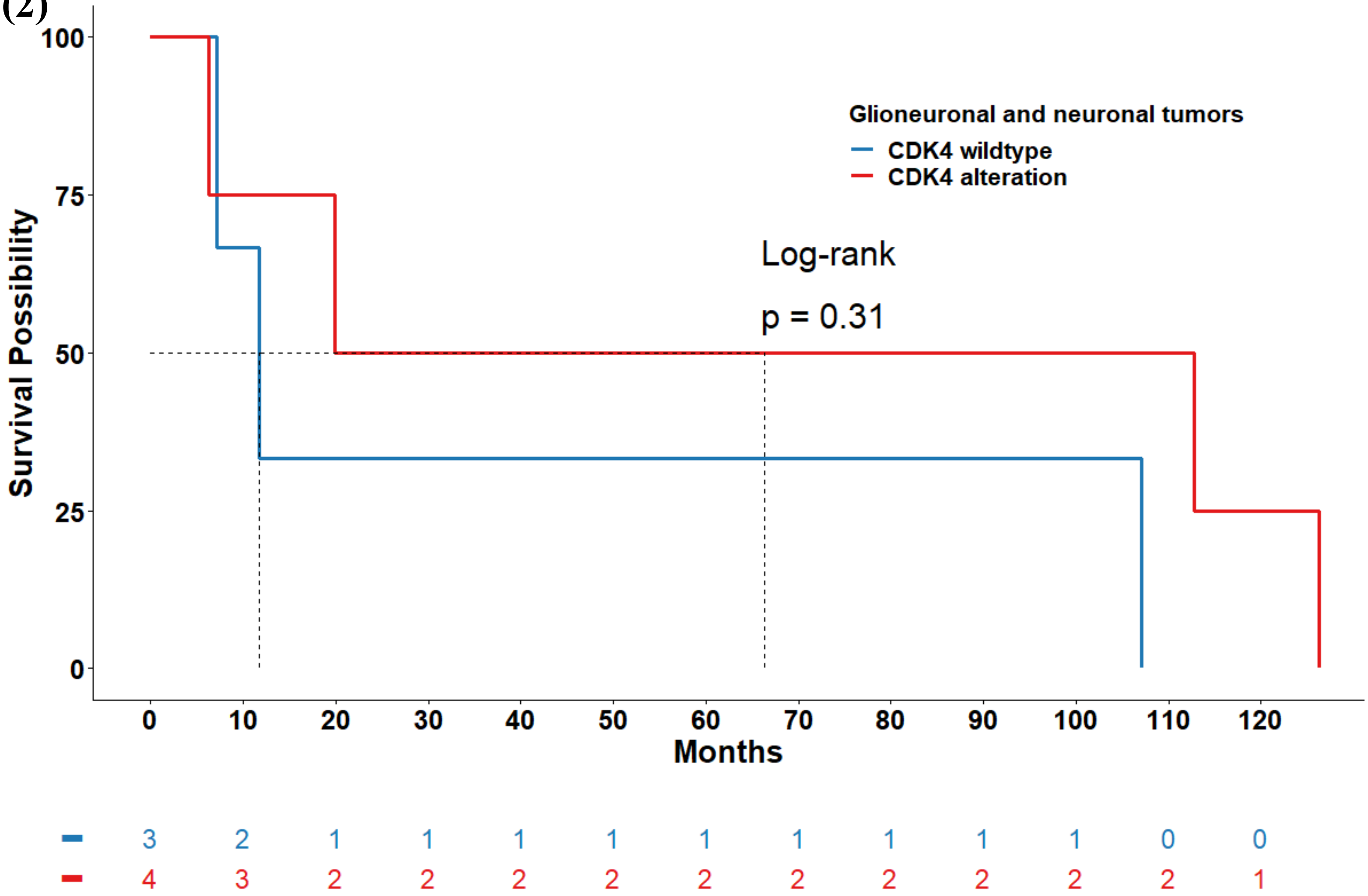
Survival Possibility
100
75
50
25
0
Glioneuronal and neuronal tumors
CDK4 wildtype
CDK4 alteration
Log-rank
p = 0.31
0
10
20
30
40
50
60
70
80
90
100
110
120
Months
3 2 1 1 1 1 1 1 1 1 1 0 0
4 3 2 2 2 2 2 2 2 2 2 2 1

(3)

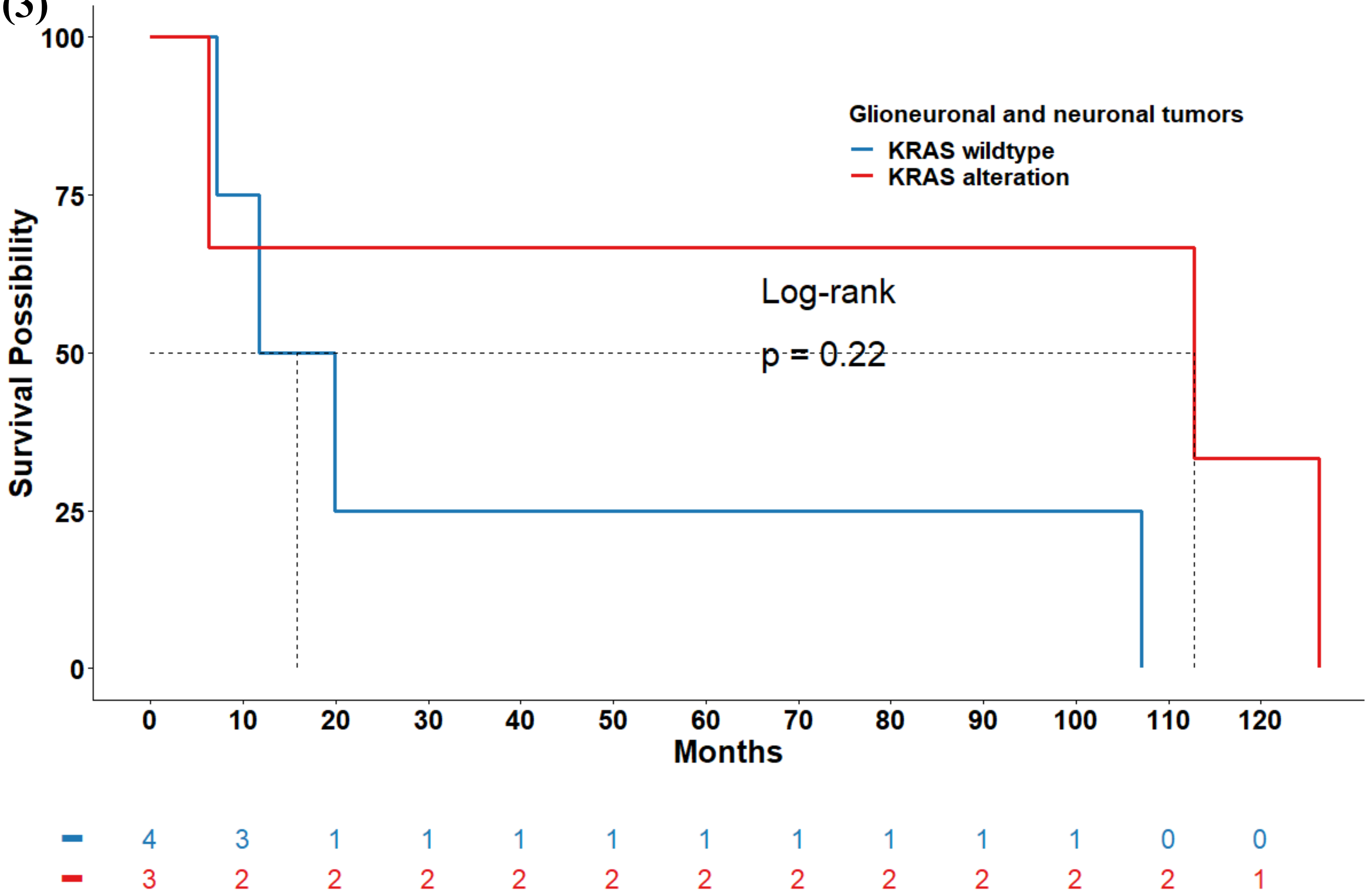

Survival Possibility
100
75
50
25
0
Glioneuronal and neuronal tumors
KRAS wildtype
KRAS alteration
Log-rank
p = 0.22
0
10
20
30
40
50
60
70
80
90
100
110
120
Months
4 3 1 1 1 1 1 1 1 1 1 0 0
3 2 2 2 2 2 2 2 2 2 2 2 1

(4)

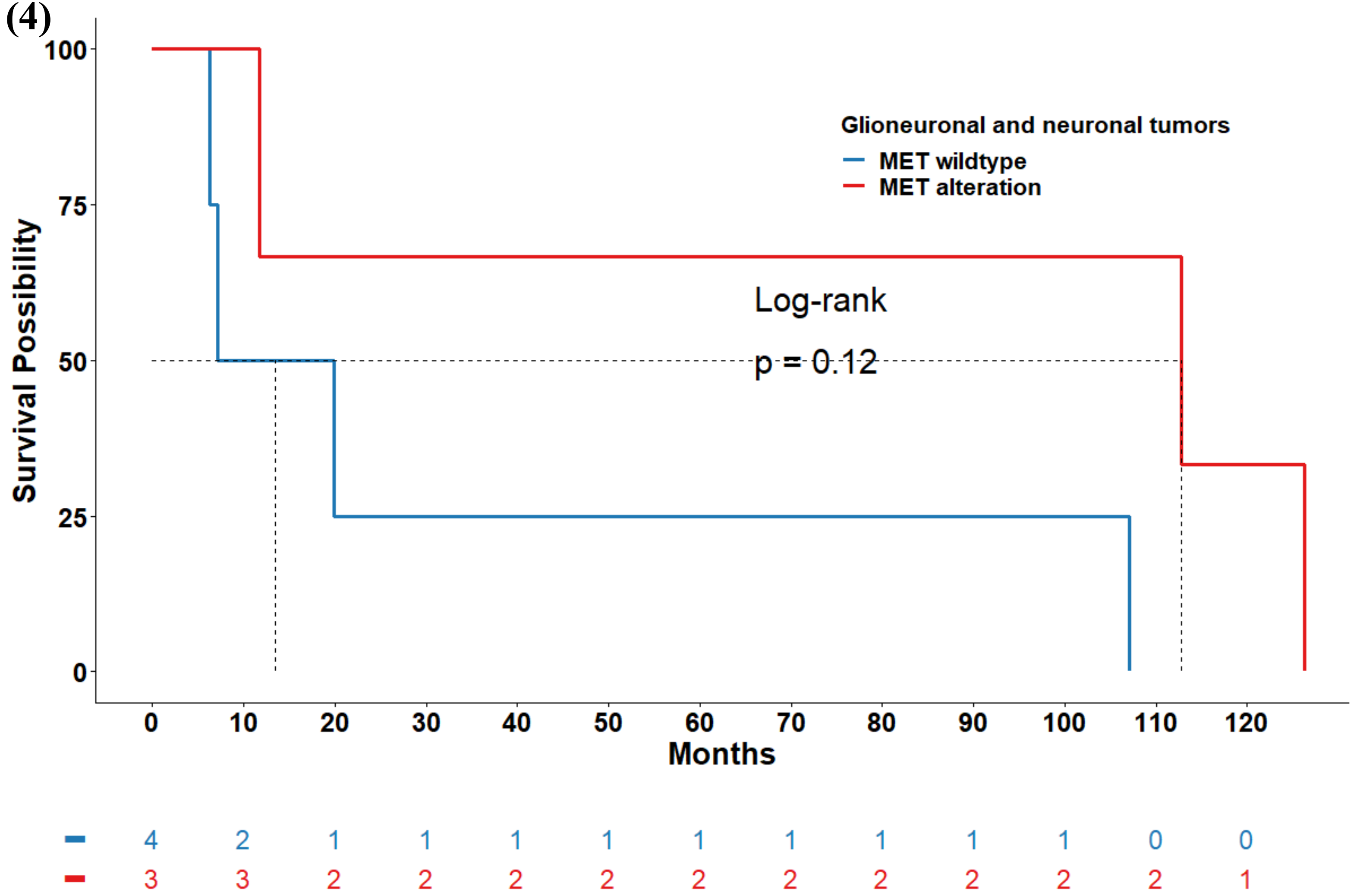
Glioneuronal and neuronal tumors
MET wildtype
MET alteration
Survival Possibility
100
75
50
25
0
Log-rank
p = 0.12
0 10 20 30 40 50 60 70 80 90 100 110 120
Months
4 2 1 1 1 1 1 1 1 1 1 0 0
3 3 2 2 2 2 2 2 2 2 2 2 1

(5)

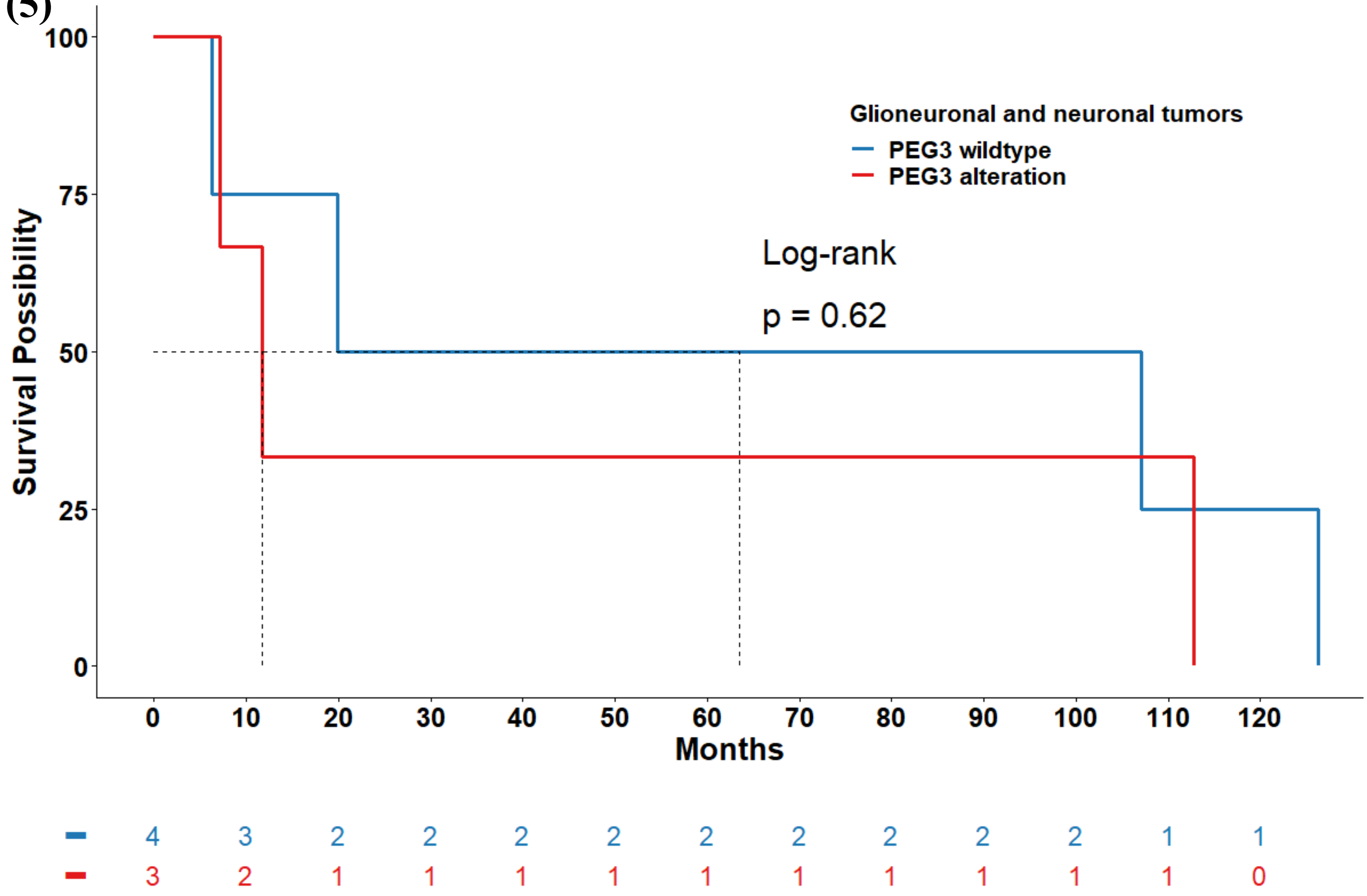

Glioneuronal and neuronal tumors
PEG3 wildtype
PEG3 alteration
Log-rank
p = 0.62
Survival Possibility
100
75
50
25
0
0
10
20
30
40
50
60
70
80
90
100
110
120
Months
4 3 2 2 2 2 2 2 2 2 2 1 1
3 2 1 1 1 1 1 1 1 1 1 1 0
