# Supplementary material for: Clinical updates on gliomas and implications of the 5th edition of the WHO classification of central nervous system tumors
Source: Front Oncol. 2023 Mar 14;13:1131642. doi: 10.3389/fonc.2023.1131642 (PMC10043404; doi:10.3389/fonc.2023.1131642)
Supplement: Supplementary file 4 [file DataSheet_4.pdf]

Supplement Figure 4. Kaplan–Meier curves showing no significant effects of certain genetic alterations on the overall survival of patients with oligodendroglioma (1-29). Since the numbers of patients divided into two groups (e.g. BRAF wildtype/ BRAF alteration) using different parameters varied, we only enrolled the parameters using which the number of patients in either group was above 3. Parameters used in this section were shown as follows.

(1) BRAF 1
(2) CDK6 2
(3) CDKN2A 3
(4) CDKN2B 4
(5) CIC 5
(6) EGFR 6
(7) FGFR1 7
(8) FGFR2 8
(9) FGFR3 9
(10) FGFR4 10
(11) KIT 11
(12) KMT5B 12
(13) KRAS 13
(14) MET 14
(15) MYB 15
(16) MYBL1 16
(17) MYC 17
(18) MYCN 18
(19) NOTCH1 19
(20) NTRK3 20
(21) PDGFRA 21
(22) PIK3CA 22
(23) PPM1D 23
(24) PTEN 24
(25) PTPN11 25
(26) RB1 26
(27) TOP3A 27
(28) TSC2 28
(29) chr7q 29

(1)

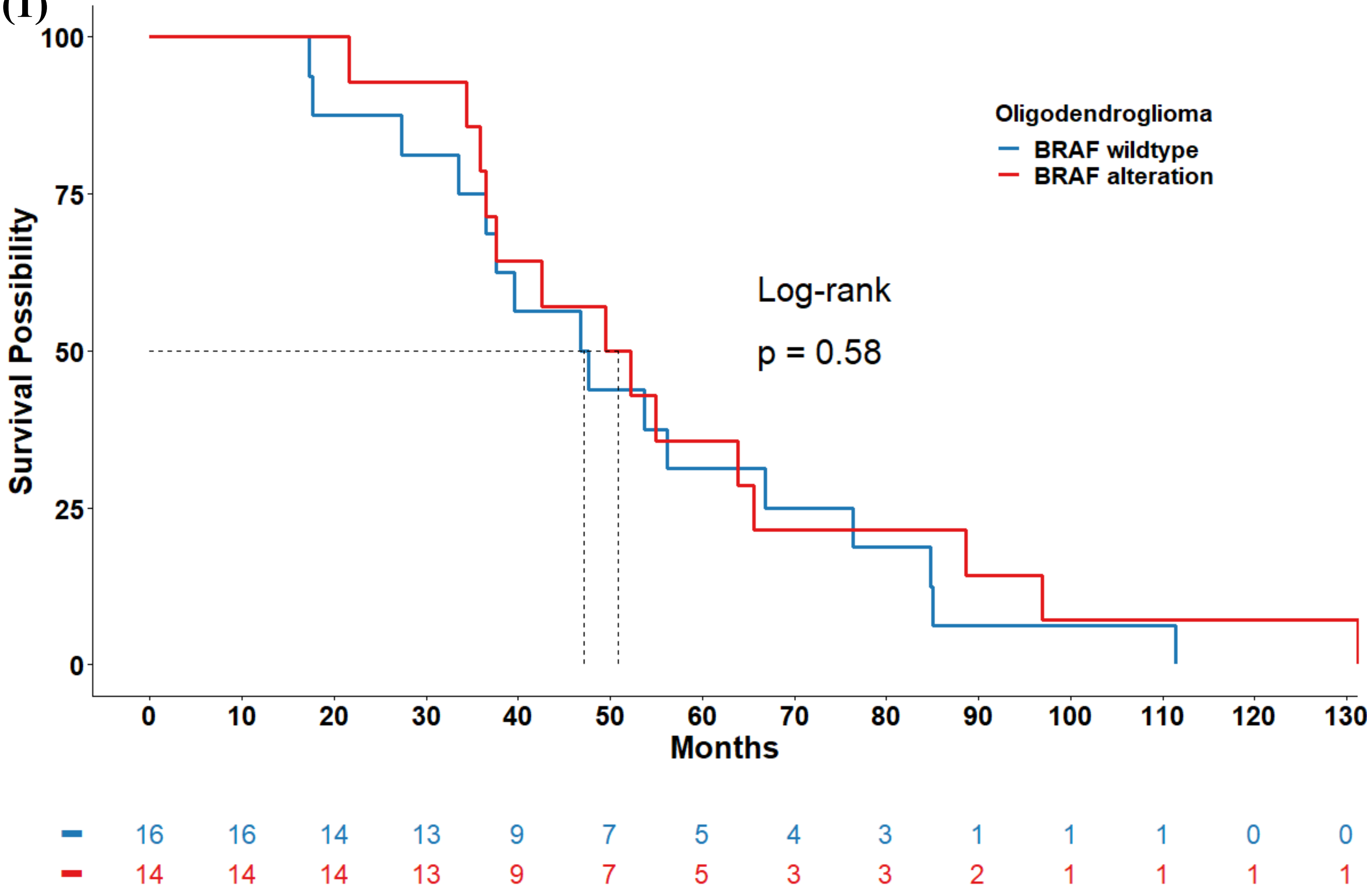

Survival Possibility
100
75
50
25
0
Oligodendroglioma
BRAF wildtype
BRAF alteration
Log-rank
p = 0.58
0
10
20
30
40
50
60
70
80
90
100
110
120
130
Months
16 16 14 13 9 7 5 4 3 1 1 1 0 0
14 14 14 13 9 7 5 3 3 2 1 1 1 1

(2)

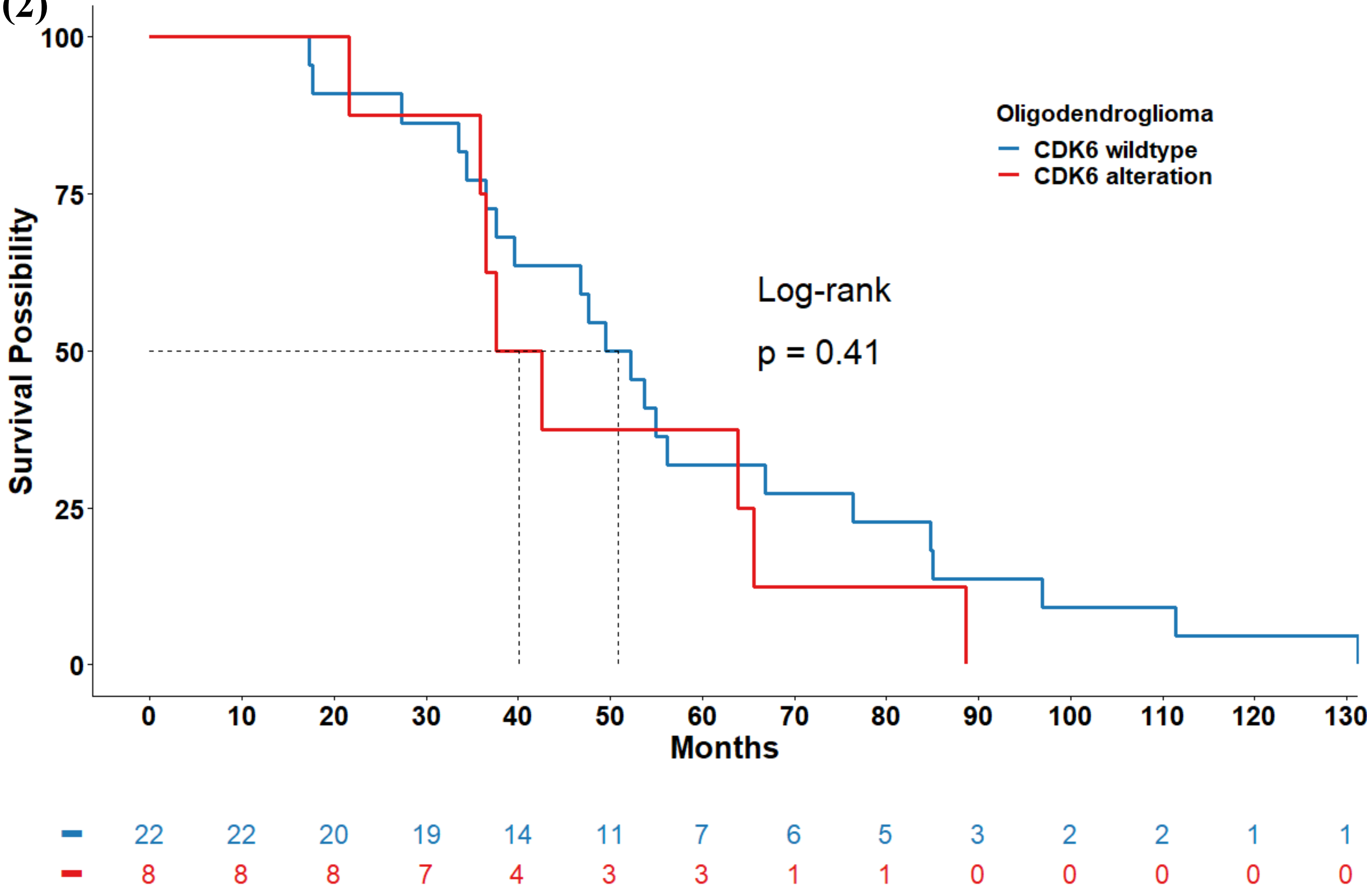
Survival Possibility
100
75
50
25
0
0 10 20 30 40 50 60 70 80 90 100 110 120 130
Months
Oligodendroglioma
CDK6 wildtype
CDK6 alteration
Log-rank
p = 0.41
22 22 20 19 14 11 7 6 5 3 2 2 1 1
8 8 8 7 4 3 3 1 1 0 0 0 0 0

(3)

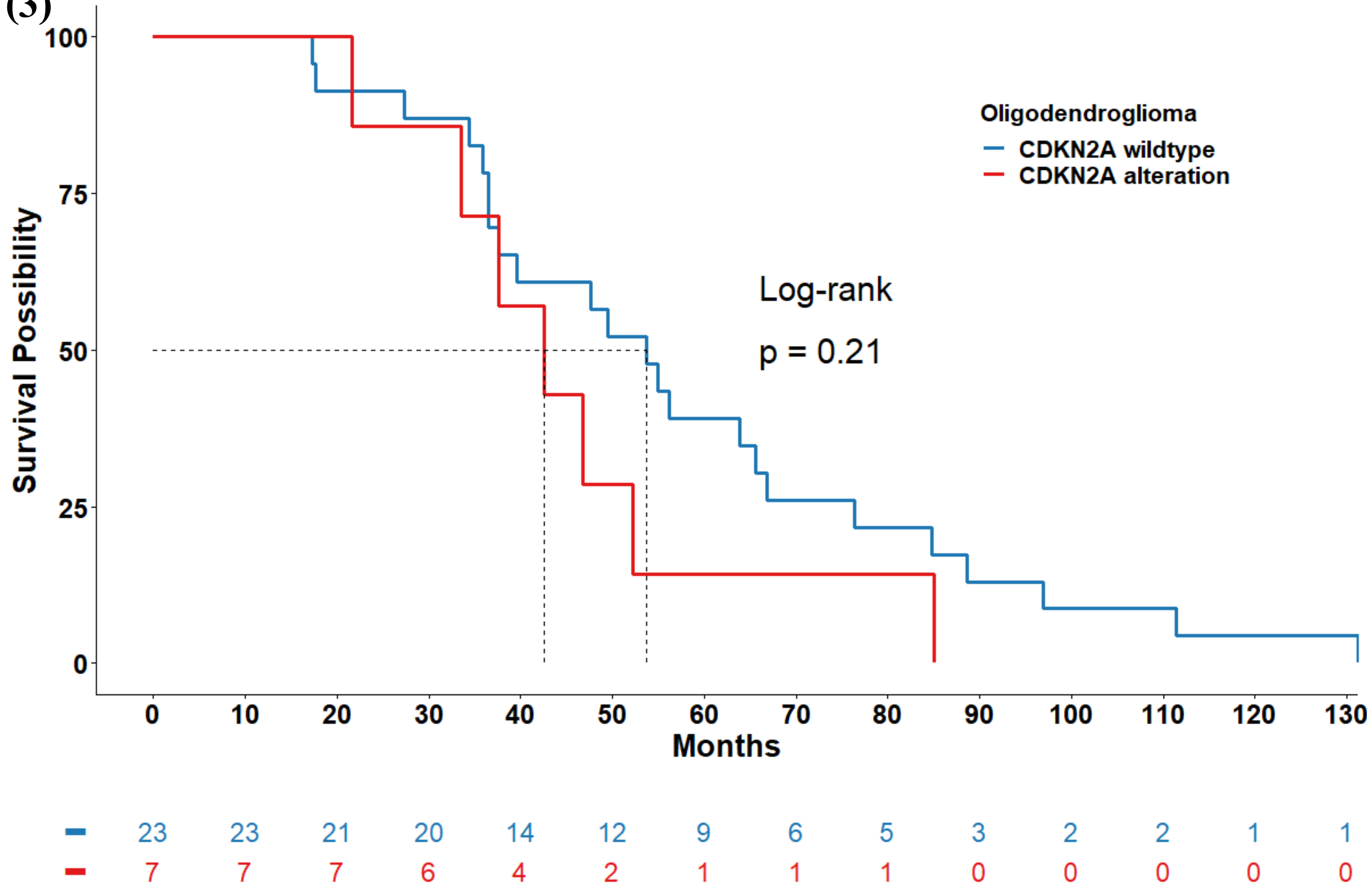

Survival Possibility
100
75
50
25
0
Oligodendroglioma
CDKN2A wildtype
CDKN2A alteration
Log-rank
p = 0.21
0 10 20 30 40 50 60 70 80 90 100 110 120 130
Months
23 23 21 20 14 12 9 6 5 3 2 2 1 1
7 7 7 6 4 2 1 1 1 0 0 0 0 0

(4)

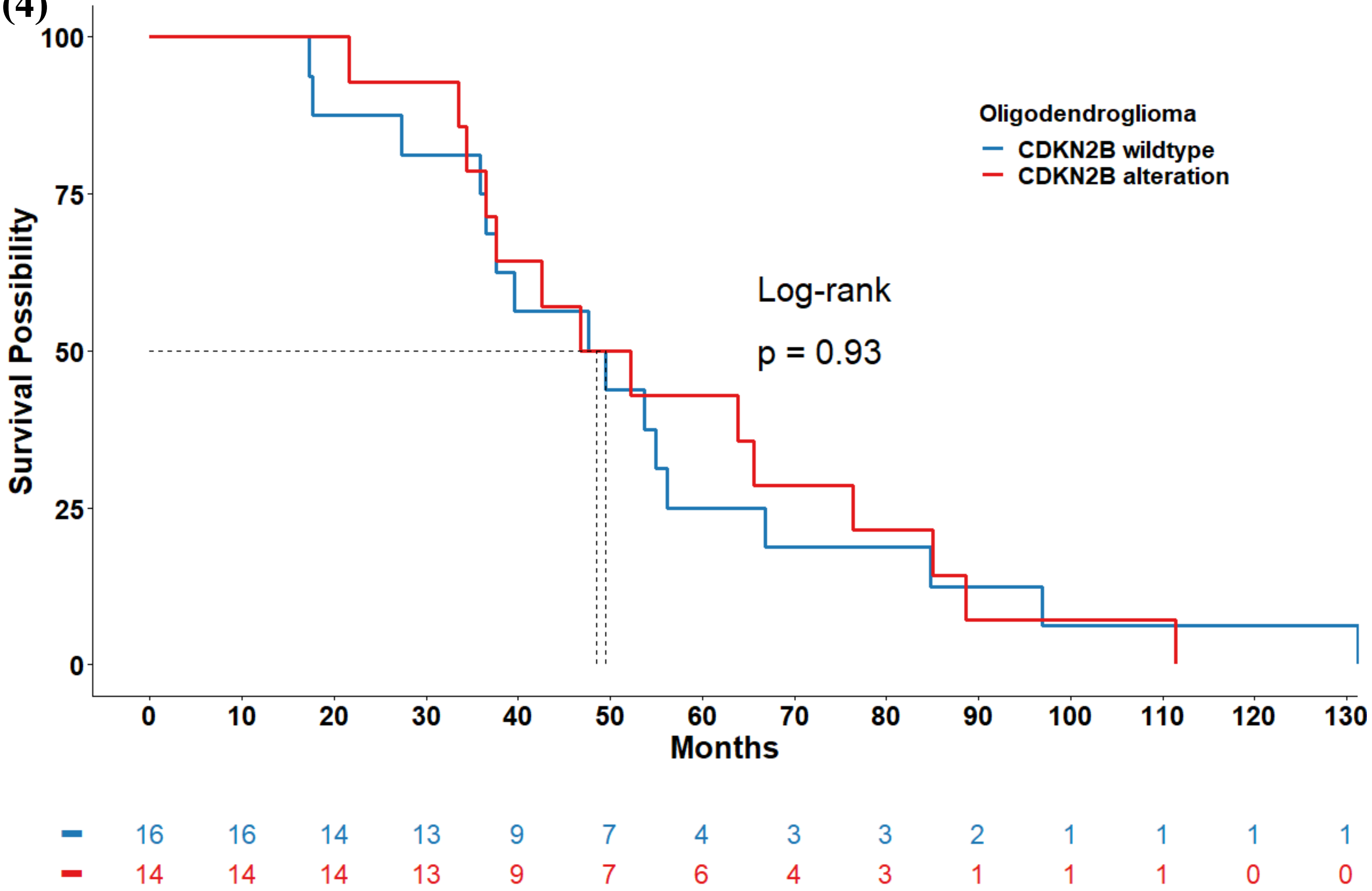

Survival Possibility
100
75
50
25
0
0 10 20 30 40 50 60 70 80 90 100 110 120 130
Months
Oligodendroglioma
CDKN2B wildtype
CDKN2B alteration
Log-rank
p = 0.93
16 16 14 13 9 7 4 3 3 2 1 1 1 1
14 14 14 13 9 7 6 4 3 1 1 1 0 0

(5)

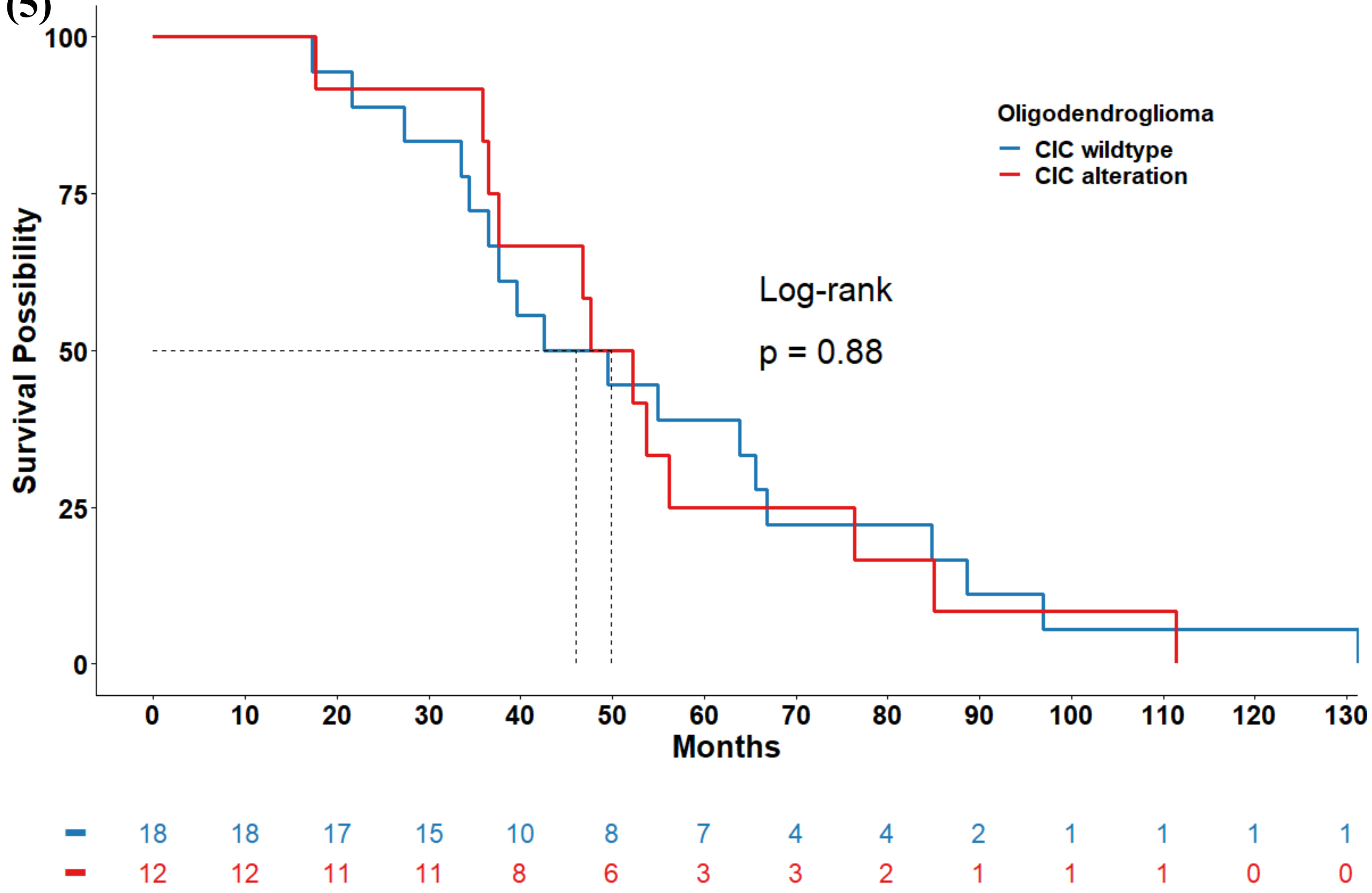
Survival Possibility
100
75
50
25
0
0
10
20
30
40
50
60
70
80
90
100
110
120
130
Months
Oligodendroglioma
CIC wildtype
CIC alteration
Log-rank
p = 0.88
18 18 17 15 10 8 7 4 4 2 1 1 1 1
12 12 11 11 8 6 3 3 2 1 1 1 0 0

(6)

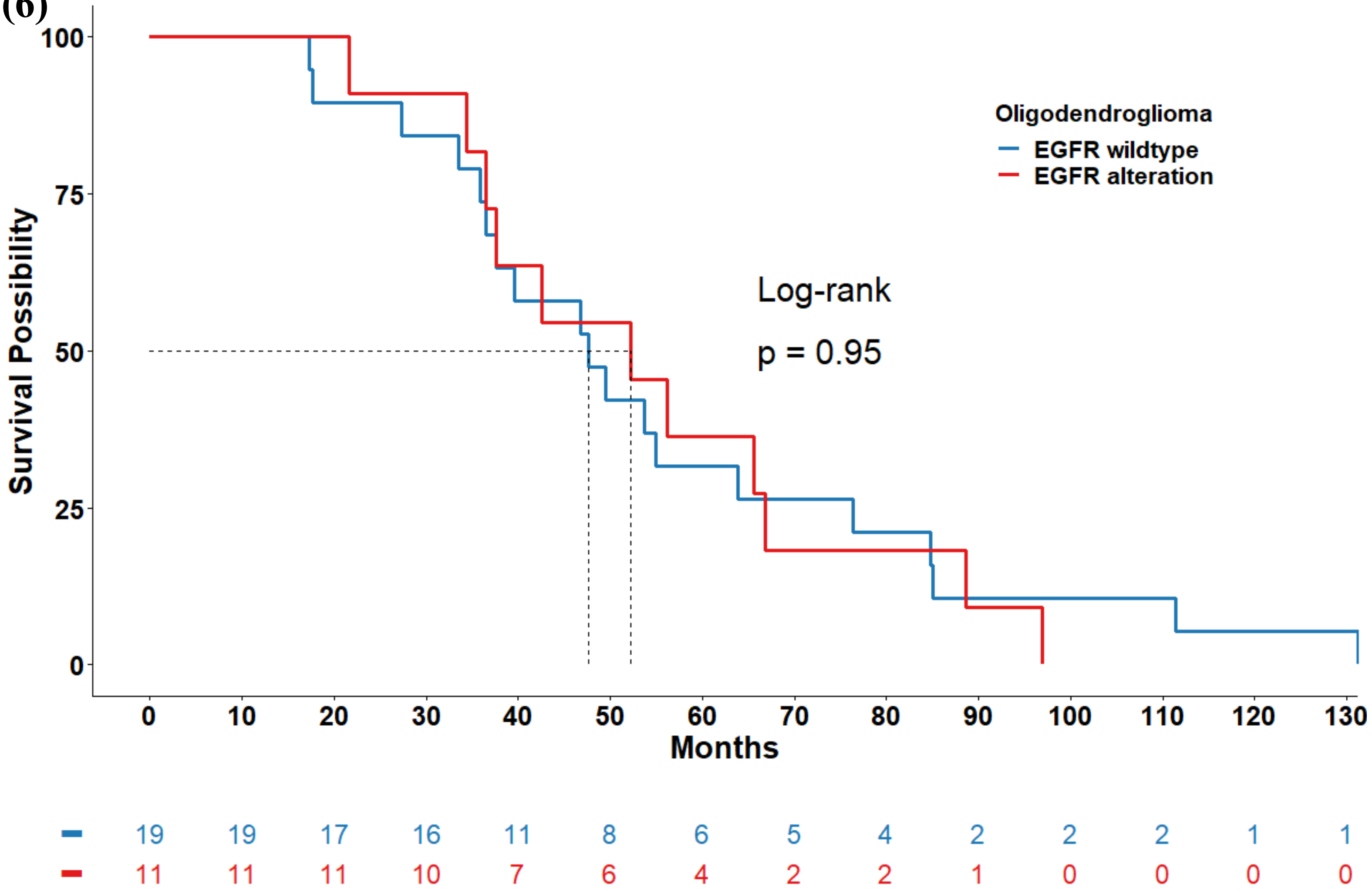
Oligodendroglioma
EGFR wildtype
EGFR alteration
Log-rank
p = 0.95
Survival Possibility
100
75
50
25
0
Months
0 10 20 30 40 50 60 70 80 90 100 110 120 130
19 19 17 16 11 8 6 5 4 2 2 2 1 1
11 11 11 10 7 6 4 2 2 1 0 0 0 0

(7)

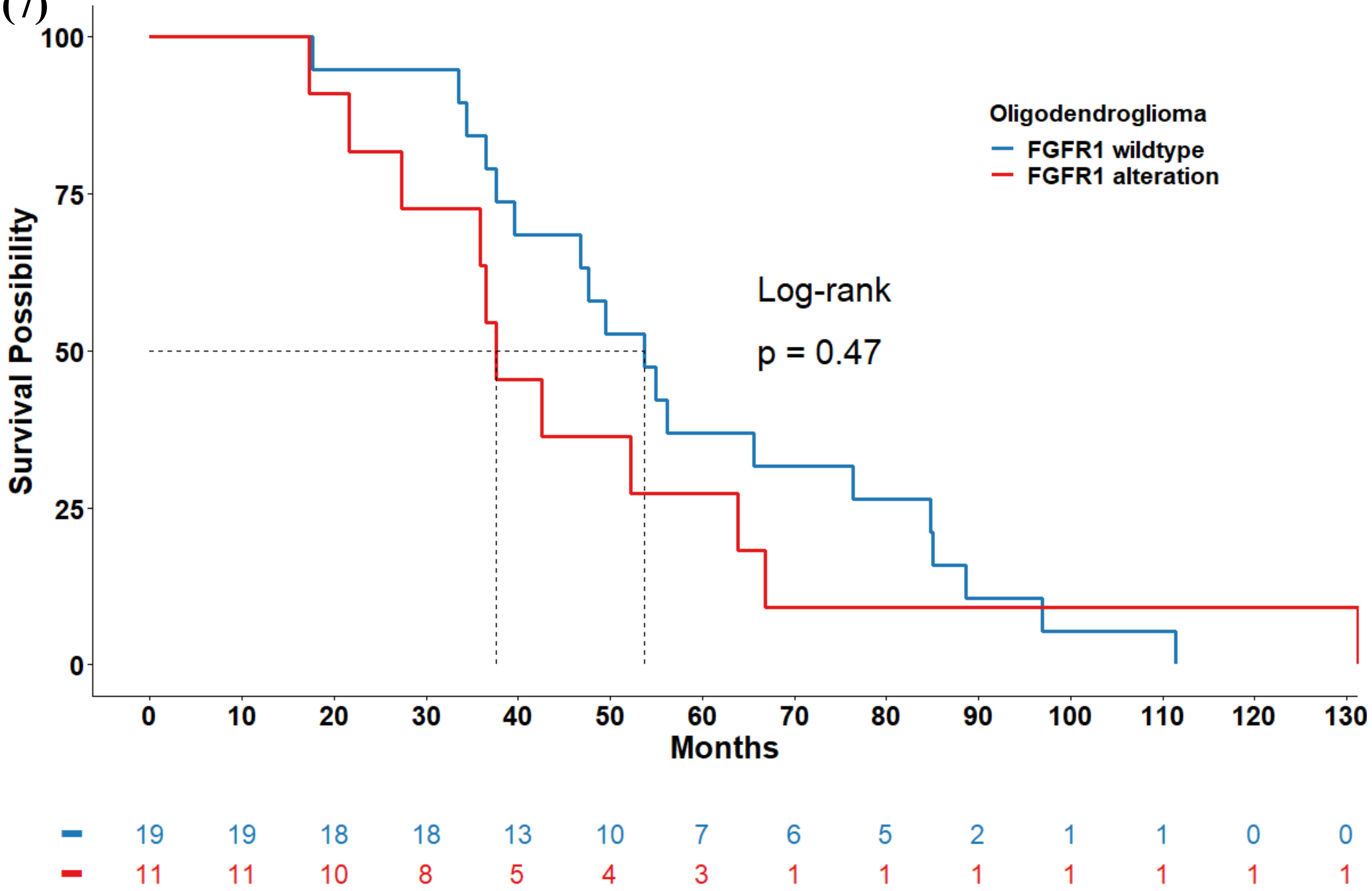
100
75
50
25
0
Survival Possibility
Oligodendroglioma
FGFR1 wildtype
FGFR1 alteration
Log-rank
p = 0.47
0
10
20
30
40
50
60
70
80
90
100
110
120
130
Months
19 19 18 18 13 10 7 6 5 2 1 1 0 0
11 11 10 8 5 4 3 1 1 1 1 1 1 1

(8)

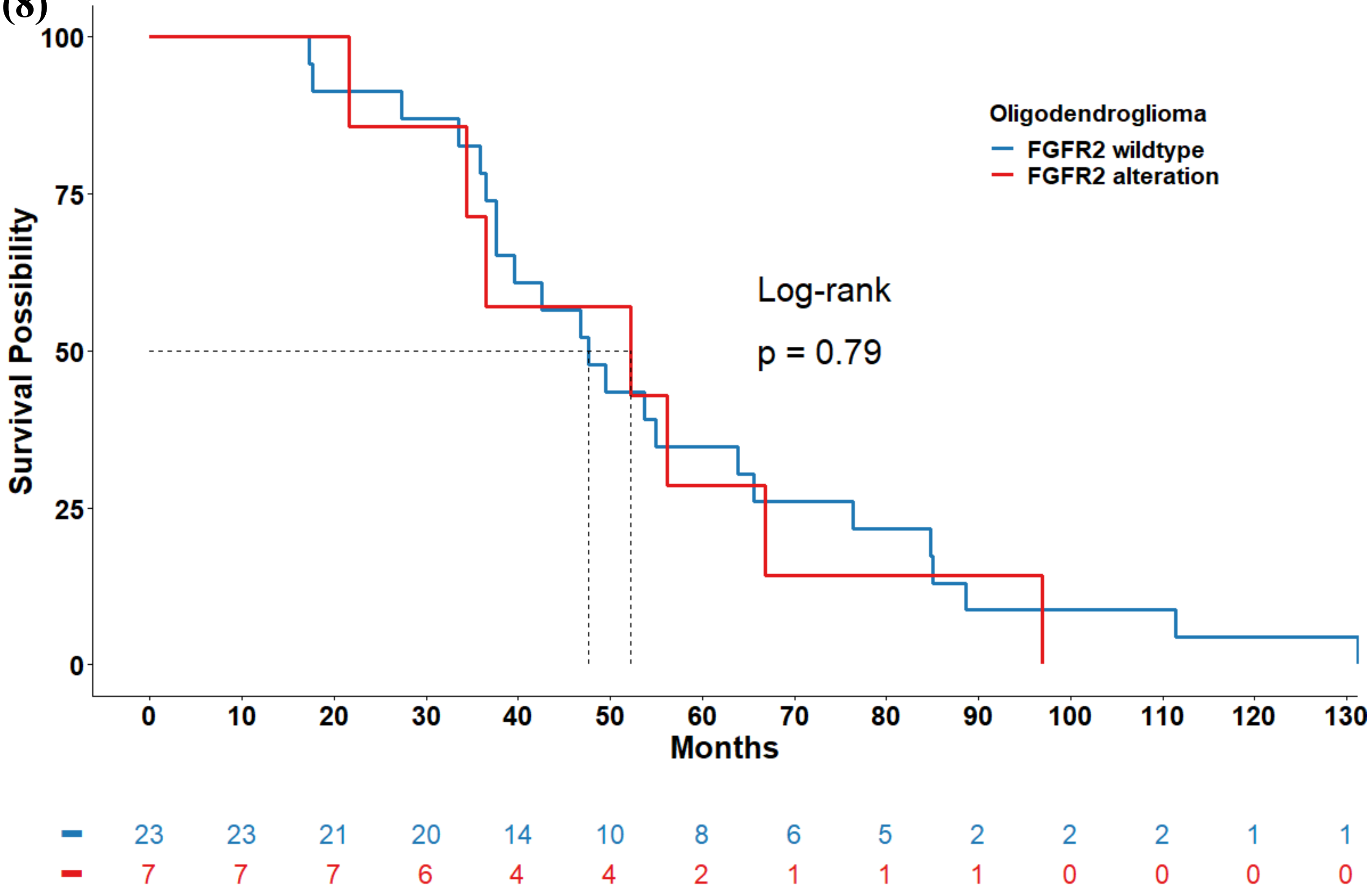

Survival Possibility
100
75
50
25
0
0 10 20 30 40 50 60 70 80 90 100 110 120 130
Months
Oligodendroglioma
FGFR2 wildtype
FGFR2 alteration
Log-rank
p = 0.79
23 23 21 20 14 10 8 6 5 2 2 2 1 1
7 7 7 6 4 4 2 1 1 1 0 0 0 0

(9)

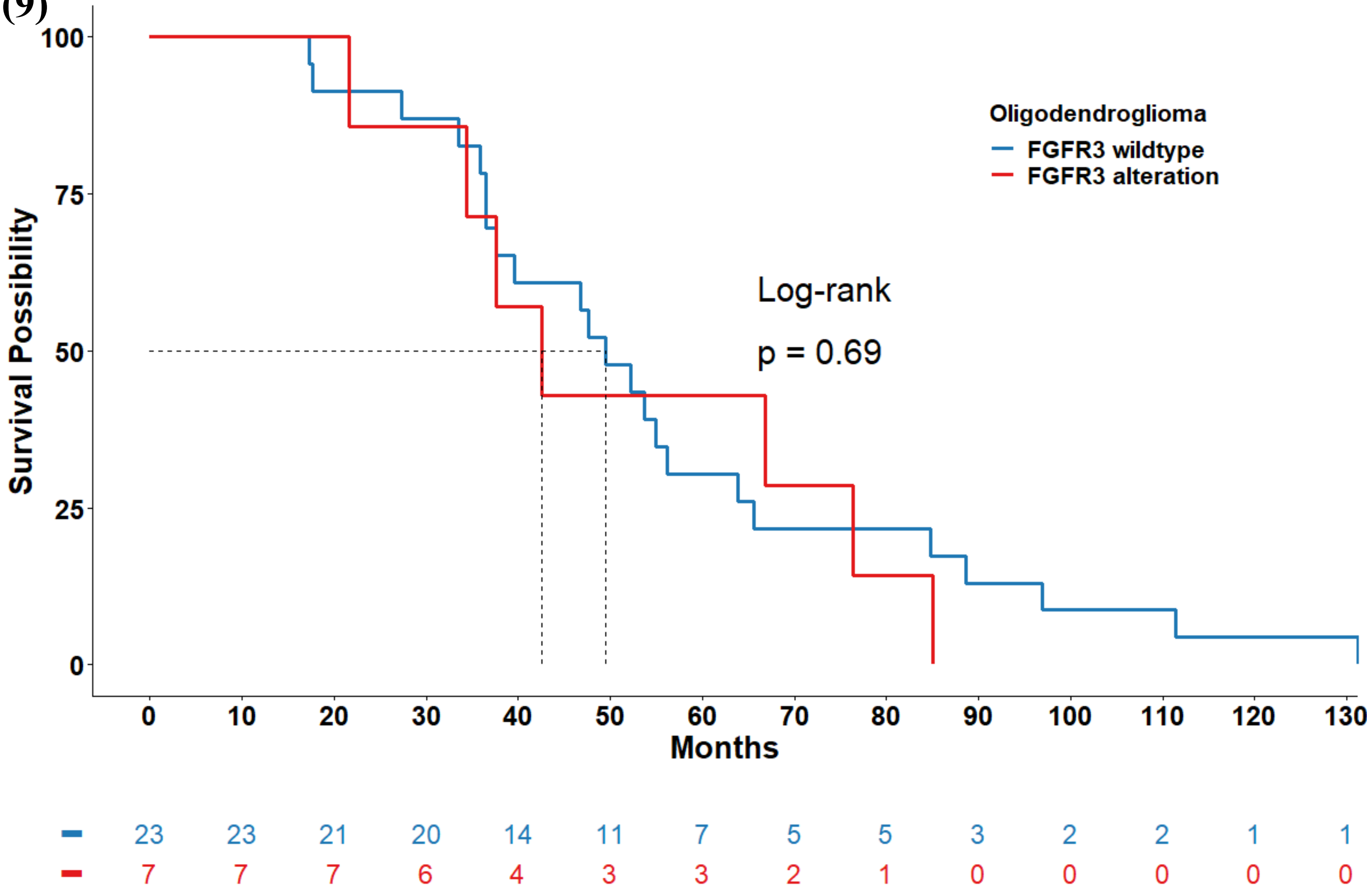

Survival Possibility
100
75
50
25
0
0
10
20
30
40
50
60
70
80
90
100
110
120
130
Months
Oligodendroglioma
FGFR3 wildtype
FGFR3 alteration
Log-rank
p = 0.69
23 23 21 20 14 11 7 5 5 3 2 2 1 1
7 7 7 6 4 3 3 2 1 0 0 0 0 0

(10)

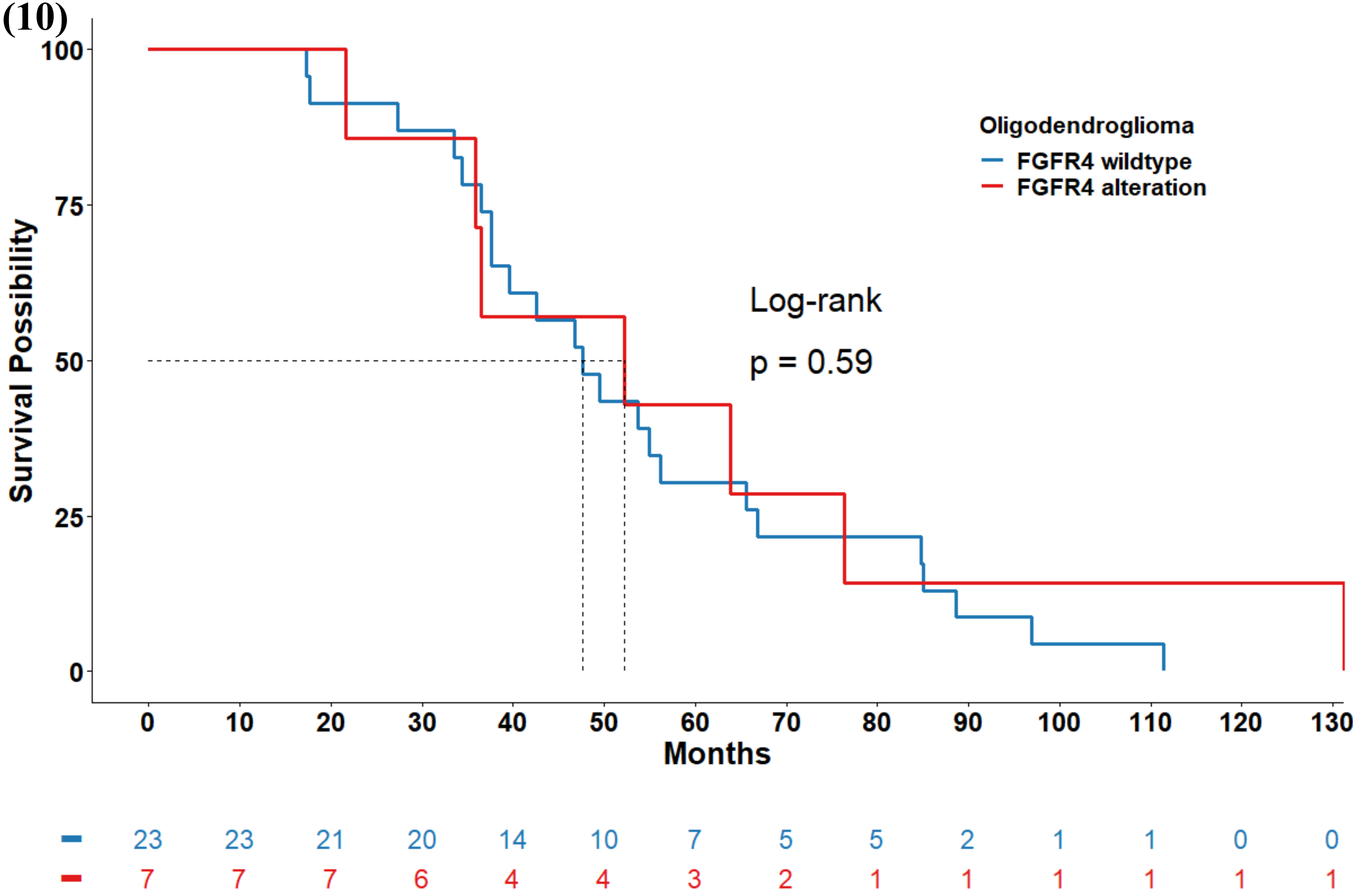
100
75
50
25
0
Survival Possibility
0 10 20 30 40 50 60 70 80 90 100 110 120 130
Months
Oligodendroglioma
FGFR4 wildtype
FGFR4 alteration
Log-rank
p = 0.59
23 23 21 20 14 10 7 5 5 2 1 1 0 0
7 7 7 6 4 4 3 2 1 1 1 1 1 1

(11)

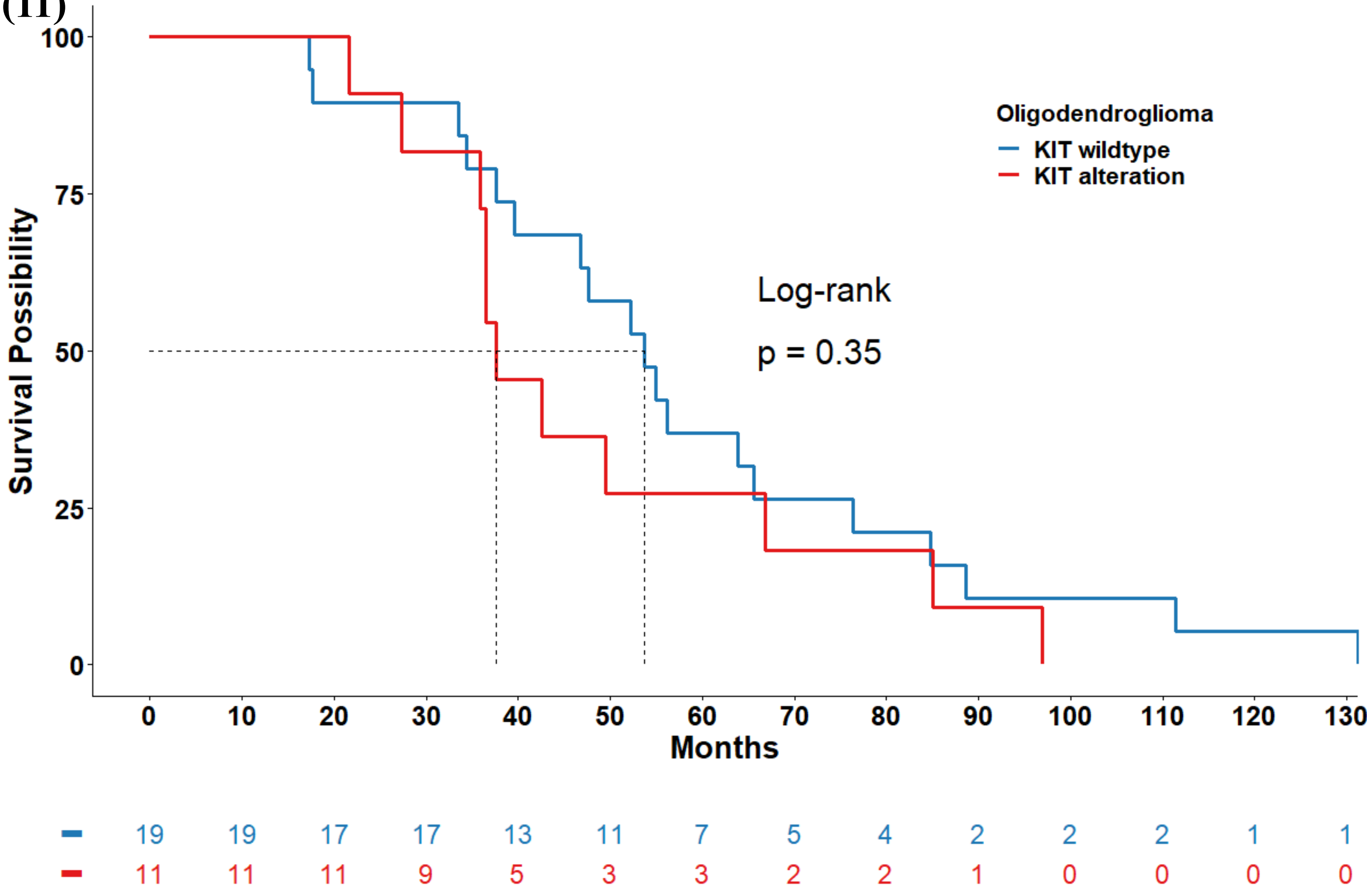

100
75
50
25
0
Survival Possibility
0
10
20
30
40
50
60
70
80
90
100
110
120
130
Months
Oligodendroglioma
KIT wildtype
KIT alteration
Log-rank
p = 0.35
19 19 17 17 13 11 7 5 4 2 2 2 1 1
11 11 11 9 5 3 3 2 2 1 0 0 0 0

(12)

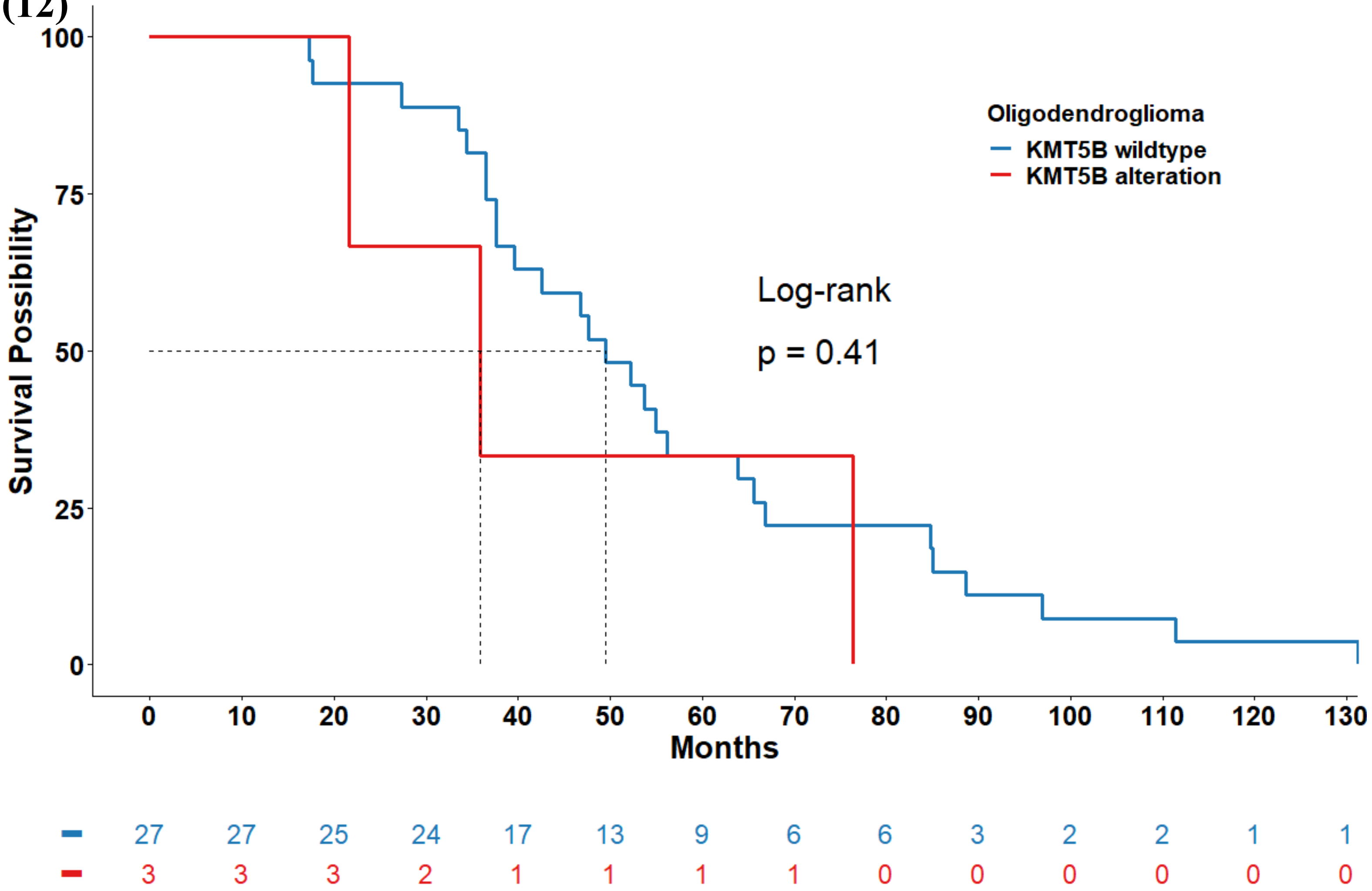

Survival Possibility
100
75
50
25
0
Oligodendroglioma
KMT5B wildtype
KMT5B alteration
Log-rank
p = 0.41
0 10 20 30 40 50 60 70 80 90 100 110 120 130
Months
27 27 25 24 17 13 9 6 6 3 2 2 1 1
3 3 3 2 1 1 1 1 0 0 0 0 0 0

(13)

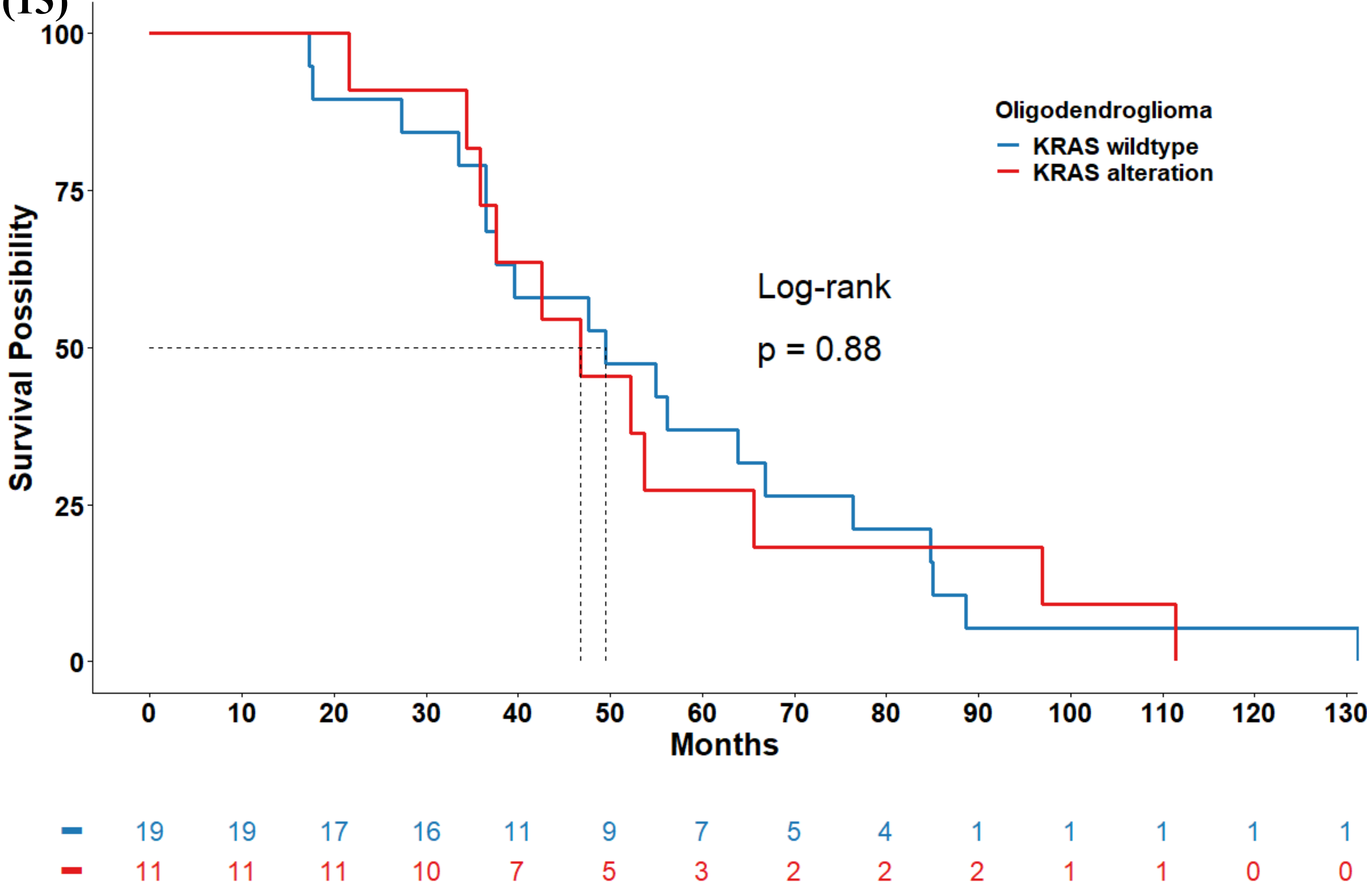

Survival Possibility
100
75
50
25
0
Oligodendroglioma
KRAS wildtype
KRAS alteration
Log-rank
p = 0.88
0 10 20 30 40 50 60 70 80 90 100 110 120 130
Months
19 19 17 16 11 9 7 5 4 1 1 1 1 1
11 11 11 10 7 5 3 2 2 2 1 1 0 0

(14)

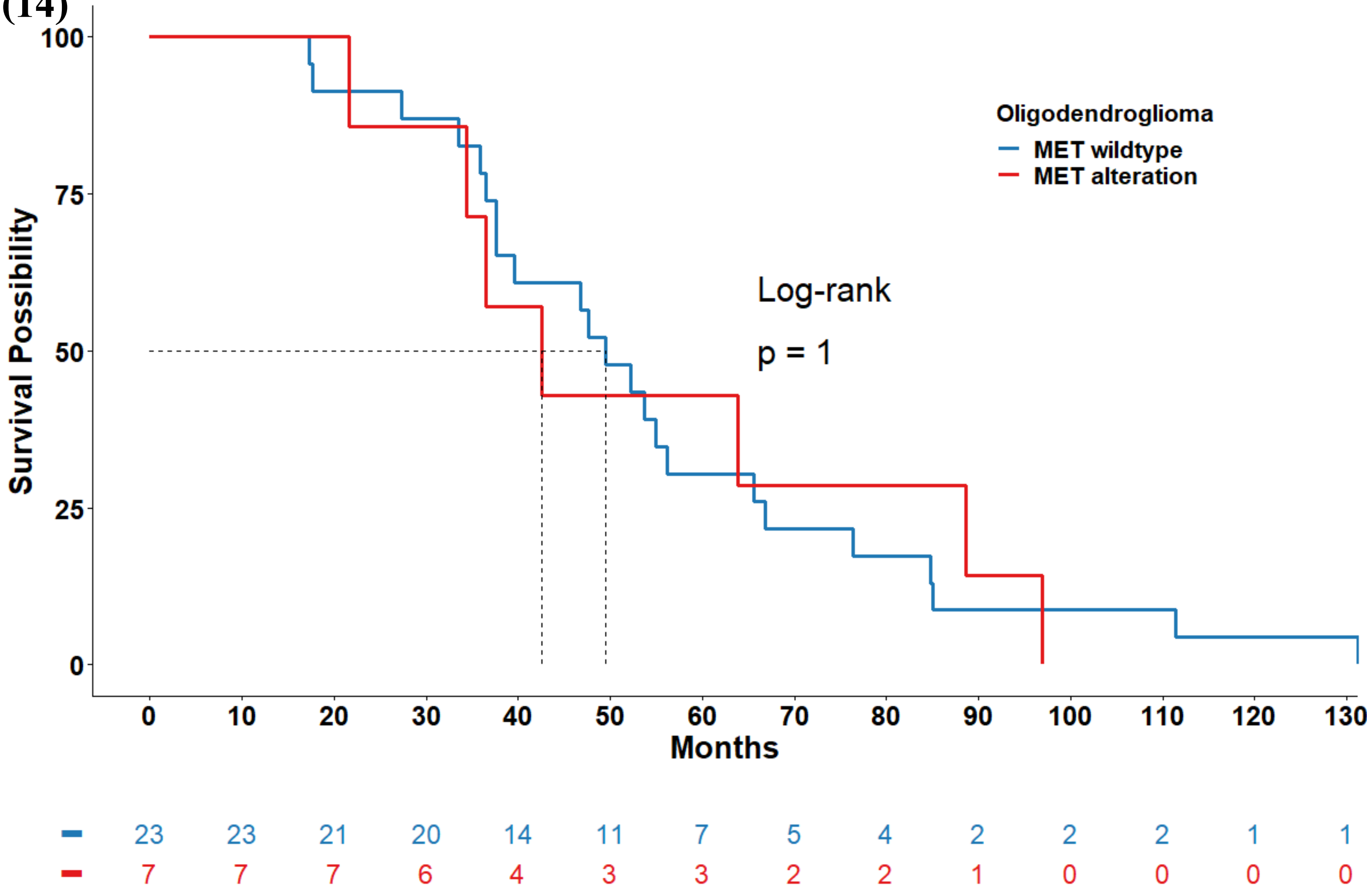

Oligodendroglioma
MET wildtype
MET alteration
Log-rank
p = 1
Survival Possibility
100
75
50
25
0
0
10
20
30
40
50
60
70
80
90
100
110
120
130
Months
23 23 21 20 14 11 7 5 4 2 2 2 1 1
7 7 7 6 4 3 3 2 2 1 0 0 0 0

(15)

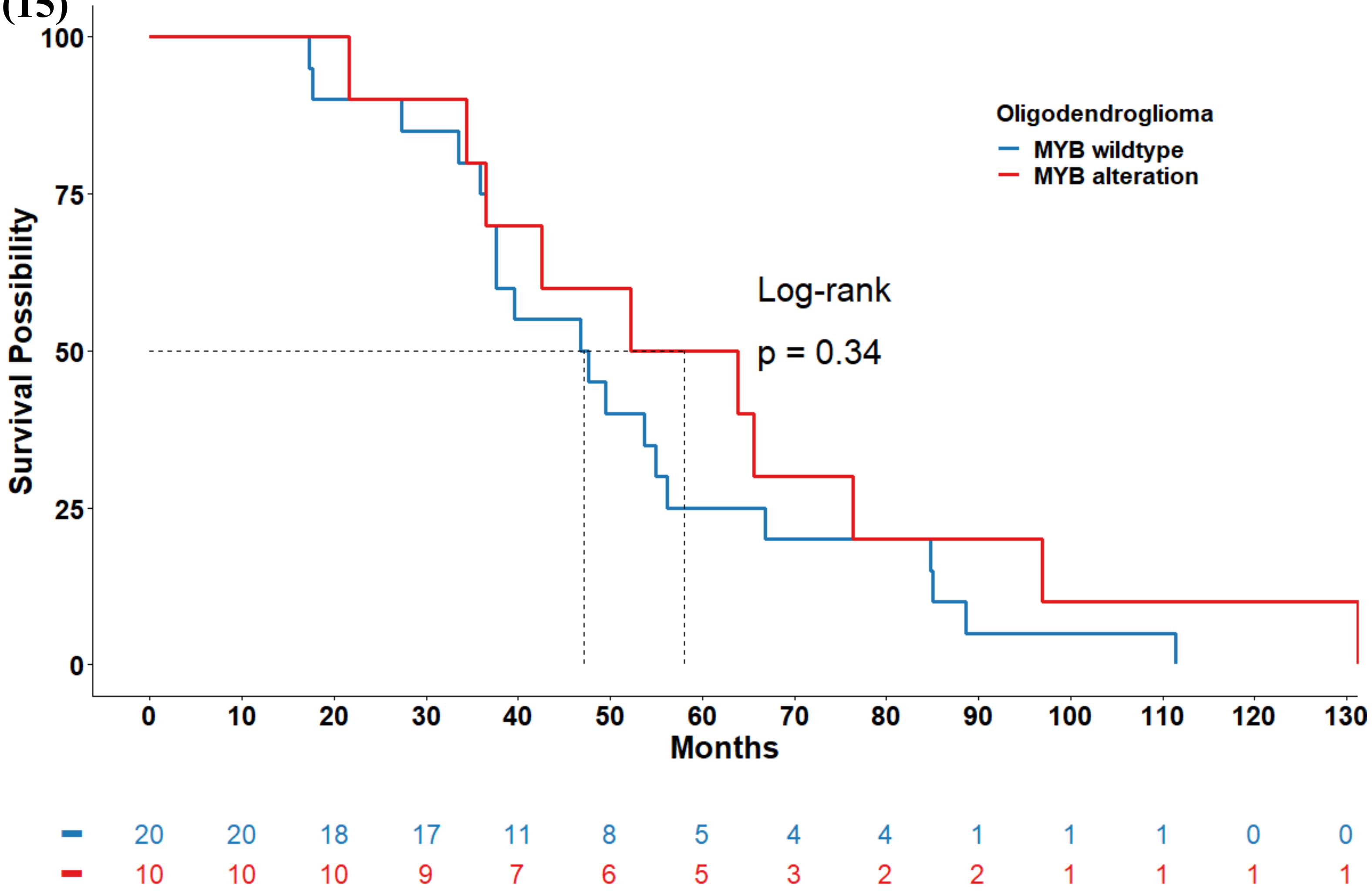
Survival Possibility
100
75
50
25
0
0 10 20 30 40 50 60 70 80 90 100 110 120 130
Months
Oligodendroglioma
MYB wildtype
MYB alteration
Log-rank
p = 0.34
20 20 18 17 11 8 5 4 4 1 1 1 0 0
10 10 10 9 7 6 5 3 2 2 1 1 1 1

(16)

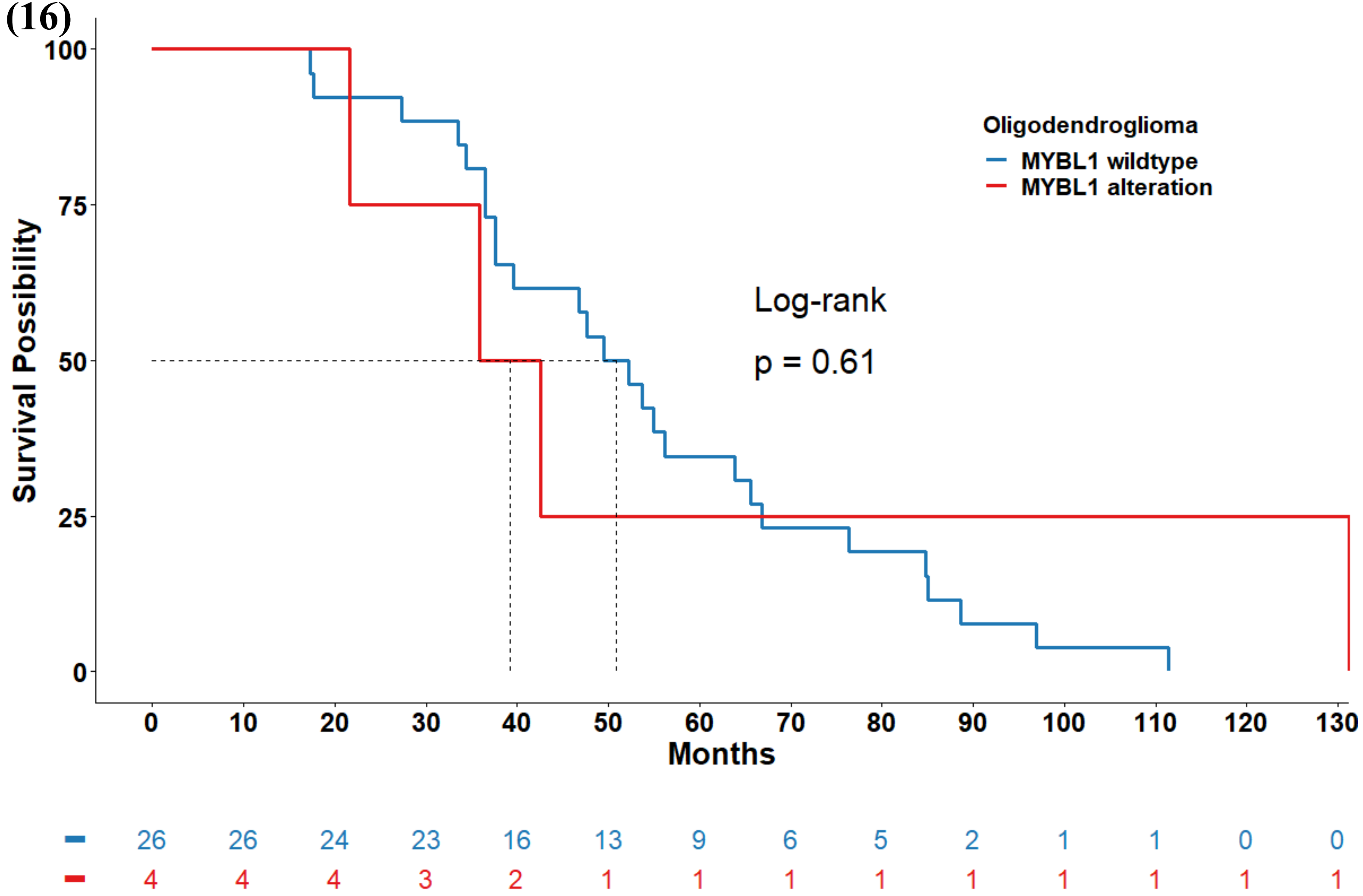

Oligodendroglioma
MYBL1 wildtype
MYBL1 alteration
Log-rank
p = 0.61
Survival Possibility
100
75
50
25
0
Months
0 10 20 30 40 50 60 70 80 90 100 110 120 130
26 26 24 23 16 13 9 6 5 2 1 1 0 0
4 4 4 3 2 1 1 1 1 1 1 1 1 1

(17)

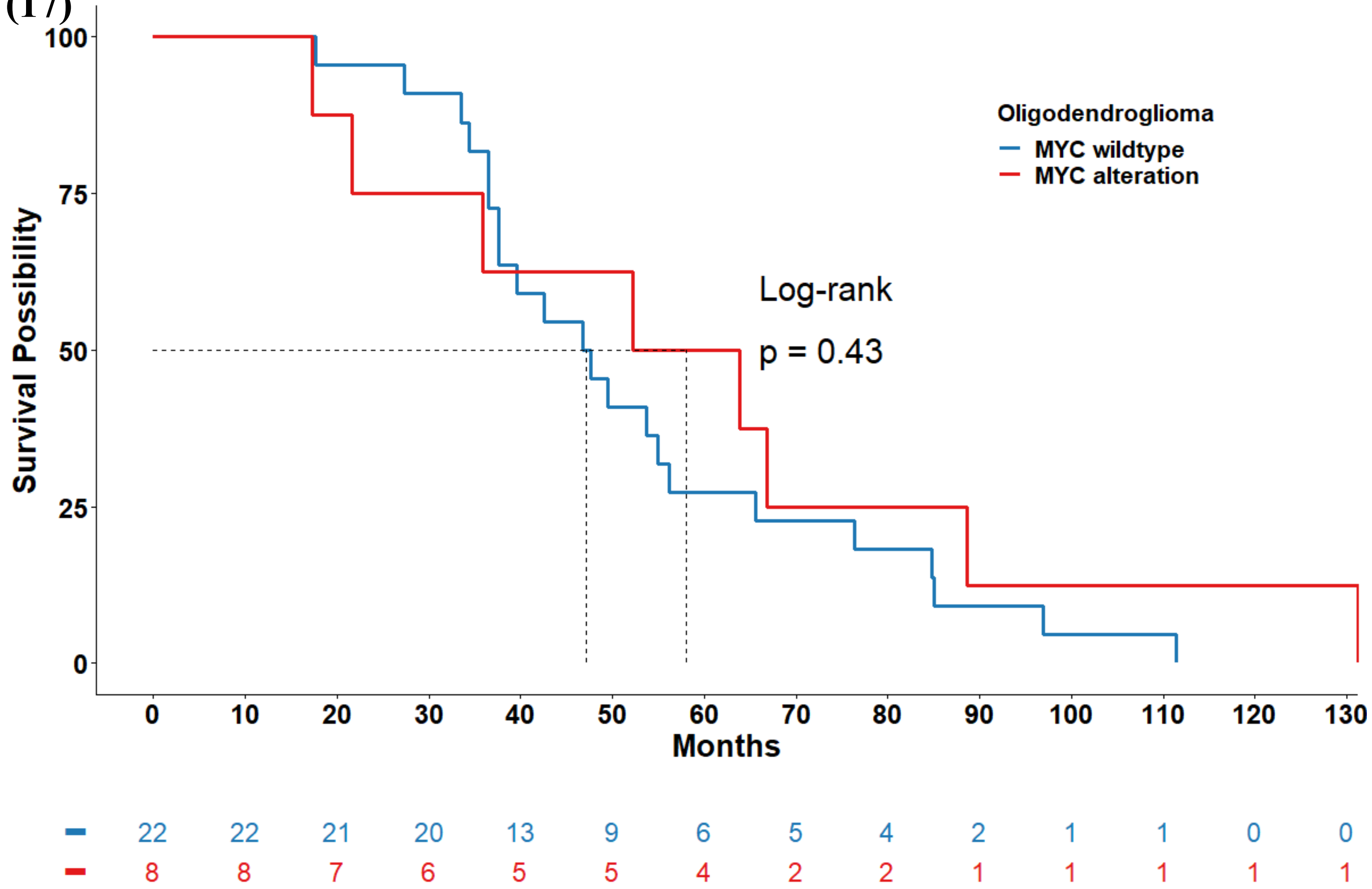

Survival Possibility
100
75
50
25
0
0 10 20 30 40 50 60 70 80 90 100 110 120 130
Months
Oligodendroglioma
MYC wildtype
MYC alteration
Log-rank
p = 0.43
22 22 21 20 13 9 6 5 4 2 1 1 0 0
8 8 7 6 5 5 4 2 2 1 1 1 1 1

(18)

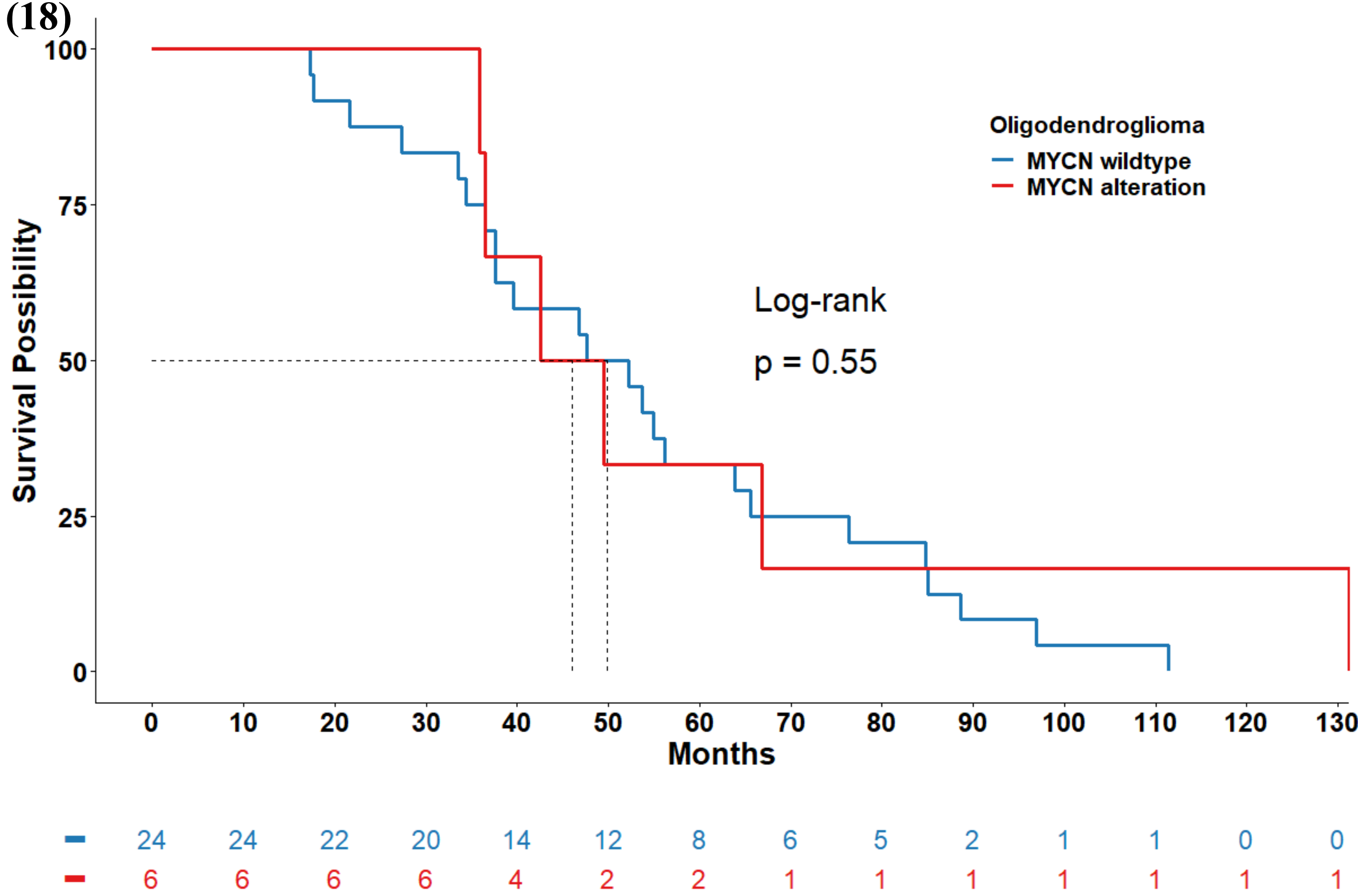
Survival Possibility
100
75
50
25
0
0
10
20
30
40
50
60
70
80
90
100
110
120
130
Months
Oligodendroglioma
MYCN wildtype
MYCN alteration
Log-rank
p = 0.55
24 24 22 20 14 12 8 6 5 2 1 1 0 0
6 6 6 6 4 2 2 1 1 1 1 1 1 1

(19)

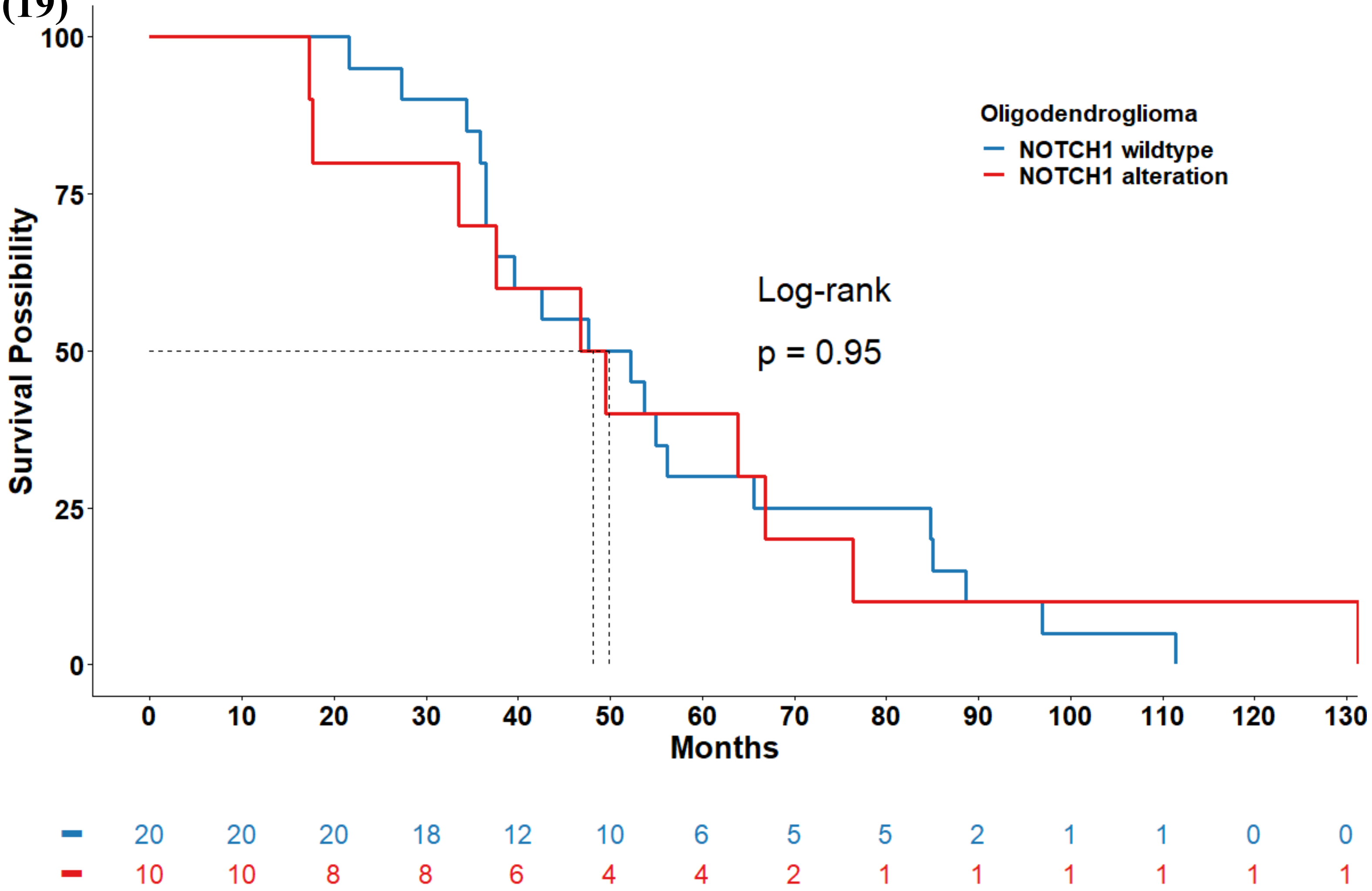

Oligodendroglioma
NOTCH1 wildtype
NOTCH1 alteration
Log-rank
p = 0.95
Survival Possibility
100
75
50
25
0
Months
0 10 20 30 40 50 60 70 80 90 100 110 120 130
20 20 20 18 12 10 6 5 5 2 1 1 0 0
10 10 8 8 6 4 4 2 1 1 1 1 1 1

(20)

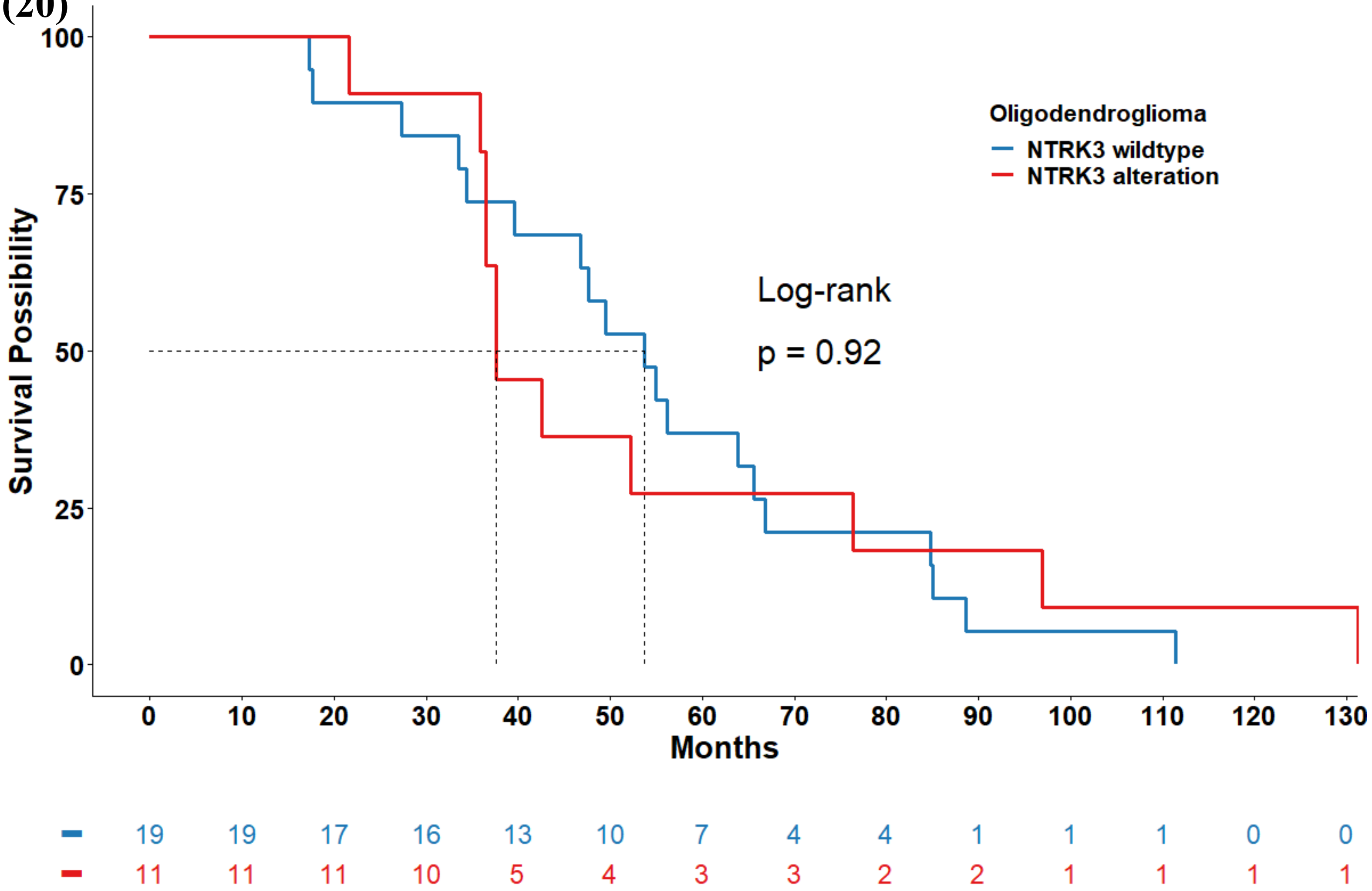
Survival Possibility
100
75
50
25
0
Oligodendroglioma
NTRK3 wildtype
NTRK3 alteration
Log-rank
p = 0.92
0
10
20
30
40
50
60
70
80
90
100
110
120
130
Months
19 19 17 16 13 10 7 4 4 1 1 1 0 0
11 11 11 10 5 4 3 3 2 2 1 1 1 1

(21)

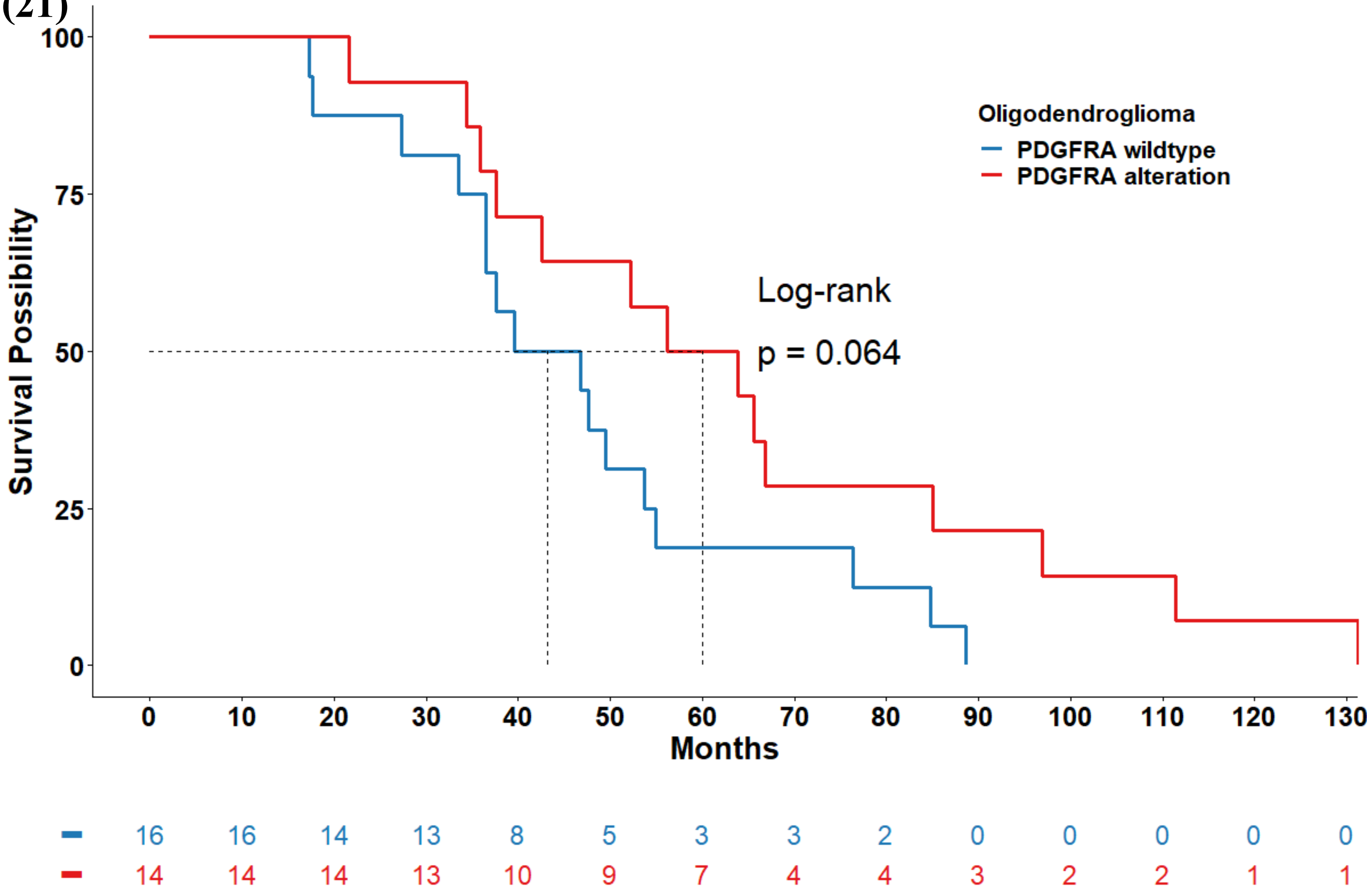
Survival Possibility
100
75
50
25
0
Oligodendroglioma
PDGFRA wildtype
PDGFRA alteration
Log-rank
p = 0.064
0
10
20
30
40
50
60
70
80
90
100
110
120
130
Months
16 16 14 13 8 5 3 3 2 0 0 0 0 0
14 14 14 13 10 9 7 4 4 3 2 2 1 1

(22)

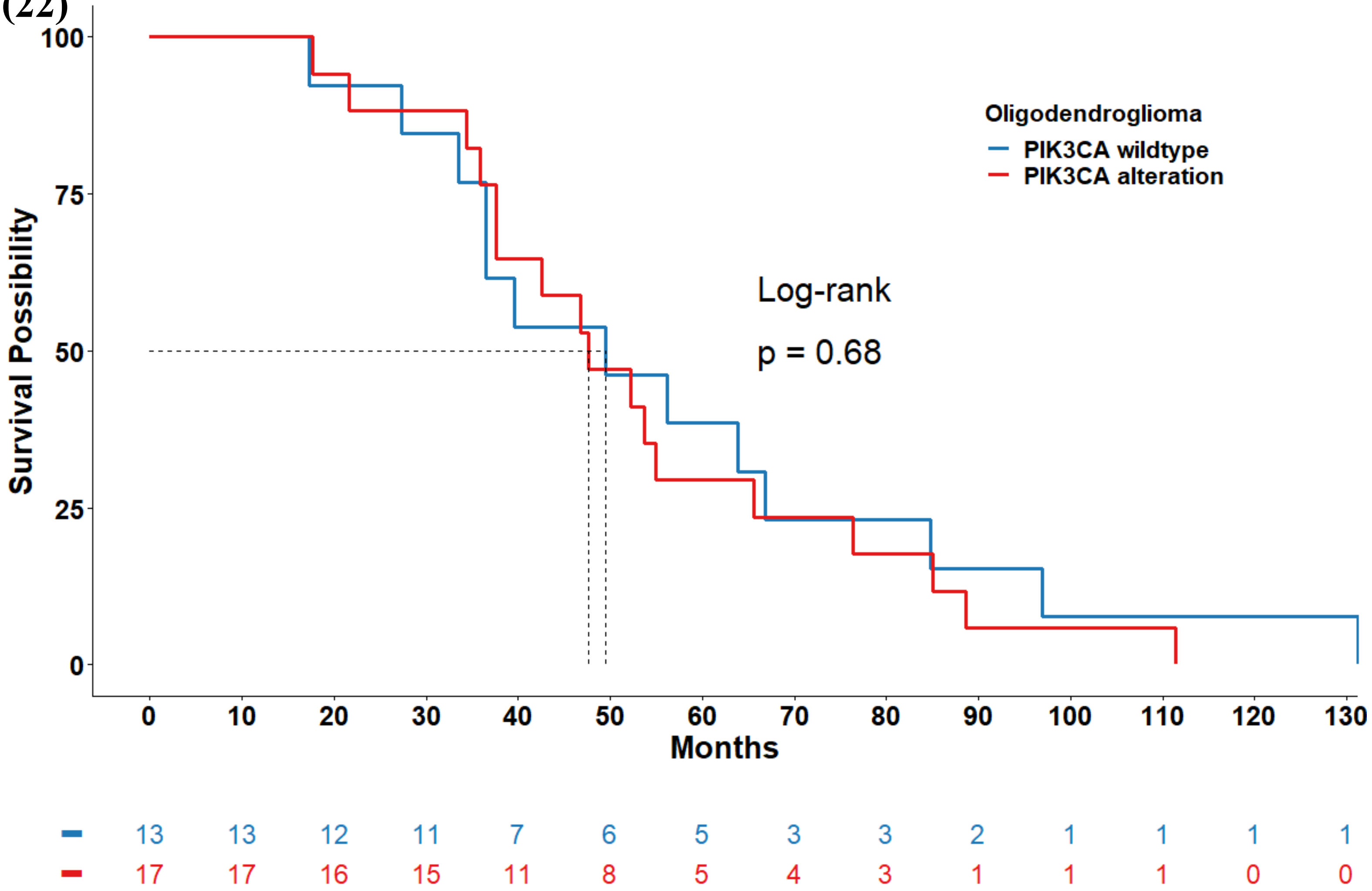
Survival Possibility
100
75
50
25
0
Months
0 10 20 30 40 50 60 70 80 90 100 110 120 130
Oligodendroglioma
PIK3CA wildtype
PIK3CA alteration
Log-rank
p = 0.68
13 13 12 11 7 6 5 3 3 2 1 1 1 1
17 17 16 15 11 8 5 4 3 1 1 1 0 0

(23)

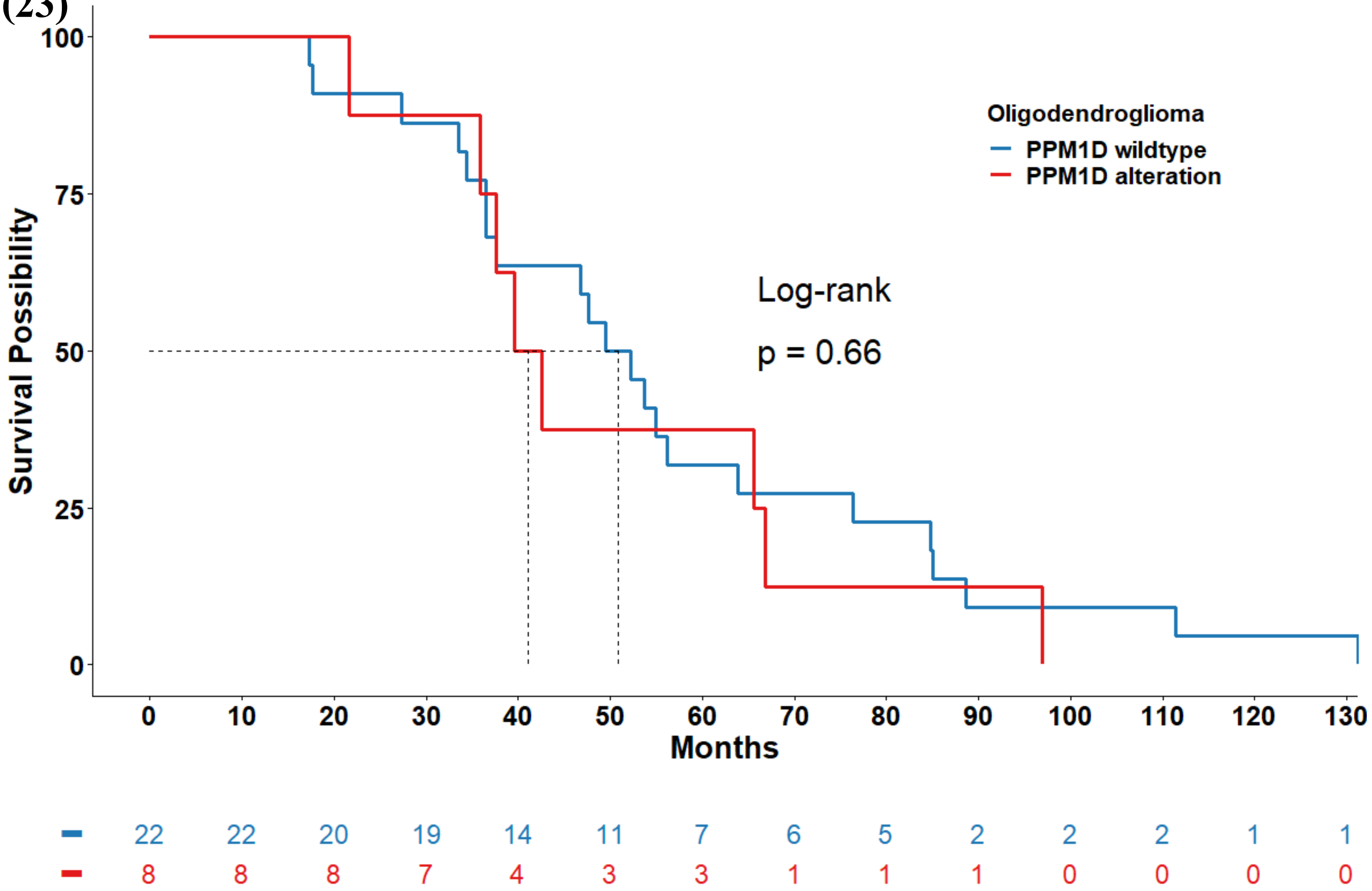

Survival Possibility
100
75
50
25
0
Oligodendroglioma
PPM1D wildtype
PPM1D alteration
Log-rank
p = 0.66
0
10
20
30
40
50
60
70
80
90
100
110
120
130
Months
22 22 20 19 14 11 7 6 5 2 2 2 1 1
8 8 8 7 4 3 3 1 1 1 0 0 0 0

(24)

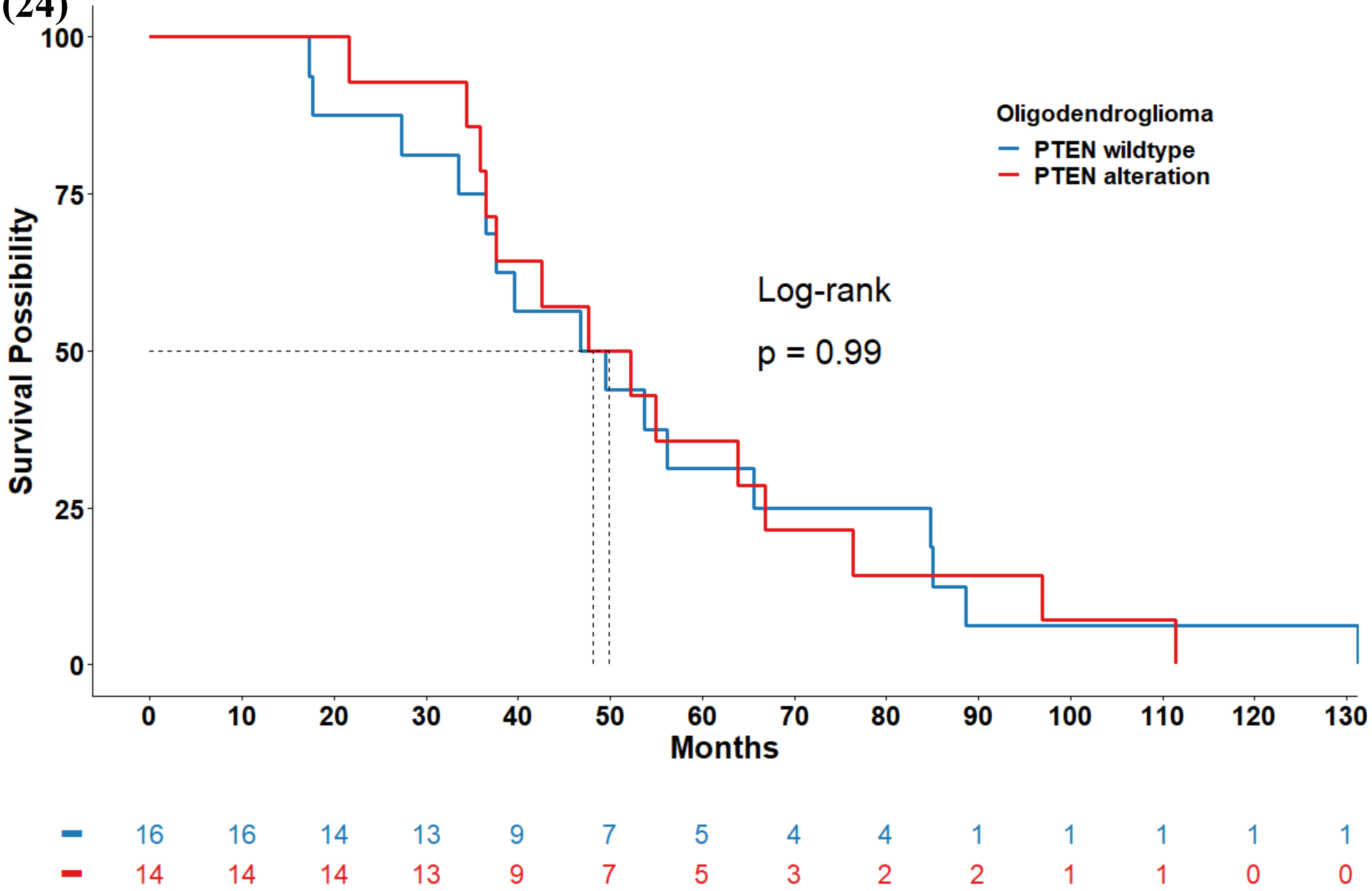
Survival Possibility
100
75
50
25
0
Oligodendroglioma
PTEN wildtype
PTEN alteration
Log-rank
p = 0.99
0
10
20
30
40
50
60
70
80
90
100
110
120
130
Months
16 16 14 13 9 7 5 4 4 1 1 1 1 1
14 14 14 13 9 7 5 3 2 2 1 1 0 0

(25)

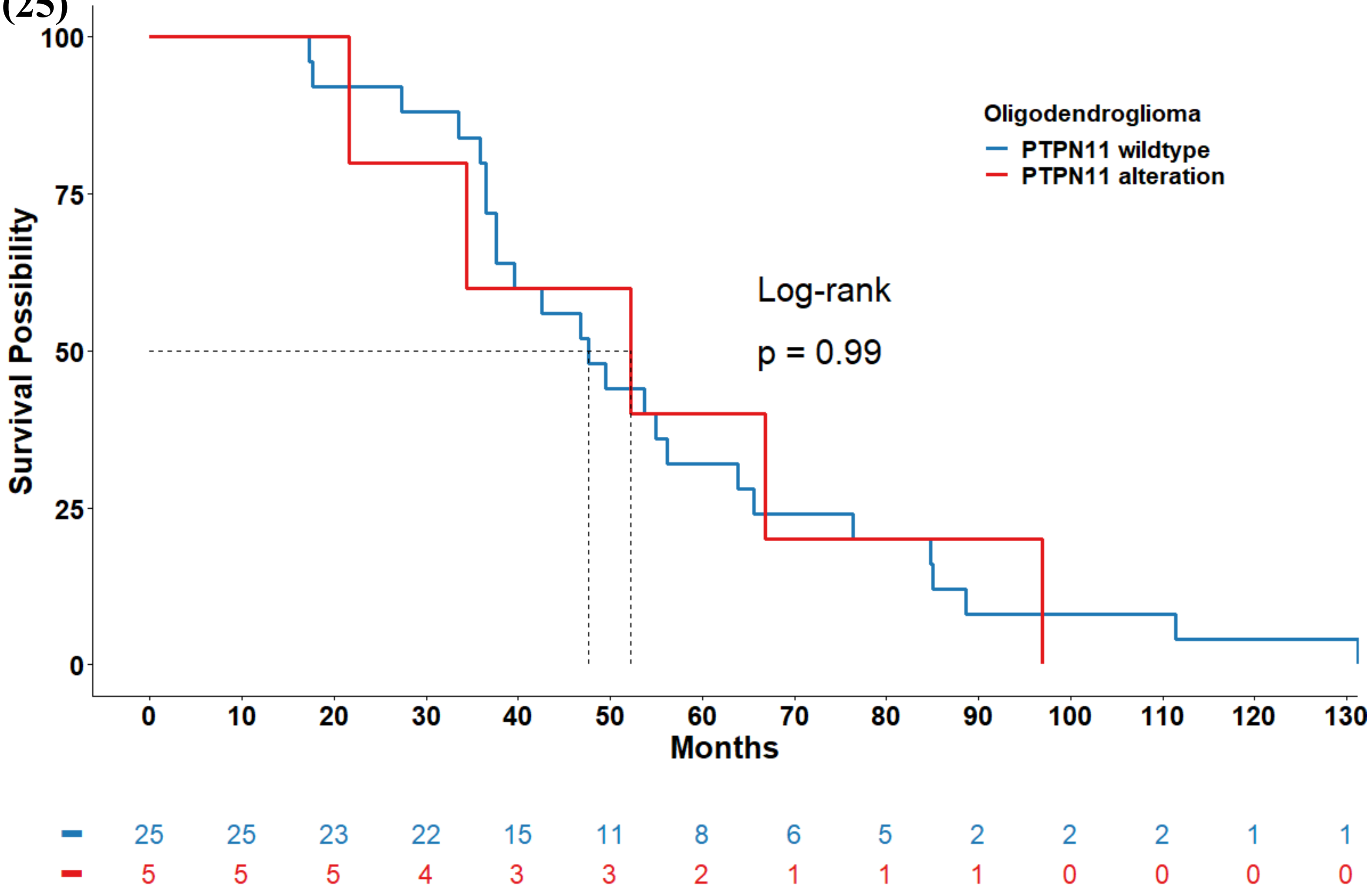

Oligodendroglioma
PTPN11 wildtype
PTPN11 alteration
Log-rank
p = 0.99
Survival Possibility
100
75
50
25
0
0 10 20 30 40 50 60 70 80 90 100 110 120 130
Months
25 25 23 22 15 11 8 6 5 2 2 2 1 1
5 5 5 4 3 3 2 1 1 1 0 0 0 0

(26)

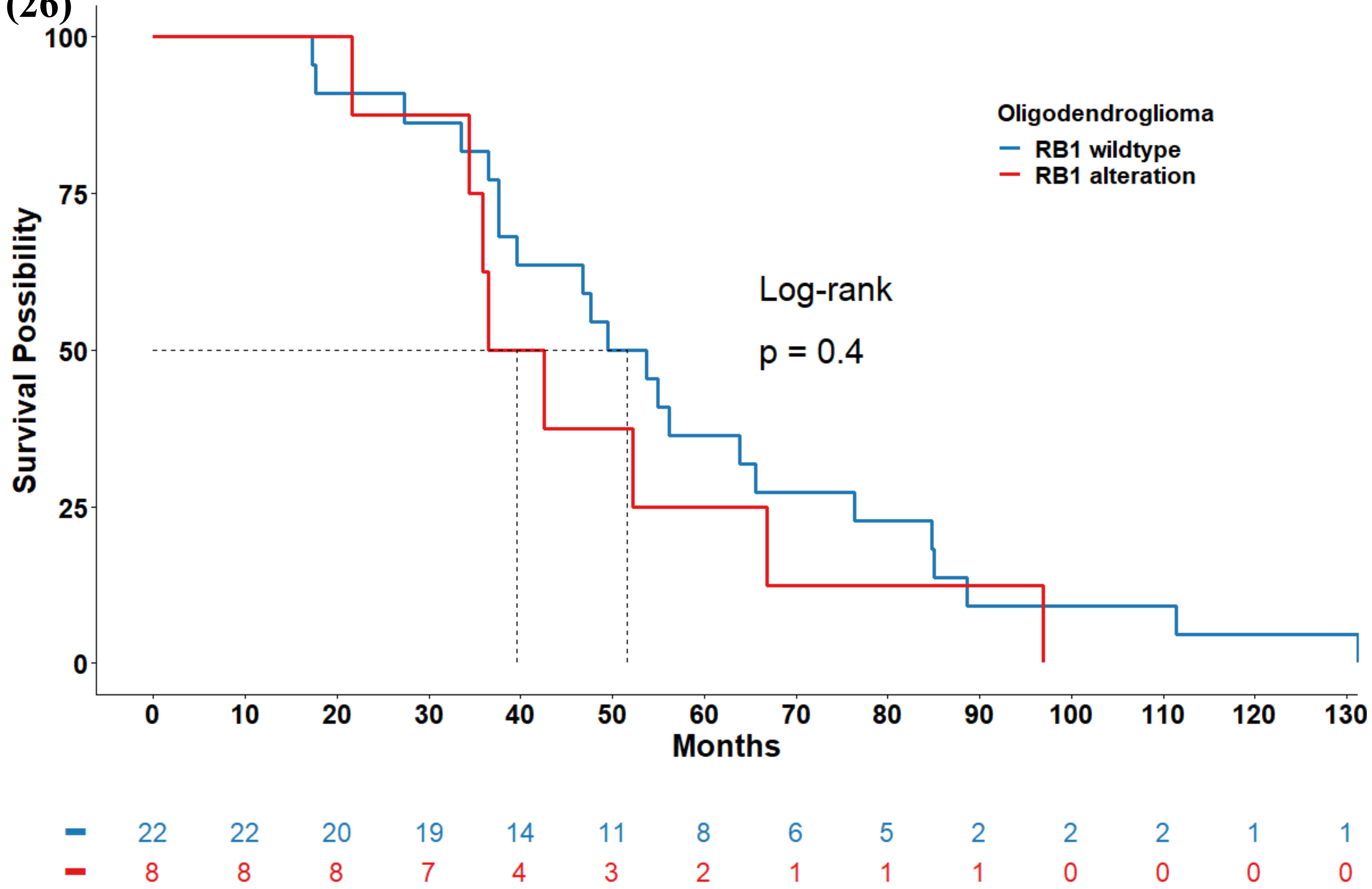
Oligodendroglioma
RB1 wildtype
RB1 alteration
Log-rank
p = 0.4
Survival Possibility
100
75
50
25
0
Months
0 10 20 30 40 50 60 70 80 90 100 110 120 130
22 22 20 19 14 11 8 6 5 2 2 2 1 1
8 8 8 7 4 3 2 1 1 1 0 0 0 0

(27)

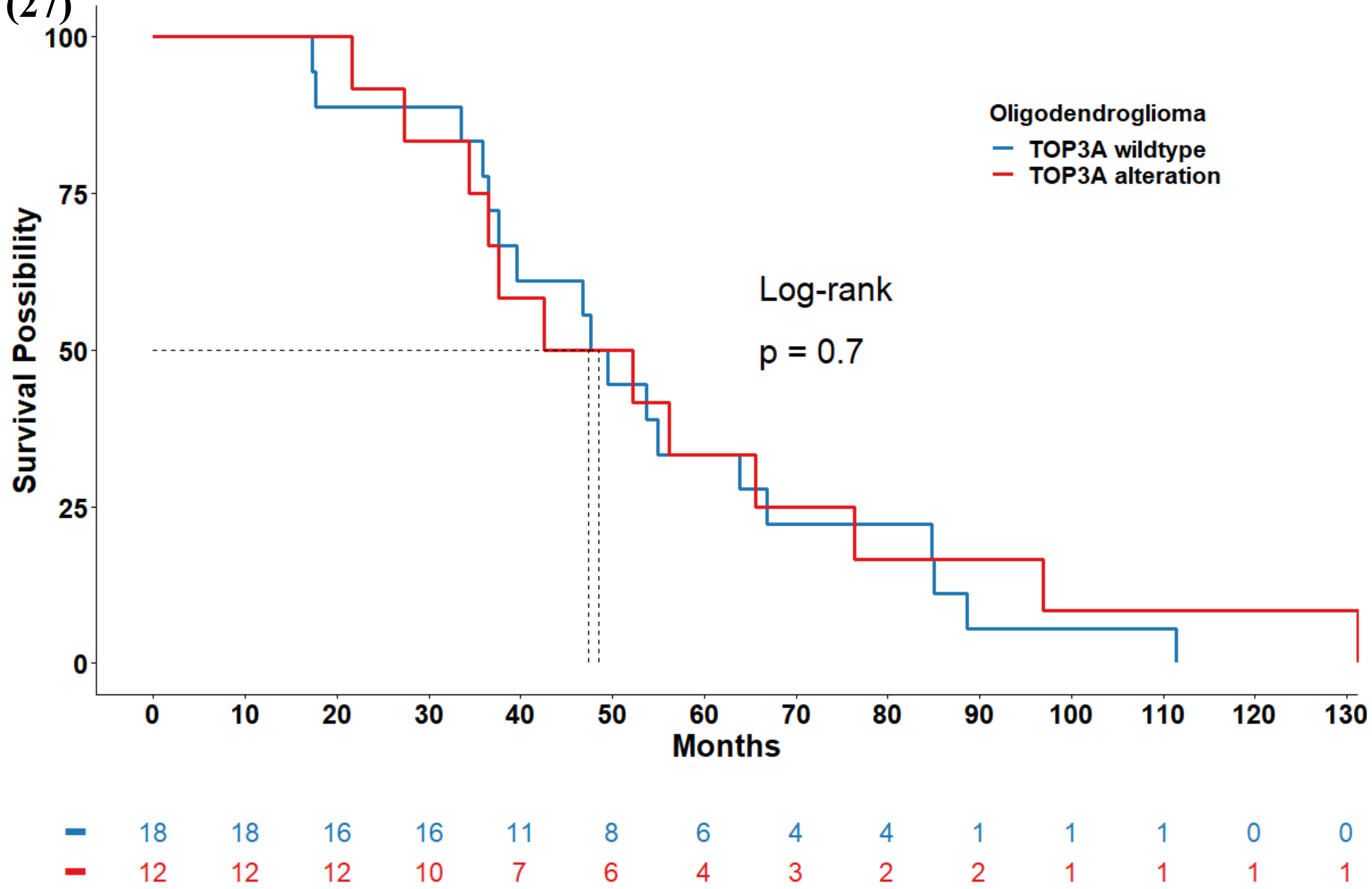

Survival Possibility
100
75
50
25
0
Oligodendroglioma
TOP3A wildtype
TOP3A alteration
Log-rank
p = 0.7
0 10 20 30 40 50 60 70 80 90 100 110 120 130
Months
18 18 16 16 11 8 6 4 4 1 1 1 0 0
12 12 12 10 7 6 4 3 2 2 1 1 1 1

(28)

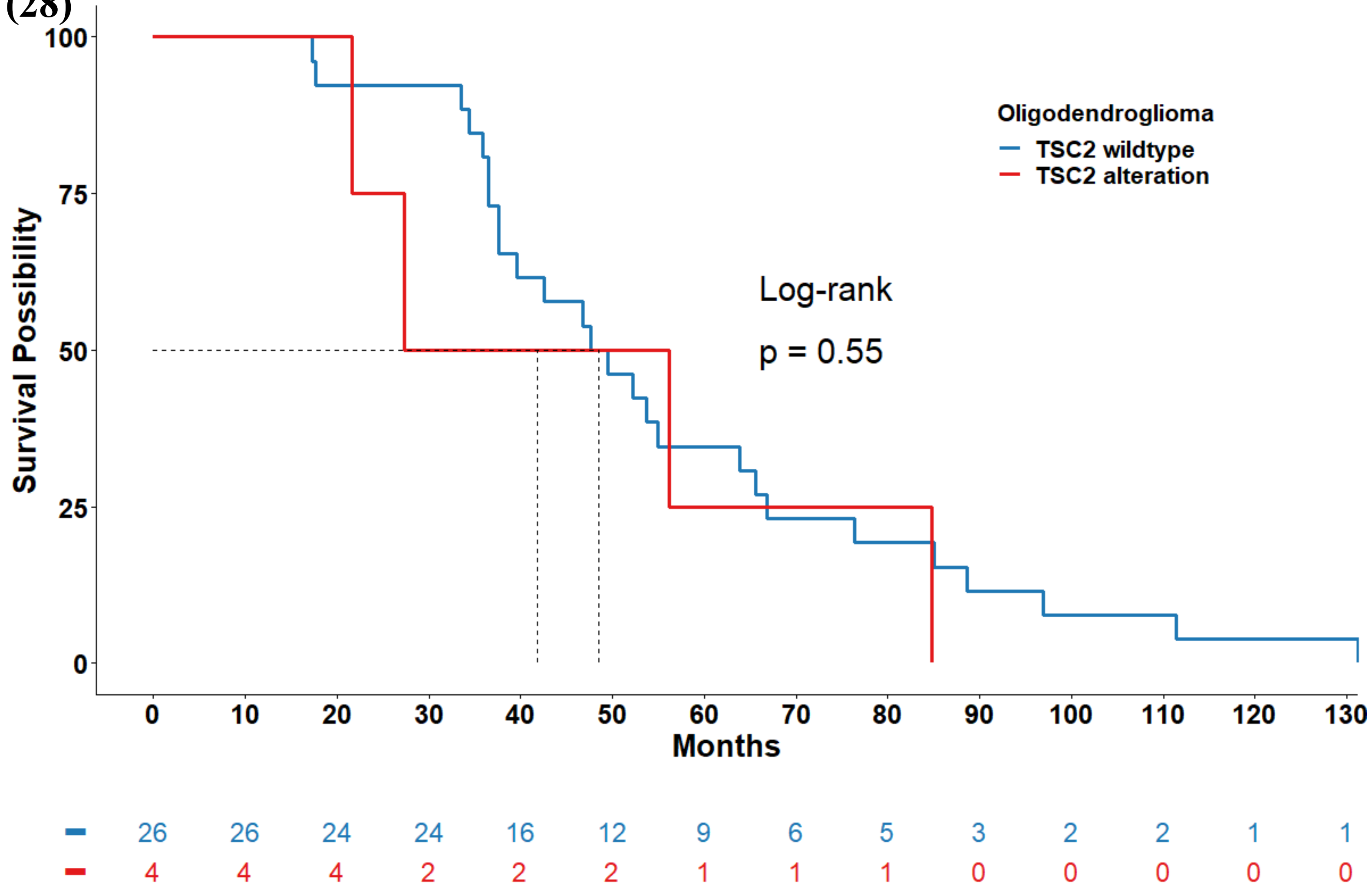

Survival Possibility
100
75
50
25
0
Oligodendroglioma
TSC2 wildtype
TSC2 alteration
Log-rank
p = 0.55
0 10 20 30 40 50 60 70 80 90 100 110 120 130
Months
26 26 24 24 16 12 9 6 5 3 2 2 1 1
4 4 4 2 2 2 1 1 1 0 0 0 0 0

(29)

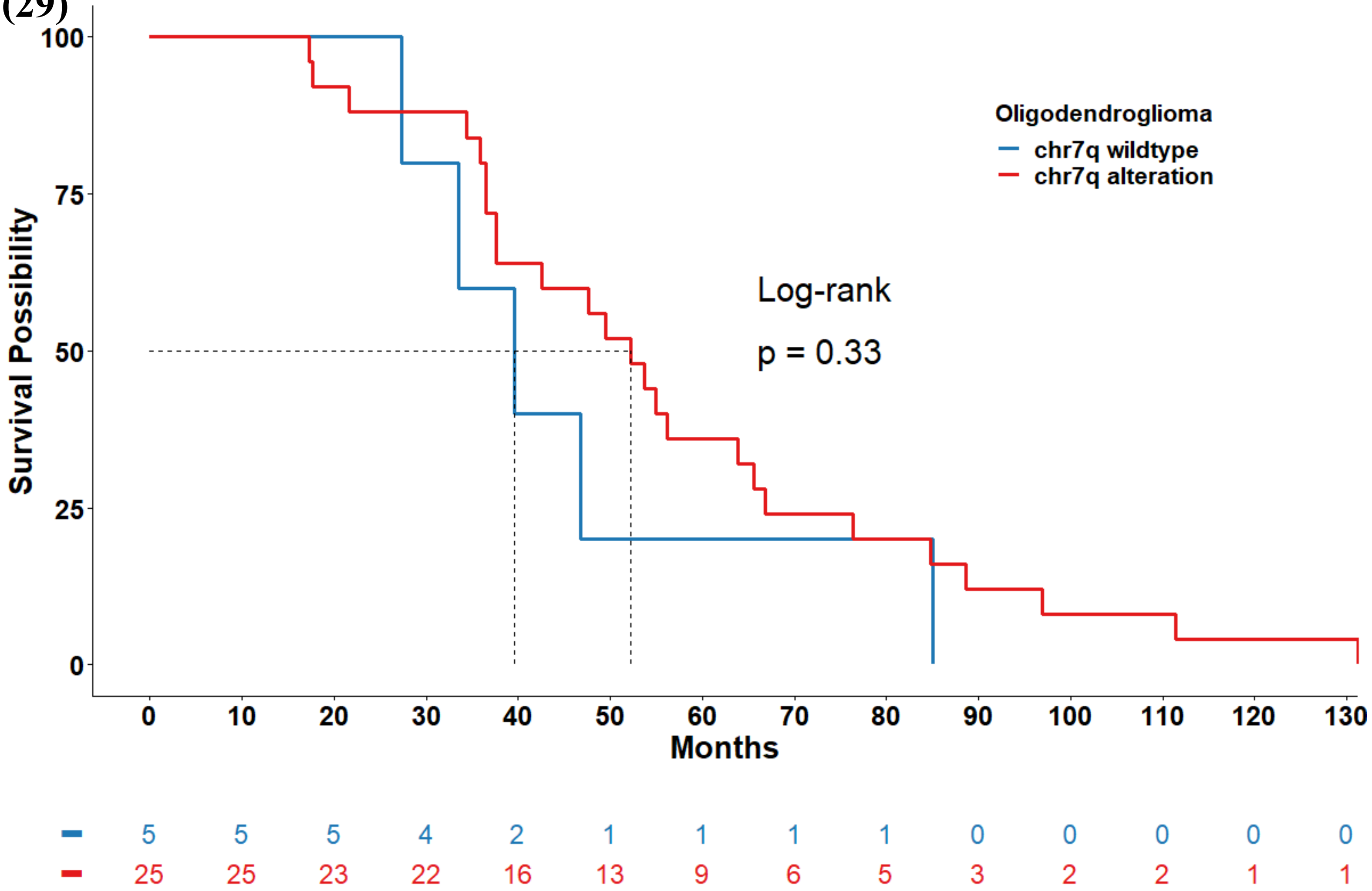

Survival Possibility
100
75
50
25
0
Oligodendroglioma
chr7q wildtype
chr7q alteration
Log-rank
p = 0.33
0
10
20
30
40
50
60
70
80
90
100
110
120
130
Months
5 5 5 4 2 1 1 1 1 0 0 0 0 0
25 25 23 22 16 13 9 6 5 3 2 2 1 1
