# Supplementary material for: Clinical updates on gliomas and implications of the 5th edition of the WHO classification of central nervous system tumors
Source: Front Oncol. 2023 Mar 14;13:1131642. doi: 10.3389/fonc.2023.1131642 (PMC10043404; doi:10.3389/fonc.2023.1131642)
Supplement: Supplementary file 5 [file DataSheet_5.pdf]

Supplement Figure 5. Kaplan–Meier curves showing no significant effects of certain genetic alterations on the overall survival of patients with paediatric-type diffuse glioma (1-27). Since the numbers of patients divided into two groups (e.g. BRAF wildtype/ BRAF alteration) using different parameters varied, we only enrolled the parameters using which the number of patients in either group was above 3. Parameters used in this section were shown as follows.

(1) BRAF 1
(2) CDK4 2
(3) CDK6 3
(4) CDKN2A 4
(5) CDKN2B 5
(6) FGFR1 6
(7) FGFR4 7
(8) KIT 8
(9) KMT5B 9
(10) KRAS 10
(11) MET 11
(12) MYB 12
(13) MYBL1 13
(14) MYC 14
(15) MYCN 15
(16) NOTCH1 16
(17) NTRK2 17
(18) NTRK3 18
(19) PDGFRA 19
(20) PIK3CA 20
(21) PPM1D 21
(22) PTEN 22
(23) PTPN11 23
(24) RB1 24
(25) TOP3A 25
(26) TP53 26
(27) chr19q 27

(1)

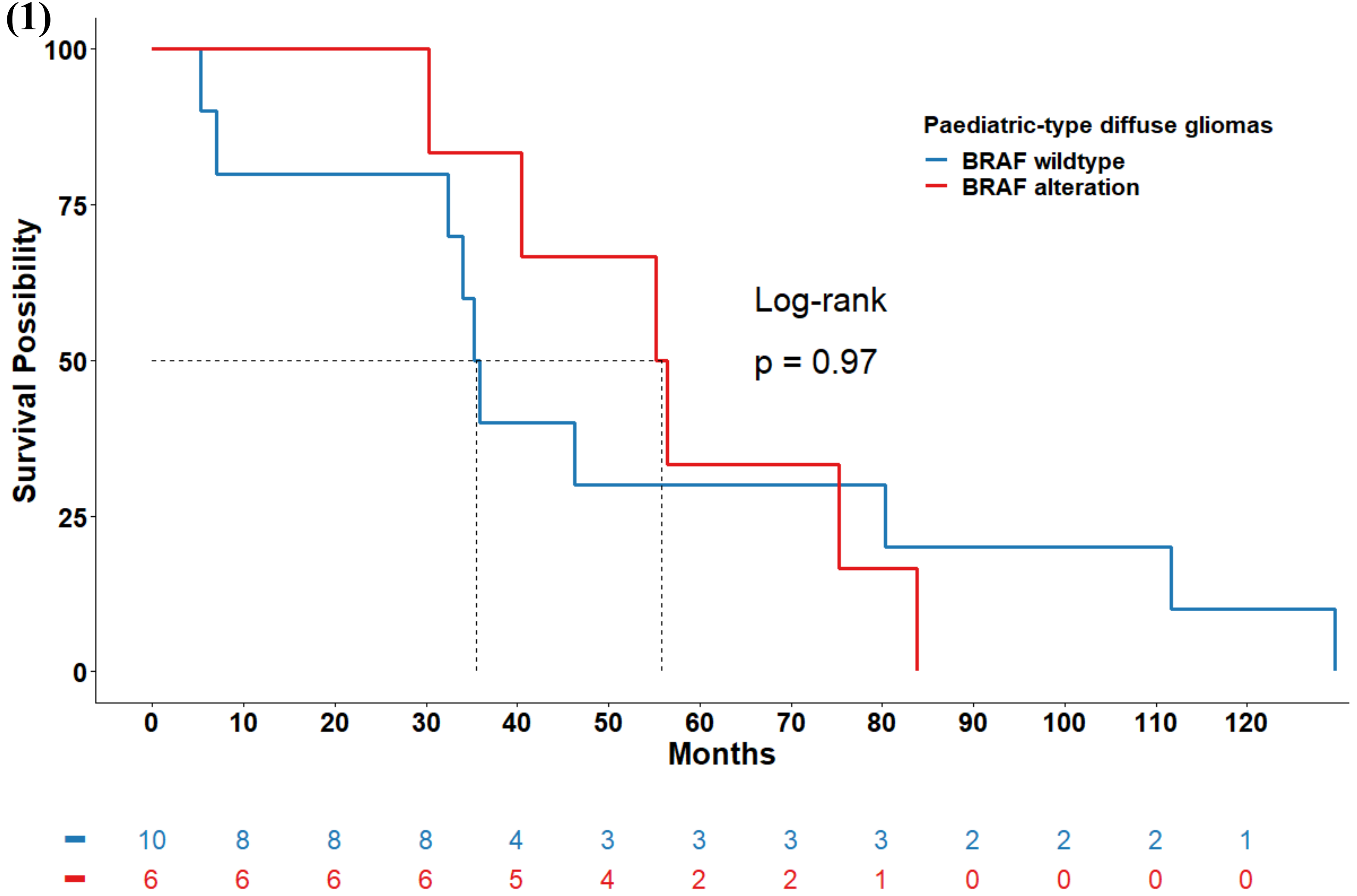

Paediatric-type diffuse gliomas
BRAF wildtype
BRAF alteration
Log-rank
p = 0.97
Survival Possibility
100
75
50
25
0
0
10
20
30
40
50
60
70
80
90
100
110
120
Months
10 8 8 8 4 3 3 3 3 2 2 2 1
6 6 6 6 5 4 2 2 1 0 0 0 0

(2)

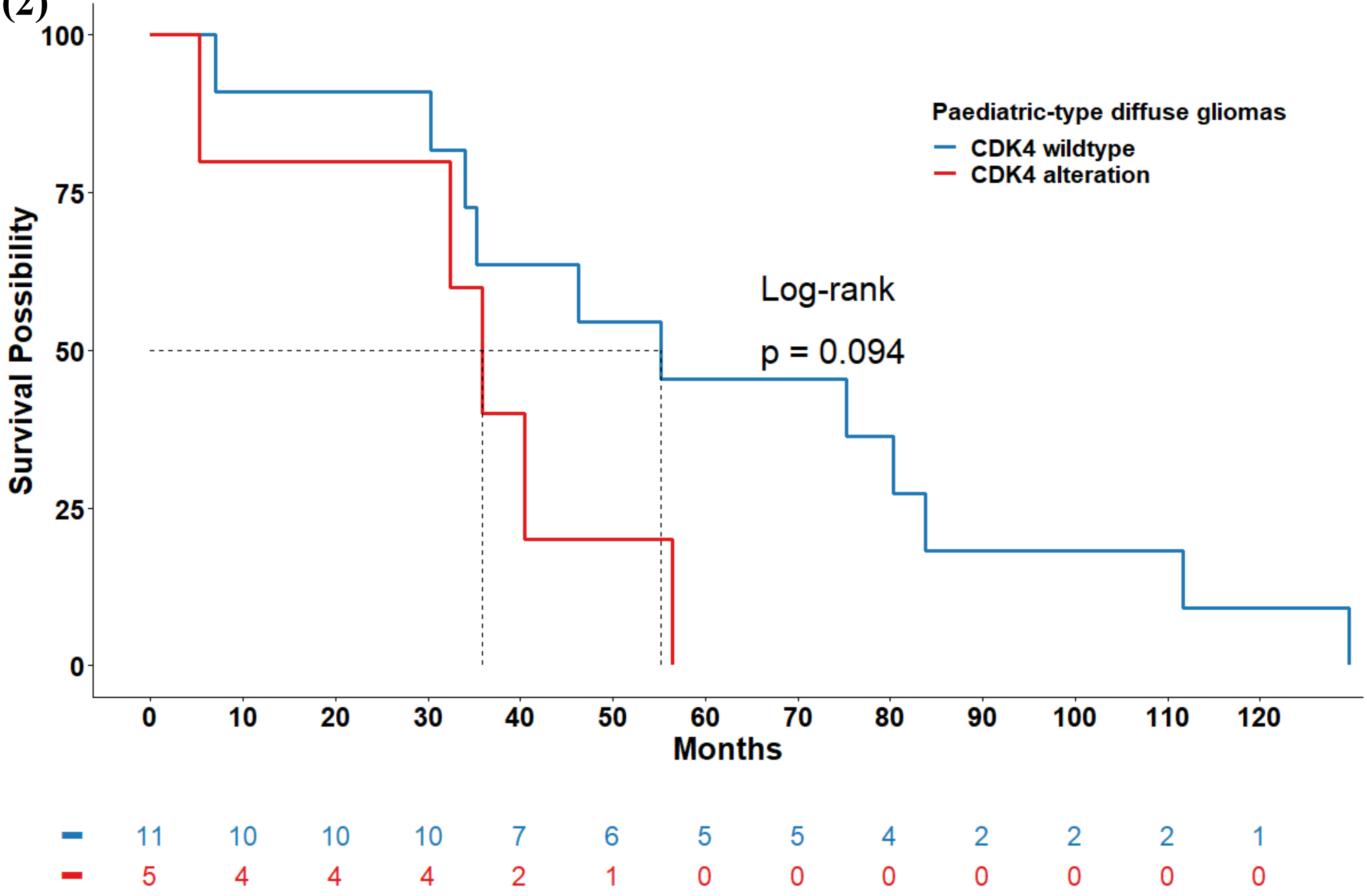
Paediatric-type diffuse gliomas
CDK4 wildtype
CDK4 alteration
Log-rank
p = 0.094
Survival Possibility
100
75
50
25
0
0
10
20
30
40
50
60
70
80
90
100
110
120
Months
11 10 10 10 7 6 5 5 4 2 2 2 1
5 4 4 4 2 1 0 0 0 0 0 0 0

(3)

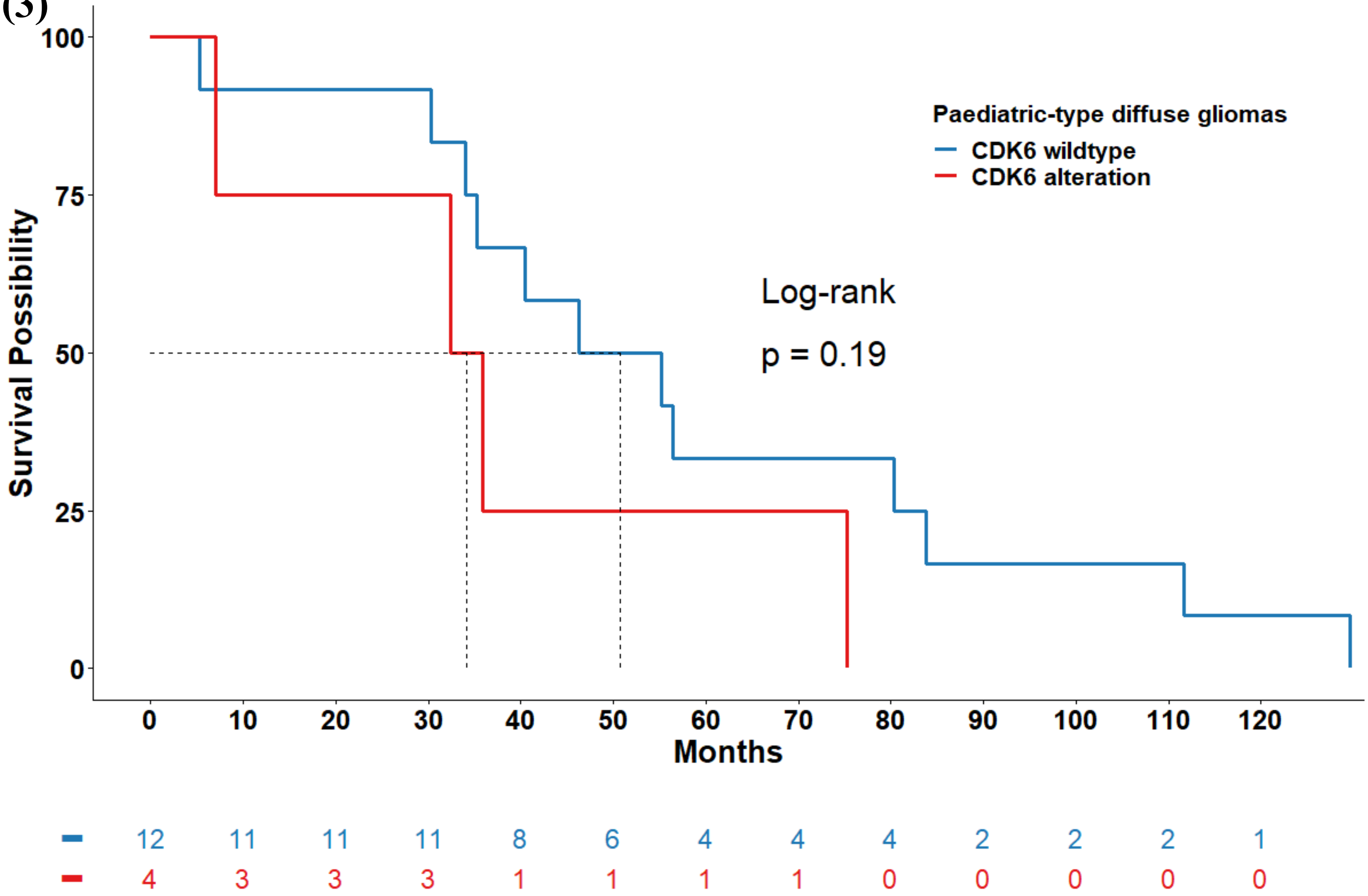

Paediatric-type diffuse gliomas
CDK6 wildtype
CDK6 alteration
Log-rank
p = 0.19
Survival Possibility
100
75
50
25
0
0 10 20 30 40 50 60 70 80 90 100 110 120
Months
12 11 11 11 8 6 4 4 4 2 2 2 1
4 3 3 3 1 1 1 1 0 0 0 0 0

(4)

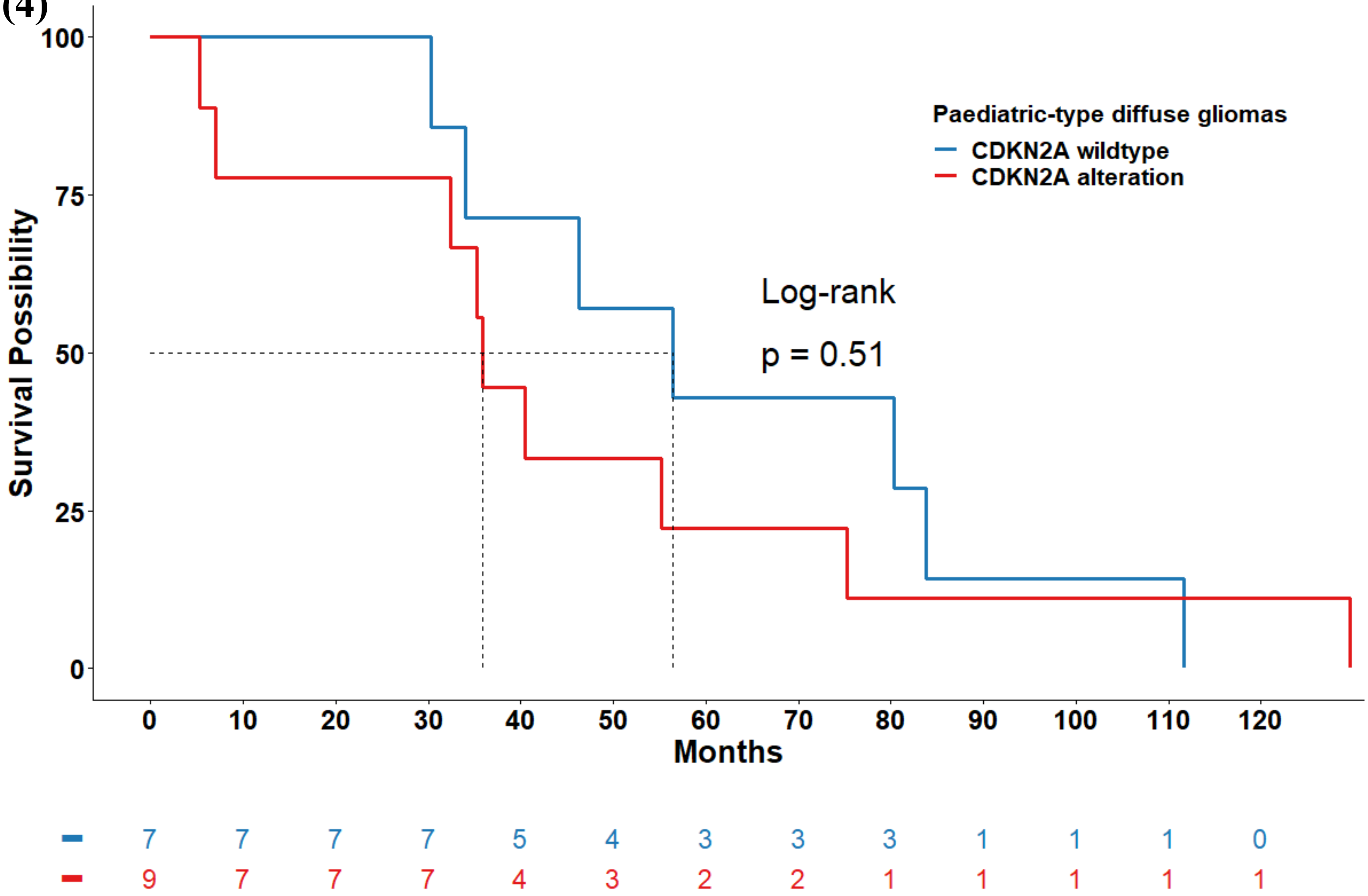
Paediatric-type diffuse gliomas
CDKN2A wildtype
CDKN2A alteration
Log-rank
p = 0.51
Survival Possibility
Months
0
25
50
75
100
0 10 20 30 40 50 60 70 80 90 100 110 120
7 7 7 7 5 4 3 3 3 1 1 1 0
9 7 7 7 4 3 2 2 1 1 1 1 1

(5)

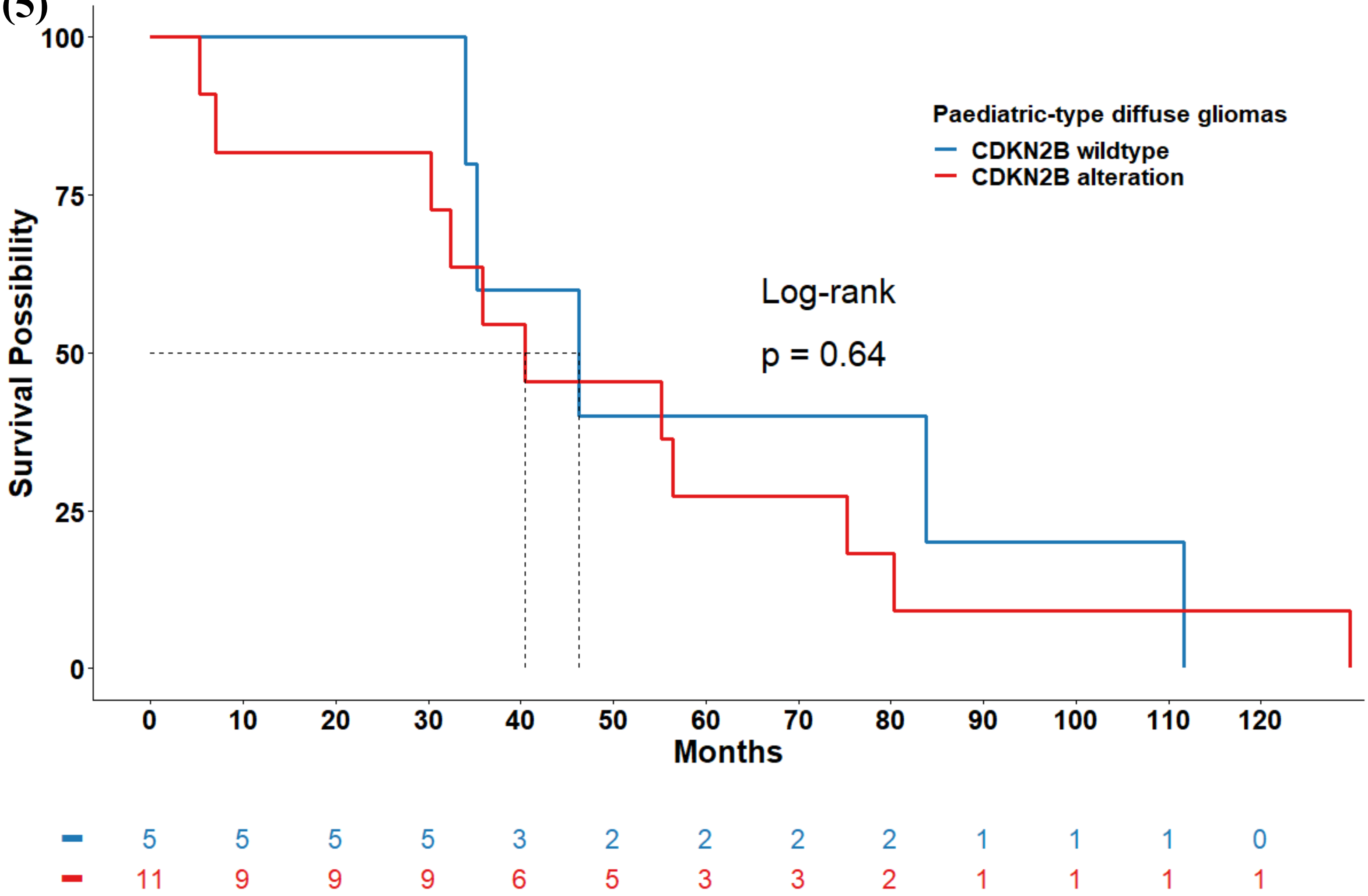

Paediatric-type diffuse gliomas
CDKN2B wildtype
CDKN2B alteration
Log-rank
p = 0.64
Survival Possibility
100
75
50
25
0
0 10 20 30 40 50 60 70 80 90 100 110 120
Months
5 5 5 5 3 2 2 2 2 1 1 1 0
11 9 9 9 6 5 3 3 2 1 1 1 1

(6)

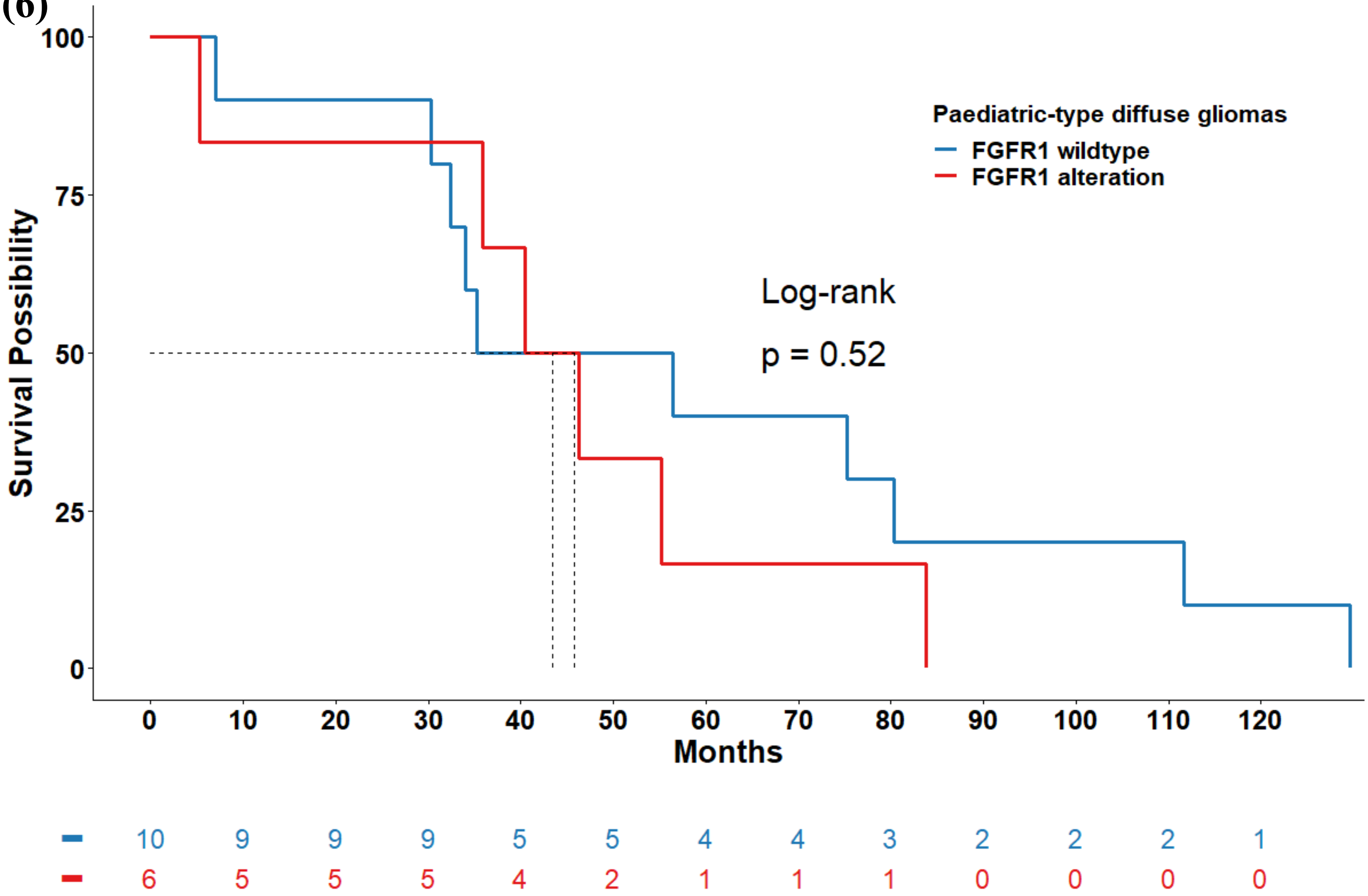
Survival Possibility
100
75
50
25
0
Paediatric-type diffuse gliomas
FGFR1 wildtype
FGFR1 alteration
Log-rank
p = 0.52
0 10 20 30 40 50 60 70 80 90 100 110 120
Months
10 9 9 9 5 5 4 4 3 2 2 2 1
6 5 5 5 4 2 1 1 1 0 0 0 0

(7)

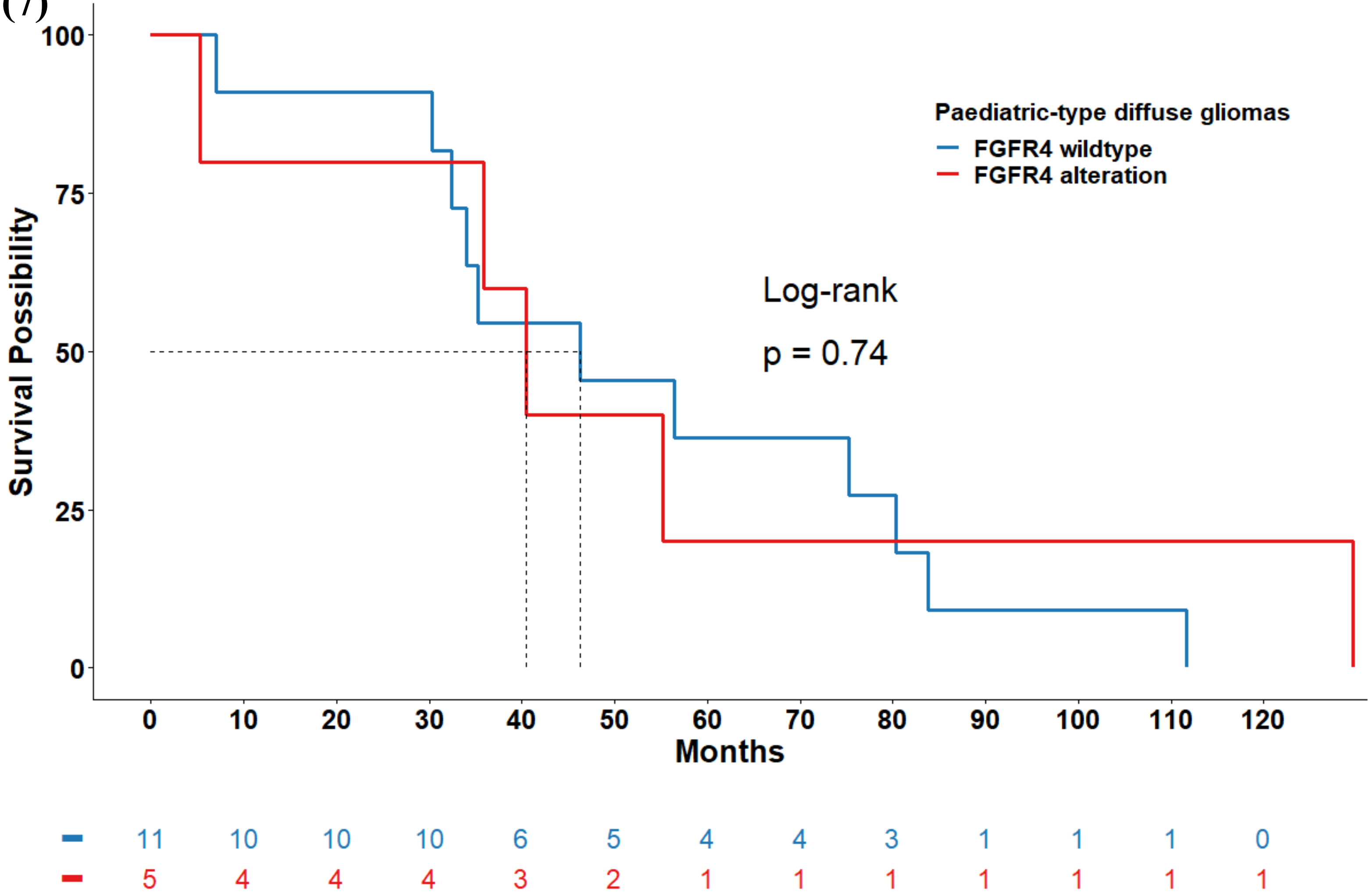
Survival Possibility
100
75
50
25
0
Paediatric-type diffuse gliomas
FGFR4 wildtype
FGFR4 alteration
Log-rank
p = 0.74
0
10
20
30
40
50
60
70
80
90
100
110
120
Months
11 10 10 10 6 5 4 4 3 1 1 1 0
5 4 4 4 3 2 1 1 1 1 1 1 1

(8)

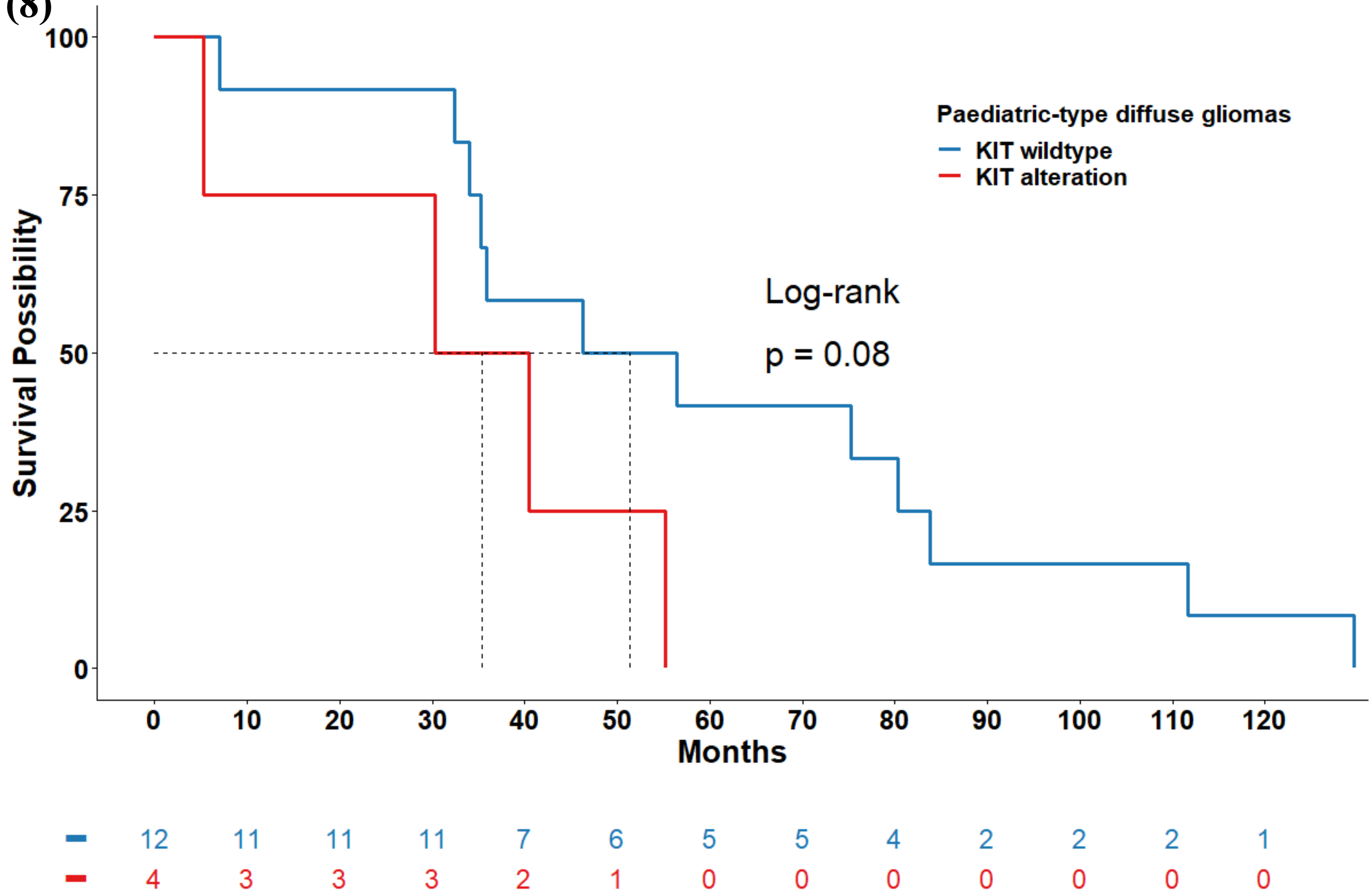

Survival Possibility
100
75
50
25
0
0
10
20
30
40
50
60
70
80
90
100
110
120
Months
Paediatric-type diffuse gliomas
KIT wildtype
KIT alteration
Log-rank
p = 0.08
12 11 11 11 7 6 5 5 4 2 2 2 1
4 3 3 3 2 1 0 0 0 0 0 0 0

(9)

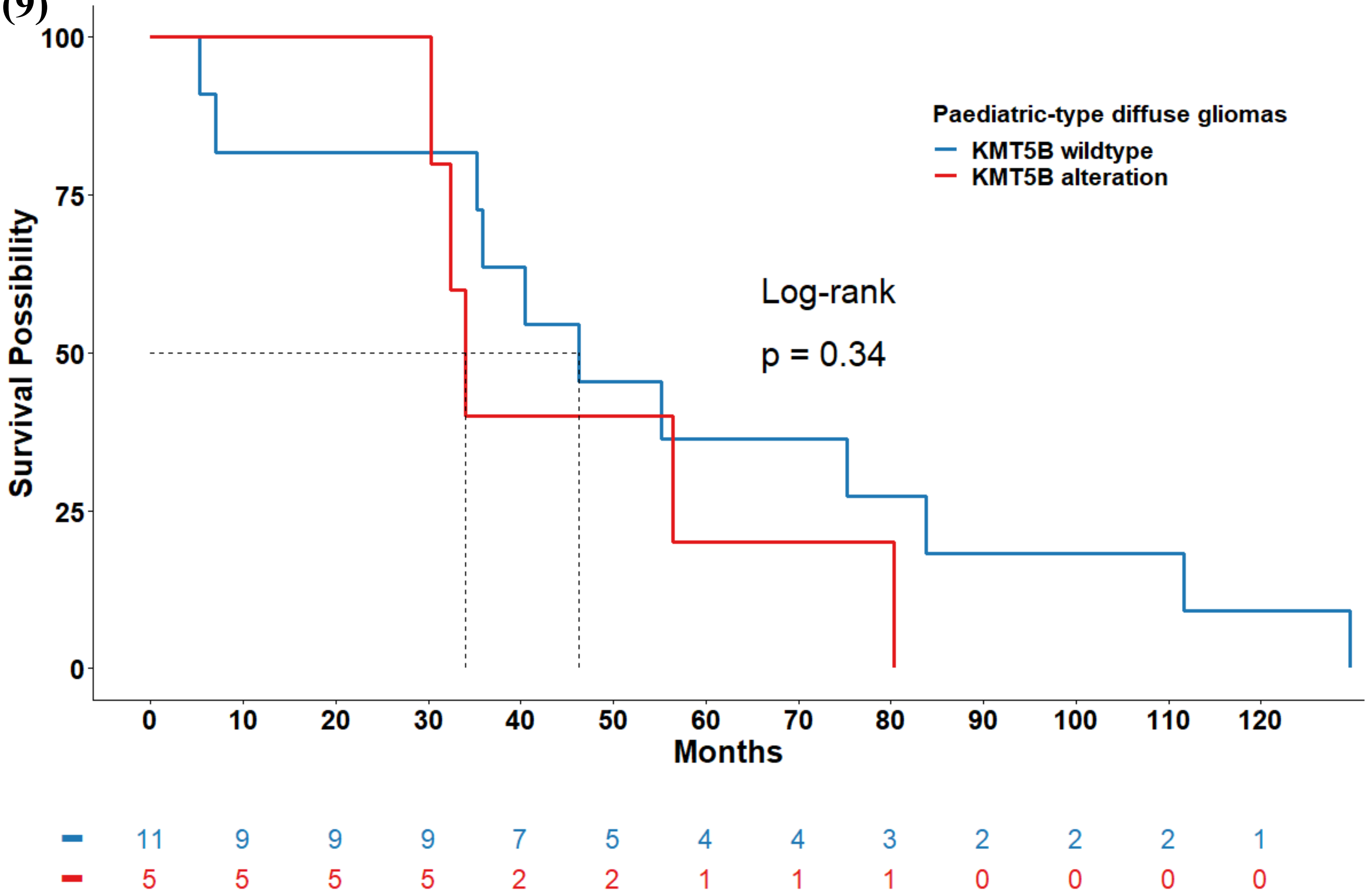

Paediatric-type diffuse gliomas
KMT5B wildtype
KMT5B alteration
Log-rank
p = 0.34
Survival Possibility
100
75
50
25
0
0 10 20 30 40 50 60 70 80 90 100 110 120
Months
11 9 9 9 7 5 4 4 3 2 2 2 1
5 5 5 5 2 2 1 1 1 0 0 0 0

(10)

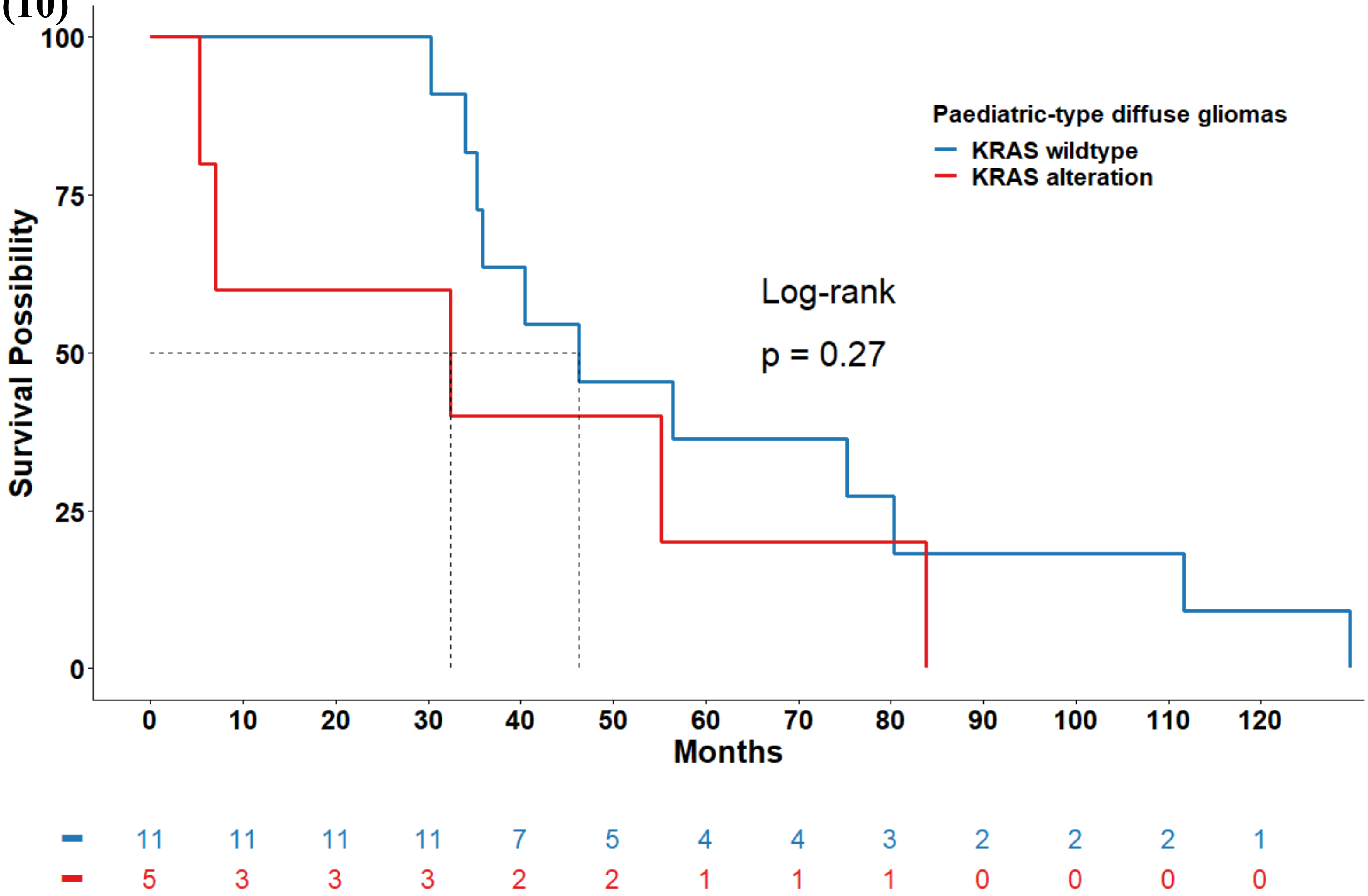
Paediatric-type diffuse gliomas
KRAS wildtype
KRAS alteration
Log-rank
p = 0.27
Survival Possibility
100
75
50
25
0
0 10 20 30 40 50 60 70 80 90 100 110 120
Months
11 11 11 11 7 5 4 4 3 2 2 2 1
5 3 3 3 2 2 1 1 1 0 0 0 0

(11)

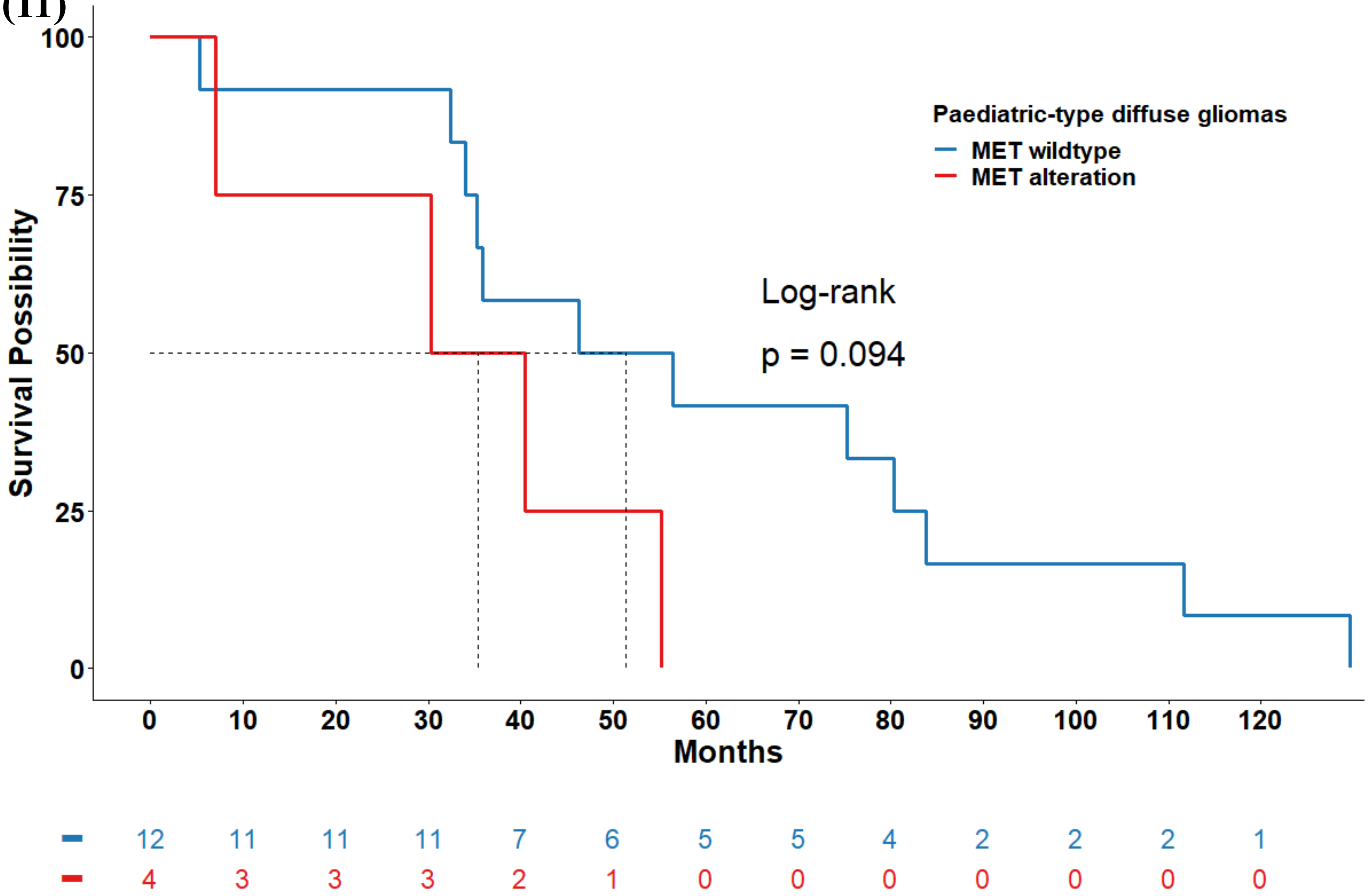

Paediatric-type diffuse gliomas
MET wildtype
MET alteration
Log-rank
p = 0.094
Survival Possibility
100
75
50
25
0
0
10
20
30
40
50
60
70
80
90
100
110
120
Months
12 11 11 11 7 6 5 5 4 2 2 2 1
4 3 3 3 2 1 0 0 0 0 0 0 0

(12)

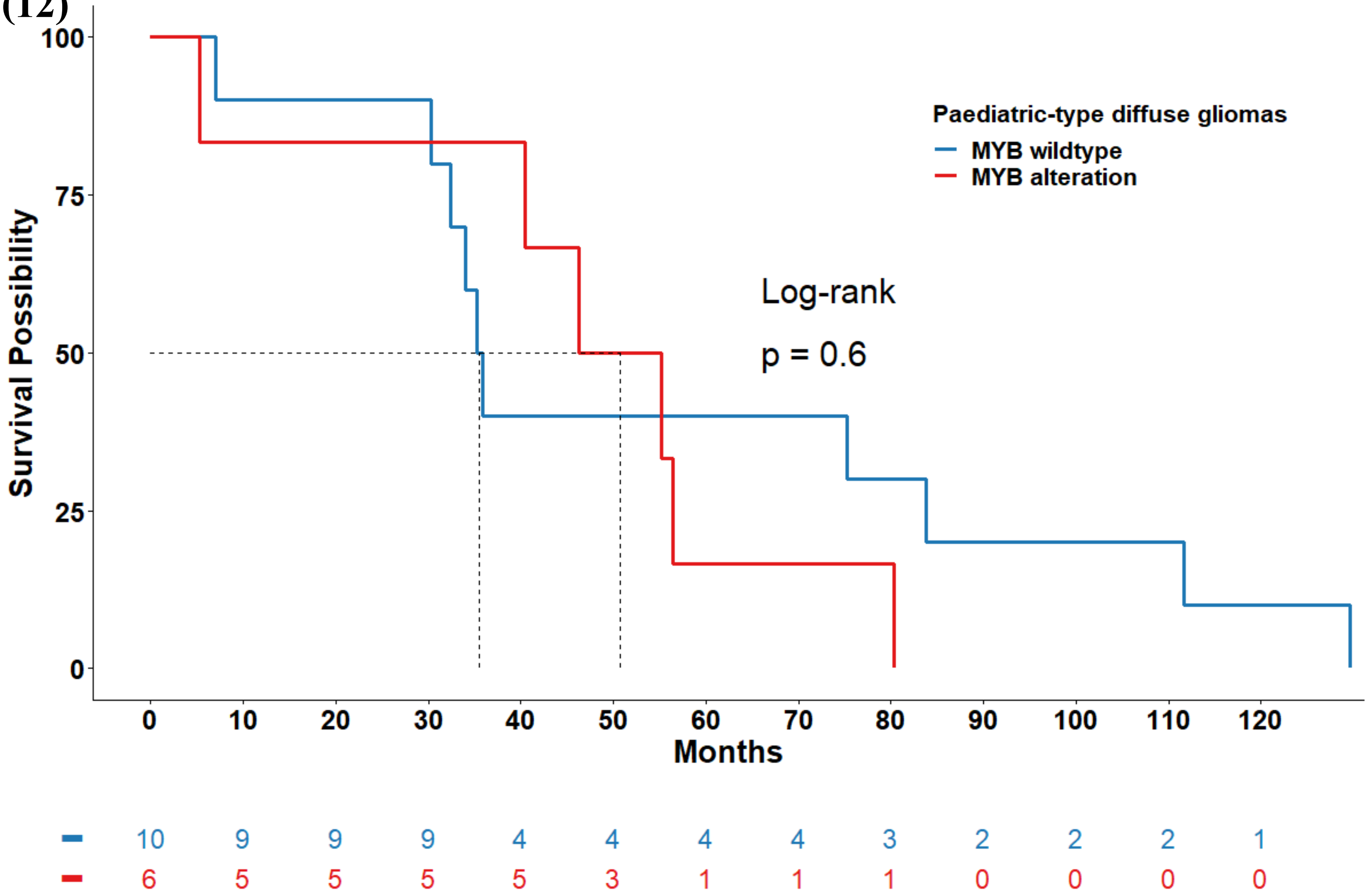

100
75
50
25
0
Survival Possibility
Paediatric-type diffuse gliomas
MYB wildtype
MYB alteration
Log-rank
p = 0.6
0
10
20
30
40
50
60
70
80
90
100
110
120
Months
10 9 9 9 4 4 4 4 3 2 2 2 1
6 5 5 5 5 3 1 1 1 0 0 0 0

(13)

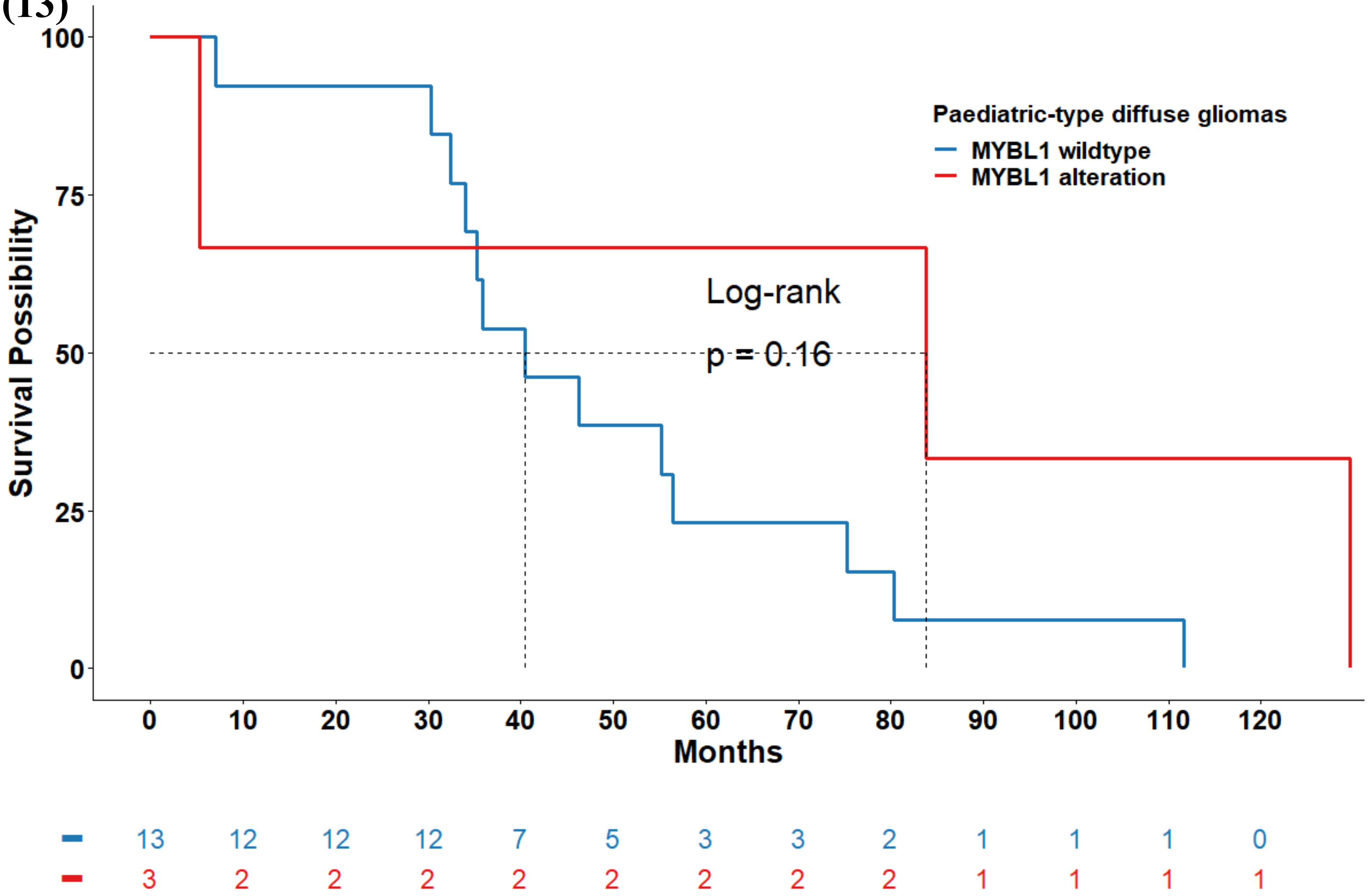

Survival Possibility
100
75
50
25
0
Paediatric-type diffuse gliomas
MYBL1 wildtype
MYBL1 alteration
Log-rank
p = 0.16
0
10
20
30
40
50
60
70
80
90
100
110
120
Months
13 12 12 12 7 5 3 3 2 1 1 1 0
3 2 2 2 2 2 2 2 2 1 1 1 1

(14)

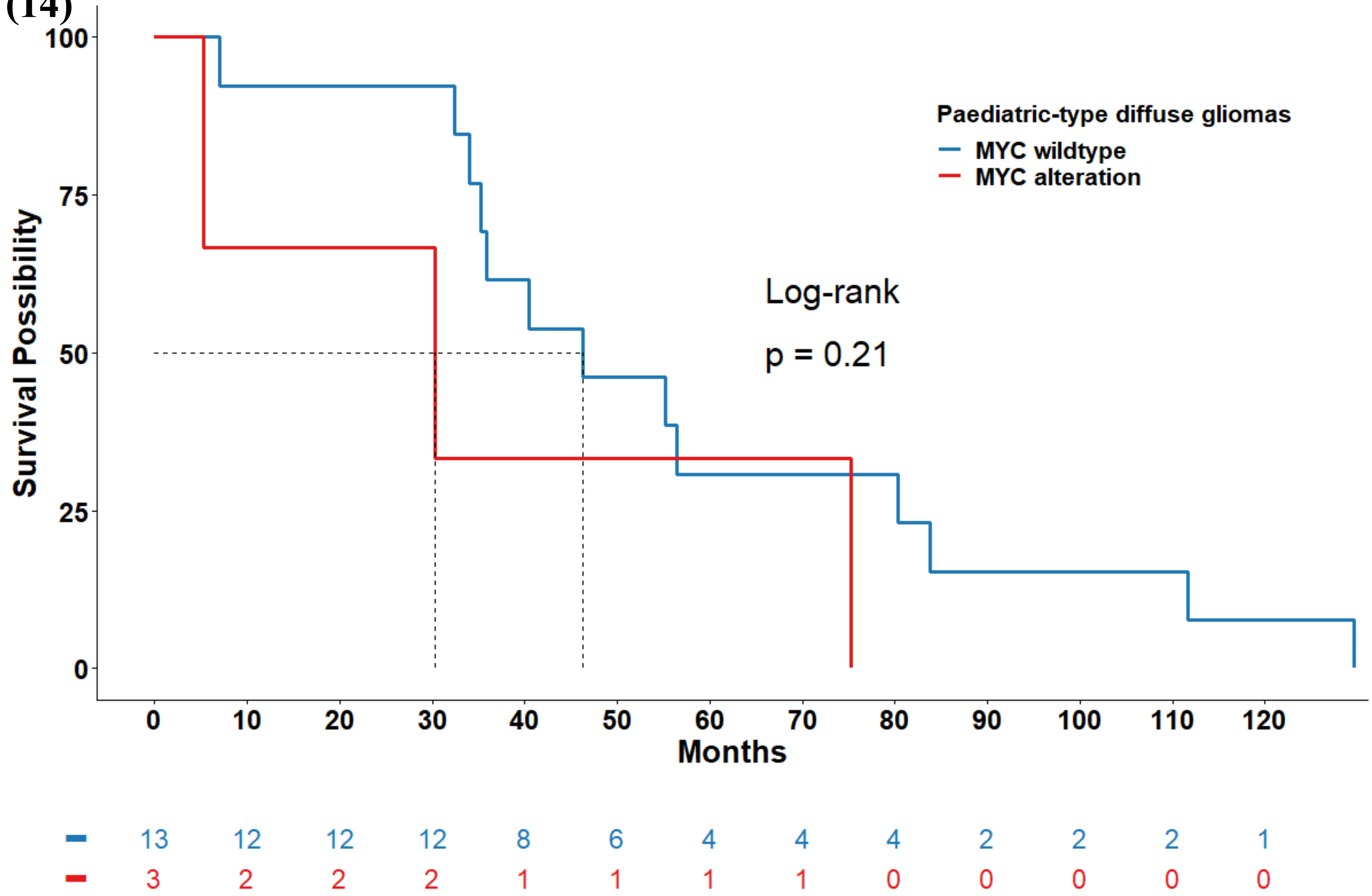

Survival Possibility
100
75
50
25
0
0
10
20
30
40
50
60
70
80
90
100
110
120
Months
Paediatric-type diffuse gliomas
MYC wildtype
MYC alteration
Log-rank
p = 0.21
13 12 12 12 8 6 4 4 4 2 2 2 1
3 2 2 2 1 1 1 1 0 0 0 0 0

(15)

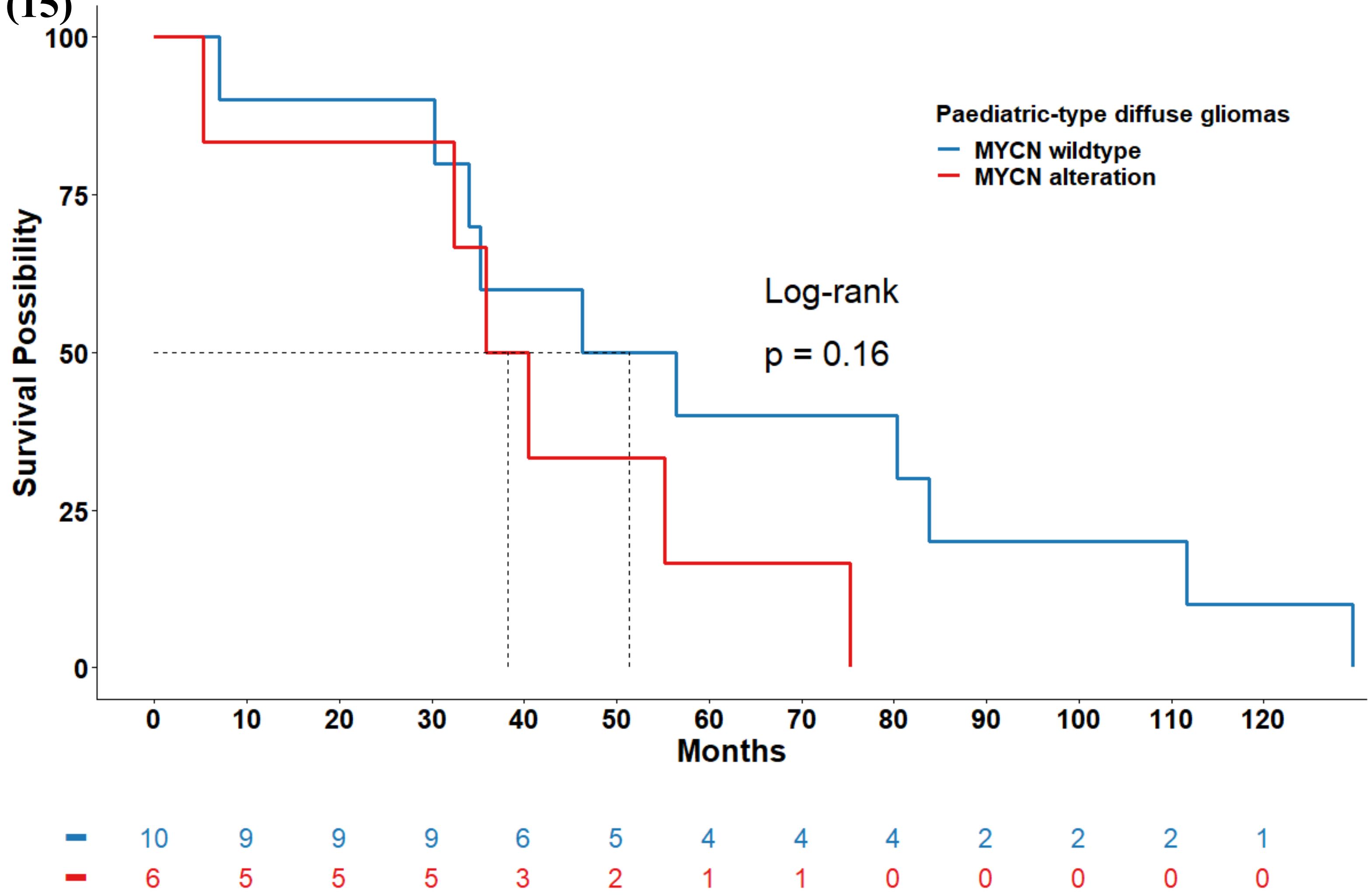

Survival Possibility
100
75
50
25
0
0
10
20
30
40
50
60
70
80
90
100
110
120
Months
Paediatric-type diffuse gliomas
MYCN wildtype
MYCN alteration
Log-rank
p = 0.16
10 9 9 9 6 5 4 4 4 2 2 2 1
6 5 5 5 3 2 1 1 0 0 0 0 0

(16)

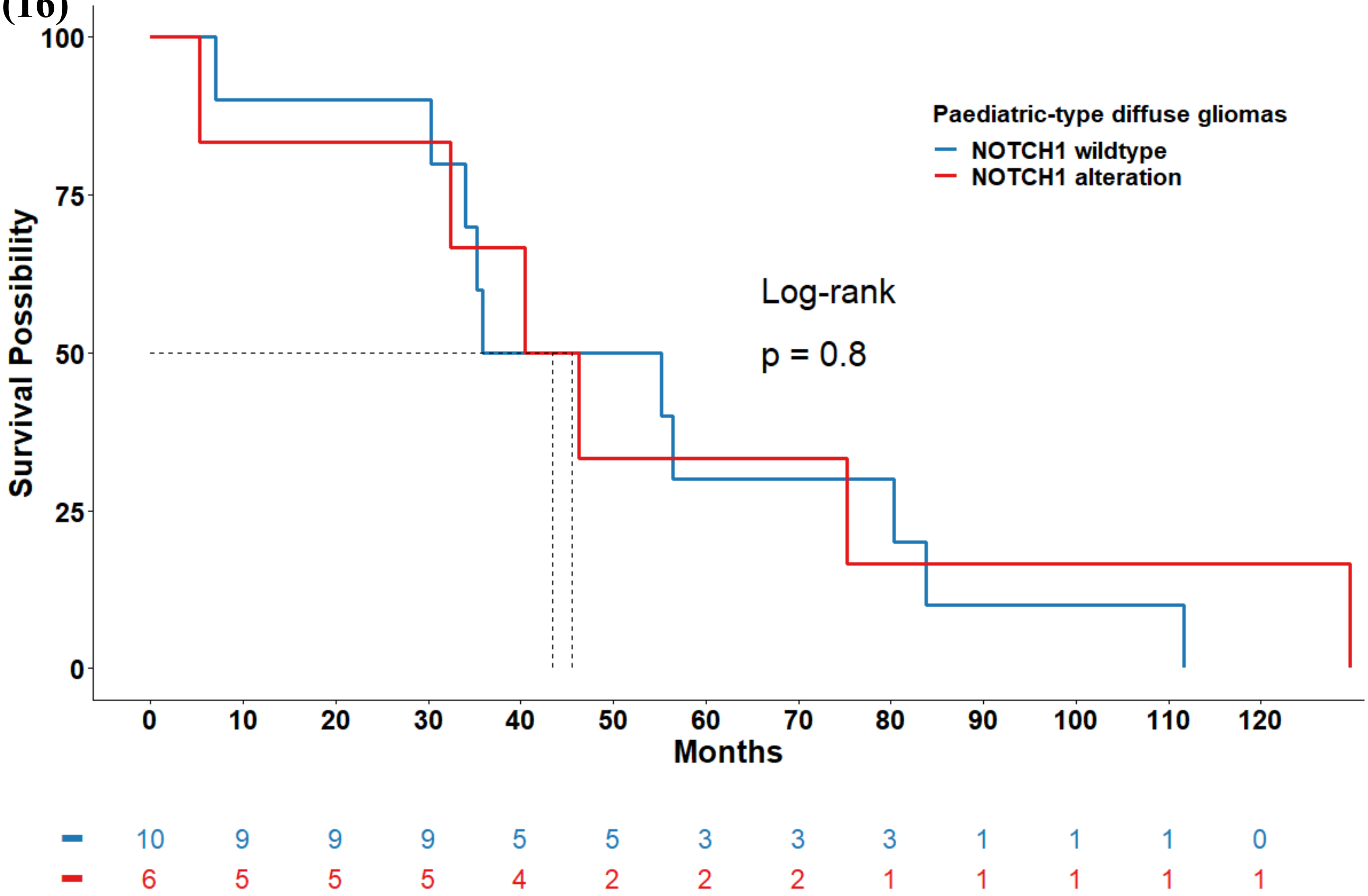

100
75
50
25
0
Survival Possibility
Paediatric-type diffuse gliomas
NOTCH1 wildtype
NOTCH1 alteration
Log-rank
p = 0.8
0
10
20
30
40
50
60
70
80
90
100
110
120
Months
10 9 9 9 5 5 3 3 3 1 1 1 0
6 5 5 5 4 2 2 2 1 1 1 1 1

(17)

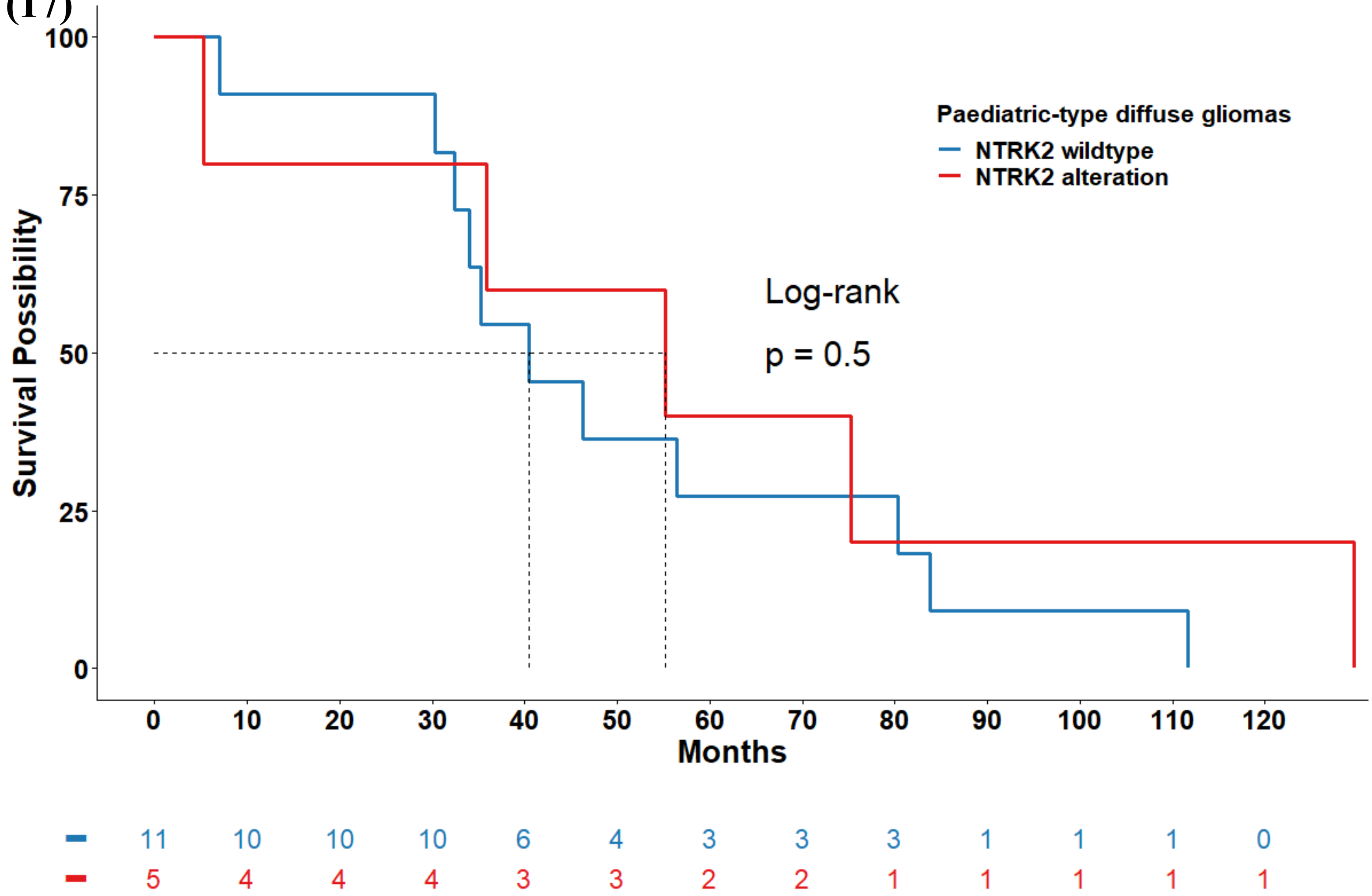

100
75
50
25
0
Survival Possibility
Paediatric-type diffuse gliomas
NTRK2 wildtype
NTRK2 alteration
Log-rank
p = 0.5
0 10 20 30 40 50 60 70 80 90 100 110 120
Months
11 10 10 10 6 4 3 3 3 1 1 1 0
5 4 4 4 3 3 2 2 1 1 1 1 1

(18)

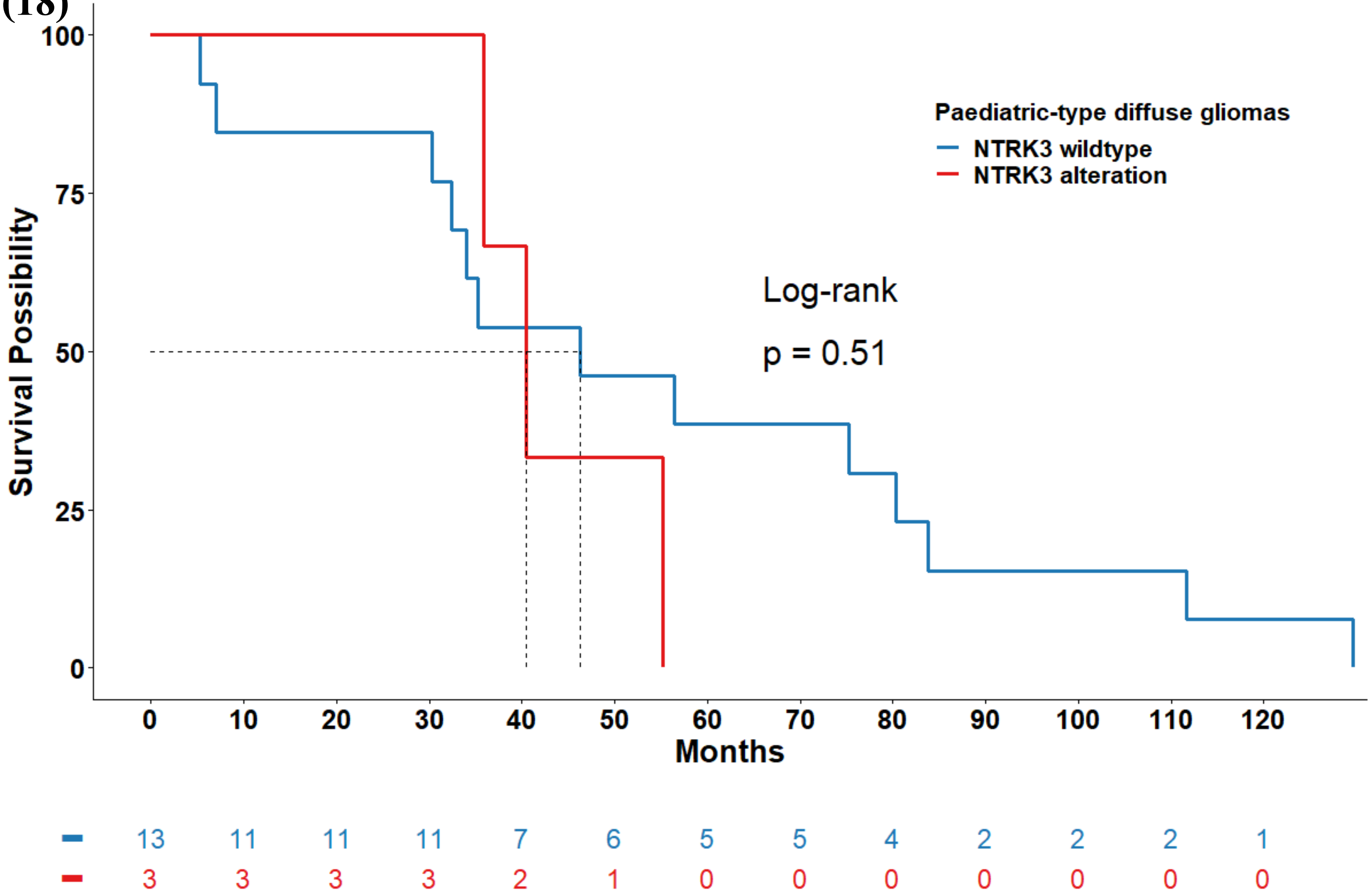

Survival Possibility
100
75
50
25
0
0 10 20 30 40 50 60 70 80 90 100 110 120
Months
Paediatric-type diffuse gliomas
NTRK3 wildtype
NTRK3 alteration
Log-rank
p = 0.51
13 11 11 11 7 6 5 5 4 2 2 2 1
3 3 3 3 2 1 0 0 0 0 0 0 0

(19)

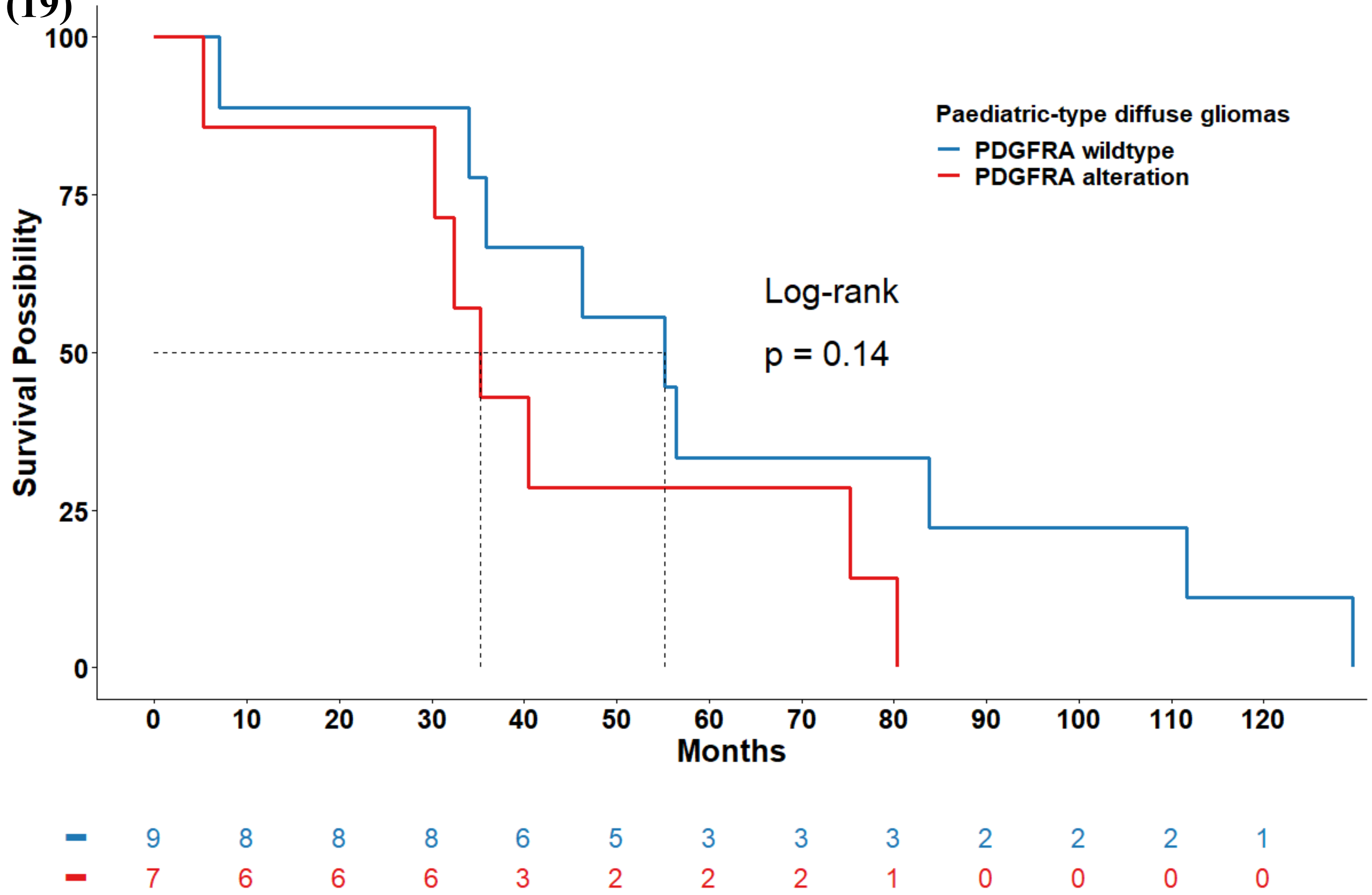

Survival Possibility
100
75
50
25
0
Months
0
10
20
30
40
50
60
70
80
90
100
110
120
Paediatric-type diffuse gliomas
PDGFRA wildtype
PDGFRA alteration
Log-rank
p = 0.14
9 8 8 8 6 5 3 3 3 2 2 2 1
7 6 6 6 3 2 2 2 1 0 0 0 0

(20)

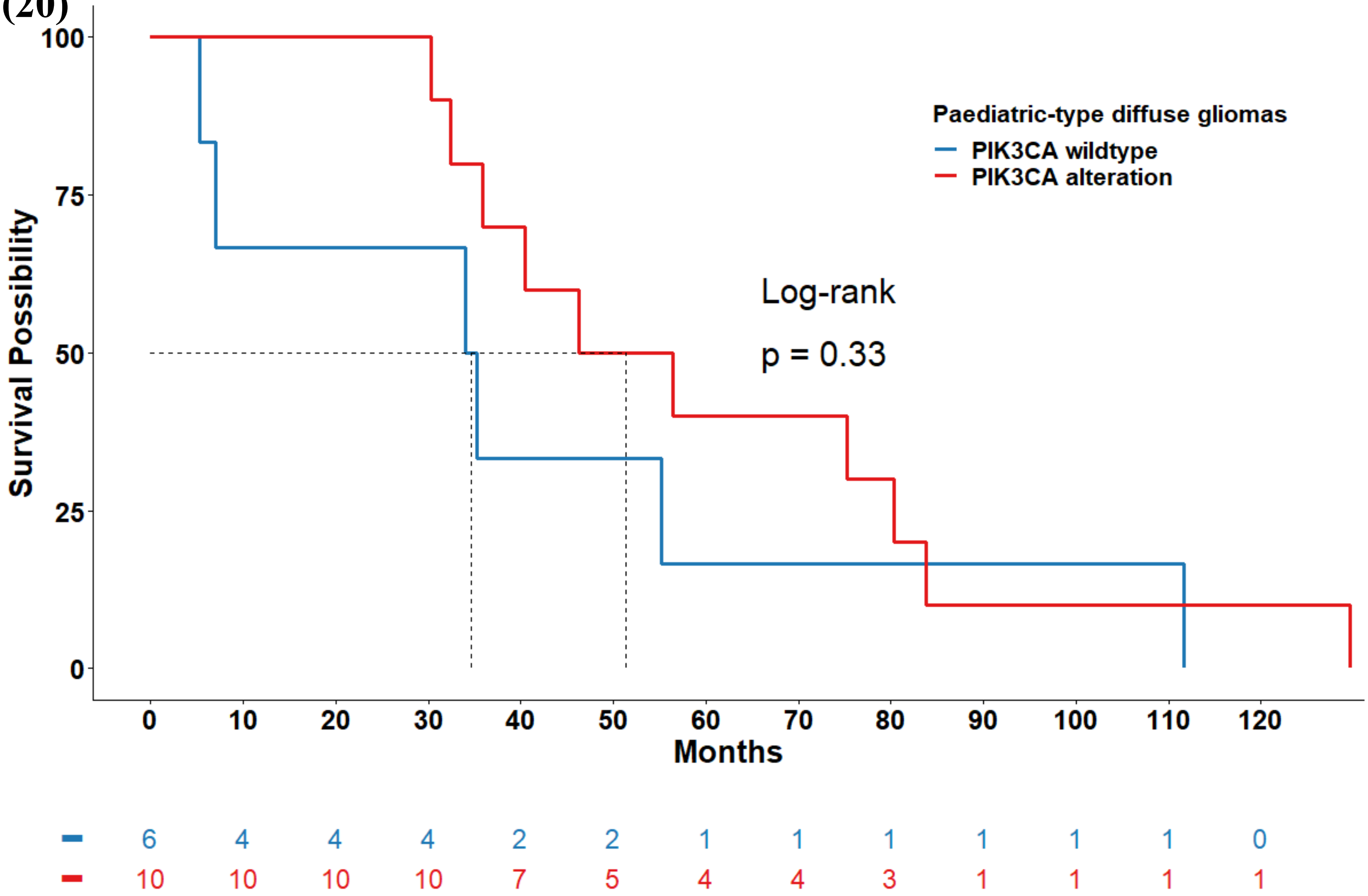

100
75
50
25
0
Survival Possibility
Paediatric-type diffuse gliomas
PIK3CA wildtype
PIK3CA alteration
Log-rank
p = 0.33
0
10
20
30
40
50
60
70
80
90
100
110
120
Months
6 4 4 4 2 2 1 1 1 1 1 1 0
10 10 10 10 7 5 4 4 3 1 1 1 1

(21)

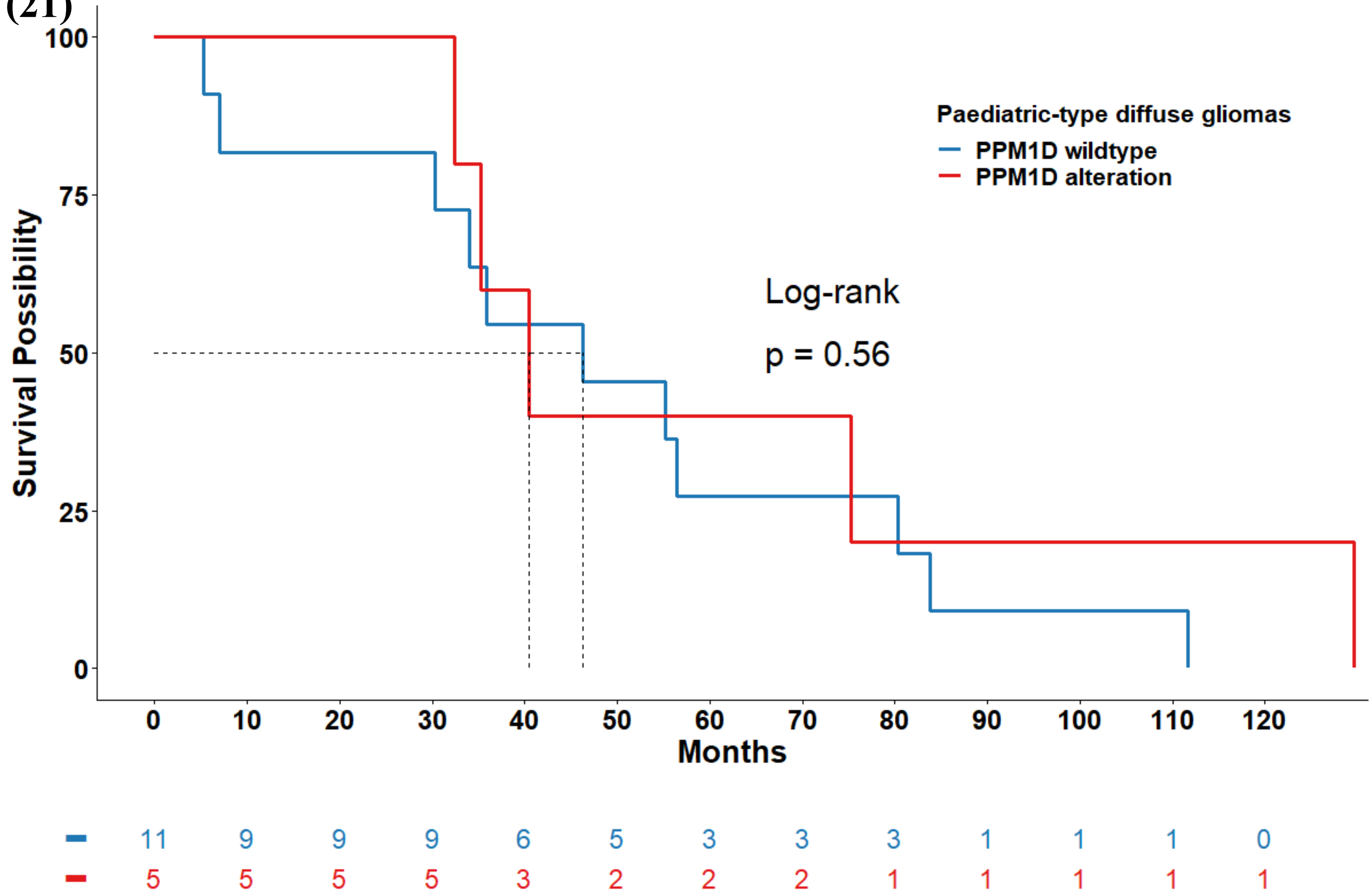

Survival Possibility
100
75
50
25
0
Paediatric-type diffuse gliomas
PPM1D wildtype
PPM1D alteration
Log-rank
p = 0.56
0
10
20
30
40
50
60
70
80
90
100
110
120
Months
11 9 9 9 6 5 3 3 3 1 1 1 0
5 5 5 5 3 2 2 2 1 1 1 1 1

(22)

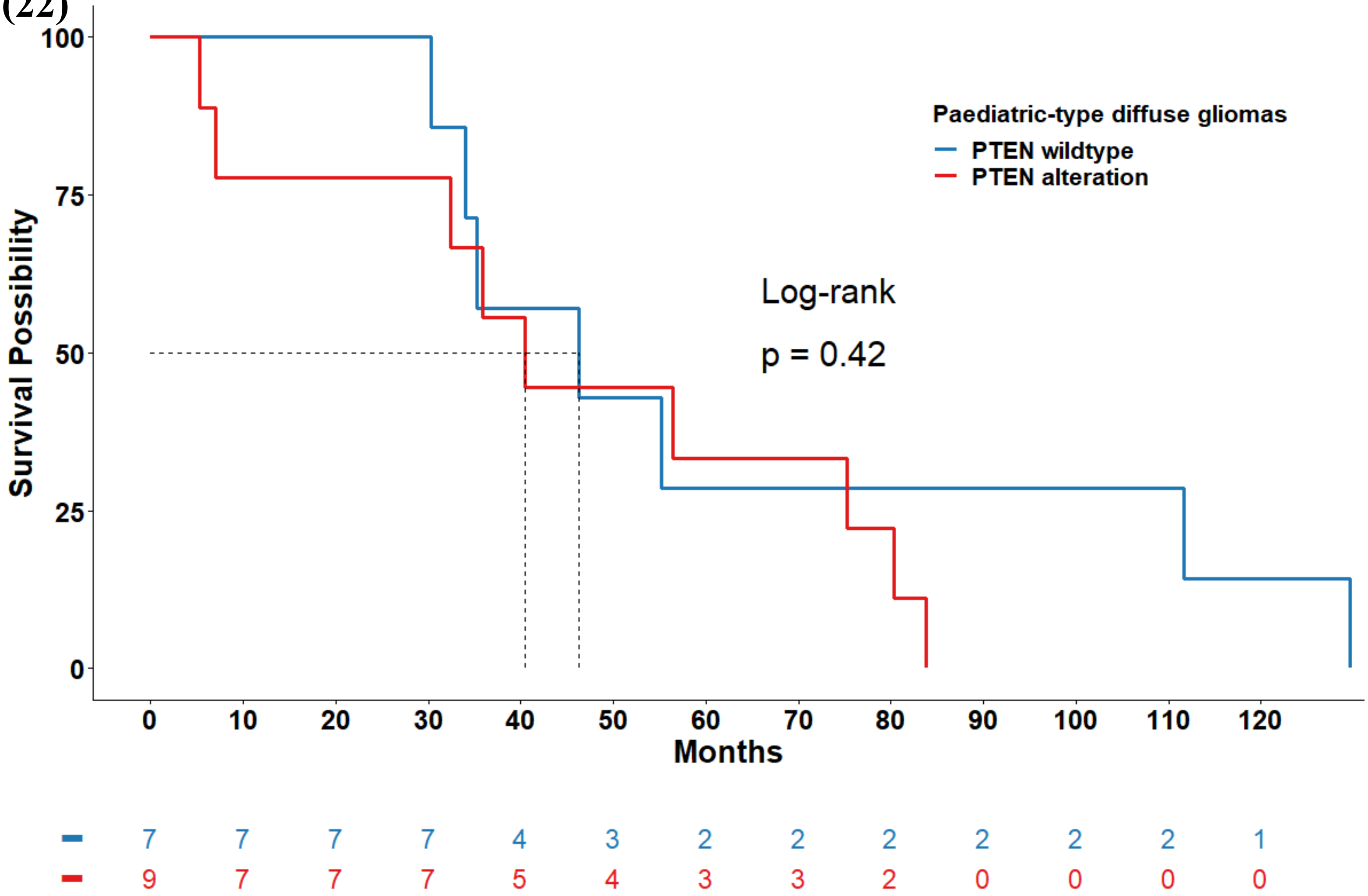

Survival Possibility
100
75
50
25
0
0
10
20
30
40
50
60
70
80
90
100
110
120
Months
Paediatric-type diffuse gliomas
PTEN wildtype
PTEN alteration
Log-rank
p = 0.42
7 7 7 7 4 3 2 2 2 2 2 2 1
9 7 7 7 5 4 3 3 2 0 0 0 0

(23)

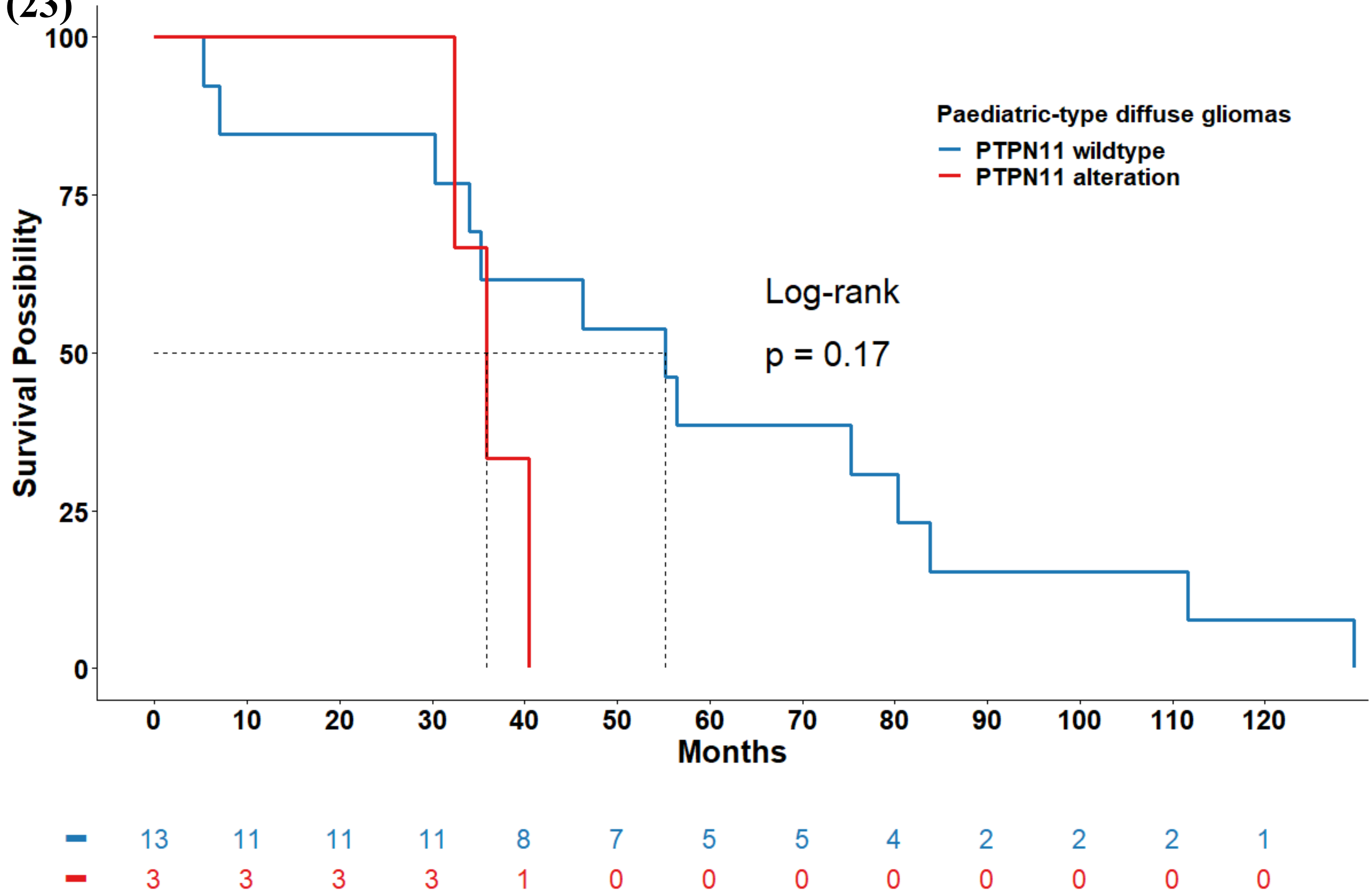

Survival Possibility
100
75
50
25
0
Paediatric-type diffuse gliomas
PTPN11 wildtype
PTPN11 alteration
Log-rank
p = 0.17
0
10
20
30
40
50
60
70
80
90
100
110
120
Months
13 11 11 11 8 7 5 5 4 2 2 2 1
3 3 3 3 1 0 0 0 0 0 0 0 0

(24)

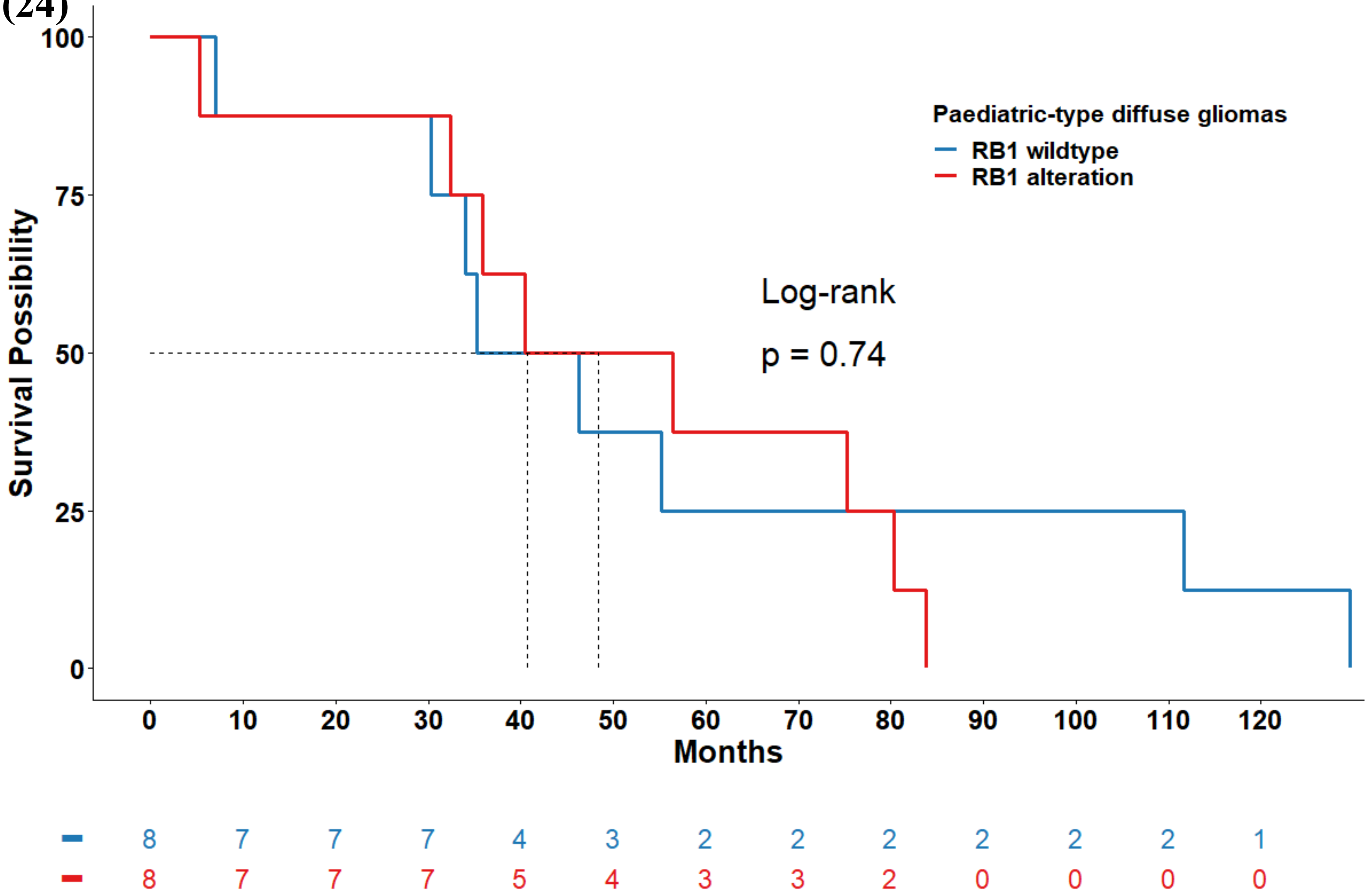

100
75
50
25
0
Survival Possibility
Paediatric-type diffuse gliomas
RB1 wildtype
RB1 alteration
Log-rank
p = 0.74
0
10
20
30
40
50
60
70
80
90
100
110
120
Months
8 7 7 7 4 3 2 2 2 2 2 2 1
8 7 7 7 5 4 3 3 2 0 0 0 0

(25)

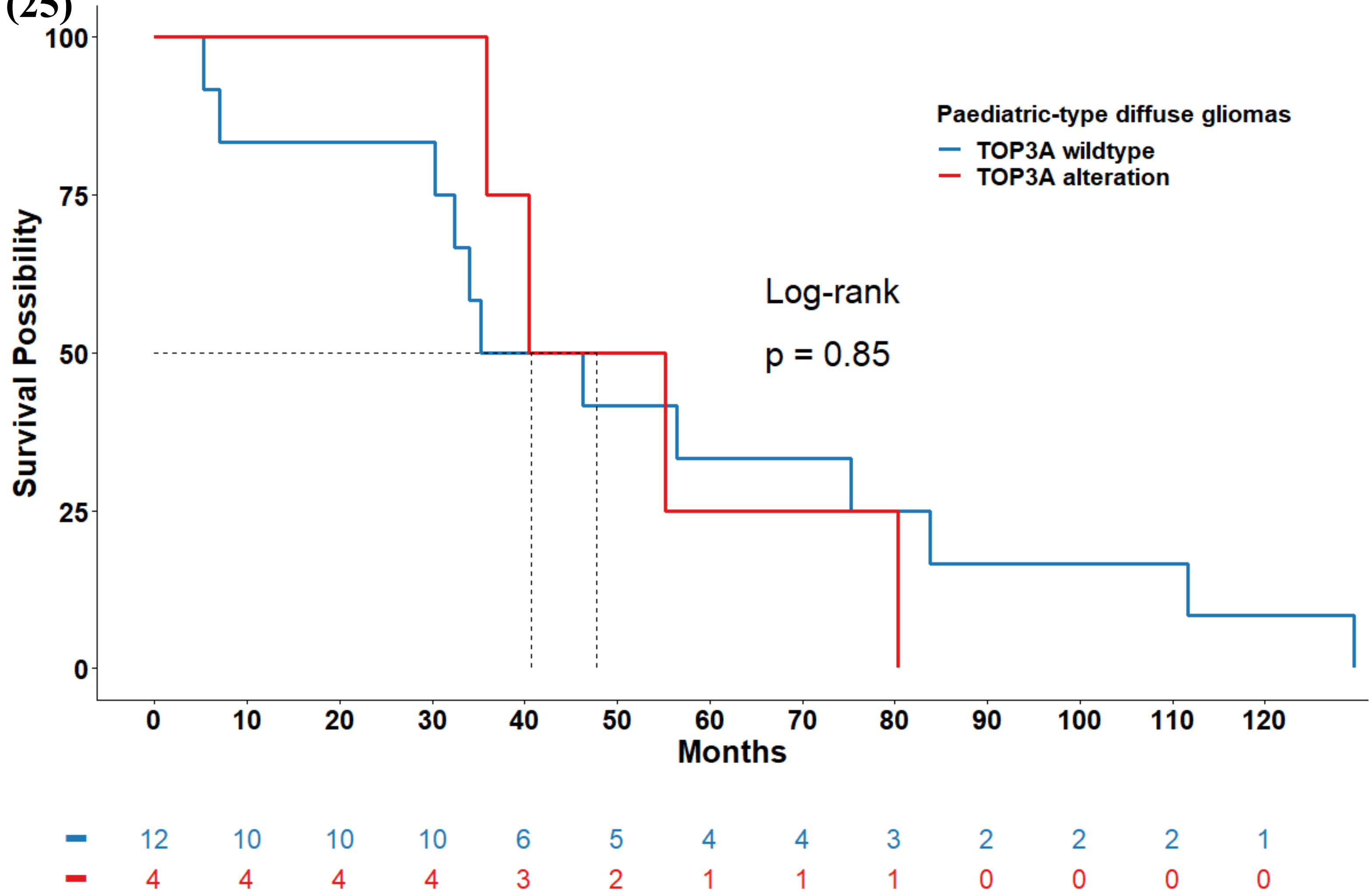

Paediatric-type diffuse gliomas
TOP3A wildtype
TOP3A alteration
Survival Possibility
100
75
50
25
0
Log-rank
p = 0.85
0 10 20 30 40 50 60 70 80 90 100 110 120
Months
12 10 10 10 6 5 4 4 3 2 2 2 1
4 4 4 4 3 2 1 1 1 0 0 0 0

(26)

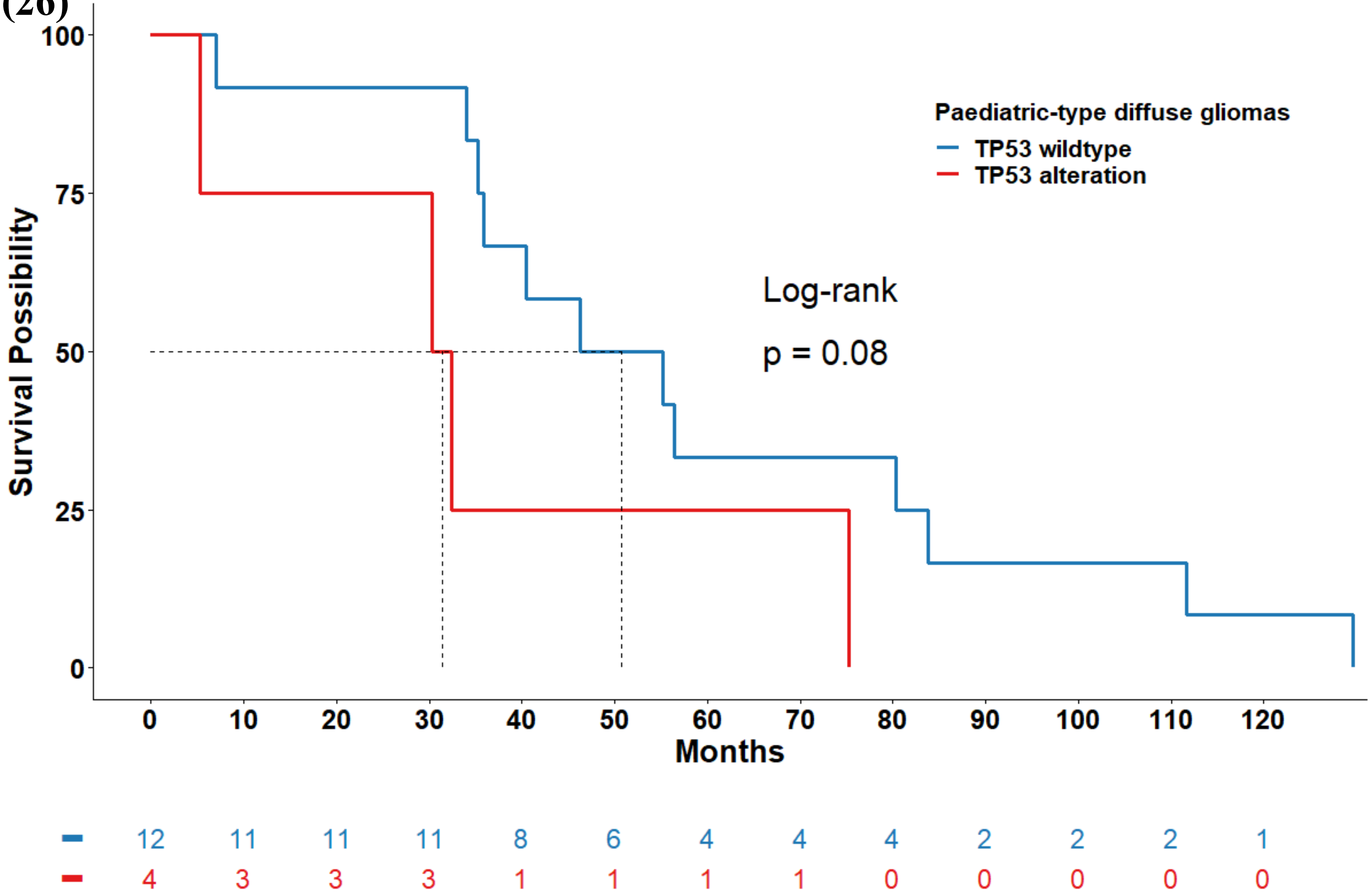

Paediatric-type diffuse gliomas
TP53 wildtype
TP53 alteration
Log-rank
p = 0.08
Survival Possibility
100
75
50
25
0
0
10
20
30
40
50
60
70
80
90
100
110
120
Months
12 11 11 11 8 6 4 4 4 2 2 2 1
4 3 3 3 1 1 1 1 0 0 0 0 0

(27)

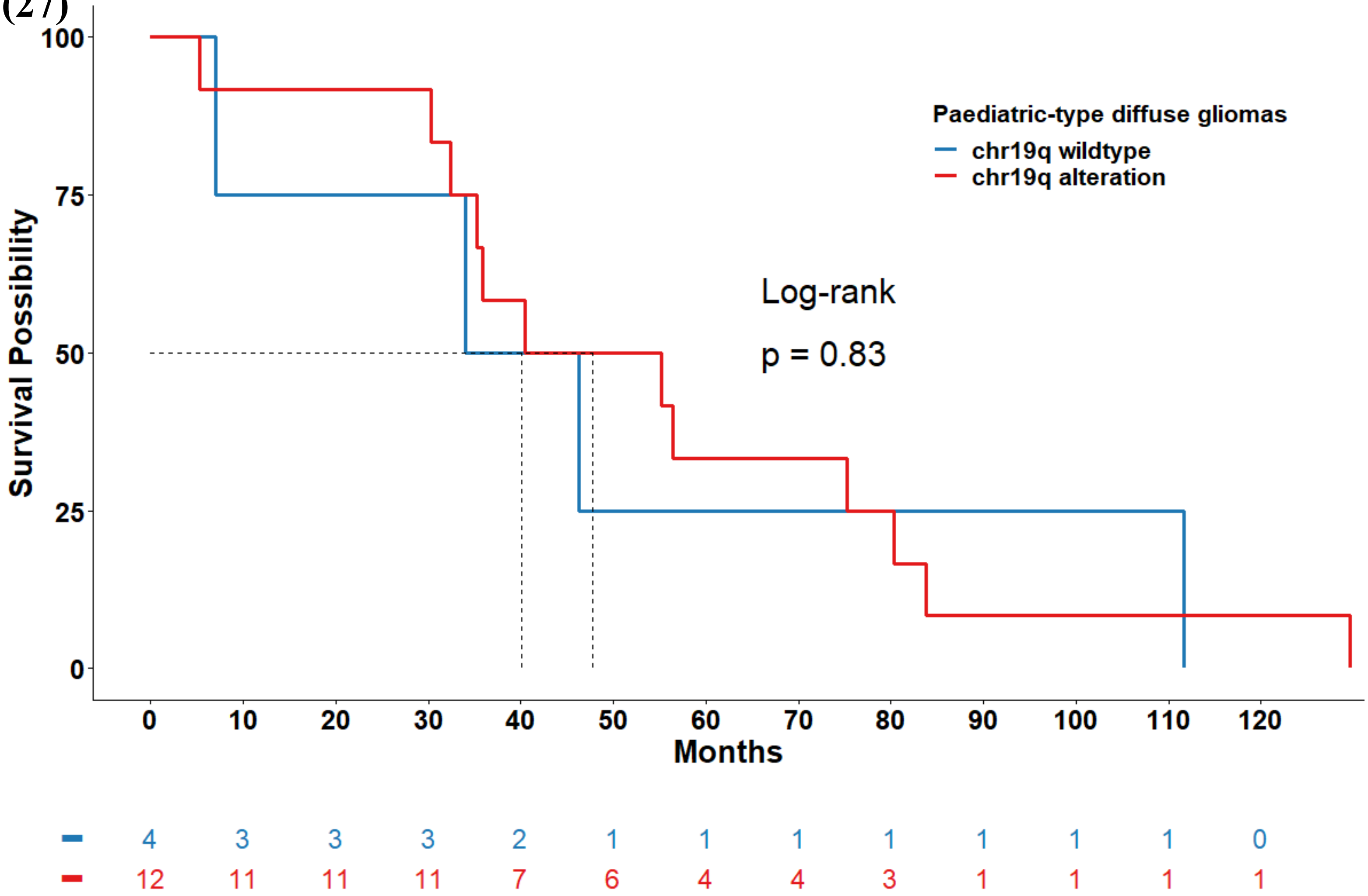
Survival Possibility
100
75
50
25
0
Paediatric-type diffuse gliomas
chr19q wildtype
chr19q alteration
Log-rank
p = 0.83
0 10 20 30 40 50 60 70 80 90 100 110 120
Months
4 3 3 3 2 1 1 1 1 1 1 1 0
12 11 11 11 7 6 4 4 3 1 1 1 1
