# Supplementary material for: Clinical updates on gliomas and implications of the 5th edition of the WHO classification of central nervous system tumors
Source: Front Oncol. 2023 Mar 14;13:1131642. doi: 10.3389/fonc.2023.1131642 (PMC10043404; doi:10.3389/fonc.2023.1131642)
Supplement: Supplementary file 6 [file DataSheet_6.pdf]

Supplement Figure 6. Kaplan–Meier curves showing no significant effects of certain genetic and immunohistochemical alterations on the overall survival of patients with glioblastoma (1-27). Since the numbers of patients divided into two groups (e.g. BRAF wildtype/ BRAF alteration) using different parameters varied, we only enrolled the parameters using which the number of patients in either group was above 3. Parameters used in this section were shown as follows.

(1) BRAF 1
(2) CDK6 2
(3) FGFR1 3
(4) FGFR2 4
(5) FGFR4 5
(6) KIT 6
(7) KRAS 7
(8) MET 8
(9) MYB 9
(10) MYBL1 10
(11) MYC 11
(12) MYCN 12
(13) NF1 13
(14) NOTCH1 14
(15) NTRK2 15
(16) NTRK3 16
(17) PDGFRA 17
(18) PEG3 18
(19) PIK3CA 19
(20) PPM1D 20
(21) PTEN 21
(22) PTPN11 22
(23) RB1 23
(24) TOP3A 24
(25) PIK3R1 25
(26) TSC2 26
(27) chr17 27

(1)

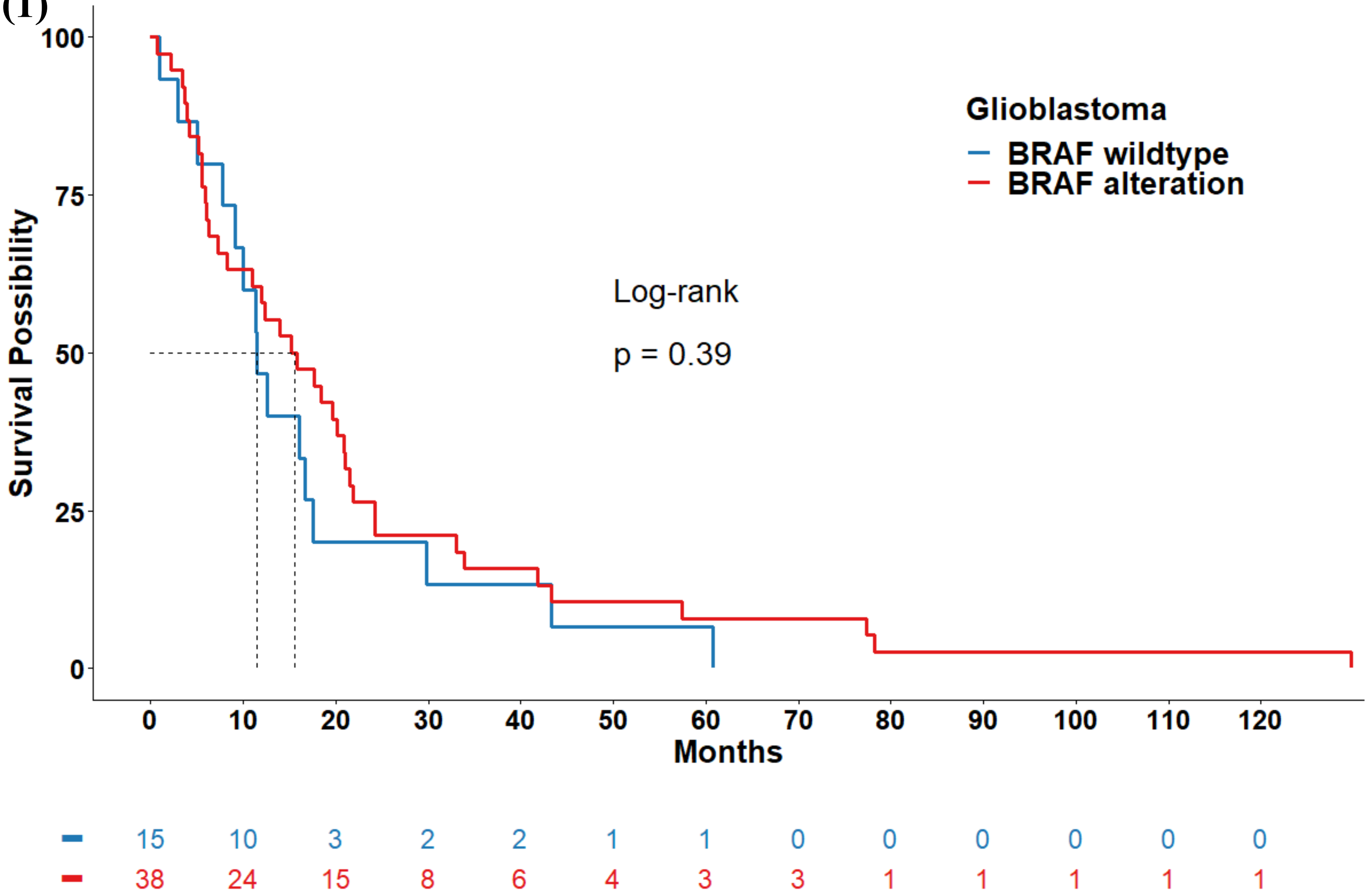

Glioblastoma
BRAF wildtype
BRAF alteration
Log-rank
p = 0.39
Survival Possibility
100
75
50
25
0
0 10 20 30 40 50 60 70 80 90 100 110 120
Months
15 10 3 2 2 1 1 0 0 0 0 0 0
38 24 15 8 6 4 3 3 1 1 1 1 1

(2)

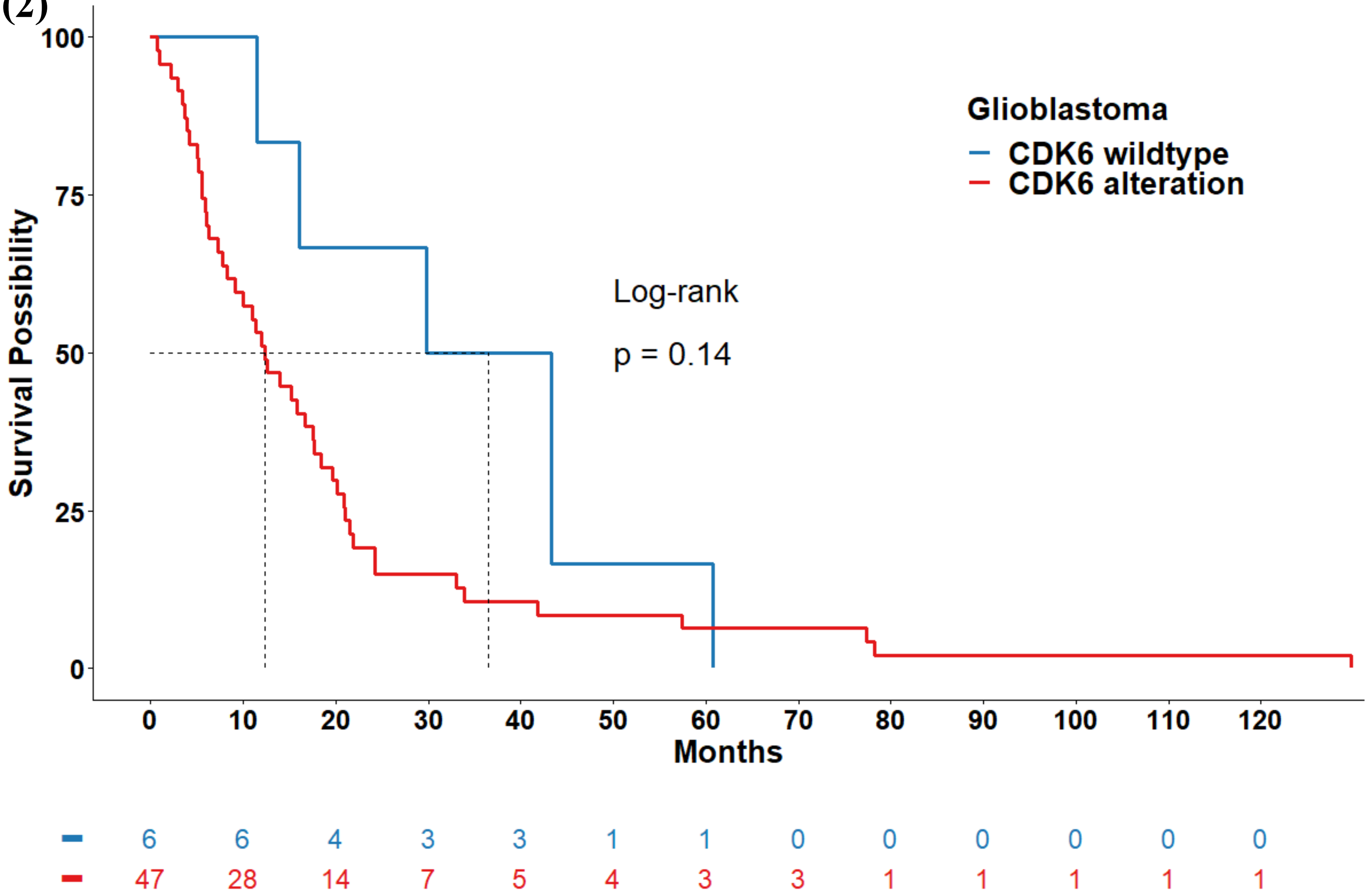
Survival Possibility
100
75
50
25
0
Glioblastoma
CDK6 wildtype
CDK6 alteration
Log-rank
p = 0.14
0
10
20
30
40
50
60
70
80
90
100
110
120
Months
6 6 4 3 3 1 1 0 0 0 0 0 0
47 28 14 7 5 4 3 3 1 1 1 1 1

(3)

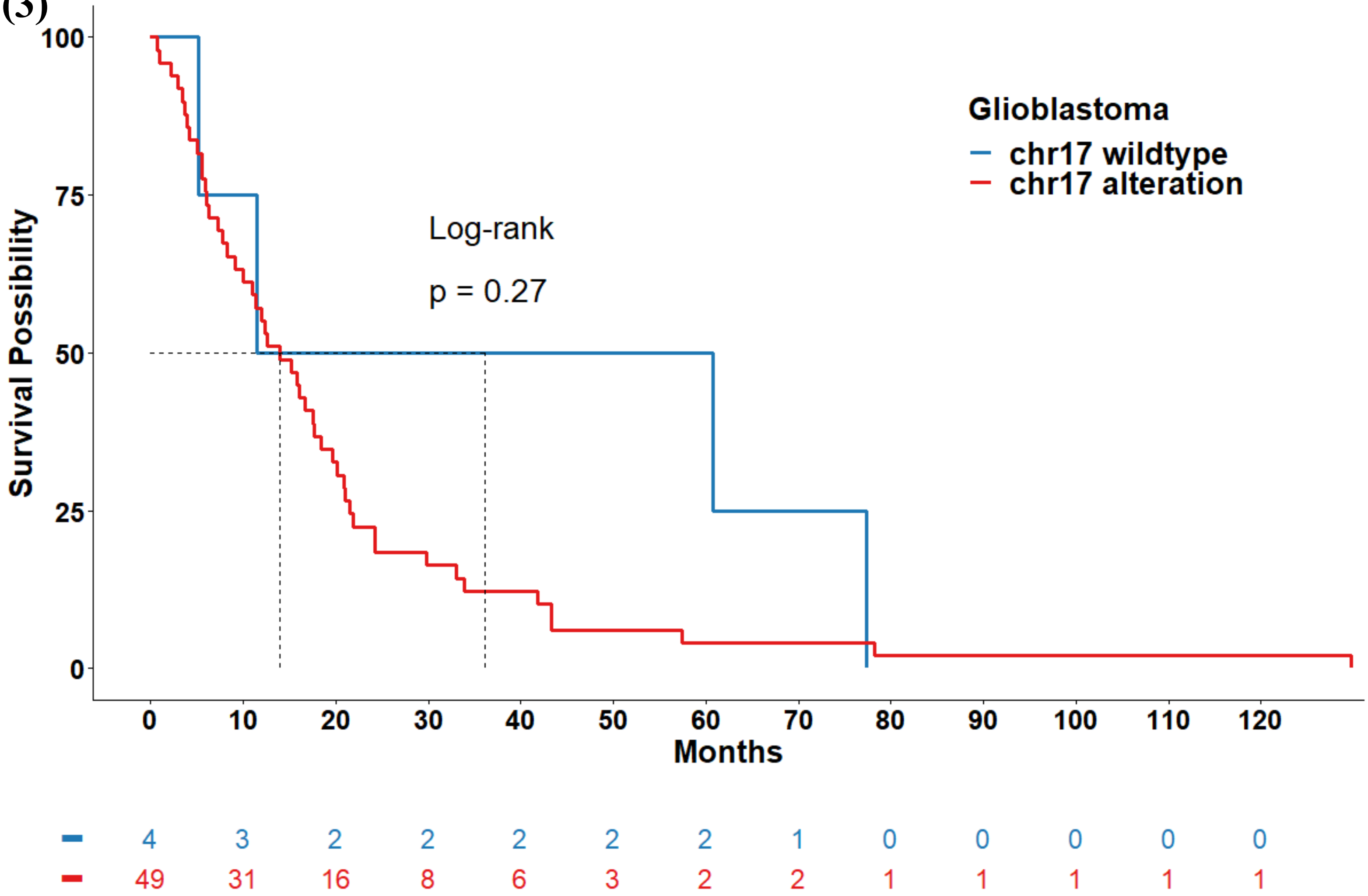

Survival Possibility
100
75
50
25
0
Log-rank
p = 0.27
Glioblastoma
chr17 wildtype
chr17 alteration
0
10
20
30
40
50
60
70
80
90
100
110
120
Months
4 3 2 2 2 2 2 1 0 0 0 0 0
49 31 16 8 6 3 2 2 1 1 1 1 1

(4)

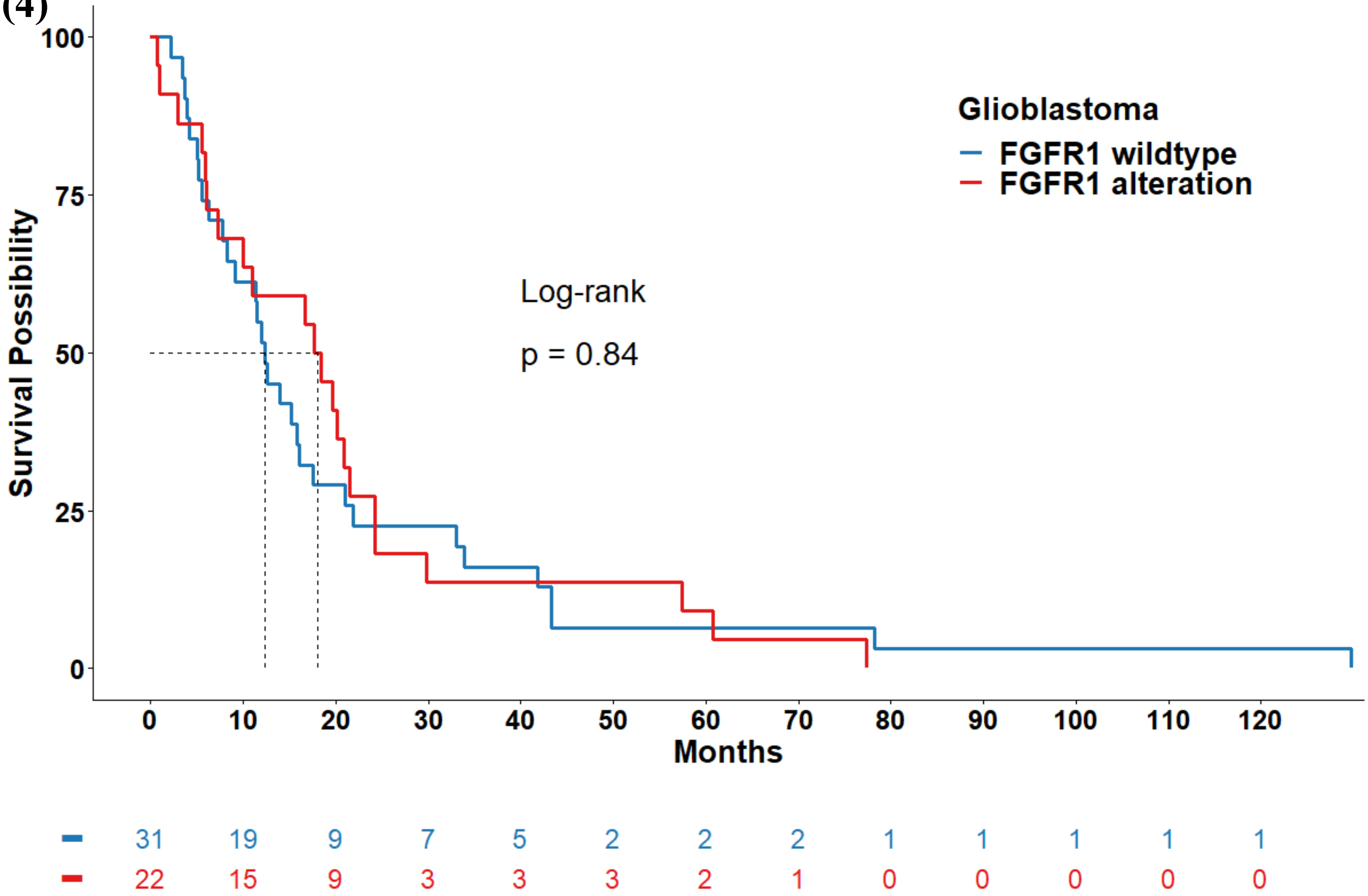
Glioblastoma
FGFR1 wildtype
FGFR1 alteration
Log-rank
p = 0.84
Survival Possibility
100
75
50
25
0
0 10 20 30 40 50 60 70 80 90 100 110 120
Months
31 19 9 7 5 2 2 2 1 1 1 1 1
22 15 9 3 3 3 2 1 0 0 0 0 0

(5)

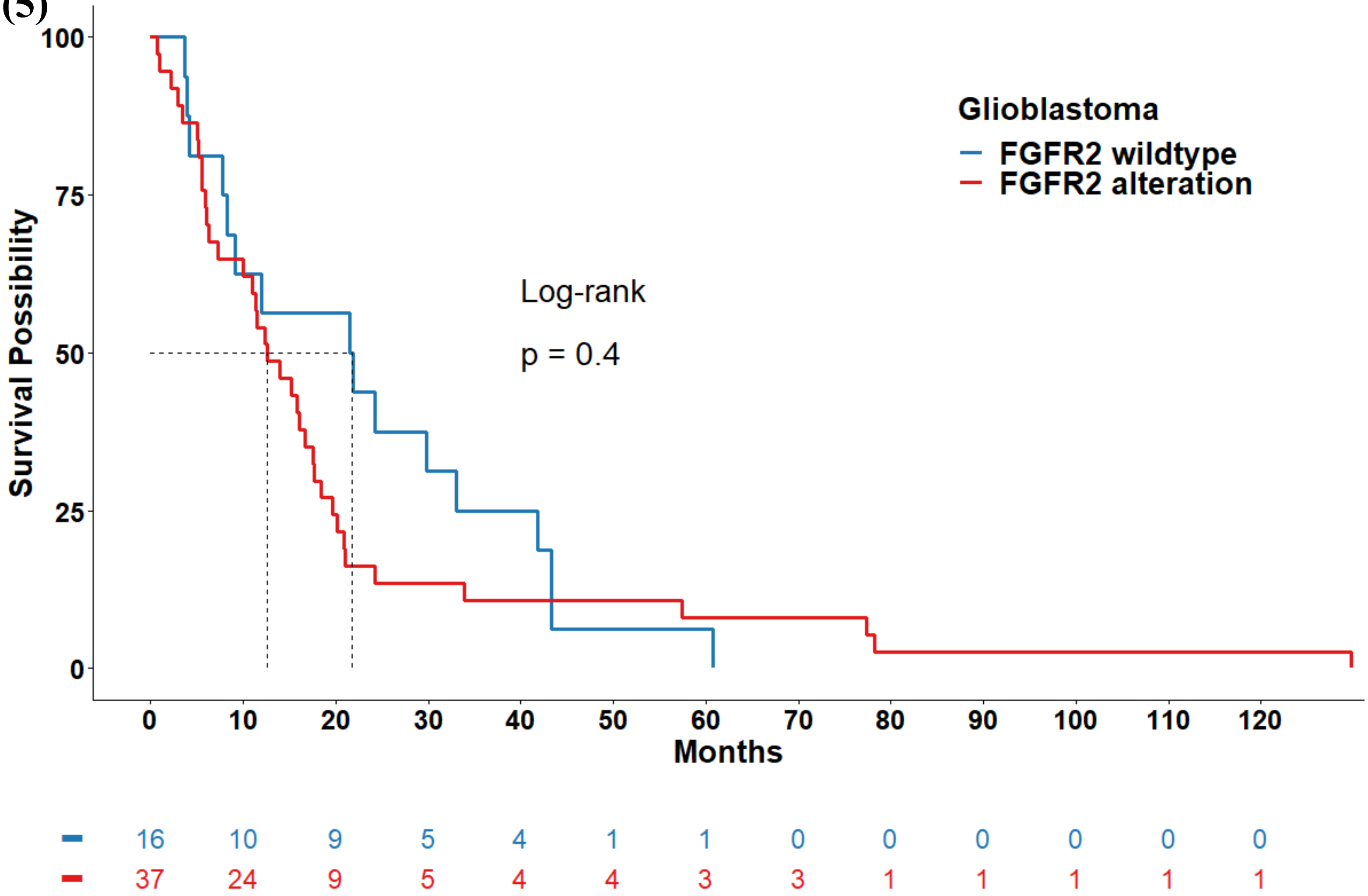

Survival Possibility
100
75
50
25
0
Log-rank
p = 0.4
Glioblastoma
FGFR2 wildtype
FGFR2 alteration
0
10
20
30
40
50
60
70
80
90
100
110
120
Months
16 10 9 5 4 1 1 0 0 0 0 0 0
37 24 9 5 4 4 3 3 1 1 1 1 1

(6)

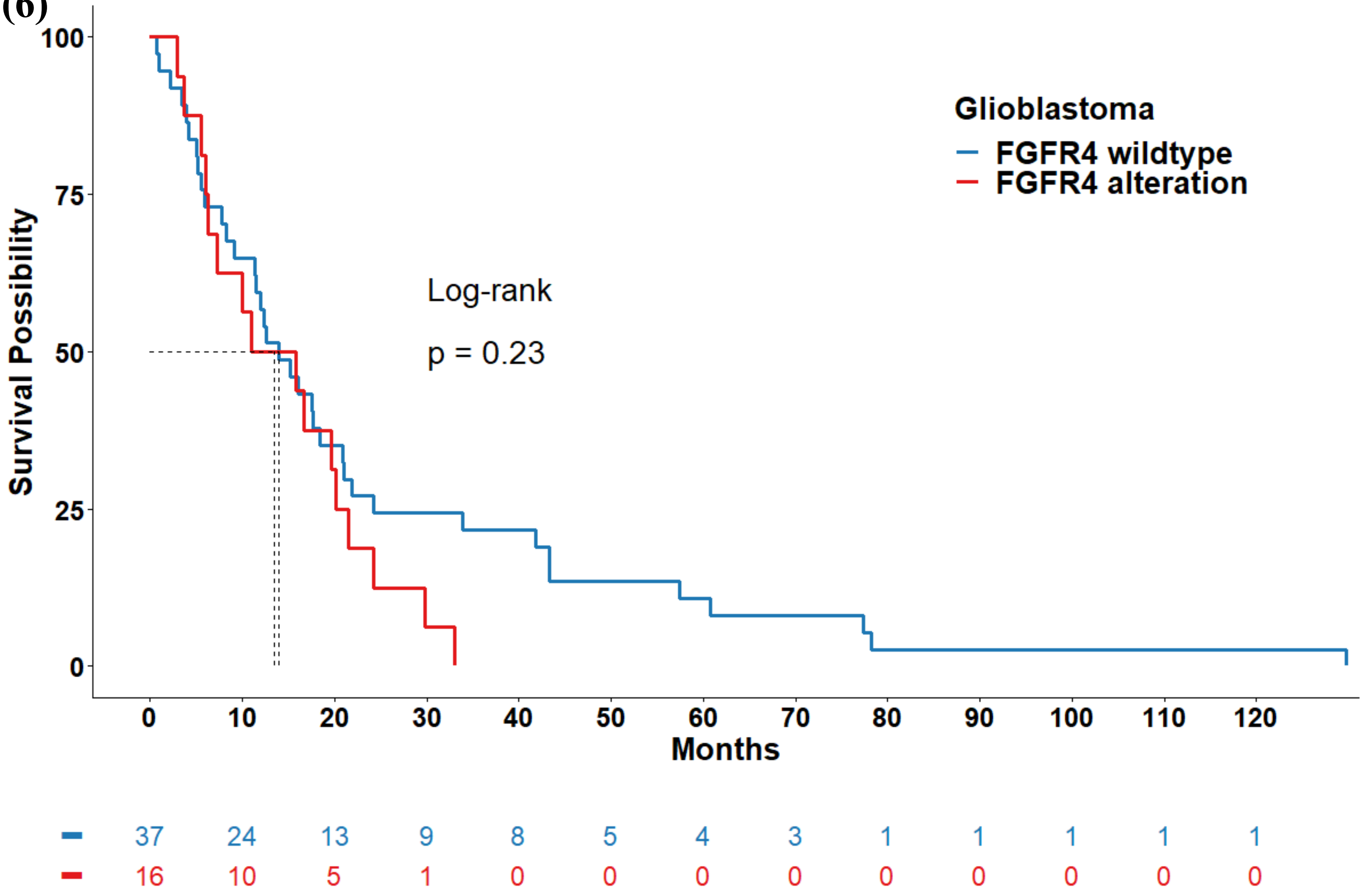

Survival Possibility
100
75
50
25
0
Log-rank
p = 0.23
Glioblastoma
FGFR4 wildtype
FGFR4 alteration
0 10 20 30 40 50 60 70 80 90 100 110 120
Months
37 24 13 9 8 5 4 3 1 1 1 1 1
16 10 5 1 0 0 0 0 0 0 0 0 0

(7)

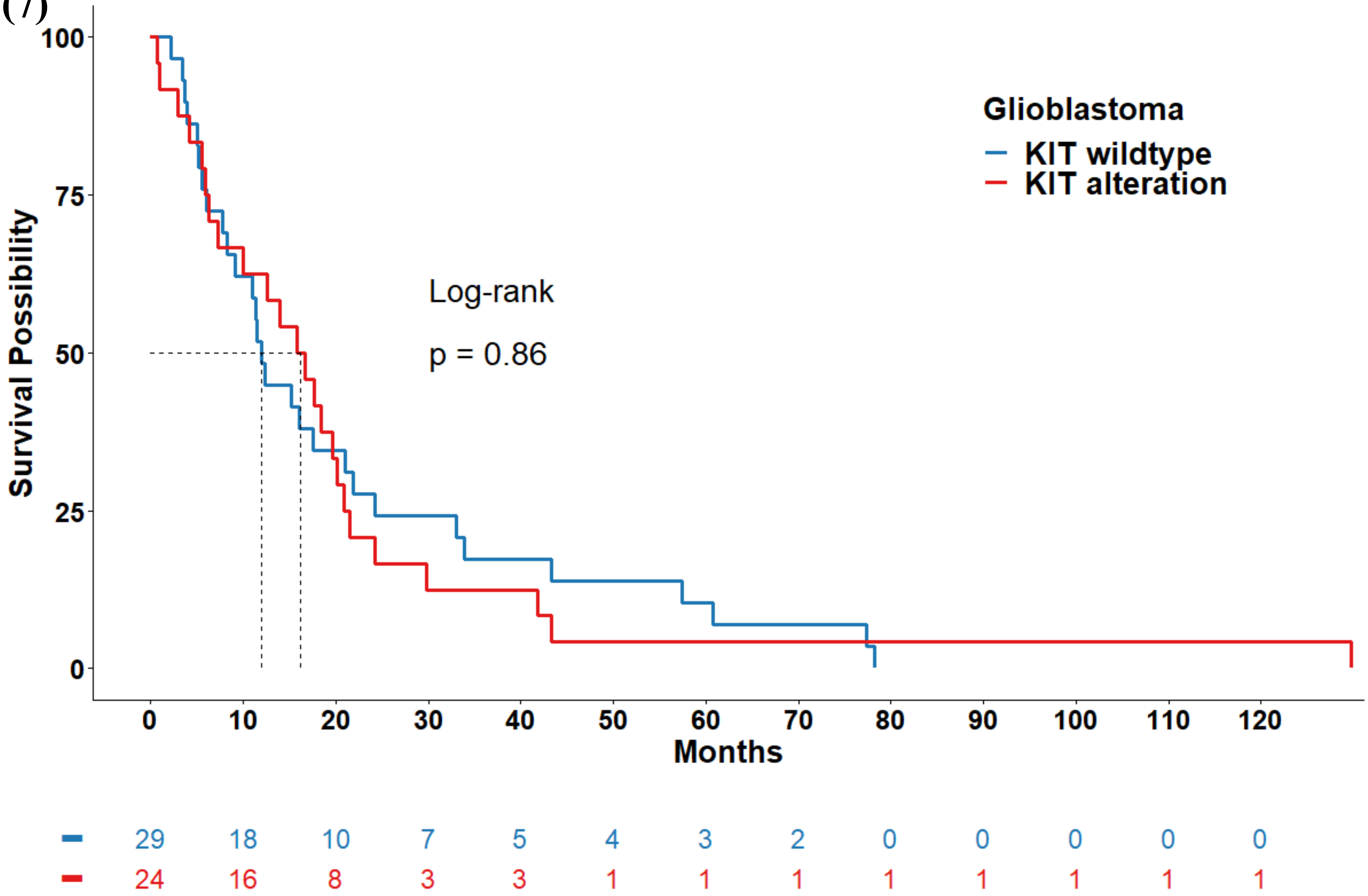

Survival Possibility
100
75
50
25
0
0 10 20 30 40 50 60 70 80 90 100 110 120
Months
Glioblastoma
KIT wildtype
KIT alteration
Log-rank
p = 0.86
29 18 10 7 5 4 3 2 0 0 0 0 0
24 16 8 3 3 1 1 1 1 1 1 1 1

(8)

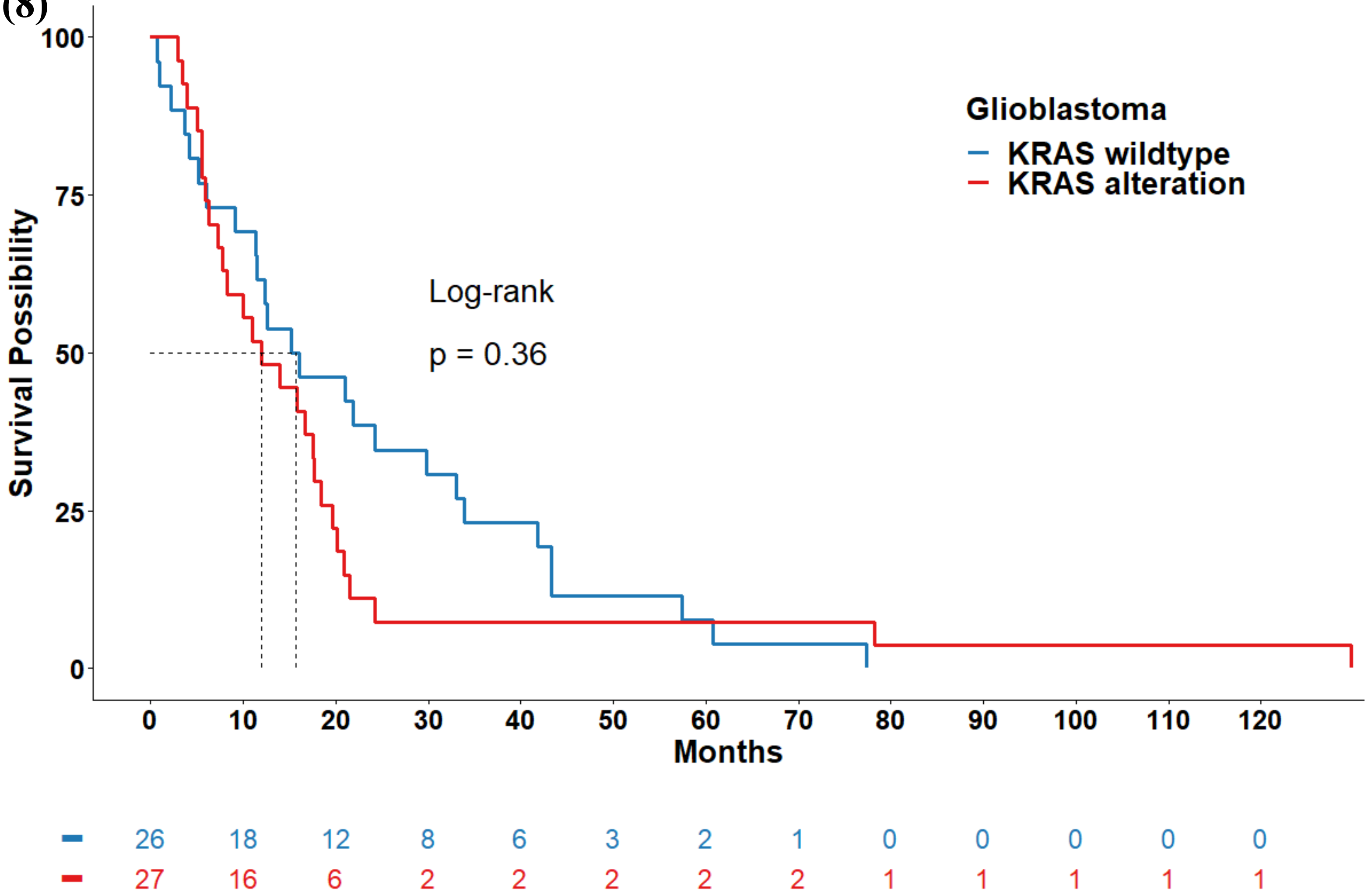

100
75
50
25
0
Survival Possibility
Log-rank
p = 0.36
Glioblastoma
KRAS wildtype
KRAS alteration
0
10
20
30
40
50
60
70
80
90
100
110
120
Months
26 18 12 8 6 3 2 1 0 0 0 0 0
27 16 6 2 2 2 2 2 1 1 1 1 1

(9)

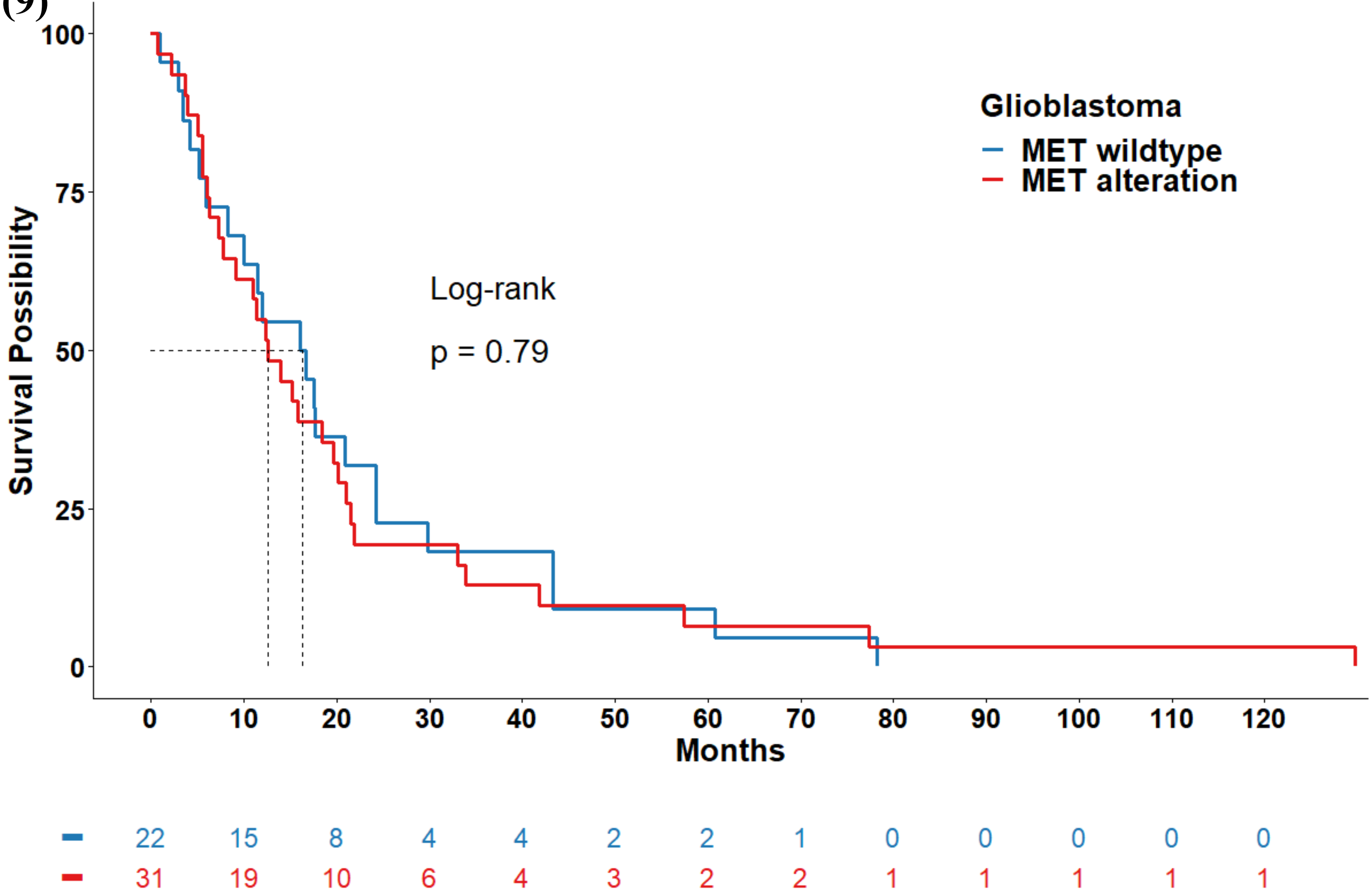

Glioblastoma
MET wildtype
MET alteration
Log-rank
p = 0.79
Survival Possibility
100
75
50
25
0
0
10
20
30
40
50
60
70
80
90
100
110
120
Months
22 15 8 4 4 2 2 1 0 0 0 0 0
31 19 10 6 4 3 2 2 1 1 1 1 1

(10)

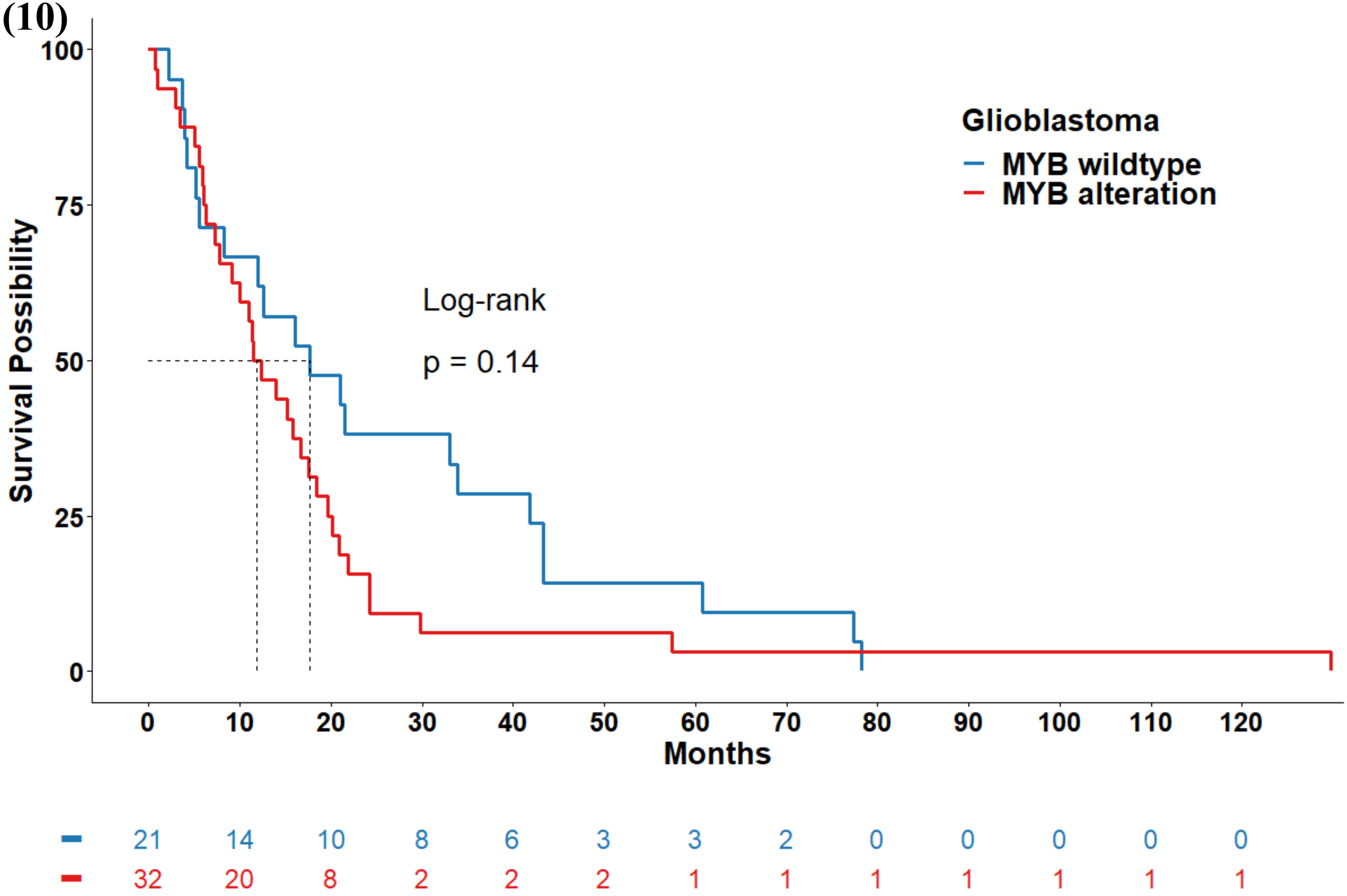

Glioblastoma
MYB wildtype
MYB alteration
Log-rank
p = 0.14
Survival Possibility
100
75
50
25
0
0
10
20
30
40
50
60
70
80
90
100
110
120
Months
21 14 10 8 6 3 3 2 0 0 0 0 0
32 20 8 2 2 2 1 1 1 1 1 1 1

(11)

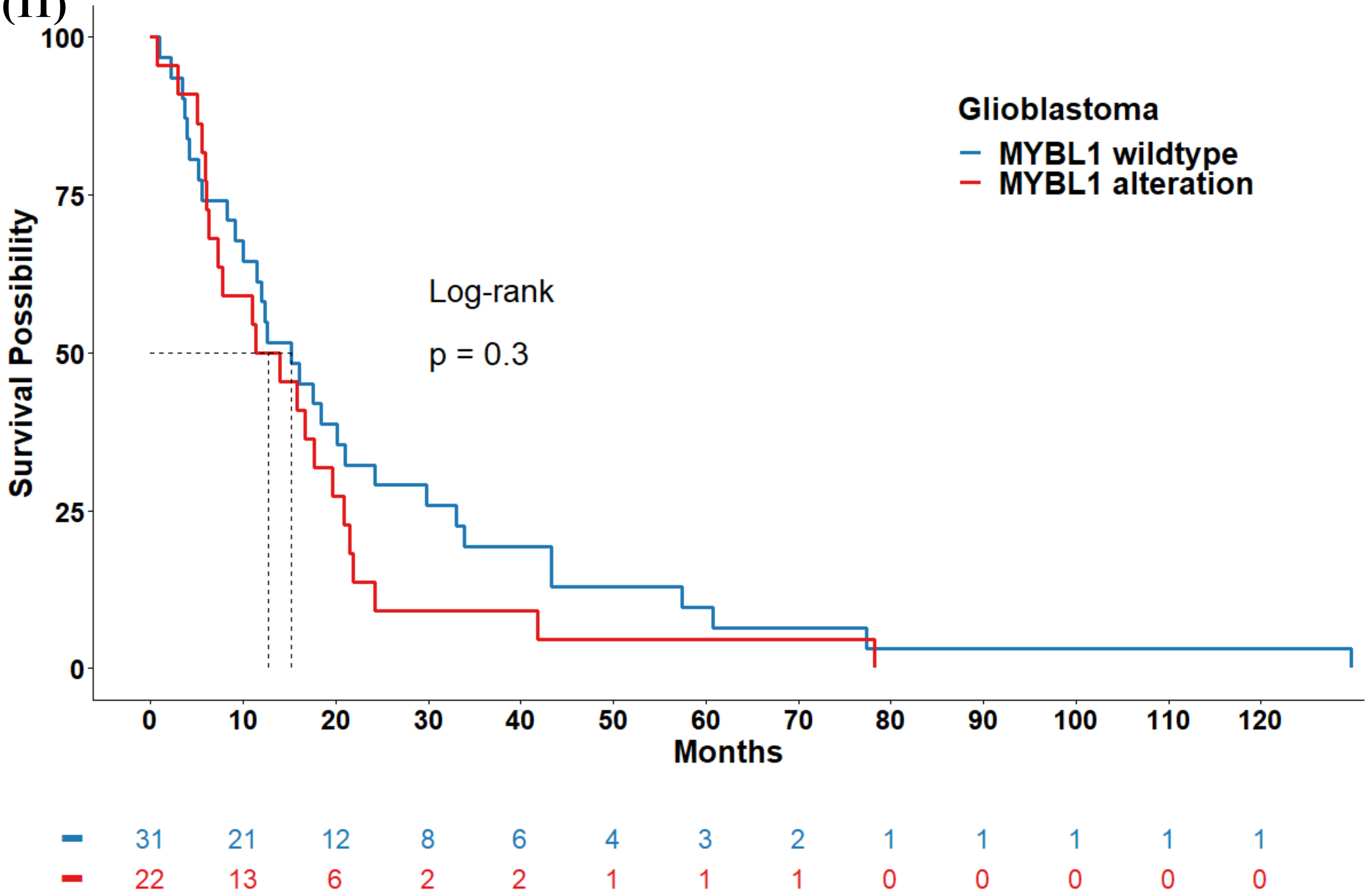
Glioblastoma
MYBL1 wildtype
MYBL1 alteration
Log-rank
p = 0.3
Survival Possibility
100
75
50
25
0
0 10 20 30 40 50 60 70 80 90 100 110 120
Months
31 21 12 8 6 4 3 2 1 1 1 1 1
22 13 6 2 2 1 1 1 0 0 0 0 0

(12)

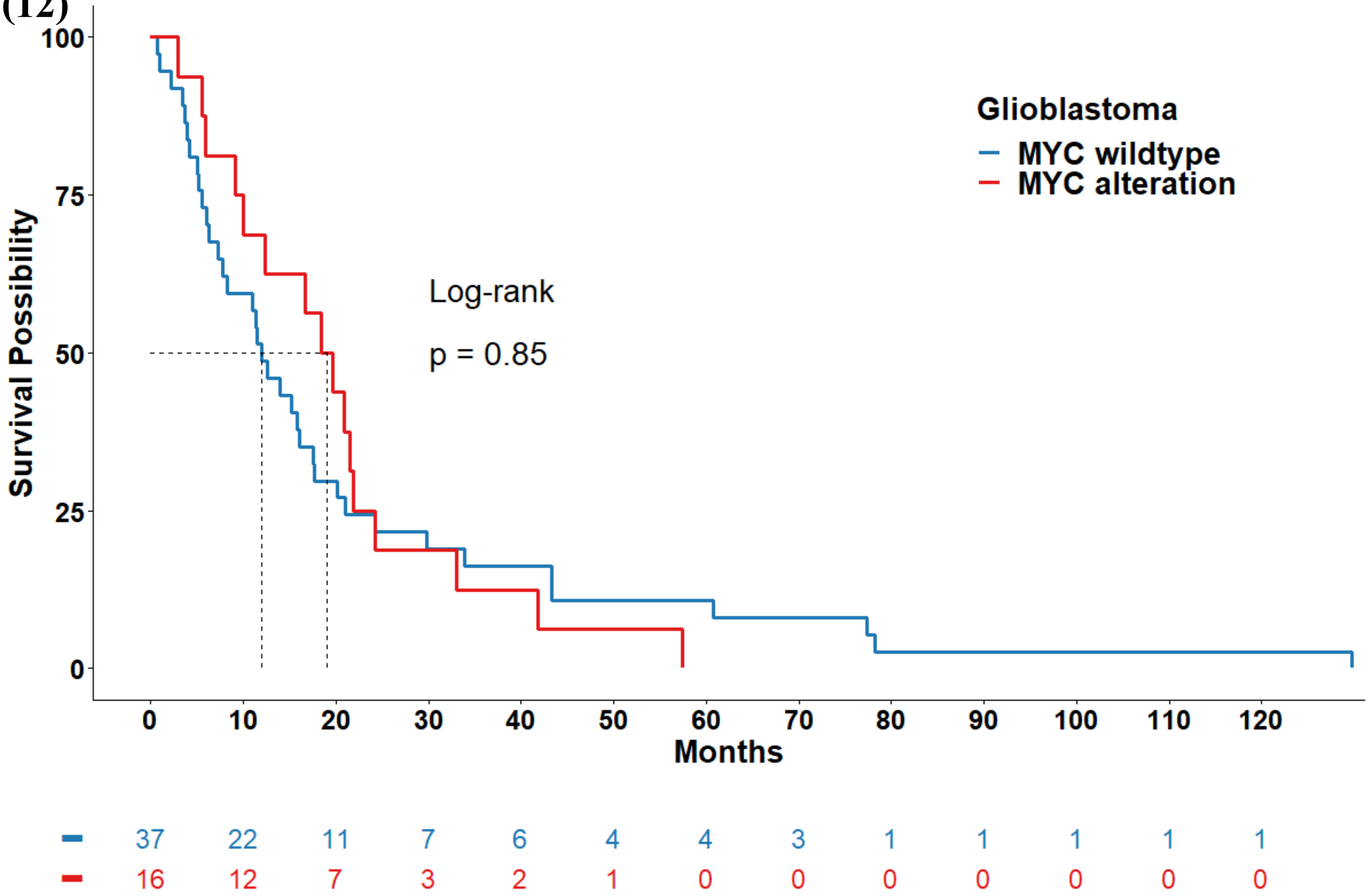

Glioblastoma
MYC wildtype
MYC alteration
Log-rank
p = 0.85
Survival Possibility
100
75
50
25
0
0 10 20 30 40 50 60 70 80 90 100 110 120
Months
37 22 11 7 6 4 4 3 1 1 1 1 1
16 12 7 3 2 1 0 0 0 0 0 0 0

(13)

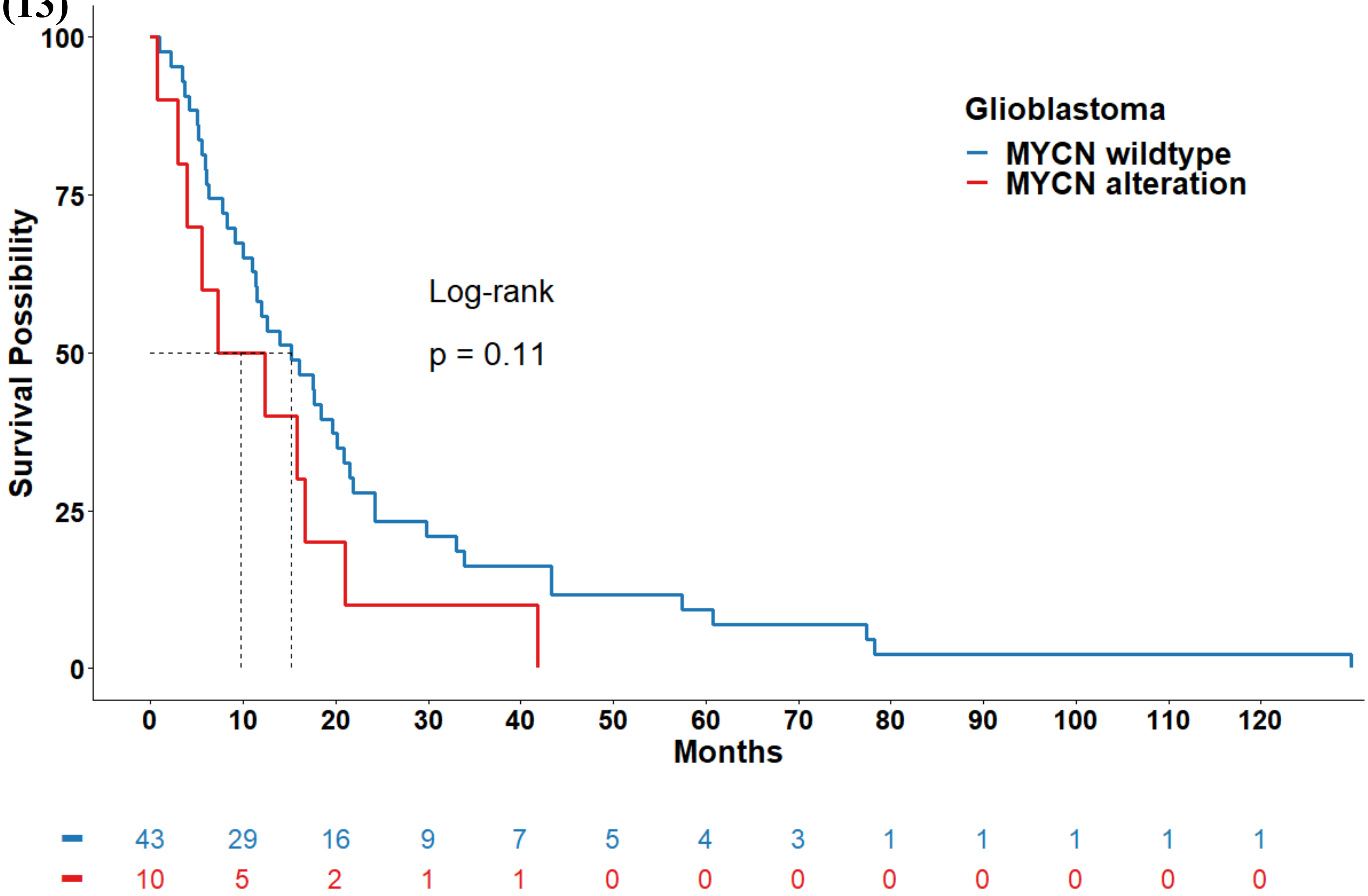

Glioblastoma
MYCN wildtype
MYCN alteration
Log-rank
p = 0.11
Survival Possibility
100
75
50
25
0
0 10 20 30 40 50 60 70 80 90 100 110 120
Months
43 29 16 9 7 5 4 3 1 1 1 1 1
10 5 2 1 1 0 0 0 0 0 0 0 0

(14)

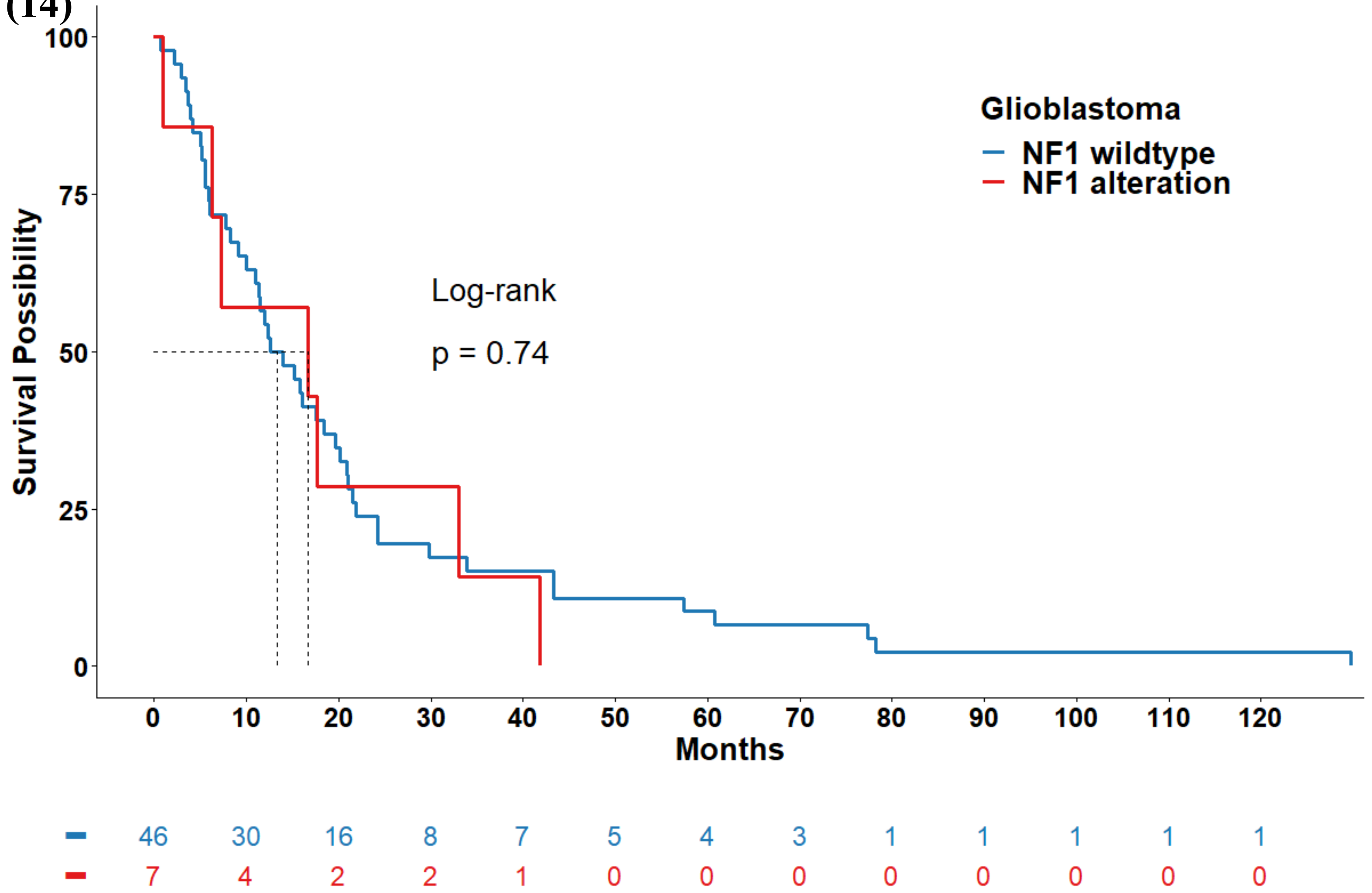

Glioblastoma
NF1 wildtype
NF1 alteration
Log-rank
p = 0.74
Survival Possibility
100
75
50
25
0
0 10 20 30 40 50 60 70 80 90 100 110 120
Months
46 30 16 8 7 5 4 3 1 1 1 1 1
7 4 2 2 1 0 0 0 0 0 0 0 0

(15)

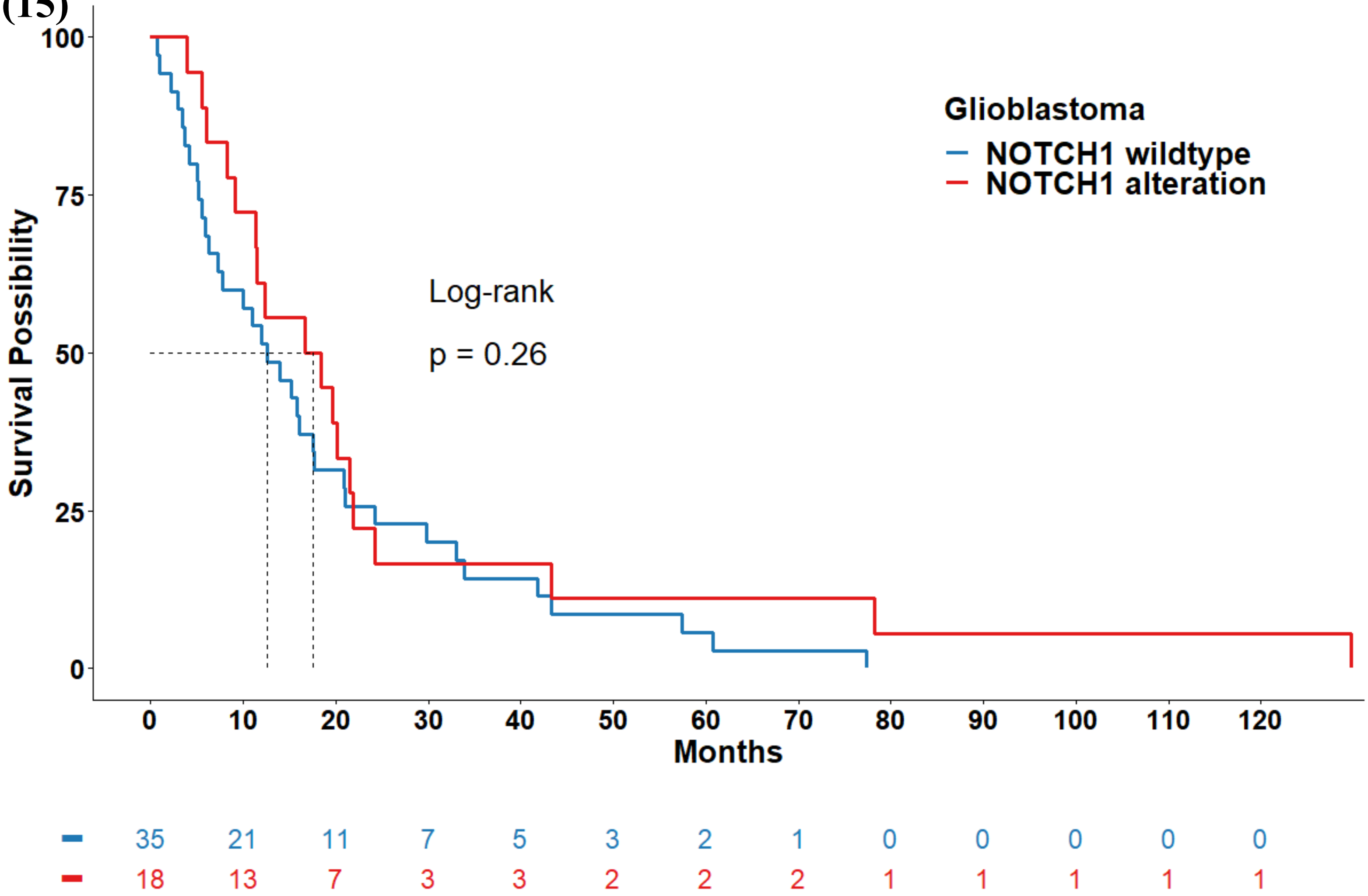
Survival Possibility
100
75
50
25
0
Log-rank
p = 0.26
Glioblastoma
NOTCH1 wildtype
NOTCH1 alteration
0 10 20 30 40 50 60 70 80 90 100 110 120
Months
35 21 11 7 5 3 2 1 0 0 0 0 0
18 13 7 3 3 2 2 2 1 1 1 1 1

(16)

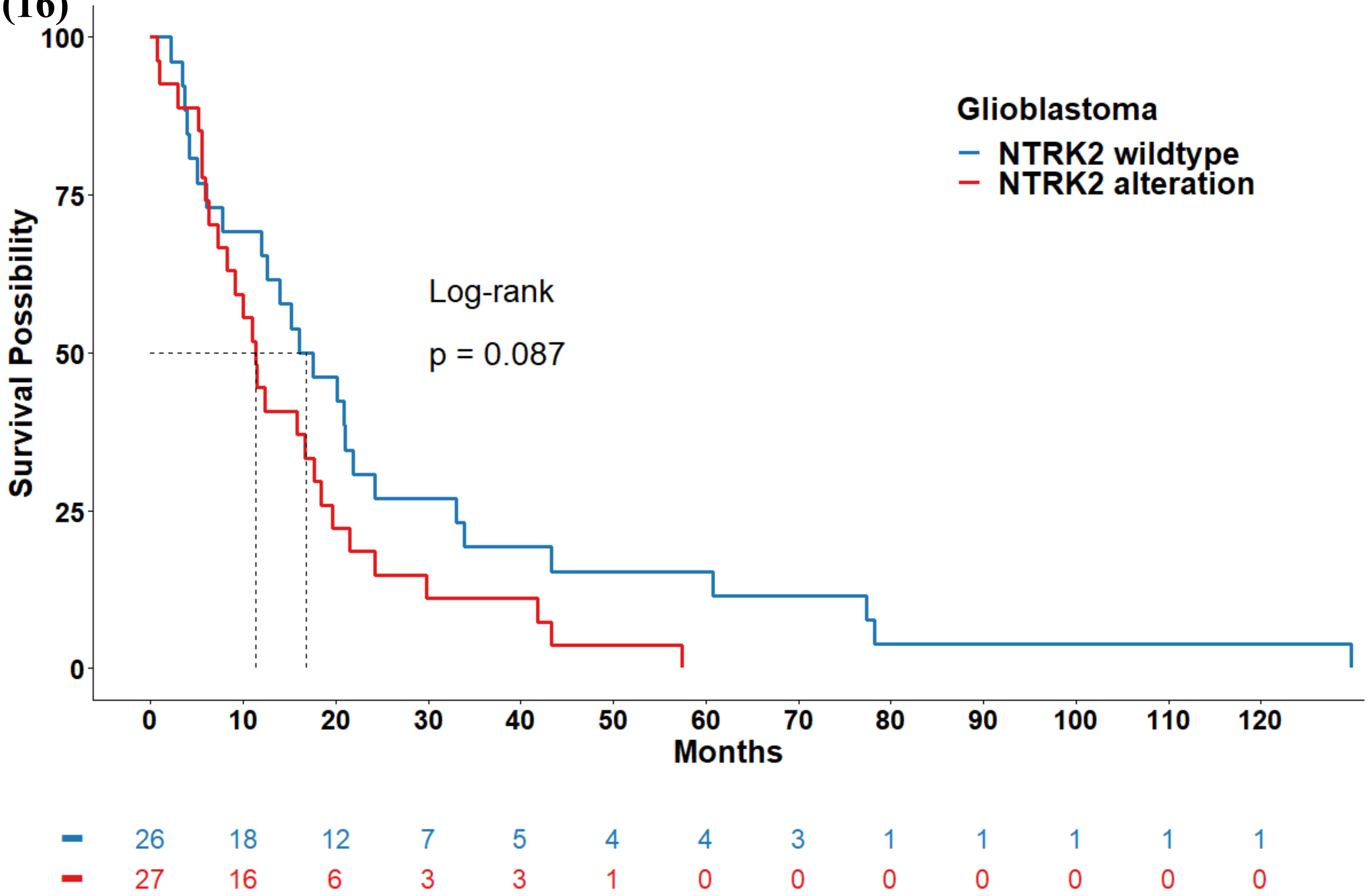

Survival Possibility
100
75
50
25
0
Log-rank
p = 0.087
Glioblastoma
NTRK2 wildtype
NTRK2 alteration
0
10
20
30
40
50
60
70
80
90
100
110
120
Months
26 18 12 7 5 4 4 3 1 1 1 1 1
27 16 6 3 3 1 0 0 0 0 0 0 0

(17)

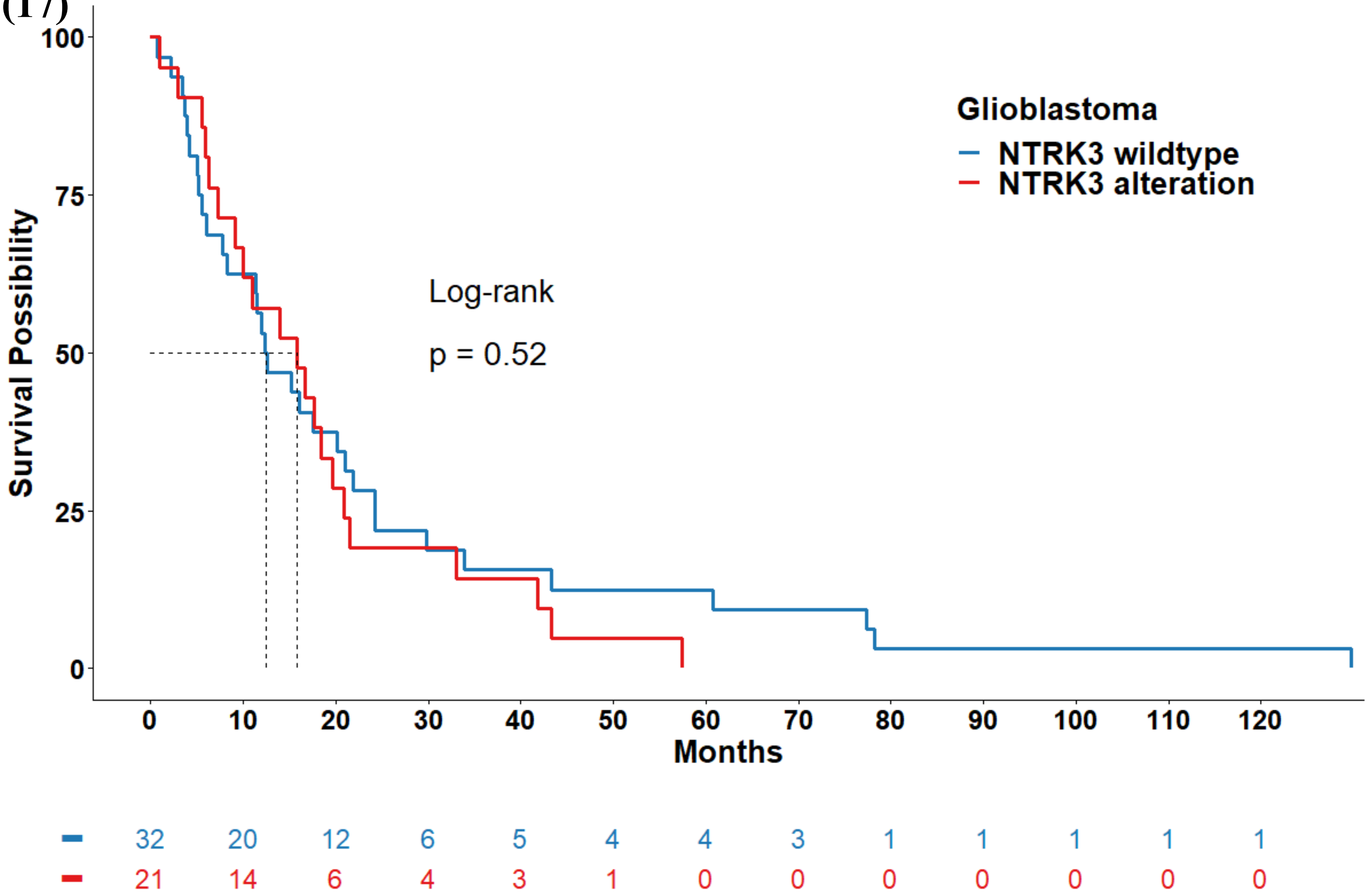

Glioblastoma
NTRK3 wildtype
NTRK3 alteration
Log-rank
p = 0.52
Survival Possibility
100
75
50
25
0
0 10 20 30 40 50 60 70 80 90 100 110 120
Months
32 20 12 6 5 4 4 3 1 1 1 1 1
21 14 6 4 3 1 0 0 0 0 0 0 0

(18)

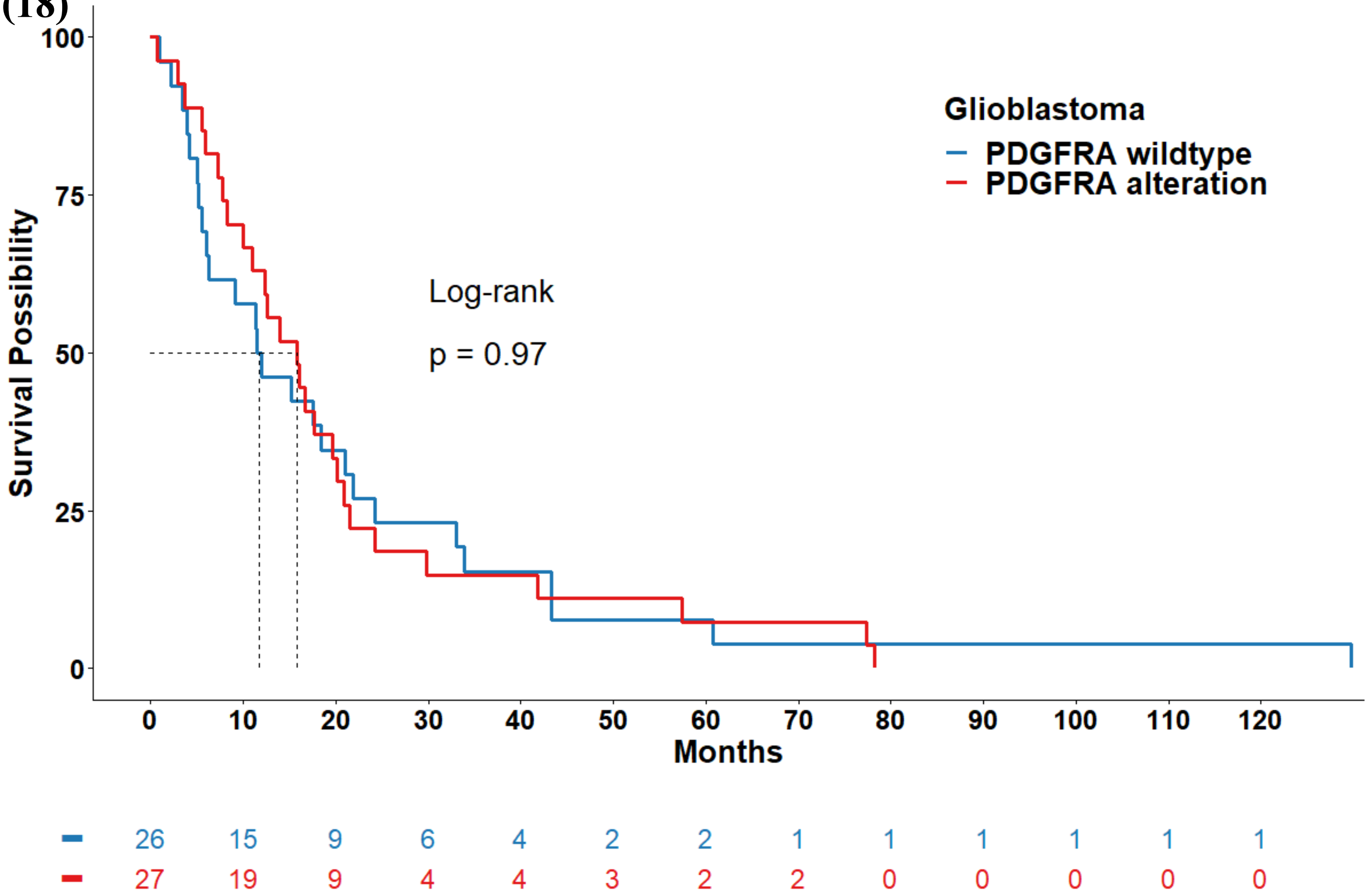

Glioblastoma
PDGFRA wildtype
PDGFRA alteration
Log-rank
p = 0.97
Survival Possibility
100
75
50
25
0
0
10
20
30
40
50
60
70
80
90
100
110
120
Months
26 15 9 6 4 2 2 1 1 1 1 1 1
27 19 9 4 4 3 2 2 0 0 0 0 0

(19)

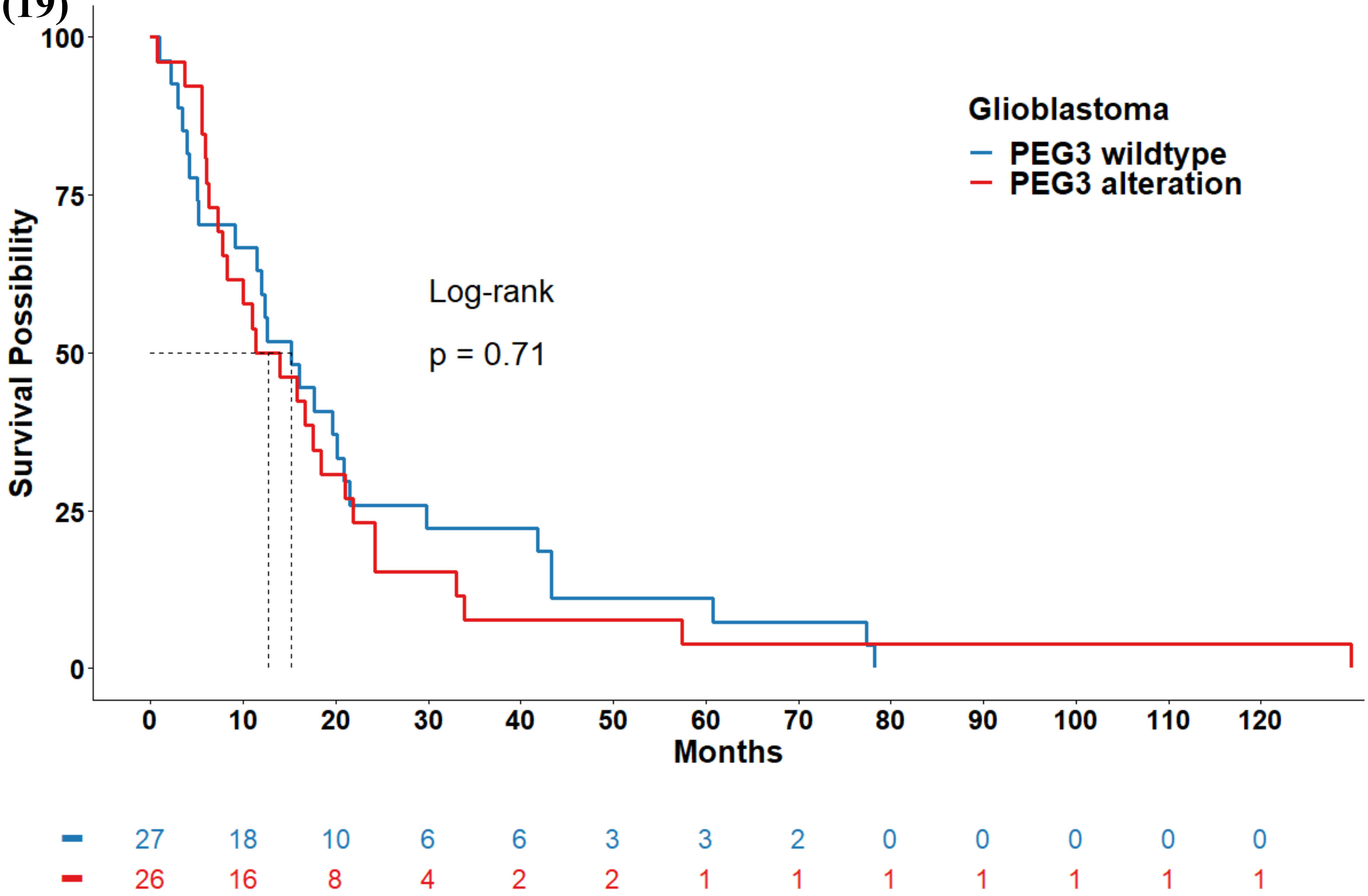

Survival Possibility
100
75
50
25
0
Log-rank
p = 0.71
Glioblastoma
PEG3 wildtype
PEG3 alteration
0
10
20
30
40
50
60
70
80
90
100
110
120
Months
27 18 10 6 6 3 3 2 0 0 0 0 0
26 16 8 4 2 2 1 1 1 1 1 1 1

(20)

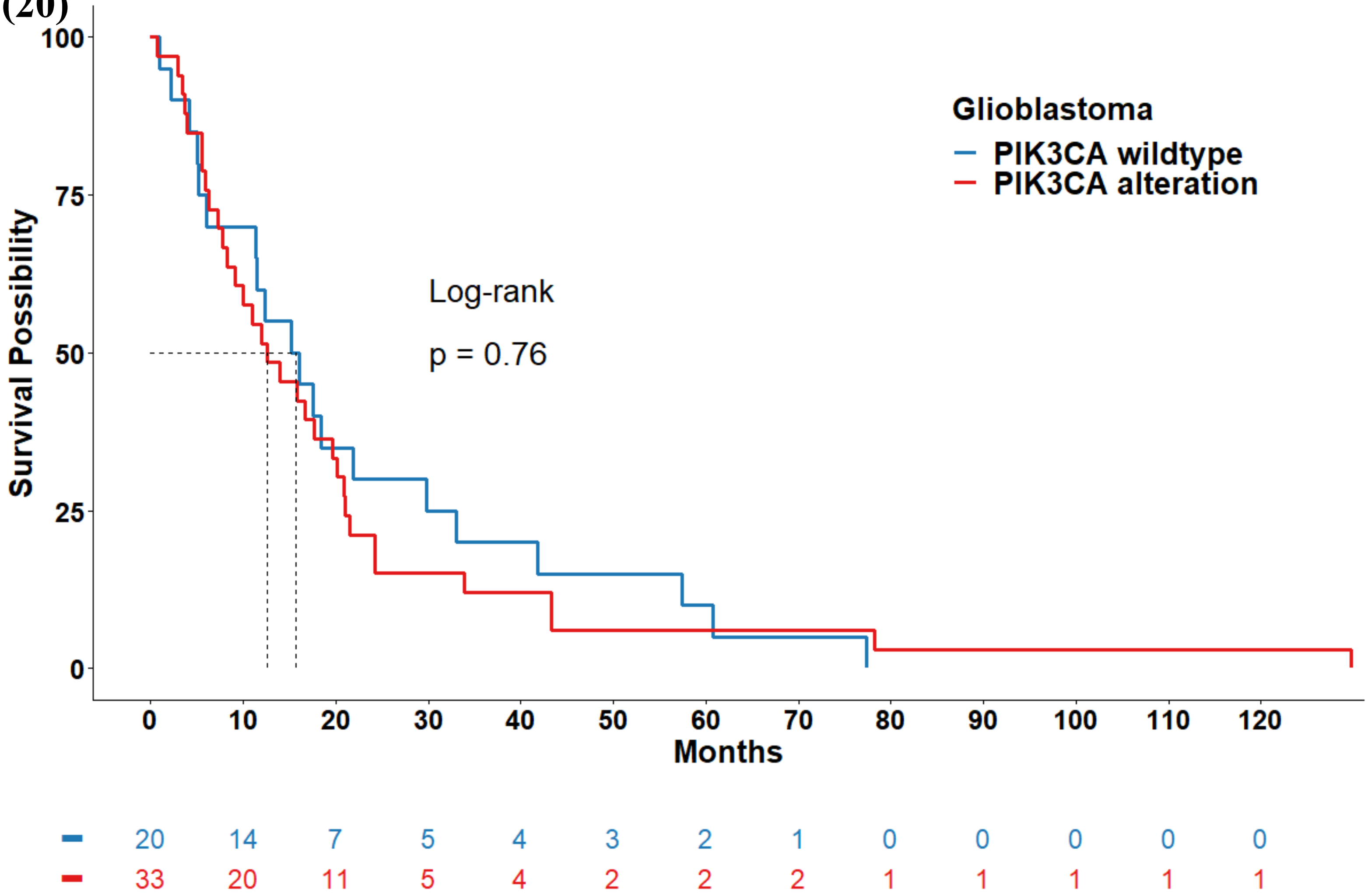
Glioblastoma
PIK3CA wildtype
PIK3CA alteration
Log-rank
p = 0.76
Survival Possibility
100
75
50
25
0
0 10 20 30 40 50 60 70 80 90 100 110 120
Months
20 14 7 5 4 3 2 1 0 0 0 0 0
33 20 11 5 4 2 2 2 1 1 1 1 1

(21)

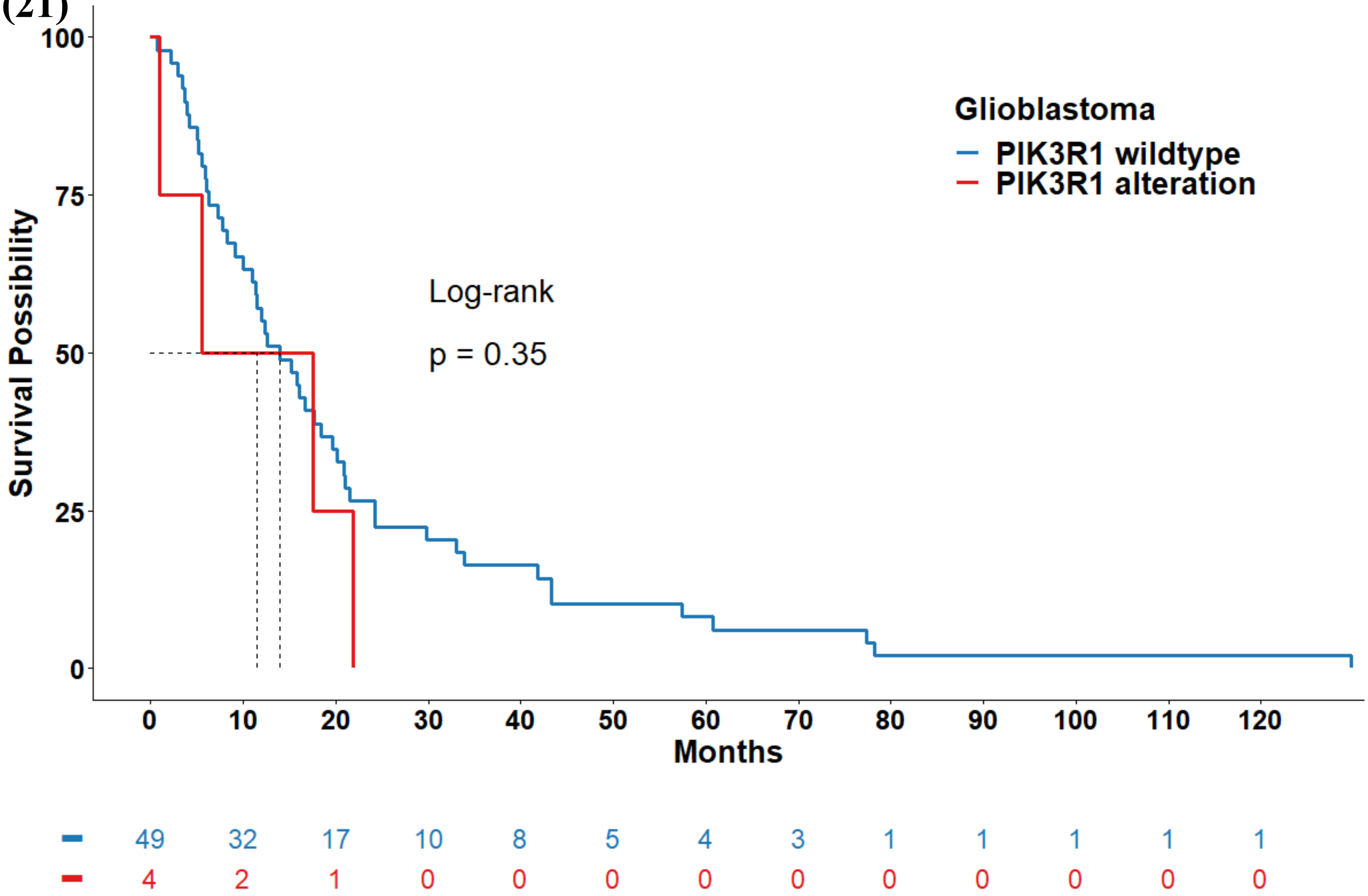
Survival Possibility
100
75
50
25
0
Log-rank
p = 0.35
Glioblastoma
PIK3R1 wildtype
PIK3R1 alteration
0
10
20
30
40
50
60
70
80
90
100
110
120
Months
49 32 17 10 8 5 4 3 1 1 1 1 1
4 2 1 0 0 0 0 0 0 0 0 0 0

(22)

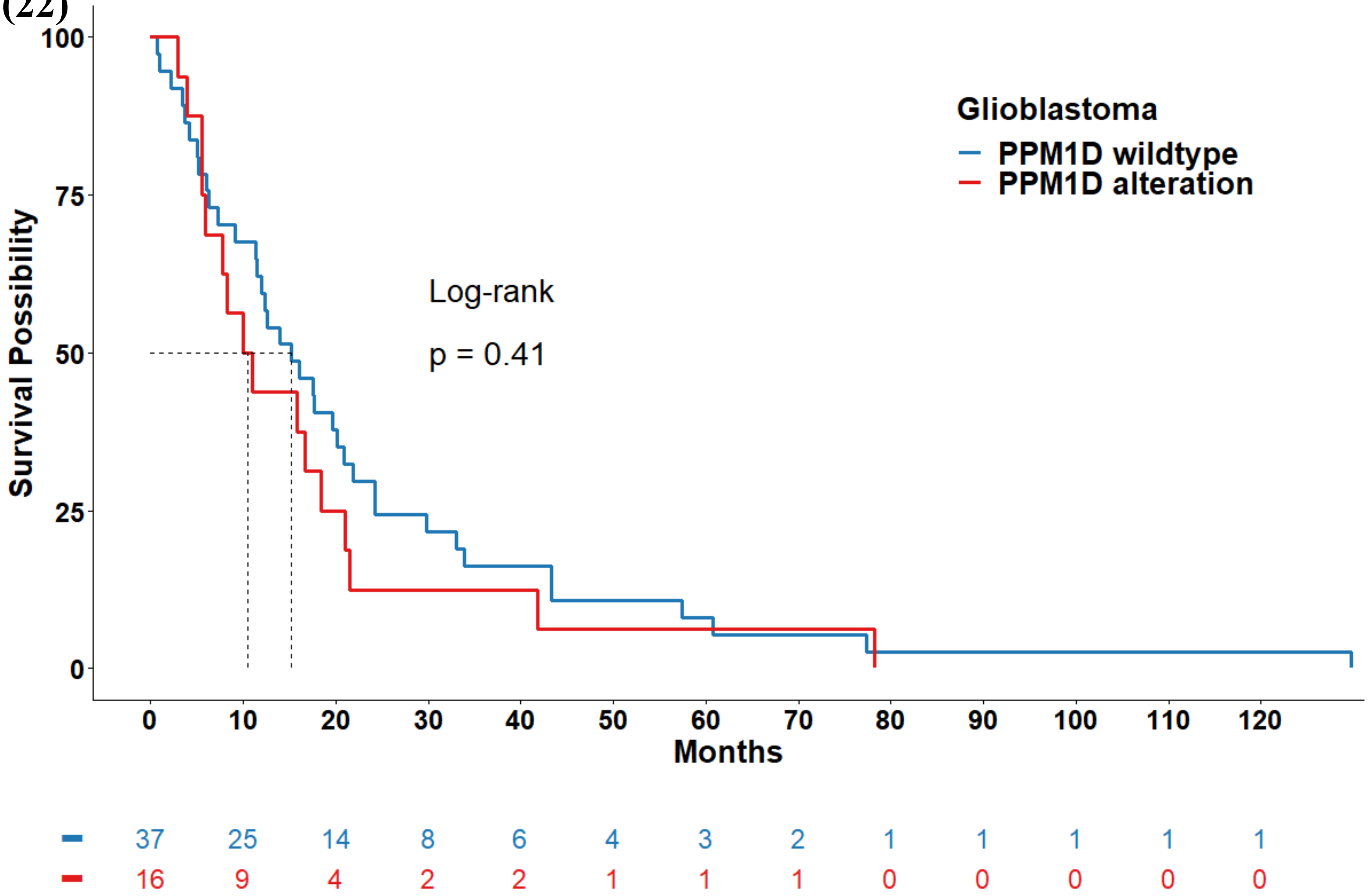

Glioblastoma
PPM1D wildtype
PPM1D alteration
Log-rank
p = 0.41
Survival Possibility
100
75
50
25
0
0 10 20 30 40 50 60 70 80 90 100 110 120
Months
37 25 14 8 6 4 3 2 1 1 1 1 1
16 9 4 2 2 1 1 1 0 0 0 0 0

(23)

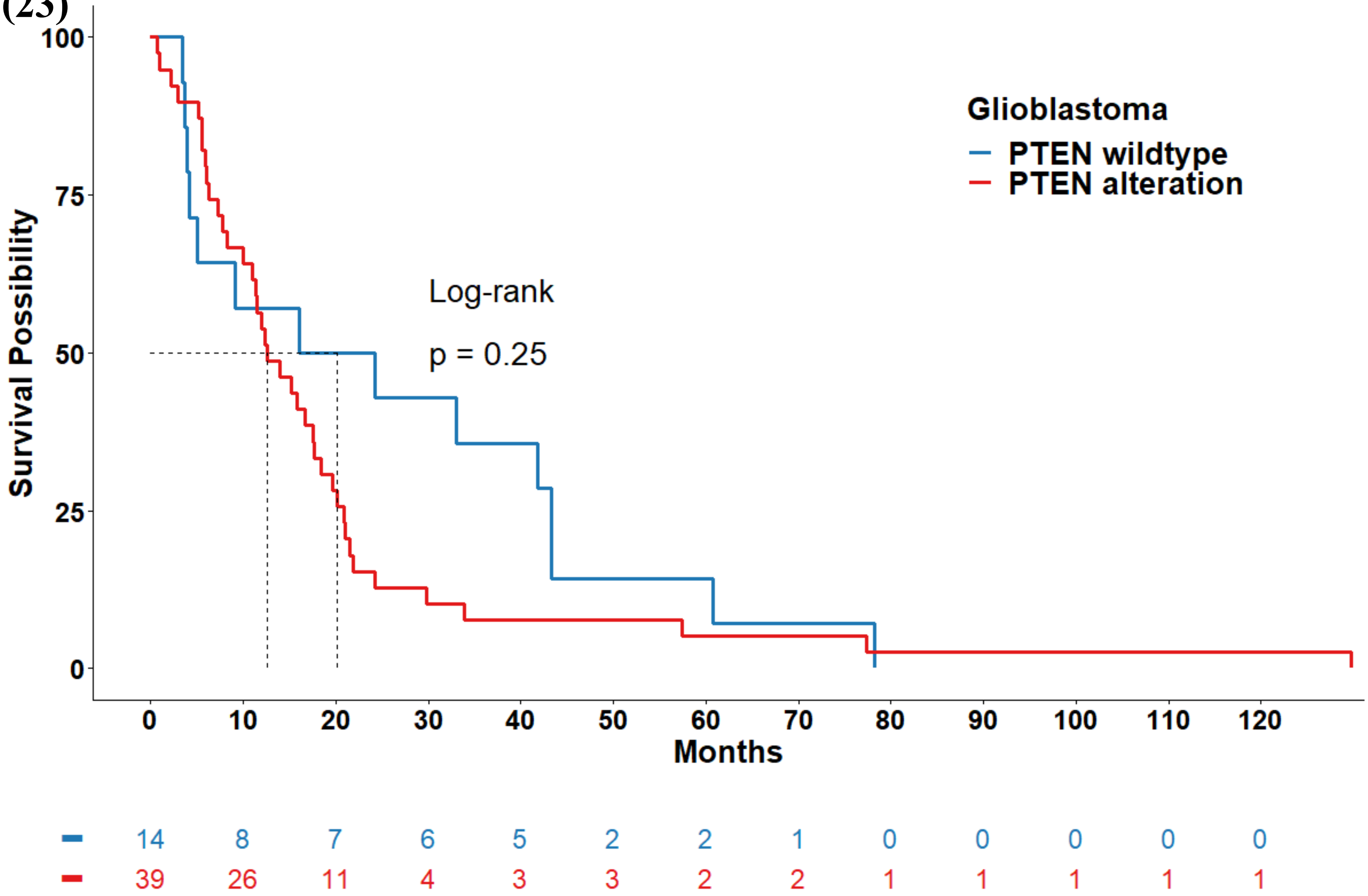
Glioblastoma
PTEN wildtype
PTEN alteration
Log-rank
p = 0.25
Survival Possibility
100
75
50
25
0
0 10 20 30 40 50 60 70 80 90 100 110 120
Months
14 8 7 6 5 2 2 1 0 0 0 0 0
39 26 11 4 3 3 2 2 1 1 1 1 1

(24)

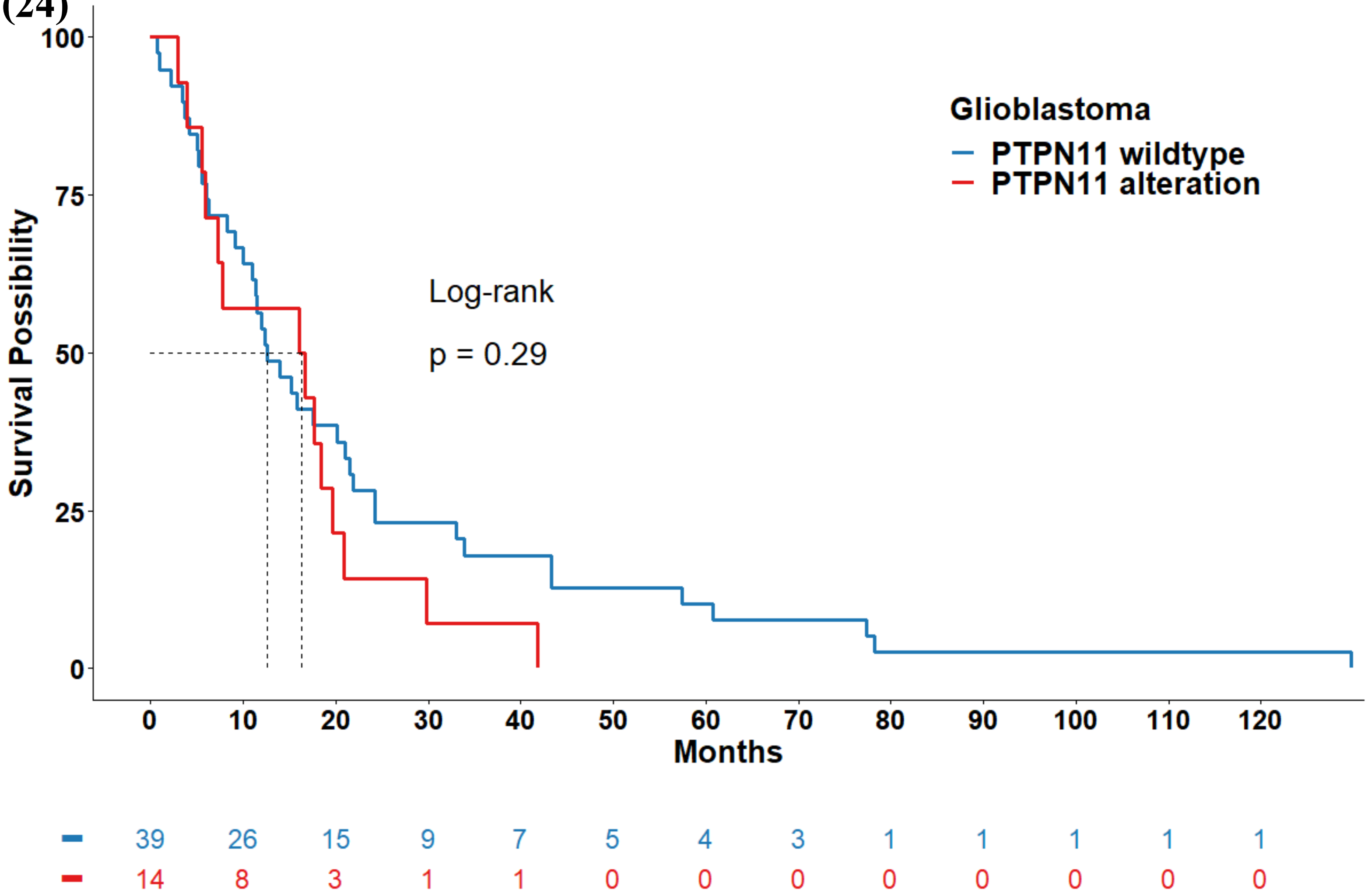

Survival Possibility
100
75
50
25
0
Glioblastoma
PTPN11 wildtype
PTPN11 alteration
Log-rank
p = 0.29
0
10
20
30
40
50
60
70
80
90
100
110
120
Months
39 26 15 9 7 5 4 3 1 1 1 1 1
14 8 3 1 1 0 0 0 0 0 0 0 0

(25)

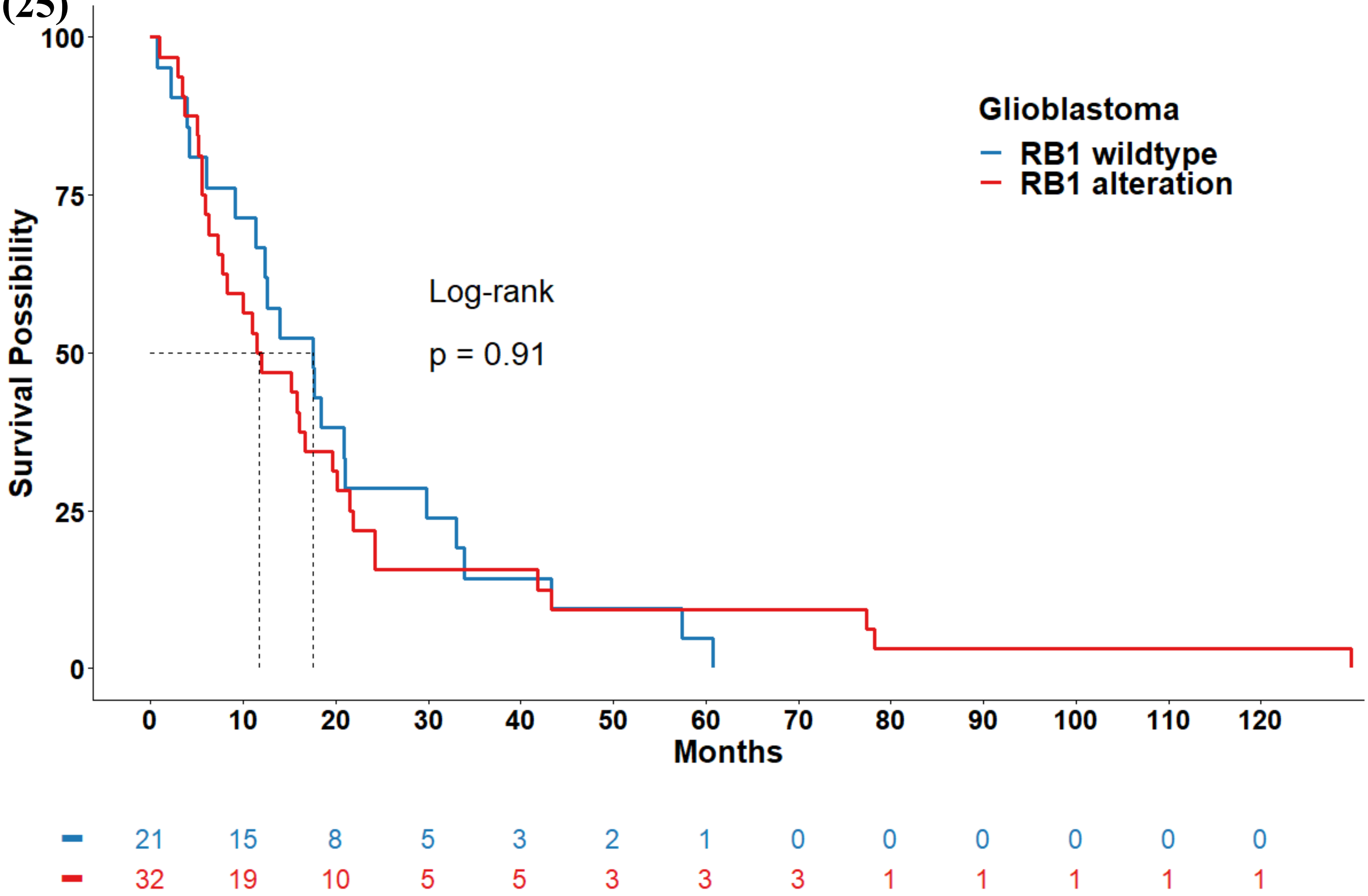

Glioblastoma
RB1 wildtype
RB1 alteration
Survival Possibility
100
75
50
25
0
Log-rank
p = 0.91
0
10
20
30
40
50
60
70
80
90
100
110
120
Months
21 15 8 5 3 2 1 0 0 0 0 0 0
32 19 10 5 5 3 3 3 1 1 1 1 1

(26)

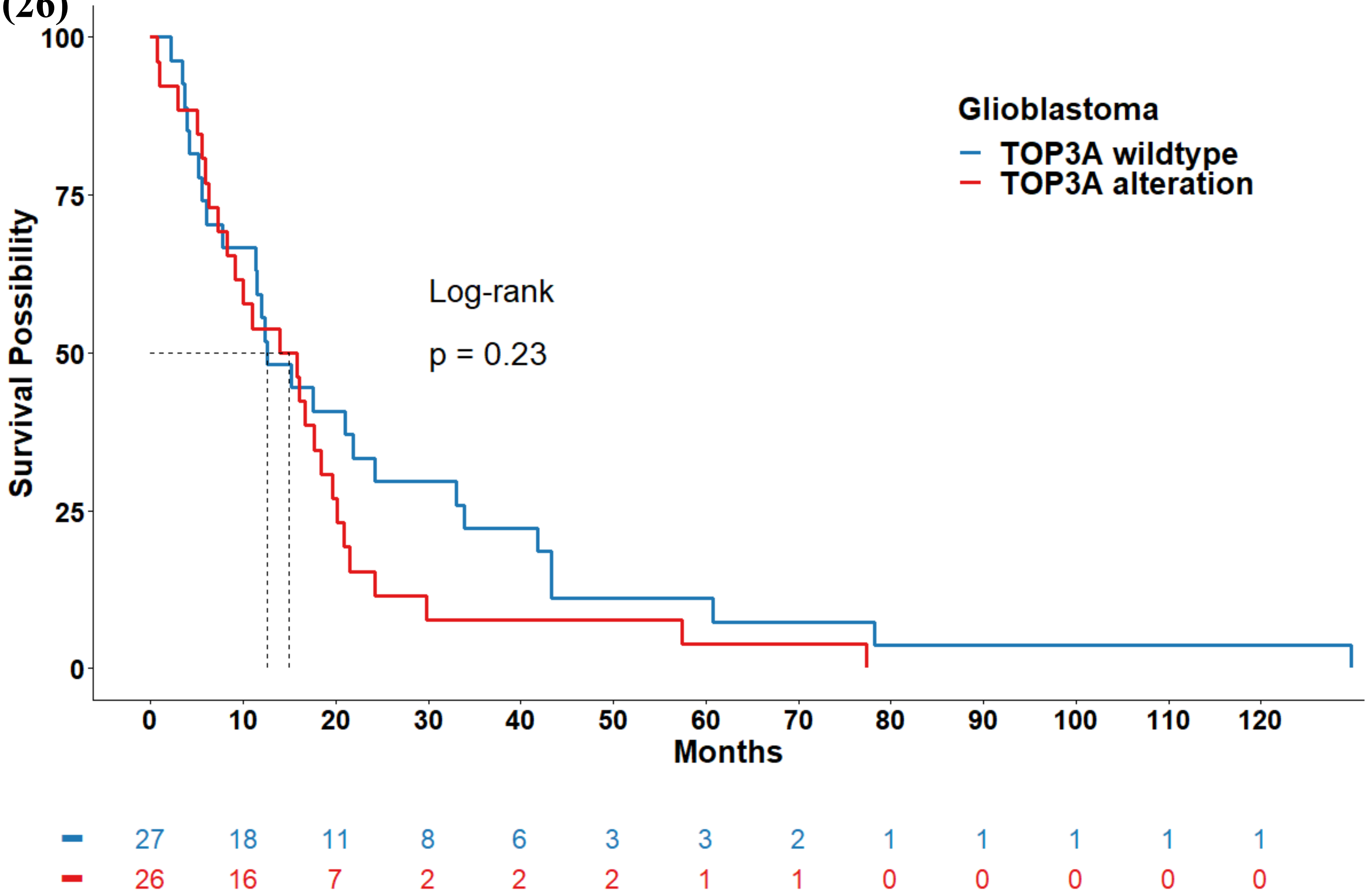

Survival Possibility
100
75
50
25
0
Log-rank
p = 0.23
Glioblastoma
TOP3A wildtype
TOP3A alteration
0
10
20
30
40
50
60
70
80
90
100
110
120
Months
27 18 11 8 6 3 3 2 1 1 1 1 1
26 16 7 2 2 2 1 1 0 0 0 0 0

(27)

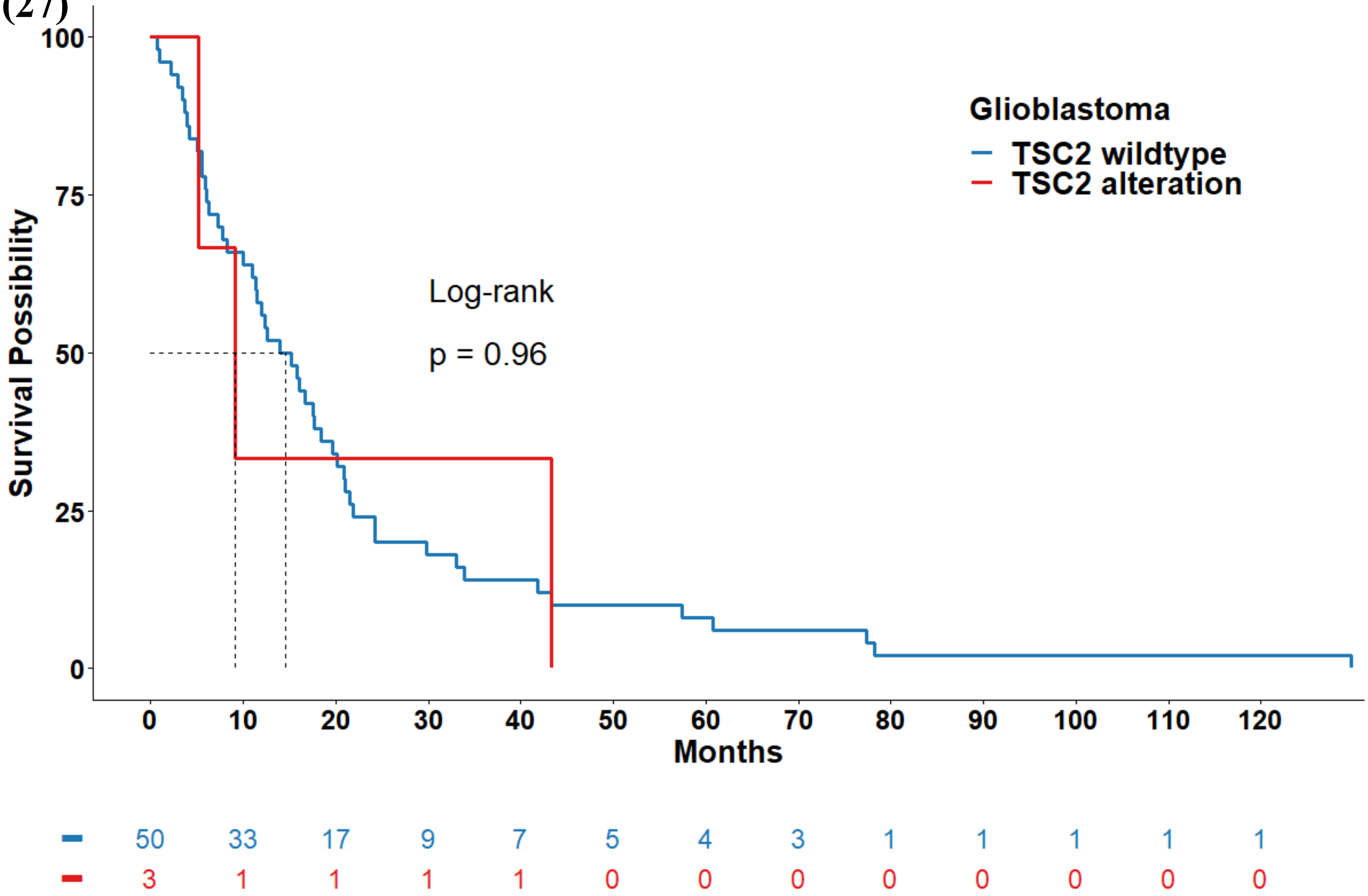

Glioblastoma
TSC2 wildtype
TSC2 alteration
Log-rank
p = 0.96
Survival Possibility
100
75
50
25
0
0 10 20 30 40 50 60 70 80 90 100 110 120
Months
50 33 17 9 7 5 4 3 1 1 1 1 1
3 1 1 1 1 0 0 0 0 0 0 0 0
